# Evolution of Symbiotic Bacteria in the Distal Human Intestine 

**DOI:** 10.1371/journal.pbio.0050156

**Published:** 2007-06-19

**Authors:** Jian Xu, Michael A Mahowald, Ruth E Ley, Catherine A Lozupone, Micah Hamady, Eric C Martens, Bernard Henrissat, Pedro M Coutinho, Patrick Minx, Philippe Latreille, Holland Cordum, Andrew Van Brunt, Kyung Kim, Robert S Fulton, Lucinda A Fulton, Sandra W Clifton, Richard K Wilson, Robin D Knight, Jeffrey I Gordon

**Affiliations:** 1 Center for Genome Sciences, Washington University School of Medicine, St. Louis, Missouri, United States of America; 2 Genome Sequencing Center, Washington University School of Medicine, St. Louis, Missouri, United States of America; 3 Department of Molecular, Cellular and Developmental Biology, University of Colorado, Boulder, Colorado, United States of America; 4 Department of Computer Science, University of Colorado, Boulder, Colorado, United States of America; 5 Universités Aix-Marseille I and II, Marseille, France; 6 CNRS, UMR6098, Marseille, France; 7 Department of Chemistry and Biochemistry, University of Colorado, Boulder, Colorado, United States of America; University of California Davis, United States of America

## Abstract

The adult human intestine contains trillions of bacteria, representing hundreds of species and thousands of subspecies. Little is known about the selective pressures that have shaped and are shaping this community's component species, which are dominated by members of the Bacteroidetes and Firmicutes divisions. To examine how the intestinal environment affects microbial genome evolution, we have sequenced the genomes of two members of the normal distal human gut microbiota, Bacteroides vulgatus and *Bacteroides distasonis,* and by comparison with the few other sequenced gut and non-gut Bacteroidetes, analyzed their niche and habitat adaptations. The results show that lateral gene transfer, mobile elements, and gene amplification have played important roles in affecting the ability of gut-dwelling Bacteroidetes to vary their cell surface, sense their environment, and harvest nutrient resources present in the distal intestine. Our findings show that these processes have been a driving force in the adaptation of Bacteroidetes to the distal gut environment, and emphasize the importance of considering the evolution of humans from an additional perspective, namely the evolution of our microbiomes.

## Author Summary

The total number of microbes that colonize the surfaces of our adult bodies is thought to be ten times greater than the total number of our human cells. Our microbial partners provide us with certain features that we have not had to evolve on our own. In this sense, we should consider ourselves to be a supraorganism whose genetic landscape includes both our own genome as well as the genomes of our resident microbes, and whose physiologic features are a synthesis of human and microbial metabolic traits. The largest collection of microbes resides in our gut, which harbors trillions of bacteria, representing hundreds of species, most falling into two groups—the Bacteroidetes and the Firmicutes. We have sequenced the genomes of two human gut-dwelling Bacteroidetes, and compared their genomes to the genomes of other bacteria that live both inside and outside of our bodies. Our results illustrate that adaptation to the gut habitat is a dynamic process that includes acquisition of genes from other microorganisms. These findings emphasize the importance of including the evolution of “our” microbial genomes when considering the evolution of humans.

## Abstract 3

Human microbiome evolution was explored by comparing human gut Bacteroidete genomic sequences to available data; common modes of evolution were revealed that have enabled these gut-dwelling microbes to adapt to their environments.

## Introduction

Our distal gut is one of the most densely populated and most thoroughly surveyed bacterial ecosystems in nature. This microbiota contains more bacterial cells than all of our body's other microbial communities combined. The gut microbial community and its collective genome (microbiome) endow us with physiological attributes that we have not had to evolve on our own, including the ability to break down otherwise indigestible polysaccharides [1,2]. The most complete 16S rRNA gene sequence–based enumerations available indicate that more than 90% of phylogenetic types (phylotypes) belong to just two of the 70 known divisions of Bacteria, the Bacteroidetes and the Firmicutes, with the remaining phylotypes distributed among eight other divisions [3]. With an estimated 500–1,000 species, and over 7,000 strains [4], the evolutionary tree of our distal intestinal microbiota can be visualized as a grove of ten palm trees (divisions), each topped by fronds representing divergent lineages, and with each frond composed of many leaves representing closely related bacteria [1]. In contrast, soil, Earth's terrestrial “gut” for degrading organic matter, can be viewed as a bush, composed of many more intermediate and deeply diverging lineages [5].

It is unclear how selective pressures, microbial community dynamics, and the environments in which we live shape the genomes and functions of members of our gut microbiota, and hence our “micro-evolution.” Ecological principles predict that functional redundancy encoded in genomes from divergent bacterial lineages ensures against disruption of food webs. These principles also predict that host-driven, “top-down” selection for such redundancy should produce a community composed of distantly related members, whose genomes convergently evolve functionally *similar* suites of genes [4]. Lateral gene transfer (LGT), which allows for rapid transfer of genes under strong selection, such as the genes encoding antibiotic resistance [6], represents one way that members of the microbiota could share metabolic and other capabilities. In contrast, competition between members of a microbiota should exert a “bottom-up” selective pressure that produces specialized genomes with functionally *distinct* suites of genes. These distinct suites define ecological niches (professions), and once established, could be maintained by barriers to homologous recombination [4].

To explore whether and how these principles apply to the gut microbiota and its microbiome, we have determined the complete genome sequences of two Bacteroidetes with highly divergent 16S rRNA phylotypes that are prominently represented in the distal gut of healthy humans—Bacteroides vulgatus and Bacteroides distasonis (now also known as Parabacteroides distasonis [7]). *B. distasonis* is basal to the *Bacteroides* clade, and diverged from the common ancestor of the other *Bacteroides* prior to their differentiation. The results of comparisons with other sequenced gut- and non-gut–associated Bacteroidetes, described below, provide insights into the evolution of niche specialization in this highly competitive ecosystem, including the role of LGT.

## Results

### Functional Categorization of Genomic Adaptations to the Distal Human Gut Habitat

The 5,163,189–base pair (bp) genome of the human gut-derived B. vulgatus type strain ATCC 8482 encodes a predicted 4,088-member proteome, whereas the 4,811,369-bp genome of B. distasonis type strain ATCC 8503 possesses 3,867 predicted protein-coding genes (Figure S1 and Table S1). These genomes were initially compared to the genomes of two other Bacteroidetes that live in the distal human gut: B. thetaiotaomicron (type stain ATCC 29148 [2] and B. fragilis (strains YCH 46 and NCTC 9343 [8,9]). We identified 1,416 sets of orthologous protein-coding genes shared among these gut Bacteroidetes; 1,129 (79.7%) of these conserved gene sets were assigned to Clusters of Orthologous Groups (COGs; see Figure S2 and Table S2 for a COG-based categorization). The two most prominently represented COG categories in each of the gut-associated Bacteroidetes proteomes are G (carbohydrate transport and metabolism) and M (cell wall/membrane/envelope biogenesis). The two most prominent COG categories in their shared proteome are E (amino acid transport and metabolism) and J (translation, ribosomal structure, and biogenesis) (Figure S2).

**Figure 1:**
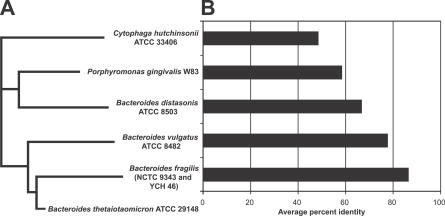
Phylogenetic Relationships of Fully Sequenced Bacteroidetes (A) The 16S rRNA sequences were taken from a previously published alignment created using the NAST aligner [60]. A maximum-likelihood tree was generated using parameters estimated with ModelTest 3.7 and PAUP* (version 4.0b10). Terminal branch lengths are not drawn to scale. (B) The average percent amino acid sequence identities were calculated using ClustalW alignments for the 530 sets of seven-way orthologs that include the five intestinal Bacteroidetes genomes, *P. gingivalis,* and *Cy. hutchinsonii. B. thetaiotaomicron* was used as a reference.

The average pairwise amino acid–sequence identity among the shared orthologs was 82.0% for *B. thetaiotaomicron–B. fragilis,* 72.1% for B. thetaiotaomicron–*B. vulgatus,* 62.1% for B. thetaiotaomicron–*B. distasonis,* and 61.7% for *B. vulgatus–B. distasonis*. These values are consistent with the 16S rRNA phylogenetic tree for Bacteroidetes (Figure 1). Although the evolution of these gut Bacteroidetes is characterized by comprehensive deterioration of global synteny (Figure S3), a total of 257 “patches” of local synteny were identified, composed of adjacent orthologous genes encompassing 765 of the 1,416 shared orthologs (54%; average of 3.0 orthologs per cluster).

The distal gut microbiota is exposed to several prominent nutrient sources: (1) dietary plant polysaccharides that are not digested in the small intestine by the host because our human proteome lacks the requisite glycoside hydrolases and polysaccharide lyases (see the Carbohydrate Active Enzymes database [CAZy] at http://www.cazy.org for a comprehensive annotation of the human “glycobiome”), (2) undigested plant proteins [10], and (3) host glycans associated with the continuously renewing epithelium that lines the gut and with the even more rapidly replenished mucus layer that overlies this epithelium.

To identify genomic features related to adaptation to life within this distal human gut habitat, we compared shared orthologs among all five completely sequenced gut Bacteroidetes genomes to the subset that is shared with the two Bacteroidetes that occupy non-gut habitats. These non-gut Bacteroidetes are Porphyromonas gingivalis W83, a member of the human oral microbiota [11], and Cytophaga hutchinsonii ATCC 33406, which is found in soil (http://www.ncbi.nlm.nih.gov/entrez/query.fcgi?db=genomeprj&cmd=Retrieve&dopt=Overview&list_uids=54). Each proteome was searched for conserved domains. These domains were used to assign a functional identifier (InterPro ID) that was then mapped onto Gene Ontology (GO) terms [12] using InterProScan [13]. The results were compiled and statistical comparisons made between the number of genes assigned to each GO term in different genomes. The complete list of GO assignments for all seven Bacteroidetes genomes is available at http://rd.plos.org/pbio.0050156 (5.3 MB).

**Figure 2:**
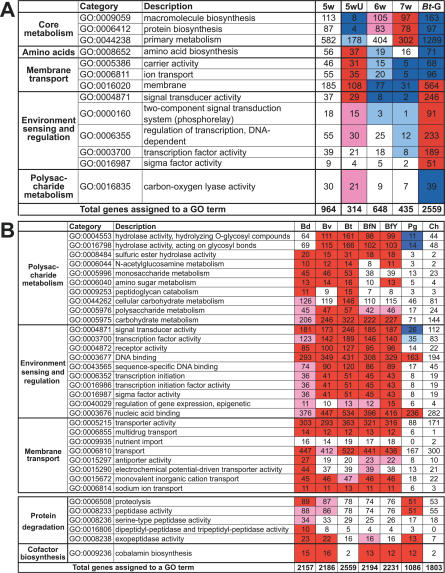
Sensing, Regulatory, and Carbohydrate Metabolism Genes Are Enriched among All Gut-Associated Bacteroidetes The number of genes assigned to each GO term from each genome is shown. Significant enrichment is denoted by pink (*p* < 0.05) or red (*p* < 0.001), whereas depletion is indicated by light blue (*p* < 0.05) or dark blue (*p* < 0.001), as calculated by a binomial comparison followed by Benjamini-Hochberg false-discovery rate correction (see Materials and Methods). (A) Genes assigned to GO terms related to core metabolic functions are enriched in the subset of common gut-associated Bacteroidetes orthologs shared with non-gut Bacteroidetes (seven-way comparison; abbreviated 7w), compared to the reference set of 1,416 orthologs common to the five sequenced gut Bacteroidetes genomes (5w), suggesting that all Bacteroidetes have inherited a core metabolome from their common ancestor. The set of orthologs that is not shared with non-gut–associated Bacteroidetes (five-way unique; 5wU) is enriched, relative to all orthologs (5w), for genes in three classes—amino acid biosynthesis; membrane transport; and two-component signal transduction systems—suggesting that these genes were important in the process of adaptation to the gut and/or other habitats by the common ancestor of gut Bacteroidetes. (B) Various GO terms related to environmental sensing, gene regulation, and carbohydrate degradation are enriched in gut Bacteroidetes relative to *Cy. hutchinsonii* (Ch)*.* A similar pattern is observed relative to P. gingivalis (Pg) (unpublished data). Note that these same classes of genes are depleted in the subset of shared gut Bacteroidetes orthologs ([A] 5w) relative to the full B. thetaiotaomicron (Bt) genome ([A] Bt-G). Thus, these classes of genes, though enriched in all gut Bacteroidetes, are widely divergent between them. Other classes of genes vary between species: B. distasonis (Bd) and B. vulgatus (Bv) show an expanded repertoire of proteases, whereas B. thetaiotaomicron lacks genes involved in synthesis of cobalamin. 6w refers to the orthologs shared by the five sequenced gut Bacteroidetes genomes (Bt, Bv, Bd, plus two B. fragilis strains [NCTC 9343 (BfN) and YCH 46 (BfY)] and Pg.

The subset of orthologs *shared* with non-gut Bacteroidetes is enriched for core metabolic activities, suggesting that all Bacteroidetes have inherited a core metabolome from their common ancestor (Figure 2A, compare data in column 7w versus data in 5w). The subset of orthologs *unique* to the gut Bacteroidetes is enriched for genes related to amino acid biosynthesis, membrane transport, carbon-oxygen lyases, and environment sensing/regulation (see GO terms highlighted in red/pink in the column labeled 5wU in Figure 2A). Furthermore, although a comparison of each gut-dwelling Bacteroidetes proteome to the proteomes of its non-gut relatives (Figure 2B) revealed that the four gut species are all enriched for genes that belong to GO categories related to three general functions—(1) polysaccharide metabolism, (2) environmental sensing and gene regulation, and (3) membrane transport—most of these GO categories are depleted among the subset of orthologs that are unique to the gut-associated Bacteroidetes (Figure 2A, 5w versus Bt-G). Thus, even though all four sequenced gut Bacteroidetes species have increased numbers of genes in categories (1) through (3), this analysis suggests that each species has evolved a divergent array of sensing, regulatory, and polysaccharide degradation genes that augment the core metabolome they share with other members of their division.

### Niche Specialization of Bacteroidetes

To further define the niches occupied by the gut Bacteroidetes, we compared each one to B. thetaiotaomicron. B. thetaiotaomicron was selected as the reference species because there is a wealth of information about its functional attributes. Scanning electron microscopy, whole-genome transcriptional profiling, and mass spectrometry–based metabolomic studies performed in gnotobiotic mice colonized with this prominent human gut symbiont have shown that B. thetaiotaomicron is a remarkably flexible forager for polysaccharides that opportunistically deploys different subsets of its 209 paralogs of SusC and SusD (two outer membrane proteins involved in the binding and import of starch and maltooligosaccharides [14,15]), and its 226 predicted glycoside hydrolases plus 15 polysaccharide lyases, so that it can feast on dietary or host mucus glycans, depending upon the polysaccharide content of the host's diet [16] (Table S1).

Compared to the other Bacteroidetes, the B. thetaiotaomicron proteome has the most glycoside hydrolases known or predicted to degrade plant glycans (e.g., 64 arabinosidases; our human proteome has none), and the most enzymes for harvesting host glycans (e.g., sulfuric ester hydrolases, hexosaminidases, and fucosidases) (Figure 2B and Table S3). It is also the only sequenced gut Bacteroidetes that possesses candidate polysaccharide lyases for degrading animal tissue glycans (e.g., heparin, chondroitin, and hyaluronan; Table S3). *B. thetaiotaomicron'*s ability to opportunistically use many glycan sources likely makes it an important generalist among intestinal Bacteroidetes.

Compared to *B. thetaiotaomicron, B. distasonis* is a specialist. It has the smallest genome among the sequenced human gut-associated Bacteroidetes, the smallest repertoire of genes that are members of the environmental sensing and gene regulation GO categories, and the smallest number of genes associated with carbon source degradation (Figure 2B and Table S1). B. distasonis lacks many accessory hemicellulases (arabinosidases, α-glucuronidases), pectinases, and other polysaccharidases that target non-plant carbohydrates, such as chitinases. Moreover, the number of genes present in each CAZy enzyme class represented in its proteome is markedly reduced compared to the other intestinal Bacteroidetes (e.g., B. distasonis has only one candidate α-fucosidase, whereas the other gut-associated species have nine or ten) (Table S3).

B. distasonis has two classes of carbohydrate-processing enzymes that are more abundant in its proteome than in the proteomes of other gut Bacteroidetes: CAZy glycoside hydrolase family 13 (α-amylase–related proteins), and family 73 (N-acetylhexosaminidases, which can target host glycans as well as bacterial cell walls). Its proteome also contains more polysaccharide deacetylases (seven versus four in *B. thetaiotaomicron,* and one to two in the B. fragilis strains, as characterized by InterPro ID IPR002509; see http://rd.plos.org/pbio.0050156.a for a complete list of InterPro ID assignments). Host epithelial glycans contain O-acetylated sugars, including sialic acids, that protect them from direct cleavage by microbial glycoside hydrolases. Thus, B. distasonis has the capacity to make the deacetylated products available for itself and other components of the microbiota. Finally, B. distasonis devotes a greater proportion of its genome to protein degradation than does B. thetaiotaomicron (GO:0006508, “proteolysis”; *p* < 0.0003 by binomial test; Figure 2B).

The B. vulgatus glycobiome has features consistent with ex vivo studies indicating that its substrate range for polysaccharides is intermediate between that of B. distasonis and B. thetaiotaomicron [17]. B. vulgatus has the largest and most complete complement of enzymes that target pectin, a common fruit-associated class of glycans (includes pectin methylesterases, pectin acetylesterases, polygalacturonases, and accessory δ-4,5 unsaturated glucuronyl hydrolases). According to the CAZy classification scheme, B. vulgatus is the only sequenced gut Bacteroidetes with a gene encoding a xylanase (Bv0041c). Together, these findings reveal overlapping, but distinct, niches among these gut Bacteroidetes. We next examined the role of LGT in shaping their genomes.

### Lateral Gene Transfer

Determining whether a gene is laterally transferred is widely acknowledged to be a difficult problem (e.g., [18–21] and Text S1). We chose a phylogenetic approach (see Materials and Methods) to identify genes that appeared to have been laterally acquired and probably selected for after the divergence of individual gut species. Our approach could potentially identify two types of genes: genes that were laterally transferred only into one lineage, and genes that were lost in all lineages except one. We also had to acknowledge the possibility that random errors in phylogenetic tree reconstruction could produce false-positive results. We confirmed that LGT was the more likely scenario for these genes by demonstrating that they differed in composition from the rest of the genome. This approach allowed us to investigate the adaptations of individual lineages to their specific niche. For simplicity, we refer to these genes as “laterally transferred” in the remainder of this study, although a minority of them may actually represent differential gene loss, which would still likely indicate species-specific selection [22].

Our approach was to use sensitive, iterated profile searches to retrieve homologs of each protein-coding gene in the genomes of interest from publicly available databases. We then built phylogenetic trees of the related sequences and bootstrapped them to minimize noise, keeping only those nodes that were supported by 70% of the replicates. We subsequently used the National Center for Biotechnology Information (NCBI) taxonomy database to assign taxonomy information to each sequence, and employed the Fitch parsimony algorithm [23] to assign the most likely bacterial taxon to each internal node. This analysis allowed us to differentiate four classes of genes: (1) those whose closest relatives are outside the gut Bacteroidetes, suggesting a lateral transfer event and/or differential gene loss; (2) those whose closest relative is within the gut Bacteroidetes, indicating likely vertical inheritance; (3) those without any homologs in the database (i.e., “novel”); and (4) those whose pattern of inheritance, whether lateral or vertical, could not be determined (i.e., “unresolved”). Parsimony was used to assign a likely direction (“in” or “out”) to each lateral transfer event where possible (see Table S4 and Materials and Methods).

We did not attempt to resolve lateral transfer events within the gut Bacteroidetes in this study, primarily because the lack of sufficient taxonomic sampling within the Bacteroidetes made it impossible to distinguish transfer from biased sampling. Previous studies have observed that a number of novel genes in other bacterial genomes seem to be laterally acquired [24]. However, for the purposes of our functional analyses, these novel genes were excluded because little functional information is available about them. Because we wished to analyze adaptation to the gut, we also excluded genes that appeared to have been transferred out of the Bacteroidetes.

Our method identified an average of 5.5% of the genes in each genome as being laterally transferred from outside the gut Bacteroidetes (312 for *B. distasonis,* 184 for *B. vulgatus,* 277 for *B. thetaiotaomicron,* 199 for B. fragilis NCTC 9343, 214 for B. fragilis YCH 46, and 103 for P. gingivalis). We verified that the genes we identified as “laterally transferred” differed from those classified as “not transferred,” both in terms of GC content (*p* < 0.0001 for each genome by two-tailed *t*-test using Welch's correction for unequal variances) and codon bias (*p* < 0.0001 for each genome by chi-square test). These results, together with the functions represented by this class of genes (see below), confirm that LGT is the most likely scenario accounting for these genes, although we cannot rule out ancient paralogs from the data available because of different rates and patterns of evolution in different lineages, and other confounding factors.

**Figure 3:**
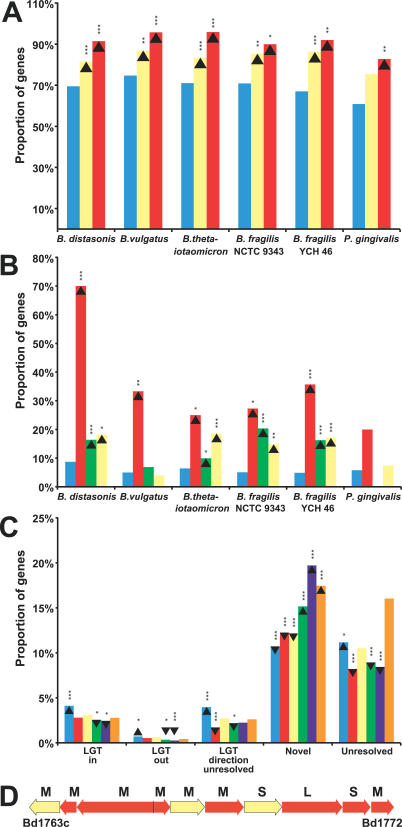
Analyses of Lateral Gene Transfer Events in Bacteroidetes Lineages Reveal Its Contribution to Niche Specialization (A) Genes involved in core metabolic processes are enriched among non-laterally transferred genes identified by a phylogenetic approach (see Materials and Methods). The proportion of genes identified as not laterally transferred in each genome (light blue), as well as assigned to the GO terms “Primary metabolism” (yellow) and “Protein biosynthesis” (red), are shown. Significant increases (enrichment) relative to each whole genome are shown by an upward-pointing arrowhead, and decreases (depletion) by a downward-pointing arrowhead, whereas the corresponding probability, determined by a binomial test, is denoted by asterisks: a single asterisk (*) indicates *p* < 0.05; double asterisks (**) indicate *p* < 0.01; and triple asterisks (***) indicate *p* < 0.001. (B) Laterally transferred genes are enriched among genes assigned to the GO term “DNA methylation” (e.g., restriction-modification systems) (red), relative to each complete genome (light blue). Glycosyltransferases (yellow) and genes located within *CPS* loci (green) are also enriched within the set of transferred genes. Significance was determined and denoted as in (A). (C) B. distasonis (light blue) possesses a significantly larger proportion of laterally transferred genes than the other Bacteroidetes, as shown by significant increases in the proportion of genes in each category of our analysis (“LGT in,” laterally transferred into the genome; “Novel,” no homologs identified from other species; “LGT direction unresolved,” laterally transferred but direction unknown; “LGT out,” laterally transferred out of the genome; and “Unresolved,” lateral transfer uncertain; see Materials and Methods for detailed explanations of categories and http://rd.plos.org/pbio.0050156.a for a complete list of genes in each category). Significant changes, denoted as in (A), were determined by a binomial test, using the average proportion within all other genomes used in the analysis as the reference. Other strains are B. vulgatus (red), *B. thetaiotaomicron* (yellow), *B. fragilis* NCTC 9343 (green), *B. fragilis* YCH 46 (purple), and *P. gingivalis* (orange). (D) A prominent laterally transferred locus within B. distasonis contains a ten-gene hydrogenase complex, likely allowing B. distasonis to use hydrogen as a terminal electron acceptor in anaerobic respiration. Genes transferred into B. distasonis are colored red, whereas genes whose phylogeny could not be resolved are shown in yellow. Letters indicate functional components of the hydrogenase complex: L, large subunit; M, maturation or accessory factor; and S, small subunit.

A complete classification of laterally transferred protein-coding genes in the gut Bacteroidetes, and *P. gingivalis,* is provided in Table S4. Genes involved in core cellular processes, such as translation (e.g., ribosomal proteins), are less susceptible to LGT than other genes [25]. Primary metabolism (GO:0044238) and protein biosynthesis (GO:0006412) are among the GO terms most enriched in the set of genes *not* subject to LGT (Figure 3A). These results suggest that our criteria exclude many genes that would be expected not to undergo LGT. In contrast, genes that are known to be subject to LGT, such as restriction-modification systems [26–28], were enriched in the set of laterally transferred genes we detected (Figure 3B).

B. distasonis has a significantly larger proportion of laterally transferred genes than the other gut Bacteroidetes (Figure 3C). This excess of LGT does not correlate with a larger number of identifiable mobile elements: B. distasonis has fewer of the integrases and transposases that can catalyze the insertion of foreign DNA than do the other Bacteroidetes, and similar numbers of phage (five versus two to five for the other species; see Table S1). The excess of LGT genes in B. distasonis is also not simply attributable to its more distant phylogenetic relationship to the other gut Bacteroidetes, because P. gingivalis does not share this feature (Figure 3C). Instead, B. distasonis has a striking elevation in the proportion of DNA methylation proteins classified as laterally transferred. Seventy percent of genes classified as “DNA methylation” (GO:0006306; e.g., restriction-modification systems) are predicted to be laterally transferred, even though B. distasonis has fewer DNA methylation genes overall (ten versus an average of 11.5 for other gut Bacteroidetes; Figure 3C). The combination of a smaller number of restriction-modification systems, together with their acquisition from unrelated bacteria, would be expected to reduce the barriers to LGT by allowing B. distasonis to acquire genes from those bacteria. These laterally acquired genes may contribute to the success of B. distasonis within the gut habitat. For example, among the set of transferred genes is a ten-gene hydrogenase complex (Figure 3D), which would allow B. distasonis to use hydrogen as a terminal electron acceptor.

### The Role of Lateral Gene Transfer in the Evolution of Capsular Polysaccharide Biosynthesis Loci

Capsular polysaccharide biosynthesis *(CPS)* locus expression and the functional importance of capsular structural variation have been best characterized in *B. fragilis.* For example, studies in gnotobiotic mice indicate that the zwitterionic capsular polysaccharide from one *B. fragilis CPS* locus *(PSA)* is presented by intestinal dendritic cells, resulting in expansion of CD4^+^ T cells, induction of IFNγ production by the T helper 1 subtype (Th1), and reversal of the T helper 2 (Th2) bias found in the absence of colonization. The result is a balanced Th1/Th2 cytokine profile that should help promote co-existence with a microbiota and, perhaps, tolerance to a variety of environmental antigens, including those found in food [29].

B. vulgatus has nine *CPS* loci, whereas B. distasonis has 13. Like B. thetaiotaomicron (eight *CPS* loci) and B. fragilis (nine each in strains NCTC 9343 and YCH 46), each *CPS* cluster is composed of a pair of linked upstream UpcY and UpcZ homologs that act as a “regulatory cassette,” and downstream genes encoding glycosyltransferases, carbohydrate transporters, and other proteins that form a “structural cassette” (Table S5).

Among gut-associated Bacteroidetes, we found that glycosyltransferases and genes in *CPS* loci are enriched for laterally transferred genes (Figure 3B). *P. gingivalis,* in contrast, does not show a biased representation of lateral transfer within its set of glycosyltransferases, suggesting that laterally acquired genes serve an important function in providing new genetic material for the rapid divergence of these loci in gut Bacteroidetes.

*CPS* loci are among the most polymorphic sites in the four gut-associated Bacteroidetes species [30,31]. A comparison of the two sequenced B. fragilis genomes [8,9] revealed that the genome-wide synteny evident in the two closely related B. fragilis strains is disrupted in eight of their nine *CPS* loci (Figure S4 and Table S6).

### Conjugative Transposons, Phage, and Other Mechanisms Involved in Promoting *CPS* Diversity

#### Conjugative transposons.

**Figure 4:**
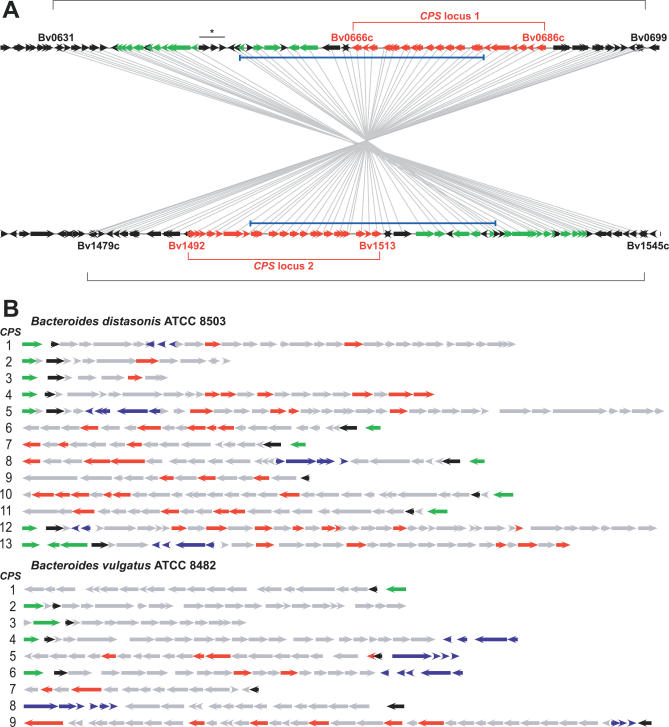
Evolutionary Mechanisms That Impact Bacteroidetes *CPS* Loci (A) CTn-mediated duplication of *B. vulgatus CPS* loci. Homologous gene pairs in the two duplicated regions are linked with fine gray lines, underscoring the high level of synteny. Genes constituting *CPS* loci 1 and 2 are highlighted in red, with the first and last genes numbered. Green denotes essential component genes of CTns. Blue brackets indicate two subregions that share 100% nucleotide sequence identity. The asterisk (*) indicates three open reading frames encoding two conserved hypothetical proteins and a hypothetical protein, suggesting an insertion that occurred after the duplication event. (B) Locations of putative glycosyltransferase xenologs and inserted phage genes in *CPS* loci of the sequenced gut Bacteroidetes. Color code: integrases (green), UpxY transcriptional regulator homologs (black), putative xenologs (primarily glycosyltransferases, red), phage genes (blue), and remaining genes (gray). See Table S5 for functional annotations.

We observed that conjugative transposons (CTns) are associated with the duplication of *CPS* loci within a genome. In *B. vulgatus,* Bv0624–Bv0699 (75,747 bp) is a copy of another region (Bv1479c–Bv1560, 75,277 bp) (Figure 4A and Table S5). Each copy contains a CTn followed by a complete *CPS* locus. The average amino acid sequence identity of the 64 homologous gene pairs comprising the repeated regions is 90%. Two exact 28,411-bp copies harbor a major portion of the structural cassettes of these duplicated *CPS* loci, plus part of a CTn (Figure 4A). The strict nucleotide-level sequence conservation in coding and non-coding sequences suggests a recent homologous recombination event at the structural cassettes of the *CPS* loci. There is also evidence that the function of *CPS* loci can be disrupted by CTns, as in *CPS* locus 8 of B. fragilis YCH 46 where an α-1,2-fucosyltransferase gene is interrupted by a 127-kilobase (Kb), 132-gene CTn (Table S5).

**Figure 5:**
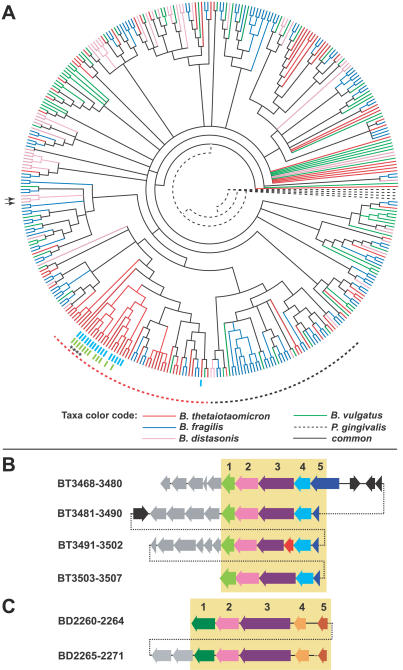
Cladogram Comparison of SusC/SusD Pairs Shows Both Specialized and Shared Branches among the Bacteroidetes (A) Cladogram generated from all fully sequenced Bacteroidetes. Branches that are unique to each species are color-coded as indicated. The homologous RagA/RagB proteins from P. gingivalis were selected as an arbitrary root (dashed branches). Dashed lines surrounding the tree indicate (1) a clade that is dominated by B. thetaiotaomicron SusC/SusD pairs (39/45 pairs, red dashes) and (2) a clade that is poorly represented in B. thetaiotaomicron (7/34 pairs, black dashes). Colored hash marks surrounding the cladogram represent the linkage of two other protein families, which show syntenic organization within related B. thetaiotaomicron SusC/SusD-containing loci: NHL repeat–containing proteins (light blue) and a group of conserved hypothetical lipidated proteins (light green). These protein families are not represented in the other sequenced Bacteroidetes, occur only adjacent to SusC/SusD pairs, and have no predicted functions. See http://rd.plos.org/pbio.0050156.a for locus tags for each taxon, branch bootstrap values, and lists of SusC/SusD-linked genes. (B) An example of a recently amplified polysaccharide utilization locus in which the synteny of three flanking SusC/SusD genes has been maintained. The locations of the four SusC/SusD pairs encoded within these amplified clusters are indicated on the cladogram shown in (A) by asterisks (*). The locus schematic is arranged so that groups of related proteins (mutual best BLAST hits) are aligned vertically within the yellow box. The functions of amplified genes are indicated by numbers over each vertical column and, where applicable, are color coded to correspond to (A): 1, conserved hypothetical lipidated protein; 2, SusD paralog; 3, SusC paralog, 4, NHLrepeat–containing protein; and 5, glutaminase A (note that in three clusters, this gene has been partially deleted). Gray-colored genes downstream of each amplified cluster encode hypothetical proteins or predicted enzymatic activities (e.g., dehydrogenase, sulfatase, and glycoside hydrolase) that are unique to each cluster. A xenolog that has been inserted in one gene cluster is indicated in red; other genes are black. Dashed lines connecting gene clusters show linkage only, and do not correspond to actual genomic distance. (C) An example of a recently duplicated locus from B. distasonis that includes duplicated regulatory genes. Syntenic regions are aligned as in (B) and include a single sulfatase (1, dark green), a SusD paralog (2, light purple), SusC paralog (3, dark purple), an anti-σ factor (4, light orange), and an ECF-σ factor (5, dark orange). Two other downstream sulfatase genes (gray) are also included in one cluster. The locations of the two SusC/SusD pairs encoded within these clusters are indicated on the cladogram shown in (A) by black arrows.

#### Phages.

Phages also appear to modulate *CPS* locus function. In *B. distasonis, CPS* locus 5 contains a block of five genes inserted between its regulatory cassette and genes encoding carbohydrate biosynthetic enzymes. This inserted segment, oriented in the opposite direction to the upstream regulatory *UpxY* (and *UpxZ*) genes and downstream carbohydrate biosynthetic genes, consists of a homolog of phage T7 lysozyme (N-acetylmuramoyl-L-alanine amidase) followed by four genes encoding hypothetical proteins. Three more *B. distasonis CPS* loci each harbor a block of these genes (two to five genes per block; each block with a similar orientation; only the T7 lysozyme is conserved among all copies of the putative phages; Figure 4B and Table S5).

B. distasonis is the only sequenced type strain in which a phage disrupts *CPS* loci between their regulatory cassettes and structural cassettes. B. vulgatus has five copies of this phage, all associated with *CPS* loci. B. thetaiotaomicron has ten copies, only two of which are associated with *CPS* loci, whereas the B. fragilis strains each have one (Table S5).

#### Phase variation.

LGT, CTn-mediated duplication and translocation of *CPS* loci, and disruption of *CPS* loci by phage appear to operate in combination with at least two other mechanisms to promote the rich diversity of surface glycan structures in Bacteroidetes. In *B. fragilis,* a serine site-specific recombinase (Mpi) regulates expression of seven of its eight *CPS* loci through phase variation (DNA inversion) at *CPS* promoters [32]. *B. vulgatus, B. distasonis,* and B. thetaiotaomicron have Mpi orthologs (one, three, and one, respectively). In addition, five of the nine *CPS* loci in *B. vulgatus,* 11 of the 13 *CPS* loci in *B. distasonis,* four of the eight *CPS* loci in *B. thetaiotaomicron,* and only one of the ten *CPS* loci in B. fragilis NCTC 9343 have a gene encoding a tyrosine-type site-specific recombinase immediately upstream of a *UpxY* homolog. This juxtaposition suggests that inversions of some *CPS* loci may be subjected to local as well as global regulation. Such sequence inversions were observed in the assemblies of the B. vulgatus and B. distasonis genomes (unpublished data).

#### Fkp and fucose utilization.

B. fragilis can also alter *CPS* glycan composition by means of Fkp, a protein whose N-terminus is homologous to mammalian L-fucose-1-P-guanyltransferase and whose C-terminus is similar to L-fucose kinases. Fkp generates GDP-L-fucose from exogenous L-fucose; fucose from GDP-L-fucose can be incorporated into *CPS* glycan structures, thereby linking L-fucose availability in the organism's intestinal habitat to *CPS* capsular structure [33]. Although Fkp is highly conserved in *B. distasonis, B. vulgatus, B. thetaiotaomicron,* and *B. fragilis,* their L-fucose acquisition and utilization capacities are not. *B. distasonis, B. vulgatus, B. thetaiotaomicron,* and B. fragilis all possess α-fucosidases for harvesting L-fucose, which is a common component of host mucus and epithelial cell glycans. In B. thetaiotaomicron and *B. fragilis,* a complete fucose utilization system is incorporated into a gene cluster *(fucRIAKXP)*. In *B. vulgatus,* this gene cluster (Bv1339c–Bv1341c) contains an ortholog of *B. thetaiotaomicron'*s L-fucose–inhibited repressor (R), fucose isomerase (I), and fucose permease (P), but not L-fuculose-1-phosphate aldolase (A) or L-fuculose kinase (K). B. distasonis lacks all elements of this gene cluster.

### The Role of Gene Duplication in Diversification of Gut Bacteroidetes: A Case Study of SusC/SusD Paralogs

As noted above, the gut Bacteroidetes genomes contain large numbers of paralogs involved in environmental sensing and nutrient acquisition. We used one of the largest families, the SusC/SusD paralogs (Table S1), as a model for investigating relationships among members. SusC paralogs are predicted to be TonB-dependent, β-barrel–type outer membrane proteins. Thus, in addition to binding nutrients such as polysaccharides, SusC paralogs likely participate in their energy-dependent transport into the periplasmic space [34]. SusD paralogs are predicted to be secreted and to have an N-terminal lipid tail that would allow them to associate with the outer membrane [14]. Genes encoding SusC and SusD paralogs are typically positioned adjacent to one another in the B. thetaiotaomicron genome (102 of 107 loci encoding SusC paralogs), and are often part of multigene clusters that also encode enzymes involved in carbohydrate metabolism (62 of 107 loci) [2]. Eighteen of the 62 clusters that encode SusC/SusD paralogs and glycoside hydrolases also contain ECF-σ factors and adjacent anti-σ factors. A subset of SusC paralogs contain an extra N-terminal domain with homology to the N-terminal domain of the Escherichia coli FecA iron-dicitrate receptor protein [35]. FecA interacts directly with an anti-σ factor (FecR) via this domain, thereby controlling gene expression through modulation of its associated ECF-σ factor (FecI).

These clusters provide case studies of the evolution of gut Bacteroidetes genomes. Their glycoside hydrolase content varies considerably within a given species (Table S7). Our studies in B. thetaiotaomicron indicate that ECF-σ factors are required for transcription of their adjacent polysaccharide utilization gene clusters, and that chromosomally linked anti-σ factors act as repressors of this transcription. Moreover, several B. thetaiotaomicron loci containing ECF-σ and anti-σ factors are differentially regulated during growth on various complex glycans ([16]; E. C. Martens and J. I. Gordon, unpublished data), suggesting that these systems act as components of carbohydrate sensors responsible for regulating loci appropriate for utilizing available nutrients.

Six of these clusters in B. distasonis (clusters 2–6 and 16 in Table S7-A) include predicted sulfatases, whereas there are fewer such loci in the other genomes: two clusters in B. vulgatus (clusters 5 and 11 in Table S7-B), four in *B. thetaiotaomicron,* and three in each of the two *B. fragilis* strains. These enzymes could be involved in the desulfation of sulfomucins that contain galactose-3-sulfate, galactose-6-sulfate, and *N*-acetylglucosamine-6-sulfate residues. These or other sulfatases could also be involved in the desulfation of glycosaminoglycans such as chondroitin and heparin.

To explore the role of gene duplication in the diversification of the Bacteroidetes, we generated lists of all paired SusC and SusD paralogs from the four gut- and one non-gut–associated Bacteroidetes species (see Materials and Methods). P. gingivalis has four such pairs, whereas the other five intestinal Bacteroidetes species have a total of 370 (Table S1). A cladogram generated from the multiple sequence alignment shows that many SusC/SusD pairs have close relatives among several Bacteroidetes. However, certain specialized groups are unique to each species, with B. thetaiotaomicron containing one particularly large expansion (Figure 5A). Gene clusters encoding related SusC/SusD pairs also contain other genes that are closely related to one another. The homology and synteny of these loci suggest that genomic duplication is a mechanism driving their amplification and diversification (e.g., Figure 5B and 5C). An intriguing feature of some of these amplified loci is that they contain clusters of genes with unique functions that are located downstream of the “core” duplicated genes; this may serve to further diversify the roles of these loci in nutrient acquisition (e.g., Figure 5B in which diverse dehydrogenase, sulfatase, and glycoside hydrolase functions are included downstream of a syntenic core of amplified genes).

## Discussion

The trillions of bacteria that colonize our distal gut largely belong to two bacterial divisions, and can be classified by 16S rRNA gene sequence analysis into hundreds of “species” that share a common ancestry [4,36], but whose genome content may vary considerably. Forces that shape the genome content of bacteria in the gut include the intermicrobial dynamics of competition and cooperation in resource partitioning that shape complex food webs, as well as other community-shaping forces, such as phage attacks, that can result in “selective sweeps” that remove cells with similar susceptibilities. In a competitive environment in which innovation in resource acquisition strategies can breed success, and resistance to phage can mean surviving a phage selective sweep, bacteria can be expected to differentiate their genome content. For the host to thrive and produce more gut habitats (by reproducing), the gut microbial ecosystem must be functionally stable over time despite the internal dynamics of the community. The constituent bacteria might therefore be expected to have a high degree of functional redundancy between species, so that the loss of one lineage would not adversely impact ecosystem services to the host. Our investigation of the genomes of human gut Bacteroidetes species shows that the “top-down” forces imposed by selection at the host level that would result in a homogenized microbiome, and the “bottom-up” forces of intermicrobial dynamics that would result in completely differentiated genomes, are both at work in the distal gut. The genomes of the gut Bacteroidetes species that have been sequenced harbor suites of genes with similar functions, but differ in the number of genes within functional categories and their specific sequence. It appears that the differences between genomes are enough to carve out specific niches within the gut habitat, such that the species are not in direct competition, but are sufficiently similar to confer resistance to disturbance to the host through functional redundancy.

Our findings demonstrate a key role for LGT in shaping the adaptation of individual Bacteroidetes to distinct niches within the human gut. It is unclear how and when laterally transferred genes were introduced during evolution of distal gut Bacteroidetes. We have performed 16S rRNA gene sequence–based enumeration studies of the fecal microbiota of 59 different mammalian species: the results reveal that none of the four sequenced gut Bacteroidetes species is restricted to the human gut (R. E. Ley and J. I. Gordon, unpublished data). Nonetheless, the impact of LGT is likely profound for these gut symbionts and their human hosts. A large and varied gene pool of glycosyltransferases provides a capacity for diversification of surface polysaccharide structures that could endow symbionts with varied capacities to shape a host immune system so that it can accommodate a microbiota (and perhaps related food and other environmental antigens). Because the environment surrounding each human being varies, this gene flow may promote the generation of host-specific microbiomes. Acquisition of new types of carbohydrate binding proteins, transporters, and degradation enzymes through both LGT and gene amplification should influence the types of substrates that can be exploited for energy harvest. It may also affect our predisposition to conditions such as obesity in which the efficiency of caloric harvest may be influenced by the relationship between an individual's microbial glycoside hydrolase repertoire and the glycan content of his/her diet [37,38].

These considerations emphasize the need to have a more comprehensive view of our genetic landscape as a composite of human and microbial genes, a transcendent view of human evolution as involving our microbial partners, and a commitment to investigating human biology in the larger framework of environmental microbiology. Attention to these issues is timely, given the onset of efforts to sequence the human “microbiome” [39]. These metagenomic studies will allow investigators to address new, but fundamental, questions about humans. Do we share an identifiable core “microbiome”? If there is such a core, how does the shell of diversity that surrounds the core influence our individual physiologic properties? How is the human microbiome evolving (within and between individuals) over varying time scales as a function of our changing diets, lifestyle, and biosphere? Finally, how should we define members of the microbiome when microbes possess pan-genomes (all genes present in any of the strains of a species) with varying degrees of “openness” to acquisition of genes from other microbes?

## Materials and Methods

### Genome sequencing.

The B. vulgatus and B. distasonis genomes were assembled from two types of whole-genome shotgun libraries: a plasmid library with an average insert size of 5 Kb, and a fosmid library with an average insert size of 40 Kb. For each genome, both Phrap (http://www.phrap.org/) and PCAP [40] assemblies were generated and then compared, resulting in a “hybrid” assembly that takes advantage of the strength of both assemblies. Regions that contained a gap in one assembly, but not in the other, were made contiguous in the final assembly for finishing by using Consed [41].

Sequence gaps were filled by primer-walking on spanning clones. Physical gaps were amplified by PCR and closed by sequencing the PCR products. Poor quality regions were detected using Consed, amplified with PCR, and resequenced. The integrity and accuracy of the assembly were verified by clone constraints. Regions of lower coverage, or that contained ambiguous assemblies, were resolved by sequencing spanning individual fosmids. Regions that underwent sequence inversions were identified based on inconsistency of constraints for a fraction of read pairs in those regions. The final assemblies consisted of 12.6X and 13.2X sequence coverage for B. vulgatus and *B. distasonis,* respectively. For each base, the Phred quality value was at least 40.

rRNA and tRNA genes were identified with BLASTN and tRNA-Scan [42], respectively. Protein-coding genes were identified using GLIMMER v.2.0 [43], ORPHEUS v.2.0 [44], and CRITICA v.0.94h [45]. WUBLAST (http://blast.wustl.edu) was used to identify all predicted proteins with significant hits to the non-redundant (nr) database. Predicted protein-coding genes containing fewer than 60 codons and without significant homology (e-value threshold of 10^−6^) to other proteins were eliminated. Gene start-site predictions were fine-tuned using MED-Start [46] and BLAST homology. In general, no overlapping genes were allowed. Potential frameshift errors were identified by sequence alignment with known proteins, and confirmed or corrected by resequencing. The final set of genes, compiled from the analysis described above, was manually curated. Protein annotation was based on homology searches against public databases and domain analysis with HMMER (http://selab.janelia.org/). Functional classification was based on homology searches against COG categories using WU-BLAST and COGnitor [47], followed by manual curation. Metabolic pathways were constructed with reference to KEGG [48]. Phage genes were identified using Prophage finder [49].

### Functional comparisons.

Orthologs of the five intestinal Bacteroidetes genomes were identified based on (1) mutual BLASTP best hits with an e-value threshold of 10^−6^ and (2) a requirement that each pairwise protein alignment cover at least 60% of query length in both search directions. The amino acid sequences of each set of orthologs were aligned using ClustalW [50] and processed with Gblocks [51].

*CPS* loci in the five intestinal Bacteroidetes genomes were defined with the following criteria. First, an intact *CPS* locus included a *UpxY* homolog (as annotated) and a number of downstream genes on the same strand. These downstream genes included those that encoded functions related to surface polysaccharide synthesis (such as glycosyltransferases, carbohydrate export proteins, epimerases, glycoside hydrolases, etc.), conserved hypothetical proteins, or hypothetical proteins. Second, the 5′ boundary of each locus was determined by the *UpxY* homolog. Third, the 3′ end of each locus generally was positioned where switch of coding strand occurred. Alternatively, the 3′ end of the locus was positioned where downstream genes on the same strand encoded functions that were defined, but unrelated, to capsular polysaccharide synthesis (e.g., rRNA/tRNA and two-component signaling systems). However, the 3′ end of the locus was extended if the coding strand was disrupted by a single hypothetical protein (to accommodate possible annotation errors), or a mobile element composed of one or multiple genes.

GO categories and InterPro ID were assigned using InterProScan release 12.1 [13]. The number of genes in each genome assigned to each GO term, or its parents in the hierarchy (according to the ontology description available as of June 6, 2006 [12]), were totaled. All terms assigned to at least ten genes in a given genome were tested for overrepresentation, and all terms with a total of ten genes across all tested genomes were tested for underrepresentation. Significantly over- and underrepresented genes were identified using a binomial comparison with the indicated reference set. To control for differences in the specificity of gene prediction, genes that could not be assigned to a GO category were excluded from the reference sets. A correction was then applied to each distinct set of tests (e.g., over- or underrepresentation in a genome) to achieve a false discovery rate of 0.05 for each set [52]. These tests were implemented using the Math::CDF Perl module (E. Callahan, Environmental Statistics, Fountain City, Wisconsin; available at http://www.cpan.org/), and scripts written in Perl.

### 16S rRNA phylogeny.

Phylogenetic trees were constructed based on alignment of 16S rRNA fragments using the NAST aligner [53]. The alignment was filtered using a Lane mask, then modeled using ModelTest 3.7 [54]: a maximum-likelihood tree was found by an exhaustive search using PAUP* (v. 4.0b10, http://paup.csit.fsu.edu/) employing parameters estimated by ModelTest.

### Laterally transferred genes.

*Overview of strategy used to identify LGT.* See Text S1.

*Identifying classes of genes that were potentially laterally transferred or otherwise under selection in the gut Bacteroidetes.* We identified genes that were laterally acquired and probably selected for after the divergence of gut Bacteroidetes species, and thus potentially involved in niche differentiation. These genes could either have been transferred into an individual species by LGT, or retained in that species despite being lost in all other related sequenced species. It is difficult, perhaps impossible, to distinguish these two cases using the tree topology alone. We identified this class of genes by determining whether each gene met one of the following criteria. (1) No homologs were found in an augmented NCBI non-redundant (nr) protein database (plus the proteins from the newly sequenced strains). This case indicated that either the gene has been lost in every other sequenced organism but retained in this genome, or that the gene was laterally transferred from an organism that is not represented in the database. (2) The only homologs found were from the same species. This case is the same as case (1), except that either the gene was sequenced multiple times and deposited in the database under separate records, or there are paralogs, i.e., multiple copies of the gene in the genome being analyzed. Both case (1) and (2) were termed “novel.” (3) The only homologs found are either from the same species or from other divisions or non-gut Bacteroidetes. This case indicates that the sequence is in this genome, and also in the genome of distantly related organisms, but not in the closely related gut Bacteroidetes genomes that have been completely sequenced. This case also provides evidence that the gene was either transferred or retained despite loss in related organisms. (4) The gene is more closely related to genes from other divisions or to non-gut Bacteroidetes than it is to other gut Bacteroidetes that are in the tree, and parsimony analysis indicates that the direction of transfer was into rather than out of the genome. This pattern is most consistent with LGT, although differential gene loss cannot in principle be ruled out (however, differences in composition between this class of genes and the rest of the genome provide compelling supporting evidence). Both cases (3) and (4) above were termed “laterally transferred” (LGT).

*Genomes.* We carried out the analysis on six different genomes: Bacteroides vulgatus ATCC 8482 and Bacteroides distasonis ATCC 8503, Bacteroides fragilis NCTC 9343 (NC_0023338), Bacteroides fragilis YCH 46 (NC_006347), Bacteroides thetaiotaomicron ATCC 29148 (NC_004663), and Porphyromonas gingivalis W83 (NC_002950).

*Finding homologs.* For each gene in each genome, we identified potential homologs using PSI-BLAST against NCBI's nr protein database. In order to use all of the available data for the Bacteroidetes and their relatives, we augmented this database with proteins predicted by Glimmer (v. 2.0) from draft genomes in the Bacteroidetes group that were available at NCBI. These additional genomes included Prevotella ruminicola 23 (The Institute for Genomic Research; TIGR; http://www.tigr.org), Prevotella intermedia 17 (TIGR), Pelodictyon phaeoclathratiforme BU-1 (Deptment of Energy–Joint Genome Institute [DOE-JGI]; http://www.jgi.doe.gov), Pelodictyon luteolum DSM 273 (DOE-JGI), Chlorobium phaeobacteroides DSM 266 (DOE-JGI), Chlorobium limicola DSM 245 (DOE-JGI), Chlorobium chlorochromatii CaD3 (DOE-JGI), B. forsythus (TIGR), and B. fragilis 638R (Wellcome Trust Sanger Institute; http://www.sanger.ac.uk).

To find the top BLAST hits using the most stringent e-value threshold possible, we used a multistep PSI-BLAST. In the first PSI-BLAST iteration, we used an e-value threshold of 10^−50^ or lower. If fewer than 50 hits were found, we used the hits to make a profile for a subsequent PSI-BLAST that was four orders of magnitude less stringent (i.e., with an e-value of 10^−46^). We repeated this procedure, increasing the e-value by a factor of 10^−4^ at each iteration, until either 50 hits were found or, after 12 iterations, the maximum allowed e-value of 10^−6^ was reached. To remove from consideration sequences that were significant only because of a conserved domain rather than similarity over the whole gene, we excluded genes that differed from the length of the query sequence by more than 30%. We also omitted hits that contained gaps greater than 50 amino acids in length or that contained gaps at greater than 50% of the positions after performing a multiple sequence alignment with the other sequences in the set.

*Making phylogenetic trees.* We performed multiple sequence alignment using MUSCLE [55], omitting sequences that were poorly aligned to the query sequence as described above. We used this alignment to make a neighbor-joining tree using ClustalW [50]. We used bootstrapping to collapse nodes that were not statistically supported. Specifically, we randomly resampled columns from the alignment 100 times and made new neighbor-joining trees with ClustalW. We collapsed into polytomies all nodes in the original tree that were recovered in fewer than 70% of the bootstrap replicates.

*Assigning taxonomy information to sequences.* We parsed the NCBI taxonomy database and used it to assign division and genus information for each PSI-BLAST hit in the phylogenetic tree. Sequences that could not be assigned to any particular division were removed from the tree. We also removed nematode and arthropod genomes because we found that these often provided close hits to the Bacteroidetes genomes. We expect that these bacteria-to-eukaryote hits actually arise because gut and/or salivary gland bacteria contaminated the DNA preparations used for genomic sequencing. We also used the genus annotations in the taxonomy to determine whether sequences from the Bacteroidetes division were from the gut. We assigned sequences as gut Bacteroidetes if they were in the genera *Prevotella, Porphyromonas, Tannerella, Dysgonomonas,* or *Bacteroides,* and as non-gut Bacteroidetes otherwise.

*Finding genes that are laterally transferred or differentially lost (under recent selective pressure).* We used the bootstrap neighbor-joining trees to identify genes that met any of the four criteria described above. We first marked genes “novel” if the PSI-BLAST protocol returned only the query gene, indicating that they met criterion (1), or if all of the genes in the tree were from the same species, indicating that they met criterion (2). We assigned each sequence to a species using the NCBI taxonomy. If genes from other species were present in the tree, we used the following algorithm. (1) Start at the query sequence. (2) Step back in tree until a bootstrap-supported node containing sequences from a different species is found. If this node has, as descendants, sequences from other gut Bacteroidetes only, mark the gene as not laterally transferred (not selected for). If the node has, as descendants, sequences from both gut Bacteroidetes and other divisions or non-gut Bacteroidetes, mark the gene as unresolved. If the node has, as descendants, sequences from other divisions or non-gut Bacteroidetes only, mark the gene as laterally transferred (selected for) and proceed to the parsimony analysis. (3) Use parsimony analysis to determine whether a potential transfer would have been into the Bacteroidetes species (indicating that it is important for the gut), or out of the Bacteroidetes species into another lineage. Assign division information to all internal nodes in the tree using the Fitch parsimony algorithm [23]. These assignments minimize the number of transfers between divisions needed to explain the distribution of divisions in the modern sequences. If the query sequence is surrounded by many sequences from unrelated divisions, the parsimony analysis will indicate that the most likely event was a transfer into the species. As noted in Text S1 (Overview of Strategy Used to Identify LGT)**,** the method we used provides an automated technique for assigning taxon labels to individual gene trees. Specifically, we treat each taxon label (division labels, “gut Bacteroidetes” or “non-gut Bacteroidetes”) as a character state, and use the Fitch parsimony algorithm [23] to infer the ancestral state at each node. We are not using this method in the sense of a formal evolutionary model of taxon switching, but as a heuristic that recaptures the intuition that a phylogenetic tree with a clade leading to sequences from one taxon that sprouts from within a clade leading to sequences from a completely different taxon probably represents a LGT event, even if the inner clade is represented by more sequences. This type of strategy has been widely applied both manually and computationally to detect lineage-specific transfers (e.g., [56–58]), and is related to a method used in studies of host–parasite co-speciation [59], a problem that is mathematically equivalent to LGT detection.

### SusC/SusD alignments.

Pairs of genes encoding SusC and SusD paralogs were identified in the Bacteroidetes genome sequences by performing individual BLASTP searches against each genome using amino acid sequences of previously annotated SusC and SusD paralogs as queries. The low-scoring hits from each search (e-values between 10^−4^ and 10^−10^) were themselves used as BLASTP queries to reveal more divergent putative paralogs in each genome. This process was repeated until no new paralogs were identified. Lists of putative SusC and SusD paralogs were compared for each species. Paralogs were included in subsequent ClustalW analysis based on the requirement that each had a separately predicted, adjacent partner. This process was instrumental in excluding related TonB-dependent hemin, vitamin B_12_ and iron-siderophore receptors from the list of putative SusC paralogs. The resulting dataset included 374 paralog pairs: 102 in *B. thetaiotaomicron,* 69 in B. fragilis NCTC 9343, 65 in B. fragilis YCH 46, 80 in *B. vulgatus,* 54 in *B. distasonis,* and four in P. gingivalis. Because polysaccharide binding by SusC and SusD has been shown to require both polypeptides [14], and because individual SusC and SusD alignments suggested these paired functions have evolved in parallel (unpublished data), each pair was joined into a single sequence prior to alignment. Sequences were aligned using ClustalW [50] (version 1.83), and a neighbor-joining cladogram was created from the alignment using PAUP* (v. 4.0b10, http://paup.csit.fsu.edu/). Bootstrap values were determined from 100 trees. Branches retained in Figure 5A represent groups with 70% or greater bootstrap values.

## Supporting Information

Figure S1 B. distasonis ATCC 8503 and B. vulgatus ATCC 8482 Chromosomes The B. distasonis ATCC 8503 chromosome is shown in (A), and the B. vulgatus ATCC 8482 chromosome is shown in (B). The coding potential of the leading and lagging strands is relatively unbiased. Circles shown in the figure represent, from inside out, GC skew, GC content variation, rRNA operons, tRNA genes, conjugative transposons (CTns), *CPS* loci, extracytoplasmic function (ECF)-σ factors, SusC paralogs, and all predicted genes with assigned functions on reverse and forward strands, respectively. Color codes for genes are based on their COG functional classification.
(2.3 MB PDF)

Figure S2 COG-Based Characterization of All Proteins with Annotated Functions in the Proteomes of Sequenced Bacteroidetes The term “Bacteroides orthologs” refers to the 1,416 orthologs shared by the sequenced gut Bacteroidetes (*B. vulgatus, B. distasonis, B. thetaiotaomicron,* plus the two B. fragilis strains). Color codes are the same as Figure S1.
(334 KB PDF)

Figure S3 Pairwise Alignments of the Human Gut Bacteroidetes Genomes Reveal Rapid Deterioration of Global Synteny with Increasing Phylogenetic Distance Each data point on the Dotplot represents one pair of mutual best hits (BLASTP) between the two genomes, plotted by pairwise genome location. Diagonal lines indicate synteny.
(822 KB PDF)

Figure S4 *CPS* Loci Are the Most Polymorphic Regions in the Gut Bacteroidetes Genomes High-resolution synteny map of *CPS* loci and flanking regions in the two sequenced B. fragilis strains**.** There are nine *CPS* loci in each genome. Each data point represents a pair of orthologs (mutual best hits; e-value cutoff: 10^−6^). Brackets define the coordinates for component genes within a given locus (some pairs are missing due to gene loss or gain): *x*-axis, coordinate of the middle point of the gene on the NCTC 9343 chromosome; *y*-axis, coordinate of the middle point of the gene on YCH 46 chromosome. With the exception of *CPS* locus 5, which is strictly conserved, the nine *CPS* loci are affected by nonhomologous gene replacement and rearrangement.
(1.0 MB PDF)

Table S1 Comparison of Genome Parameters for B. distasonis ATCC 8503, B. vulgatus ATCC 8482, B. thetaiotaomicron ATCC 29148, B. fragilis NCTC 9343, and B. fragilis YCH 46 An asterisk (*) indicates the numbers of SusC/SusD homologs provided are based on BLASTP e-value equal to 10^−20^or lower; the numbers shown in parentheses are based on criteria described in *SusC/SusD alignments* in Materials and Methods. See http://rd.plos.org/pbio.0050156.a for complete lists of SusC/SusD homologs. A hybrid two-component system protein contains all of the domains present in classical two-component systems, but in one polypeptide [50].
(92 KB PDF)

Table S2 Shared Orthologs in B. distasonis ATCC 8503, B. vulgatus ATCC 8482, B. thetaiotaomicron ATCC 29148, and B. fragilis Strains NCTC 9343 and YCH 46 For an explanation of COG-based functional codes, see Figure S1.
(277 KB PDF)

Table S3 Glycoside Hydrolases Found in B. distasonis ATCC 8503, B. vulgatus ATCC 8482, B. thetaiotaomicron ATCC 29148, and B. fragilis Strains NCTC 9343 and YCH 46 The classification scheme used is described in the Carbohydrate-Active enZYme (CAZy) database.
(70 KB PDF)

Table S4 List of Putative Xenologs in B. distasonis ATCC 8503, B. vulgatus ATCC 8482, B. thetaiotaomicron ATCC 29148, B. fragilis NCTC 9343, and B. fragilis YCH 46 The putative xenologs are listed for B. distasonis ATCC 8503 (A), B. vulgatus ATCC 8482 (B), B. thetaiotaomicron ATCC 29148 (C), B. fragilis NCTC 9343 (D), and B. fragilis YCH 46 (E). For an explanation of COG-based functional codes, see Figure S1. The lateral gene transfer (LGT) column defines the predicted evolutionary history of the coding sequence: LGT-in, laterally transferred into the genome; LGT-out, laterally transferred out of the genome; and LGT-unresolved, laterally transferred but direction unknown. See Materials and Methods for detailed explanations.
(447 KB PDF)

Table S5 *CPS* Loci of B. distasonis ATCC 8503, B. vulgatus ATCC 8482, B. thetaiotaomicron ATCC 29148, B. fragilis NCTC 9343, and B. fragilis YCH 46 Shown are Gene ID, annotated function, GC content (%), and the predicted evolutionary history of the coding sequence for B. distasonis ATCC 8503 (A), B. vulgatus ATCC 8482 (B), B. thetaiotaomicron ATCC 29148 (C), B. fragilis NCTC 9343 (D), and B. fragilis YCH 46 (E).
LGT-in, laterally transferred into the genome; LGT-out, laterally transferred out of the genome; LGT-unresolved, laterally transferred but direction unknown; NO, not laterally transferred; NOVEL, no homologs found in any other genomes in public databases; UNRESOLVED, whether laterally transferred or not is not resolved. See Materials and Methods for detailed explanations. Color codes are the same as in Figure 4B.
(509 KB PDF)

Table S6 *CPS* Loci Are among the Most Polymorphic Regions in the Two B. fragilis Genomes The *p*-value is based on the tail probability of a binomial distribution. Gene loss/gain events (3,531 in total) are counted as the difference between the total number of genes and the total number of genes shared between the two genomes.
(61 KB PDF)

Table S7 ECF-σ Factor–Containing Polysaccharide Utilization Gene Clusters in B. distasonis ATCC 8503 and B. vulgatus ATCC 8482 B. distasonis ATCC 8503 is shown in (A), and B. vulgatus ATCC 8482 is shown in (B). The three columns represent Gene ID, functional annotation, and predicted evolutionary history of the gene (labeled as in Table S5).
(184 KB PDF)

Text S1 Overview of Strategy Used to Identify Lateral Gene Transfer (148 KB DOC)

### Accession Numbers

The genome sequences of B. vulgatus ATCC 8482 and B. distasonis ATCC 8503 have been deposited in GenBank (http://www.ncbi.nlm.nih.gov/Genbank) under accession numbers CP000139 and CP000140, respectively.

## Abbreviations

bp: base pair
CAZy: Carbohydrate Active Enzymes database
COG: Clusters of Orthologous Groups
CPS: capsular polysaccharide synthesis
CTn: conjugative transposon
GO: Gene Ontology
LGT: lateral gene transfer
NCBI: National Center for Biotechnology Information
