# Supplementary material for: Evolution of Symbiotic Bacteria in the Distal Human Intestine
Source: PLoS Biol. 2007 Jun 19;5(7):e156. doi: 10.1371/journal.pbio.0050156 (PMC1892571; doi:10.1371/journal.pbio.0050156)
Supplement: Supplementary file 1 [file pbio.0050156.sg001.pdf]

FIGURE S1A

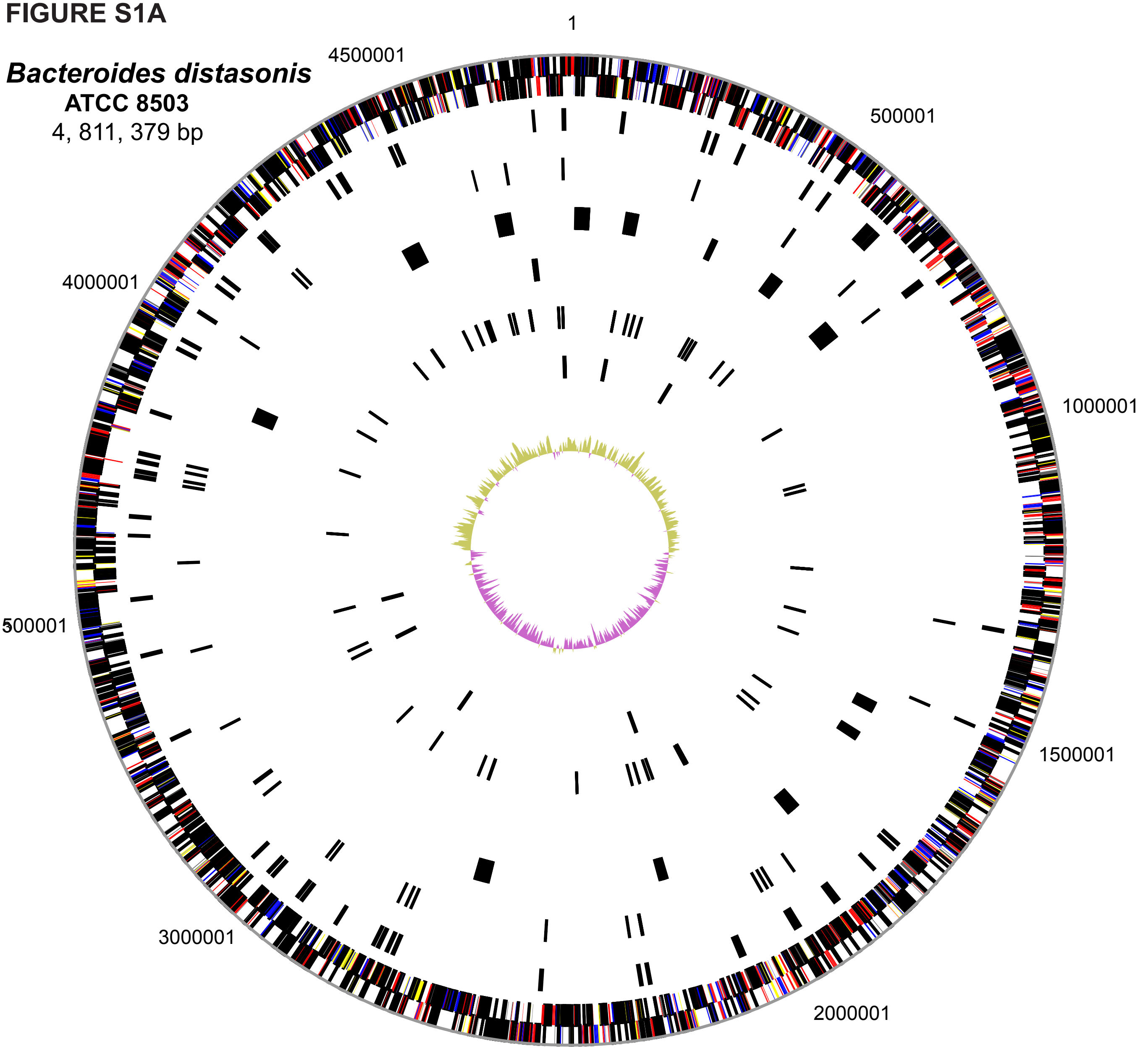

COG functional category

- M Cell wall/membrane/envelope biogenesis
- G Carbohydrate transport and metabolism
- L Defense mechanisms
- V Replication, recombination and repair
- C Energy production and conversion
- D Cell division, chromosome partitioning
- E Amino acid transport and metabolism
- F Nucleotide transport and metabolism
- H Coenzyme transport and metabolism
- I Lipid transport and metabolism
- J Translation, ribosomal structure and biogenesis
- K Transcription
- O Posttranslational modification, protein turnover, chaperones
- P Inorganic ion transport and metabolism
- Q Secondary metabolites biosynthesis, transport and catabolism
- T Signal transduction mechanisms
- U Intracellular trafficking, secretion, and vesicular transport

FIGURE S1B

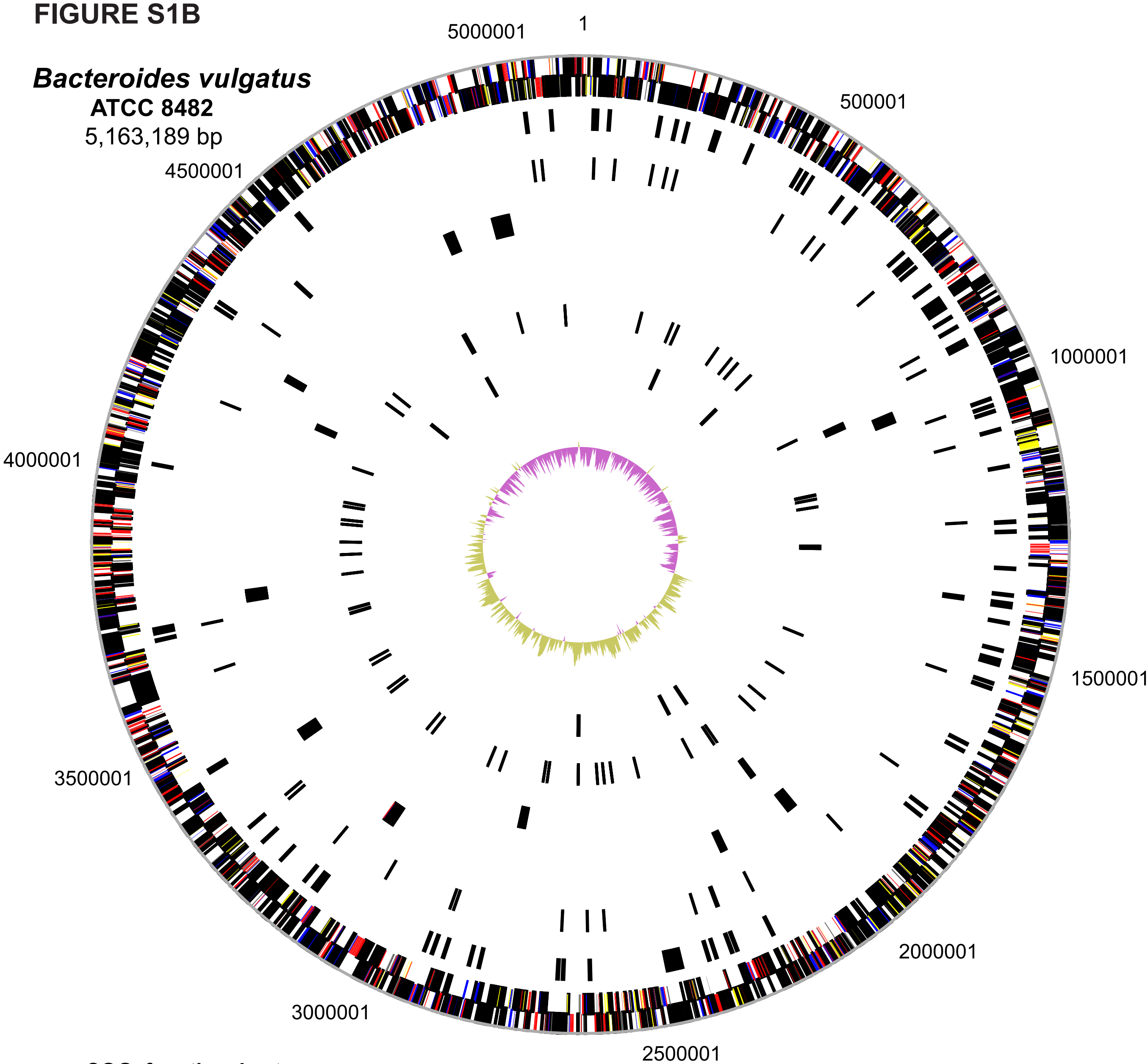

COG functional category

M	Cell wall/membrane/envelope biogenesis	I	Lipid transport and metabolism
G	Carbohydrate transport and metabolism	J	Translation, ribosomal structure and biogenesis
L	Defense mechanisms	K	Transcription
V	Replication, recombination and repair	O	Posttranslational modification, protein turnover, chaperones
C	Energy production and conversion	P	Inorganic ion transport and metabolism
D	Cell division, chromosome partitioning	Q	Secondary metabolites biosynthesis, transport and catabolism
E	Amino acid transport and metabolism	T	Signal transduction mechanisms
F	Nucleotide transport and metabolism	U	Intracellular trafficking, secretion, and vesicular transport
H	Coenzyme transport and metabolism		
