# Supplementary material for: Evolution of Symbiotic Bacteria in the Distal Human Intestine
Source: PLoS Biol. 2007 Jun 19;5(7):e156. doi: 10.1371/journal.pbio.0050156 (PMC1892571; doi:10.1371/journal.pbio.0050156)
Supplement: Supplementary file 2 [file pbio.0050156.sg002.pdf]

FIGURE S2

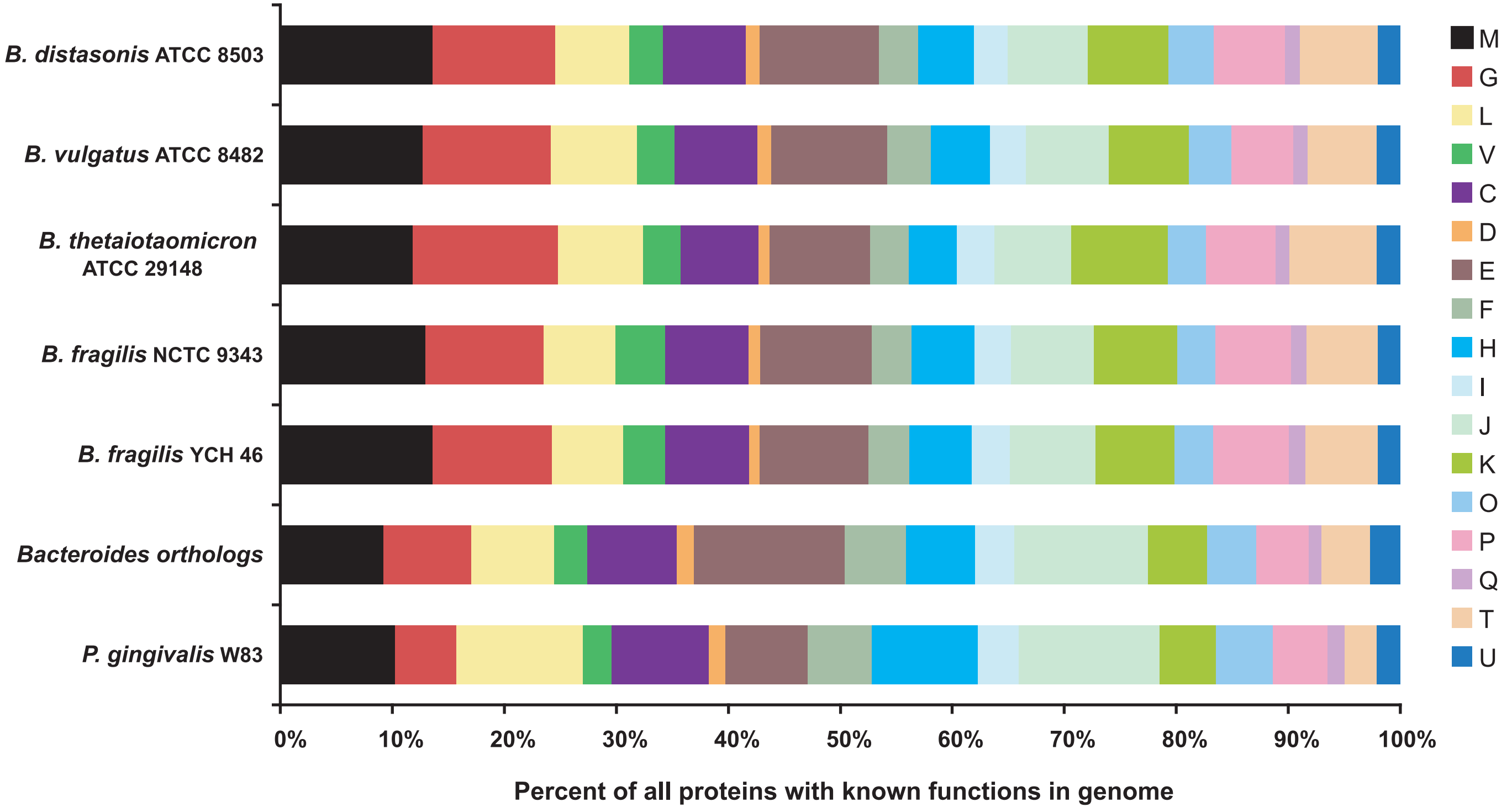

B. distasonis ATCC 8503
B. vulgatus ATCC 8482
B. thetaiotaomicron ATCC 29148
B. fragilis NCTC 9343
B. fragilis YCH 46
Bacteroides orthologs
P. gingivalis W83
0%
10%
20%
30%
40%
50%
60%
70%
80%
90%
100%
Percent of all proteins with known functions in genome
M
G
L
V
C
D
E
F
H
I
J
K
O
P
Q
T
U
