# Supplementary material for: Evolution of Symbiotic Bacteria in the Distal Human Intestine
Source: PLoS Biol. 2007 Jun 19;5(7):e156. doi: 10.1371/journal.pbio.0050156 (PMC1892571; doi:10.1371/journal.pbio.0050156)
Supplement: Supplementary file 3 [file pbio.0050156.sg003.pdf]

FIGURE S3

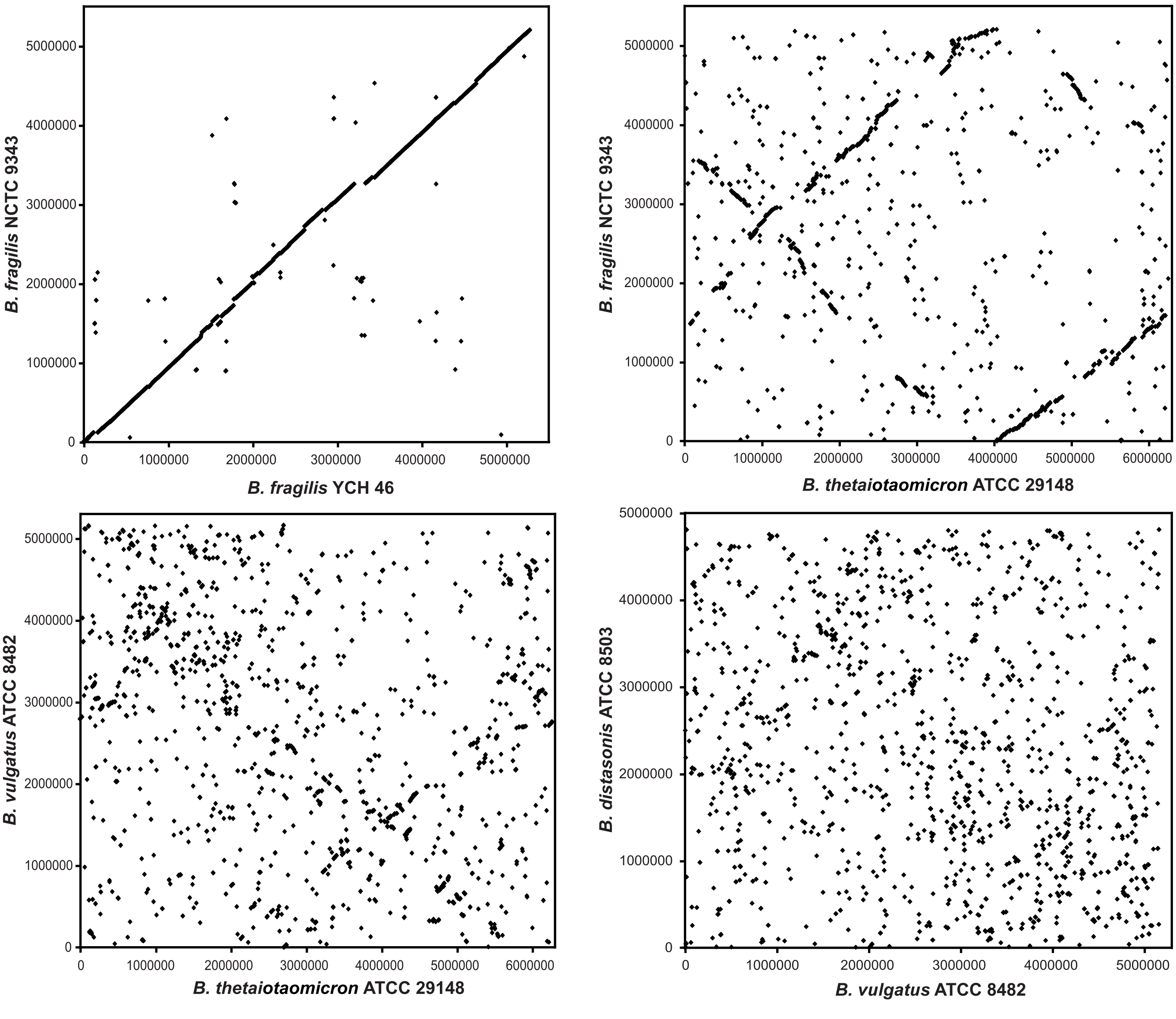

5000000
4000000
3000000
2000000
1000000
0
B. fragilis NCTC 9343
0
1000000
2000000
3000000
4000000
5000000
B. fragilis YCH 46
B. fragilis NCTC 9343
6000000
B. thetaiotaomicron ATCC 29148
B. vulgatus ATCC 8482
B. thetaiotaomicron ATCC 29148
B. distasonis ATCC 8503
B. vulgatus ATCC 8482
