# Supplementary material for: Evolution of Symbiotic Bacteria in the Distal Human Intestine
Source: PLoS Biol. 2007 Jun 19;5(7):e156. doi: 10.1371/journal.pbio.0050156 (PMC1892571; doi:10.1371/journal.pbio.0050156)
Supplement: Supplementary file 4 [file pbio.0050156.sg004.pdf]

FIGURE S4

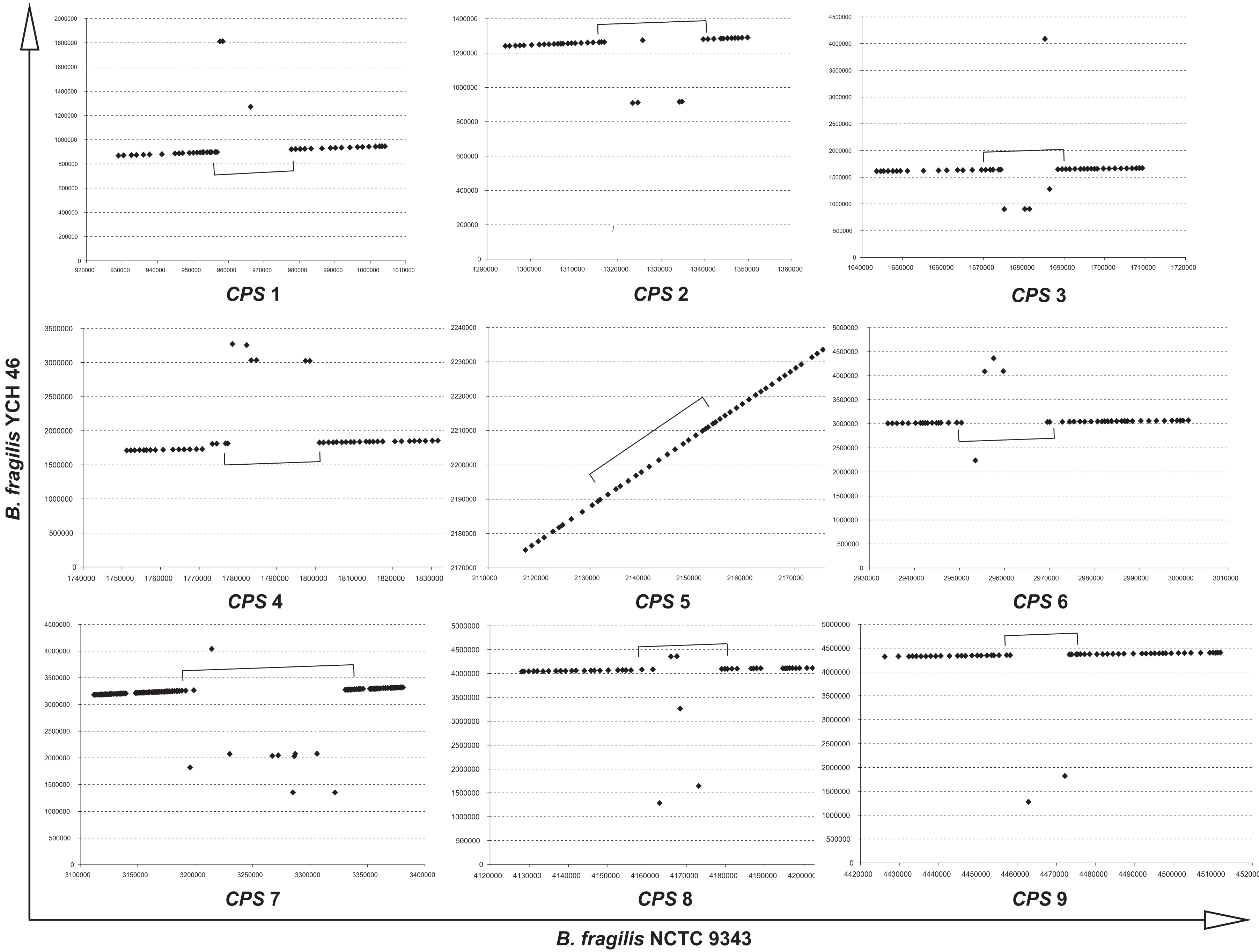
CPS 1
CPS 2
CPS 3
CPS 4
CPS 5
CPS 6
CPS 7
CPS 8
CPS 9
B. fragilis YCH 46
B. fragilis NCTC 9343
