# Supplementary material for: Evolution of Symbiotic Bacteria in the Distal Human Intestine
Source: PLoS Biol. 2007 Jun 19;5(7):e156. doi: 10.1371/journal.pbio.0050156 (PMC1892571; doi:10.1371/journal.pbio.0050156)
Supplement: Supplementary file 5 [file pbio.0050156.st001.pdf]

Table S1. Comparison of genome parameters for *B. distasonis* ATCC 8503, *B. vulgatus* ATCC 8482, *B. thetaiotaomicron* ATCC 29148, *B. fragilis* NCTC 9343 and *B. fragilis* YCH 46.

**Feature**	***B. distasonis* ATCC 8503**	***B. vulgatus* ATCC 8482**	***B. thetaiotaomicron* ATCC 29148**	***B. fragilis* NCTC 9343**	***B. fragilis* YCH 46**
Genome size (bp)	**4,811,379**	**5,163,189**	6,260,361	5,205,140	5,277,274
G + C (%)	**45.1**	**42.2**	42.8	43.2	43.3
Protein coding (%)	**90.3**	**89.1**	89.5	89.3	90.1
Gene density (no. of CDS per Kb)	**0.804**	**0.792**	0.763	0.818	0.867
Average CDS length (bp)	**1,123**	**1,126**	1,173	1,091	1,039
Protein-coding genes (no.)	**3,867**	**4,088**	4,778	4,189	4,578
CDS with functional assignment	**2,427**	**2,505**	2,709	2,753	2,959
CDS without functional asignment	**1,440**	**1,583**	2,069	1,436	1,619
conserved hypothetical protein	**1,005**	**1,123**	1,532	858	950
hypothetical protein	**435**	**460**	537	578	669
Ribosomal RNA loci	**7**	**7**	5	6	6
Transfer RNAs	**83**	**85**	71	73	74
Conjugative transposons	**1**	**4**	4	2	2
Transposases	**61**	**121**	109	47	60
Phages	**5**	**3**	5	2	4
Glycoside hydrolases (GH)	**97**	**159**	226	126	132
Polysaccharide lyases (PL)	**0**	**7**	15	1	1
Glycosyltransferases (GT)	**78**	**76**	85	79	81
SusC homologs*	**63 (60)**	**89 (86)**	107 (107)	88 (88)	77 (77)
SusD homologs*	**48 (54)**	**74 (80)**	57 (102)	48 (71)	47 (65)
ECF-σ factors	**36**	**41**	50	42	41
Anti-σ factors	**23**	**21**	25	25	25
Hybrid two-component systems	**7**	**22**	32	14	12

Legend: '*', the numbers of SusC/SusD homologs provided are based on BLASTP e-value ≤10^{-20}; the numbers shown in parentheses are based on criteria described in SusC/SusD alignments in **Materials and Methods**. See http://gordonlab.wustl.edu/BvBd.html for complete lists of SusC/SusD homologs. A hybrid two-component system protein contains all of the domains present in classical two-component systems but in one polypeptide (Xu, *et al,* Science 299:2074-6, 2003).
