# Supplementary material for: Evolution of Symbiotic Bacteria in the Distal Human Intestine
Source: PLoS Biol. 2007 Jun 19;5(7):e156. doi: 10.1371/journal.pbio.0050156 (PMC1892571; doi:10.1371/journal.pbio.0050156)
Supplement: Supplementary file 6 [file pbio.0050156.st002.pdf]

Table S2. Shared orthologs in *B. distasonis* ATCC 8503, *B. vulgatus* ATCC 8482, *B. thetaiotaomicron* ATCC 29148, and *B. fragilis* strains NCTC 9343 and YCH 46.

B. distasonis **ATCC 8503**	***B. vulgatus*** **ATCC 8482**	***B. thetaiotaomicron*** **ATCC 29148**	***B. fragilis*** **NCTC 9343**	***B. fragilis*** **YCH 46**	**Annotation**	**COG functional code**
Bd0001	Bv0001	29347553	60683067	53715109	chromosomal replication initiator protein DnaA	L
Bd0002	Bv0003	29347555	60683069	53715111	putative ribonucleoside reductase	F
Bd0003	Bv1354c	29347556	60683070	53715112	glycoside hydrolase family 77, candidate 4-alpha-	G
Bd0005	Bv0005	29347559	60683072	53715114	dihydroneopterin aldolase	H
Bd0006	Bv0338	29347244	60683111	53715176	putative hydrogenase	C
Bd0008	Bv0220c	29347680	60680154	53711954	MarC family integral membrane protein	U
Bd0016	Bv1685	29346749	60682925	53714950	undecaprenyl-phosphate alpha-N-acetylglucosaminyltransferase	M
Bd0046c	Bv0007c	29347561	60683075	53715138	glycosyltransferase family 2, related to beta-glycosyltransferases	R
Bd0047c	Bv1031	29347562	60683076	53715139	putative acetyltransferase	n/a
Bd0048c	Bv0008c	29347563	60683077	53715140	putative Fe-S oxidoreductase	J
Bd0051	Bv0009	29347564	60683078	53715141	putative long-chain-fatty-acid-CoA ligase	I
Bd0059c	Bv0564c	29348437	60680326	53712140	conserved hypothetical protein	n/a
Bd0067c	Bv0572c	29349166	60680052	53711822	acetylornithine aminotransferase	E
Bd0068c	Bv0573c	29349167	60680053	53711823	N-acetyl-gamma-glutamyl-phosphate reductase	E
Bd0069c	Bv0574c	29349168	60680054	53711824	argininosuccinate synthase	E
Bd0070c	Bv0575c	29349169	60680055	53711825	conserved hypothetical protein	n/a
Bd0071c	Bv0576c	29349170	60680056	53711826	transcriptional regulator	K
Bd0073c	Bv0756	29348106	60683407	53715435	DNA mismatch repair protein MutS	L
Bd0079	Bv2526c	29349026	60679956	53711718	conserved hypothetical protein	n/a
Bd0080	Bv2525c	29345543	60679955	53711717	putative glucokinase/SIS domain protein	R
Bd0081	Bv3168	29349892	60682837	53714854	conserved hypothetical protein	S
Bd0082	Bv2030c	29349883	60682844	53714862	conserved hypothetical protein	n/a
Bd0083	Bv2029c	29349877	60682848	53714867	conserved hypothetical protein	L
Bd0088c	Bv0570c	29349462	60680280	53712087	putative transcriptional regulator	K
Bd0089c	Bv0716c	29348540	60679827	53711578	glycoside hydrolase family 36, candidate alpha-glycosidase; related to alpha-galactosidases	n/a
Bd0091	Bv2554c	29345597	60682502	53714479	conserved hypothetical protein	R
Bd0092c	Bv1897c	29347460	60682984	53715021	conserved hypothetical protein	n/a
Bd0097	Bv3189	29347160	60682640	53714621	glycine betaine/L-proline transport system permease	E
Bd0103	Bv0188c	29346686	60682087	53713879	conserved hypothetical protein	n/a
Bd0114	Bv3187c	29347843	60680096	53711868	two-component system response regulator	T
Bd0115	Bv3169c	29347830	60680102	53711874	two-component system sensor histidine kinase, FrrD	T
Bd0133	Bv0249	29349320	60683200	53715263	conserved hypothetical protein	n/a

Bd0136	Bv0252	29349312	60683205	53715269	conserved hypothetical protein	M
Bd0137	Bv0253	29349311	60683206	53715270	conserved hypothetical protein	V
Bd0138	Bv0254	29349310	60683207	53715271	putative transport-related membrane protein	V
Bd0141c	Bv4005	29347537	60683056	53715098	putative beta-phosphoglucomutase	R
Bd0143c	Bv4004	29347534	60683054	53715096	glucose-6-phosphate isomerase	G
Bd0144c	Bv4003	29347533	60683053	53715095	glycerol-3-phosphate dehydrogenase	C
Bd0145c	Bv4002	29347532	60683052	53715094	lysyl-tRNA synthetase	J
Bd0150c	Bv1602c	29347421	60682959	53714993	putative nicotinate-nucleotide adenylyltransferase	H
Bd0151c	Bv1603c	29347419	60682958	53714992	guanylate kinase	F
Bd0152c	Bv1604c	29347418	60682957	53714991	conserved hypothetical protein	S
Bd0153	Bv1605	29347417	60682956	53714989	putative glycoprotease	O
Bd0155c	Bv2427c	29347099	60682600	53714578	oxaloacetate decarboxylase beta chain	C
Bd0156c	Bv2428c	29347098	60682599	53714577	biotin carboxyl carrier protein	I
Bd0157c	Bv2429c	29347097	60682598	53714576	conserved hypothetical protein	n/a
Bd0158c	Bv2430c	29347096	60682597	53714575	propionyl-CoA carboxylase beta chain	I
Bd0159c	Bv2431c	29347095	60682596	53714574	lactoyl gluthatione lyase	E
Bd0161	Bv2433	29347090	60682593	53714571	putative NADH dehydrogenase/NAD(P)H nitroreductase	C
Bd0162	Bv2435	29347088	60682591	53714569	conserved hypothetical protein	n/a
Bd0163	Bv2436	29347087	60682590	53714568	putative hydrolase	R
Bd0164	Bv2437	29347086	60682589	53714567	putative septum formation-related protein	D
Bd0166c	Bv2567c	29349968	60680674	53712474	thiamine biosynthesis lipoprotein apbE precursor	H
Bd0168c	Bv2568c	29349969	60680675	53712475	conserved hypothetical protein	S
Bd0170c	Bv2571c	29349972	60680678	53712478	glycosyltransferase family 2, related to beta-glycosyltransferases	M
Bd0173	Bv2504c	29349774	60680532	53712364	putative regulatory protein	K
Bd0174	Bv2503c	29349775	60680533	53712365	putative outer membrane protein	n/a
Bd0175	Bv2502c	29349776	60680534	53712366	conserved hypothetical protein	n/a
Bd0176	Bv2501c	29349777	60680536	53712367	conserved hypothetical protein	n/a
Bd0177	Bv2500c	29349778	60680537	53712368	putative transmembrane CBS domain transporter	R
Bd0178	Bv2499c	29349779	60680538	53712369	peptidyl-prolyl cis-trans isomerase	n/a
Bd0179	Bv2537	29349780	60680539	53712370	conserved hypothetical protein	R
Bd0180	Bv2538	29349781	60680540	53712371	conserved hypothetical protein	n/a
Bd0181	Bv2539	29349782	60680541	53712372	4-hydroxythreonine-4-phosphate dehydrogenase	H
Bd0186	Bv2548	29349783	60680542	53712373	transcriptional regulator	T
Bd0187	Bv2549	29349784	60680543	53712374	conserved hypothetical protein	n/a
Bd0188	Bv2550	29349785	60680544	53712375	conserved hypothetical protein	n/a
Bd0190	Bv1295c	29348388	60683723	53715796	putative membrane protein	n/a
Bd0192c	Bv2555c	29349661	60680426	53712248	putative 6,7-dimethyl-8-ribityllumazine synthase	H
Bd0193c	Bv2556c	29349662	60680427	53712249	conserved hypothetical protein	n/a
Bd0194	Bv2557	29349663	60680428	53712250	DNA replication and repair protein RecF	L

Bd0195	Bv2558	29349664	60680429	53712251	conserved hypothetical protein	n/a
Bd0196c	Bv2560c	29349666	60680431	53712254	putative 5-formyltetrahydrofolate cyclo-ligase	H
Bd0197c	Bv2561c	29349667	60680432	53712255	carboxy-terminal processing protease precursor	M
Bd0198c	Bv2562c	29349668	60680433	53712256	deoxycytidylate deaminase	F
Bd0199c	Bv2564c	29349670	60680435	53712258	putative peptidyl-dipeptidase	E
Bd0200	Bv2708	29349711	60680468	53712290	conserved hypothetical protein	G
Bd0201	Bv2709	29349712	60680469	53712291	glycosyltransferase family 4, related to alpha-glycosyltransferases	M
Bd0202	Bv2710	29349713	60680470	53712292	glycoside hydrolase family 57, candidate alpha-glycosidase; related to alpha-amylases	G
Bd0203c	Bv2823c	29349714	60680471	53712293	conserved hypothetical protein	n/a
Bd0204c	Bv2824c	29349715	60680472	53712294	glycosyltransferase family 5, related to bacterial glycogen synthase; possible fragment	G
Bd0205	Bv2825	29349716	60680473	53712295	pantoate--beta-alanine ligase	H
Bd0206	Bv2826	29349717	60680474	53712296	aspartate 1-decarboxylase precursor	H
Bd0208	Bv2422c	29347921	60680802	53712589	putative transcriptional regulator	n/a
Bd0209	Bv2423c	29347922	60680801	53712588	cation-transporting ATPase, P-type, putative zinc-transporting ATPase	P
Bd0210	Bv2157	29347011	60682525	53714504	putative signal recognition protein	U
Bd0211	Bv2158	29347017	60682527	53714506	methylenetetrahydrofolate dehydrogenase/methenyltetrahydrofolate cyclohydrolase	H
Bd0218	Bv3951c	29345836	60681192	53712980	conserved hypothetical protein	n/a
Bd0220c	Bv0966	29349649	60680402	53712222	glycoside hydrolase family 2, candidate beta-galactosidase	G
Bd0221c	Bv1636c	29346225	60681855	53713572	conserved hypothetical protein	n/a
Bd0224	Bv2003	29348521	60680351	53712168	hydrolase of the alpha/beta superfamily	R
Bd0225	Bv1977	29349217	60683356	53715387	ABC transporter ATP-binding protein	R
Bd0226	Bv4028c	29346085	60682422	53714402	N-acetylglucosamine-6-phosphate deacetylase	G
Bd0227	Bv0057c	29346086	60682224	53714027	N-acetylglucosamine-6-phosphate deacetylase	G
Bd0228	Bv4016	29345668	60682423	53714403	putative glucosamine-6-phosphate deaminase	G
Bd0229	Bv0609c	29349401	60680244	53712049	RNA polymerase ECF-type sigma factor	K
Bd0235	Bv4086c	29347550	60683064	53715106	putative sodium-dependent transporter	n/a
Bd0246c	Bv3139	29346821	60683535	53715584	methylated-DNA-protein-cysteine methyltransferase	L
Bd0249c	Bv1997	29347811	60680128	53711901	putative threonine synthase	E
Bd0250c	Bv1996	29347812	60680127	53711900	phosphoglycerate mutase	G
Bd0251c	Bv1995	29347813	60680126	53711899	putative aspartokinase I-homoserine dehydrogenase	EE
Bd0252c	Bv1917c	29349459	60680278	53712083	conserved hypothetical protein	GER
Bd0253c	Bv2927	29346129	60681714	53713468	ATP synthase gamma chain	C
Bd0254c	Bv2926	29346128	60681713	53713467	ATP synthase alpha chain	C
Bd0255c	Bv2925	29346127	60681712	53713466	ATP synthase delta chain	n/a
Bd0256c	Bv2924	29346126	60681711	53713465	ATP synthase B subunit	C

Bd0258c	Bv2922	29346124	60681709	53713463	ATP synthase A subunit	C
Bd0259c	Bv2921	29346123	60681708	53713462	conserved hypothetical protein	n/a
Bd0260c	Bv2920	29346122	60681707	53713461	ATP synthase epsilon chain	C
Bd0261c	Bv2919	29346121	60681706	53713460	ATP synthase beta chain	C
Bd0262	Bv3793	29349824	60682866	53714886	putative copper homeostasis protein	P
Bd0272	Bv2965c	29345980	60682121	53713912	UvrABC SOS-repair system protein B	L
Bd0273c	Bv3482c	29349749	60680504	53712333	conserved hypothetical protein	n/a
Bd0275	Bv3484	29349746	60680502	53712329	transcriptional regulator	T
Bd0276c	Bv3486c	29349729	60680485	53712311	2-dehydro-3-deoxyphosphooctonate aldolase	M
Bd0277c	Bv3488c	29349731	60680487	53712313	tRNA delta(2)-isopentenylpyrophosphate transferase	J
Bd0278c	Bv3489c	29349671	60680436	53712259	glyceraldehyde 3-phosphate dehydrogenase	G
Bd0280c	Bv2230c	29345724	60681132	53712914	ribosomal protein L11 methyltransferase	J
Bd0299	Bv3538	29349722	60680479	53712302	50S ribosomal protein L21	J
Bd0300	Bv3539	29349721	60680478	53712301	50S ribosomal protein L27	J
Bd0301	Bv3734c	29349990	60680806	53712593	amidophosphoribosyltransferase precursor	F
Bd0302	Bv3733c	29349991	60680807	53712594	peptidase T (tripeptide aminopeptidase)	E
Bd0303	Bv3732c	29349992	60680808	53712595	aminomethyltransferase	E
Bd0309c	Bv2306c	29345615	60682492	53714469	glutamine-dependent NAD+ synthetase	RH
Bd0312c	Bv3901c	29345957	60682142	53713935	aspartate aminotransferase	E
Bd0313c	Bv3902c	29345958	60682141	53713934	diaminopimelate epimerase	E
Bd0315c	Bv3905	29345961	60682137	53713931	asparagine synthetase B	E
Bd0319	Bv1655	29349463	60680335	53712150	TonB-dependent receptor	n/a
Bd0322c	Bv3729	29349995	60680687	53712487	conserved hypothetical protein	J
Bd0323c	Bv3728	29349996	60680688	53712488	peptidyl-tRNA hydrolase	J
Bd0324c	Bv3727	29349997	60680689	53712489	putative 50S ribosomal protein L25	J
Bd0328c	Bv0230c	29348096	60683398	53715425	putative membrane fusion protein transporter	M
Bd0329c	Bv0231c	29348097	60683399	53715426	putative outer membrane efflux protein	MU
Bd0330c	Bv0232c	29348098	60683400	53715427	putative transcriptional regulator	K
Bd0332c	Bv3980c	29345952	60682143	53713936	conserved hypothetical protein	n/a
Bd0334	Bv1190c	29347241	60680019	53711787	conserved hypothetical protein	n/a
Bd0335	Bv1189c	29347242	60680018	53711786	conserved hypothetical protein	n/a
Bd0337	Bv3440c	29346877	60681104	53712886	ABC transporter permease	M
Bd0340c	Bv3889	29347496	60683039	53715078	choloylglycine hydrolase	M
Bd0342	Bv2655	29346727	60682239	53714044	riboflavin synthase alpha chain	H
Bd0344	Bv2657c	29347221	60682675	53714662	conserved hypothetical protein	n/a
Bd0346c	Bv2656c	29346725	60682237	53714041	ribonuclease BN	S
Bd0348	Bv2671	29346722	60682233	53714036	serine protease precursor	O
Bd0349	Bv2672	29346721	60682231	53714034	RNA polymerase sigma factor rpoD (Sigma-A)	K
Bd0355c	Bv1645	29349799	60680578	53712416	putative metalloendopeptidase	M

Bd0360c	Bv1230	29346842	60679738	53711468	mannonate dehydratase	G
Bd0361c	Bv1229	29346843	60679739	53711469	putative oxidoreductase	IQR
Bd0364	Bv0236c	29348748	60679752	53711482	AcrA/AcrE family multidrug resistance protein	M
Bd0366	Bv0235c	29348747	60679751	53711481	AcrB/AcrD family multidrug resistance protein	V
Bd0367	Bv0234c	29348746	60679750	53711480	outer membrane protein oprM precursor	MU
Bd0373c	Bv0248c	29349319	60683199	53715262	malate dehydrogenase	C
Bd0376c	Bv3527c	29349739	60680495	53712322	conserved hypothetical protein	R
Bd0377c	Bv3528c	29349740	60680496	53712323	conserved hypothetical protein	n/a
Bd0378c	Bv3529c	29349741	60680497	53712324	conserved hypothetical protein	n/a
Bd0379	Bv0559c	29346373	60683528	53715577	putative ferredoxin-type protein	C
Bd0381	Bv0560c	29346372	60683529	53715578	aldo/keto reductase	R
Bd0383	Bv3530	29349742	60680498	53712325	FtsK/SpoIIIE family cell division protein	D
Bd0384	Bv3531	29349743	60680499	53712326	conserved hypothetical protein	n/a
Bd0385	Bv3532	29349744	60680500	53712327	thioredoxin reductase	O
Bd0387	Bv0089	29347154	60682632	53714611	conserved hypothetical protein	S
Bd0391	Bv0052c	29350128	60680838	53712627	putative RNA polymerase ECF-type sigma factor	K
Bd0396	Bv2465	29345588	60682511	53714488	conserved hypothetical protein	n/a
Bd0397	Bv2466	29345589	60682510	53714487	SsrA-binding protein	O
Bd0399	Bv2467	29345590	60682509	53714486	5-methyltetrahydrofolate-homocysteine methyltransferase	EE
Bd0430	Bv0495c	29349759	60680512	53712341	conserved hypothetical protein	n/a
Bd0432	Bv2984	29346216	60681847	53713564	isoleucyl-tRNA synthetase	J
Bd0433	Bv2985	29346217	60681848	53713565	DnaK suppressor protein, putative	T
Bd0434	Bv2986	29346218	60681849	53713566	putative signal peptidase	MU
Bd0435	Bv2987	29346219	60681850	53713567	conserved hypothetical protein	n/a
Bd0436c	Bv2989c	29346221	60681852	53713569	putative tRNA/rRNA methyltransferase	J
Bd0437	Bv2990	29346224	60681854	53713571	putative surface membrane protein	n/a
Bd0438	Bv2310c	29345629	60682485	53714462	thioredoxin	OC
Bd0440c	Bv3167	29346047	60682056	53713843	conserved hypothetical protein	S
Bd0441	Bv3840	29346739	60682249	53714055	putative thiol peroxidase	O
Bd0443	Bv1827c	29347651	60680190	53711987	DNA-damage-inducible protein F	V
Bd0444c	Bv0320	29347216	60682673	53714660	acyl-CoA dehydrogenase	I
Bd0445c	Bv0318	29347215	60682672	53714659	electron transfer flavoprotein alpha-subunit	C
Bd0446c	Bv0317	29347214	60682671	53714658	electron transfer flavoprotein beta-subunit	C
Bd0450c	Bv2945c	29346557	60681563	53713314	glycine dehydrogenase	EE
Bd0451c	Bv2946c	29346556	60681564	53713315	putative metallo-beta-lactamase	R
Bd0452c	Bv2947c	29346555	60681565	53713316	glucose-inhibited division protein	M
Bd0453c	Bv2948c	29346554	60681566	53713317	conserved hypothetical protein	n/a
Bd0459	Bv1606	29347416	60682955	53714988	conserved hypothetical protein	n/a
Bd0460	Bv1607	29347415	60682954	53714987	UDP-N-acetylglucosamine 1-carboxyvinyltransferase	M

Bd0461	Bv1608	29347414	60682953	53714986	RimM protein, required for 16S rRNA processing	n/a
Bd0462	Bv1609	29347413	60682952	53714985	putative membrane peptidase	M
Bd0463	Bv1610	29347412	60682951	53714984	putative terpenoid biosynthesis related reductoisomerase	I
Bd0464	Bv1611	29347411	60682950	53714983	membrane-associated zinc metalloprotease	M
Bd0466c	Bv3711	29346964	60680918	53712768	alanine dehydrogenase	E
Bd0467c	Bv2507	29349770	60680529	53712360	putative preprotein translocase SecA component	U
Bd0468c	Bv2506	29349771	60680530	53712361	putative alkaline phosphatase	n/a
Bd0469c	Bv2505	29349772	60680531	53712362	conserved hypothetical protein	n/a
Bd0470	Bv1305	29348804	60679792	53711531	putative acetylglutamate kinase	E
Bd0474	Bv2721	29347072	60682562	53714540	putative molybdenum transport ATP-binding protein	P
Bd0480	Bv1795	29347677	60680159	53711957	tyrosine type site-specific recombinase	L
Bd0486	Bv3807c	29346615	60681434	53713173	putative ATPase, AAA family	L
Bd0487	Bv3159	29346563	60681556	53713307	phosphoserine aminotransferase	HE
Bd0488	Bv3160	29346562	60681557	53713308	D-3-phosphoglycerate dehydrogenase	HE
Bd0489	Bv3161	29346561	60681558	53713309	conserved hypothetical protein	S
Bd0492c	Bv3779c	29346033	60682092	53713884	putative UDP-glucose 4-epimerase	M
Bd0493c	Bv3781c	29346032	60682093	53713885	Na+-transporting NADH:ubiquinone oxidoreductase, electron transport complex protein rnfA	C
Bd0494c	Bv3782c	29346031	60682094	53713886	Na+-transporting NADH:ubiquinone oxidoreductase, electron transport complex protein rnfE	C
Bd0495c	Bv3783c	29346030	60682095	53713887	electron transport complex protein RnfG	C
Bd0496c	Bv3784c	29346029	60682096	53713888	Na+-transporting NADH:ubiquinone oxidoreductase, electron transport complex protein rnfD	C
Bd0497c	Bv3785c	29346028	60682097	53713889	Na+-transporting NADH:ubiquinone oxidoreductase, electron transport complex protein rnfC	C
Bd0498c	Bv3786c	29346027	60682098	53713890	electron transport complex protein RnfB	C
Bd0499c	Bv3787c	29346026	60682099	53713891	conserved hypothetical protein	n/a
Bd0500c	Bv3267c	29346001	60682101	53713893	conserved hypothetical protein	n/a
Bd0501	Bv3268	29346000	60682103	53713894	CTP synthase (UTP-ammonia ligase)	F
Bd0502	Bv3269	29345999	60682104	53713895	conserved hypothetical protein	U
Bd0506	Bv2032c	29349885	60682842	53714860	conserved hypothetical protein	n/a
Bd0508	Bv1453	29346607	60681443	53713181	RNA polymerase ECF-type sigma factor	K
Bd0509c	Bv3766	29346636	60681376	53713110	putative long-chain-fatty-acid--CoA ligase	I
Bd0510	Bv3259	29346605	60681447	53713186	glycosyltransferase family 51, candidate monofunctional peptidoglycan glycosyltransferase	M
Bd0512	Bv1217	29348735	60679746	53711476	putative nifR3 family TIM-barrel enzyme	J
Bd0513c	Bv3143c	29346331	60681993	53713723	conserved hypothetical protein	n/a
Bd0514	Bv3195	29346330	60681992	53713722	putative O-sialoglycoprotein endopeptidase	O
Bd0520	Bv3536	29349725	60680481	53712307	putative lipoprotein protein	M

Bd0521	Bv3542	29349720	60680477	53712299	seryl-tRNA synthetase	J
Bd0523c	Bv2363	29347844	60680094	53711866	pyridine nucleotide-disulphide oxidoreductase	POR
Bd0525c	Bv3765	29346743	60682297	53714240	putative dihydroorotate dehydrogenase	F
Bd0526c	Bv3764	29346742	60682296	53714239	conserved hypothetical protein	R
Bd0527c	Bv3763	29346741	60682295	53714238	conserved hypothetical protein	n/a
Bd0560	Bv3895c	29345965	60682136	53713929	putative phosphoribosylpyrophosphate amidotransferase	F
Bd0561	Bv3894c	29345966	60682135	53713928	glutamine-hydrolyzing carbamoyl phosphate synthetase III	EF
Bd0562	Bv3893c	29345967	60682134	53713927	carbamyl phosphate synthetase	EF
Bd0563	Bv3769c	29346644	60681369	53713102	conserved hypothetical protein	n/a
Bd0564	Bv2806	29349863	60682860	53714879	oxidoreductase, putative glycolate oxidase	C
Bd0565	Bv2807	29349864	60682859	53714878	putative electron transport protein	C
Bd0566	Bv2808	29349865	60682858	53714877	conserved hypothetical protein	S
Bd0567c	Bv0198	29345945	60682146	53713939	putative alcohol dehydrogenase	C
Bd0568c	Bv3988	29345937	60682153	53713948	tryptophan synthase alpha chain	E
Bd0569c	Bv3987	29345938	60682152	53713947	putative N-(5'-phosphoribosyl) anthranilate isomerase	E
Bd0570c	Bv3986	29345939	60682151	53713946	indole-3-glycerol phosphate synthase	E
Bd0571c	Bv3985	29345940	60682150	53713945	anthranilate phosphoribosyltransferase	E
Bd0572c	Bv3984	29345941	60682149	53713944	anthranilate synthase component II	EH
Bd0573c	Bv3983	29345942	60682148	53713943	anthranilate synthase component I	EH
Bd0574c	Bv3982	29345943	60682147	53713942	tryptophan synthase beta chain	E
Bd0575	Bv3776c	29348164	60683471	53715498	aspartate ammonia-lyase	E
Bd0578c	Bv1848c	29347070	60682560	53714538	phosphoglycerate mutase	G
Bd0579c	Bv1427	29350106	60680805	53712592	conserved hypothetical protein	S
Bd0584	Bv2147c	29347970	60683553	53715609	putative outer membrane protein, probably involved in nutrient binding	n/a
Bd0589	Bv2663c	29349882	60682845	53714863	conserved hypothetical protein	E
Bd0590	Bv3165	29346287	60681947	53713674	ferredoxin domain containing protein	n/a
Bd0592c	Bv2977c	29346680	60682201	53714005	putative Na+/H+ antiporter	C
Bd0598c	Bv2211	29345662	60682433	53714414	transcription-repair coupling factor	LK
Bd0615	Bv0595c	29346444	60680833	53712622	putative signal transducer	GEPR
Bd0616	Bv0594c	29346443	60680834	53712623	conserved hypothetical protein	G
Bd0618	Bv3969c	29347223	60682678	53714665	putative sulfatase	R
Bd0619	Bv0371	29349213	60683362	53715394	two-component system sensor histidine kinase, with response regulator receiver domain	T
Bd0621	Bv0372	29349209	60683366	53715398	two-component system response regulator	T
Bd0624c	Bv0501c	29349096	60679995	53711762	putative acetyltransferase	n/a
Bd0625c	Bv0502c	29349097	60679996	53711763	conserved hypothetical protein	n/a
Bd0628	Bv2729	29346632	60681408	53713143	6-phosphogluconate dehydrogenase,decarboxylating	G
Bd0629	Bv2730	29346631	60681409	53713144	glucose-6-phosphate 1-dehydrogenase	G

Bd0630	Bv2731	29346630	60681410	53713145	6-phosphogluconolactonase	G
Bd0633	Bv3841c	29346635	60681377	53713111	GDP-4-keto-6-deoxy-D-mannose-3,5-epimerase-4-reductase	MG
Bd0637	Bv3957c	29345829	60681184	53712972	putative endonuclease	L
Bd0638	Bv3956c	29345830	60681185	53712973	peptide deformylase(PDF)	J
Bd0641	Bv3955c	29345831	60681187	53712975	putative exported Tpr repeat-family protein	R
Bd0642	Bv3954c	29345832	60681188	53712976	threonyl-tRNA synthetase	J
Bd0643	Bv3953c	29345833	60681189	53712977	translation initiation factor IF-3	J
Bd0645	Bv3952c	29345835	60681191	53712979	50S ribosomal protein L20	J
Bd0647	Bv4031c	29345870	60681305	53713025	glycoside hydrolase family 20, candidate beta-N-acetylhexosaminidase	G
Bd0648	Bv4030c	29346442	60681306	53713026	glycoside hydrolase family 92, related to an ill-defined alpha-1,2-mannosidase	G
Bd0649c	Bv0821	29348823	60679833	53711582	Mg2+/Co2+ transporter	P
Bd0651	Bv3382	29346035	60682088	53713880	DNA helicase	L
Bd0654c	Bv2149	29347082	60682576	53714555	phosphoglycerate kinase	G
Bd0658c	Bv3219	29346306	60681970	53713699	putative patatin-like phospholipase	R
Bd0660c	Bv3218	29346307	60681971	53713700	chaperone protein	O
Bd0670c	Bv1569	29348801	60679786	53711523	nitroreductase	R
Bd0671	Bv3197	29346326	60681990	53713720	50S ribosomal protein L28	J
Bd0672	Bv3198	29346325	60681989	53713719	50S ribosomal protein L33	J
Bd0673	Bv2821c	29346774	60682327	53714271	DNA polymerase III, beta chain	L
Bd0676	Bv2819c	29346772	60682325	53714269	flavoprotein	H
Bd0678	Bv2818c	29346771	60682324	53714268	DNA repair protein recN (Recombination protein N)	L
Bd0682c	Bv3504	29346676	60683725	53715798	putative two-component system sensor histidine kinase	T
Bd0683c	Bv3503	29346677	60683726	53715799	outer membrane protein	MU
Bd0684c	Bv3501	29346678	60683727	53715800	putative transmembrane AcrB/D/F-family transporter	V
Bd0685c	Bv3500	29346679	60683728	53715801	component of multidrug efflux system	M
Bd0686	Bv1789c	29347685	60680149	53711949	thymidine kinase	F
Bd0687	Bv2817c	29346770	60682323	53714267	tRNA/rRNA methyltransferase	J
Bd0688	Bv2861	29346746	60682321	53714265	endoribonuclease L-PSP, putative	J
Bd0689c	Bv2221c	29345664	60682432	53714413	putative Fe-S oxidoreductase	C
Bd0690	Bv0444	29347920	60680060	53711832	malate dehydrogenase	C
Bd0694c	Bv3381c	29346034	60682089	53713881	putative terpenoid biosynthesis-related protein	I
Bd0739c	Bv1637	29349787	60680546	53712377	putative oxalate:formate antiporter	n/a
Bd0741	Bv3385	29346038	60682067	53713854	conserved hypothetical protein	n/a
Bd0742c	Bv1280c	29346142	60681731	53713487	two-component system response regulator	TK
Bd0743c	Bv1281c	29346143	60681732	53713488	putative two-component system sensor histidine kinase	T
Bd0748c	Bv2566	29349709	60680466	53712288	conserved hypothetical protein, putative membrane protein	U
Bd0752c	Bv3081c	29346244	60681871	53713591	conserved hypothetical protein	R

Bd0753c	Bv3082c	29346245	60681872	53713592	queuine tRNA-ribosyltransferase	J
Bd0758c	Bv2381	29350074	60680719	53712536	putative K+ uptake system protein	P
Bd0759c	Bv2380	29350073	60680718	53712535	K+ uptake protein	P
Bd0760	Bv3851c	29346201	60681834	53713549	conserved hypothetical protein	F
Bd0761	Bv3850c	29346202	60681835	53713550	xylulose kinase (xylulokinase)	G
Bd0762	Bv3849c	29346203	60681836	53713551	xylose isomerase	G
Bd0763	Bv3968c	29346204	60681837	53713552	putative sugar-transport membrane protein	GEPR
Bd0765	Bv1387c	29348364	60683602	53715663	formate C-acetyltransferase	C
Bd0771	Bv0407c	29345821	60681167	53712955	CysQ, sulfite synthesis pathway protein	P
Bd0772	Bv0406c	29345822	60681168	53712956	putative Na+/sulfate symporter	P
Bd0773	Bv0405c	29345823	60681169	53712957	putative adenylylsulfate kinase	P
Bd0774	Bv0404c	29345824	60681170	53712958	sulfate adenylyltransferase subunit 2	EH
Bd0775	Bv0403c	29345825	60681171	53712959	sulfate adenylyltransferase subunit 1/adenylylsulfate kinase	P
Bd0776	Bv0402c	29345826	60681172	53712960	conserved hypothetical protein	n/a
Bd0777	Bv3961c	29345827	60681173	53712961	conserved hypothetical protein	n/a
Bd0778	Bv3221c	29346304	60681965	53713694	DNA ligase	L
Bd0780	Bv3220c	29346305	60681966	53713695	putative dihydrodipicolinate synthase	EM
Bd0786c	Bv3991	29346893	60681086	53712867	two-component system sensor histidine kinase	T
Bd0787c	Bv3990	29346892	60681087	53712868	two-component system response regulator	TK
Bd0791	Bv2680	29346108	60681699	53713452	3-methyl-2-oxobutanoate hydroxymethyltransferase	H
Bd0794c	Bv2299c	29345594	60682505	53714482	uridine kinase	F
Bd0795c	Bv2969c	29345986	60682115	53713906	conserved hypothetical protein	n/a
Bd0796c	Bv2970c	29345987	60682114	53713905	LysM-repeat proteins and domains	M
Bd0798	Bv0587	29349895	60682833	53714850	glycoside hydrolase family 23, related to peptidoglycan lytic transglycosylases	M
Bd0799c	Bv3792	29347981	60679979	53711746	putative glutaminase	E
Bd0800c	Bv3791	29347980	60679978	53711745	glutamate decarboxylase	E
Bd0801	Bv3790c	29347978	60679977	53711743	putative sugar transporter	GEPR
Bd0802	Bv3789c	29347977	60680130	53711903	putative permease	GER
Bd0803c	Bv2699c	29345991	60682110	53713901	putative acetyltransferase	n/a
Bd0804	Bv3476c	29346711	60682222	53714026	V-type ATP synthase subunit E	C
Bd0805	Bv3475c	29346710	60682221	53714025	conserved hypothetical protein	n/a
Bd0806	Bv3474c	29346709	60682220	53714024	V-type ATP synthase subunit A	C
Bd0807	Bv3473c	29346708	60682219	53714023	V-type ATP synthase subunit B	C
Bd0808	Bv3472c	29346707	60682218	53714022	V-type ATP synthase subunit D	C
Bd0809	Bv3471c	29346706	60682217	53714021	putative V-type ATP synthase subunit I	C
Bd0810	Bv3470c	29346705	60682216	53714020	V-type ATP synthase subunit K	C
Bd0811	Bv0114c	29349606	60680371	53712191	putative regulatory protein	n/a
Bd0814c	Bv3735	29349982	60680682	53712482	conserved hypothetical protein	D

Bd0815c	Bv2179c	29347079	60682573	53714552	phenylalanyl-tRNA synthetase alpha chain	J
Bd0816	Bv1298c	29345558	60680851	53712692	conserved hypothetical protein	R
Bd0817c	Bv3255	29346149	60681738	53713494	conserved hypothetical protein	n/a
Bd0818c	Bv3254	29346150	60681739	53713495	flavoredoxin	R
Bd0819c	Bv3253	29346151	60681740	53713496	aspartate carbamoyltransferase regulatory chain	F
Bd0820c	Bv3252	29346152	60681741	53713497	aspartate carbamoyltransferase catalytic chain	F
Bd0890c	Bv3493c	29347384	60682899	53714921	putative Xaa-Pro dipeptidase	E
Bd0891c	Bv3238c	29346180	60681209	53712998	conserved hypothetical protein	G
Bd0892	Bv2949	29346501	60681570	53713321	cation-transporting ATPase	P
Bd0894	Bv1614c	29347393	60682942	53714976	hemolysin III	R
Bd0897c	Bv0513c	29345648	60682479	53714457	transcriptional regulator	R
Bd0900c	Bv3797	29347983	60680058	53711830	glutamate/gamma-aminobutyrate anti-porter	E
Bd0901	Bv3796c	29347982	60680059	53711831	putative potassium channel subunit	n/a
Bd0902	Bv2971	29345988	60682113	53713904	excinuclease ABC subunit A	L
Bd0903	Bv2963	29346560	60681560	53713311	conserved hypothetical protein	S
Bd0905	Bv3800c	29346044	60682058	53713845	putative Na+/H+ antiporter	P
Bd0916	Bv3447c	29346067	60682035	53713817	putative helicase	L
Bd0917	Bv3446c	29346084	60682034	53713816	carboxynorspermidine decarboxylase	E
Bd0918	Bv3199	29346324	60681988	53713717	recognition particle-docking protein FtsY	U
Bd0919	Bv3200	29346323	60681987	53713716	putative Fe-S oxidoreductase	J
Bd0920	Bv3201	29346322	60681986	53713715	DNA-binding protein HU	L
Bd0922	Bv3203	29346320	60681984	53713713	magnesium chelatase, subunit I	R
Bd0923	Bv3204	29346319	60681983	53713712	conserved hypothetical protein	R
Bd0924	Bv3205	29346318	60681982	53713711	conserved hypothetical protein	n/a
Bd0925	Bv3206	29346317	60681981	53713710	conserved hypothetical protein BatA	R
Bd0926	Bv3207	29346316	60681980	53713709	aerotolerance-related membrane protein	R
Bd0928	Bv3209	29346314	60681978	53713707	BatD, conserved hypothetical protein	n/a
Bd0929	Bv3210	29346313	60681977	53713706	BatE, TRP domain containing protein	n/a
Bd0930	Bv3211	29347264	60682722	53714717	putative membrane-associated phospholipid phosphatase	n/a
Bd0931	Bv3212	29346312	60681976	53713705	conserved hypothetical protein	n/a
Bd0932	Bv3213	29346311	60681975	53713704	putative universal stress protein UspA	T
Bd0935	Bv0227c	29348092	60683395	53715422	putative periplasmic protein	n/a
Bd0937	Bv0225c	29348090	60683393	53715420	alginate O-acetylation protein	M
Bd0938c	Bv0308c	29349461	60680279	53712086	putative membrane transport protein	M
Bd0943	Bv2302	29345610	60682500	53714477	ATP phosphoribosyltransferase	E
Bd0944	Bv2303	29345611	60682499	53714476	putative histidinol dehydrogenase	E
Bd0945	Bv2304	29345612	60682498	53714475	histidinol-phosphate aminotransferase	E
Bd0946	Bv2305	29345613	60682497	53714474	histidine biosynthesis bifunctional protein HisB	EE
Bd0947c	Bv0392	29348489	60683773	53715855	putative cysteine synthase	E

Bd0949c	Bv1633	29347376	60682814	53714828	multidrug resistance protein, AcrB/AcrD family	V
Bd0950c	Bv1632	29347377	60682813	53714826	multidrug efflux protein	M
Bd0951	Bv1631c	29347378	60682812	53714824	transcriptional regulator	K
Bd0955c	Bv3011	29346138	60683537	53715587	putative transcriptional regulator	n/a
Bd0958	Bv4073	29346809	60681610	53713368	conserved hypothetical protein	n/a
Bd0959	Bv4074	29346808	60681612	53713370	putative aminotransferase	E
Bd0960c	Bv0589	29348749	60679753	53711483	glycoside hydrolase family 2, candidate beta-galactosidase	G
Bd0966	Bv3235c	29346339	60681995	53713725	prolyl-tRNA synthetase	J
Bd0991c	Bv2942	29346147	60681735	53713491	formate-tetrahydrofolate ligase	F
Bd1003c	Bv3067c	29345580	60681927	53713653	conserved hypothetical protein	S
Bd1006c	Bv2298c	29345593	60682506	53714483	glycoside hydrolase family 23, related to peptidoglycan lytic transglycosylases	M
Bd1007c	Bv3272c	29346053	60682048	53713830	RNA methyltransferase	J
Bd1008	Bv3273	29346054	60682047	53713829	pyruvate,phosphate dikinase	G
Bd1023c	Bv2478c	29349621	60680387	53712207	conserved hypothetical protein	R
Bd1024c	Bv2477c	29349620	60680386	53712206	conserved hypothetical protein	S
Bd1027	Bv3883c	29346469	60681668	53713421	putative UDP-glucose 4-epimerase	MG
Bd1034	Bv2438	29346985	60680903	53712752	putative dehydrogenase	CHR
Bd1035	Bv2019	29347192	60682654	53714639	putative phosphoesterase	R
Bd1036c	Bv3452	29346060	60682040	53713822	thiamine biosynthesis protein ThiC	H
Bd1037c	Bv3450	29346062	60682038	53713820	putative thiamine-phosphate pyrophosphorylase	H
Bd1038c	Bv3459	29346057	60682044	53713826	thiamine phosphate pyrophosphorylase	n/a
Bd1039c	Bv3884	29346200	60681833	53713548	phosphomethylpyrimidine kinase	H
Bd1040c	Bv0408c	29346572	60681547	53713298	conserved hypothetical exported protein	n/a
Bd1048	Bv2206c	29346146	60680958	53712811	putative transmembrane protein	R
Bd1050	Bv2205c	29346145	60680957	53712810	putative aspartate decarboxylase	E
Bd1051	Bv1909	29347482	60683001	53715039	aconitase protein	C
Bd1052	Bv1910	29347481	60683000	53715038	isocitrate dehydrogenase	C
Bd1053	Bv1911	29347480	60682999	53715037	citrate synthase	C
Bd1055	Bv3676	29346284	60681944	53713671	conserved hypothetical protein	n/a
Bd1058c	Bv0751	29349904	60682401	53714380	putative oxidoreductase	IQR
Bd1060	Bv2475c	29347495	60683038	53715077	putative peptidase	E
Bd1062	Bv1566c	29347291	60682744	53714738	purine nucleoside phosphorylase I	F
Bd1063c	Bv3217	29346308	60681972	53713701	negative regulator of genetic competence	O
Bd1064	Bv3216c	29346309	60681973	53713702	DNA gyrase A subunit	L
Bd1065	Bv3215c	29346310	60681974	53713703	putative tetratricopeptide repeat family protein	n/a
Bd1072	Bv2018c	29347932	60683580	53715640	Xaa-Pro aminopeptidase	E
Bd1074c	Bv3079	29346243	60681870	53713590	conserved hypothetical protein	n/a
Bd1075	Bv1580	29346716	60683378	53715412	putative ABC transporter ATP-binding protein	V

Bd1077	Bv1582	29346714	60683376	53715410	putative transcriptional regulator	n/a
Bd1080c	Bv3755c	29347194	60681857	53713575	conserved hypothetical protein	n/a
Bd1081	Bv3078c	29346242	60681869	53713589	putative phosphoserine phosphatase	TE
Bd1082	Bv2300	29345595	60682504	53714481	conserved hypothetical protein	n/a
Bd1083	Bv3805c	29346041	60682062	53713849	conserved hypothetical protein	n/a
Bd1085	Bv2552	29346040	60682064	53713851	exodeoxyribonuclease (exoA)	L
Bd1086c	Bv3224c	29346302	60681963	53713691	dihydroorotate dehydrogenase	F
Bd1087c	Bv3225c	29346301	60681962	53713690	dihydroorotate dehydrogenase, electron transfer subunit	HC
Bd1088c	Bv3226c	29346300	60681961	53713689	conserved hypothetical protein	n/a
Bd1089c	Bv3227c	29346299	60681960	53713687	conserved hypothetical protein	n/a
Bd1090c	Bv3228c	29346298	60681959	53713686	AMP nucleosidase	F
Bd1091	Bv3229	29346297	60681958	53713685	conserved hypothetical protein	n/a
Bd1094c	Bv3157	29346565	60681554	53713305	Na+-translocating NADH-quinone reductase subunit F	C
Bd1095c	Bv3156	29346566	60681553	53713304	Na+-translocating NADH-quinone reductase subunit E	C
Bd1096c	Bv3155	29346567	60681552	53713303	Na+-translocating NADH-quinone reductase subunit D	C
Bd1097c	Bv3154	29346568	60681551	53713302	Na+-translocating NADH-quinone reductase subunit C	C
Bd1098c	Bv3153	29346569	60681550	53713301	Na+-translocating NADH-quinone reductase subunit B	C
Bd1099c	Bv3152	29346570	60681549	53713300	Na+-translocating NADH-quinone reductase subunit A	C
Bd1103c	Bv1059c	29346551	60683767	53715848	cation efflux system protein	P
Bd1104c	Bv2035c	29347490	60683018	53715056	putative inner membrane protein	S
Bd1106	Bv1908c	29347483	60683002	53715040	putative helicase	L
Bd1107	Bv3166c	29348761	60679758	53711489	haloacid dehalogenase-like hydrolase	R
Bd1109	Bv2360c	29350108	60680818	53712607	isochorismate synthase EntC	HQ
Bd1110	Bv2359c	29350109	60680819	53712608	2-oxoglutarate decarboxylase	H
Bd1111	Bv2358c	29350110	60680821	53712610	dihydroxynapthoic acid synthetase	H
Bd1112	Bv2357c	29350111	60680822	53712611	putative muconate cycloisomerase	MR
Bd1113	Bv2356c	29350112	60680823	53712612	putative O-succinylbenzoate--CoA ligase	IQ
Bd1116	Bv2014c	29349803	60683241	53715306	glycoside hydrolase family 84, related to beta-N-acetylhexosaminidases	n/a
Bd1117	Bv2183c	29347081	60682575	53714554	endonuclease III	L
Bd1121c	Bv2182	29346777	60682330	53714274	metal-dependent hydrolase	R
Bd1122c	Bv2181	29346778	60682331	53714275	UDP-N-acetylenolpyruvoylglucosamine reductase	M
Bd1123	Bv3250c	29349294	60683223	53715287	conserved hypothetical protein	n/a
Bd1125	Bv0954	29348200	60683661	53715724	uracil phosphoribosyltransferase	F
Bd1133c	Bv1125	29346163	60682378	53714360	putative membrane protein	n/a
Bd1134c	Bv1124	29346162	60682380	53714361	putative ECF sigma factor	K
Bd1135c	Bv0388c	29347295	60681162	53712950	ATP-dependent RNA helicase DeaD	LKJ
Bd1136	Bv1958c	29347258	60682718	53714712	putative ATP-dependent DNA helicase	L
Bd1139	Bv2445	29347349	60682537	53714516	putative outer membrane receptor protein	n/a

Bd1147c	Bv2176	29346178	60681814	53713529	conserved hypothetical protein	n/a
Bd1148c	Bv2175	29346177	60681813	53713528	putative para-aminobenzoate synthase component I	EH
Bd1149c	Bv3141c	29346285	60681945	53713672	beta-ureidopropionase	R
Bd1150c	Bv3142c	29346286	60681946	53713673	conserved hypothetical protein	E
Bd1151c	Bv0265	29347531	60683051	53715093	putative cation transport related membrane protein	V
Bd1160	Bv3554	29347220	60681365	53713098	GAF domain-containing protein involved in signal transduction	T
Bd1161c	Bv3380	29346467	60681670	53713423	conserved hypothetical protein	n/a
Bd1162c	Bv0828c	29348088	60680312	53712124	putative ion-channel protein, putative oxidoreductase	C
Bd1163c	Bv1017	29349204	60680354	53712171	putative secreted sulfatase precursor	P
Bd1164c	Bv2968	29345985	60682116	53713907	conserved hypothetical protein	n/a
Bd1165c	Bv2967	29345984	60682117	53713908	conserved hypothetical protein	n/a
Bd1166c	Bv2966	29345983	60682118	53713909	conserved hypothetical protein	n/a
Bd1167c	Bv2218	29345982	60682119	53713910	conserved hypothetical protein	R
Bd1168c	Bv2217	29345981	60682120	53713911	phenylacetate-coenzyme A ligase	H
Bd1170	Bv2679	29346107	60681696	53713449	putative phosphatase/phosphohexomutase	R
Bd1172c	Bv3709c	29346959	60680920	53712770	putative secreted peptidase	n/a
Bd1174c	Bv2376	29350059	60680716	53712533	endonuclease IV	L
Bd1175c	Bv2301	29345609	60682501	53714478	conserved hypothetical protein	n/a
Bd1177	Bv3448	29346065	60682036	53713818	superoxide dismutase	P
Bd1187	Bv2692c	29346102	60681688	53713441	putative calcineurin superfamily phosphohydrolase	n/a
Bd1188	Bv2691c	29346101	60681687	53713440	conserved hypothetical protein	n/a
Bd1189c	Bv2201c	29347848	60680090	53711862	conserved hypothetical protein	n/a
Bd1190c	Bv2202c	29347847	60680091	53711863	conserved hypothetical protein	n/a
Bd1191c	Bv3881	29346594	60681531	53713283	conserved hypothetical protein	n/a
Bd1192c	Bv3258	29346604	60681448	53713187	outer membrane protein, OmpA family	M
Bd1193c	Bv1840	29347638	60680204	53712003	conserved hypothetical protein	n/a
Bd1195c	Bv3719	29350005	60680697	53712497	endopeptidase Clp ATP-binding chain B	O
Bd1198	Bv0400	29349277	60683240	53715305	conserved hypothetical protein	S
Bd1205c	Bv3384	29346037	60682068	53713855	conserved hypothetical protein, with a conserved domain of unknown function	S
Bd1206c	Bv3383	29346036	60682069	53713856	phenylalanyl-tRNA synthetase beta chain	JR
Bd1207	Bv3222	29346303	60681964	53713693	tRNA (Guanine-N(1)-)-methyltransferase	J
Bd1208c	Bv0958	29346780	60682333	53714277	NAD dependent nucleotide-diphosphate-sugar epimerase	MG
Bd1209	Bv0957c	29346781	60682334	53714278	2-amino-3-ketobutyrate coenzyme A ligase	H
Bd1212c	Bv2270c	29346654	60681310	53713031	chaperone protein dnaJ	O
Bd1213c	Bv2271c	29346653	60681311	53713032	GrpE protein	O
Bd1215	Bv2390c	29350088	60680741	53712559	DNA mismatch repair protein MutS	L
Bd1216	Bv2636	29350087	60680740	53712558	Mg2+/Co2+ transport protein	P
Bd1238c	Bv2038	29350147	60681592	53713347	transposase	L

Bd1240	Bv2682	29346596	60681528	53713280	ABC transporter ATP-binding protein	R
Bd1242	Bv3256	29346148	60681737	53713493	serine hydroxymethyltransferase	E
Bd1244c	Bv3444c	29346214	60681839	53713554	putative carbon-nitrogen hydrolase	R
Bd1246	Bv3852	29345934	60682156	53713952	putative two-component system response regulator	T
Bd1247	Bv3853	29345933	60682157	53713953	putative glycosyltransferase	M
Bd1248	Bv3854	29346573	60681546	53713297	conserved hypothetical protein	n/a
Bd1249	Bv3855	29346574	60681545	53713296	conserved hypothetical protein	n/a
Bd1250	Bv3856	29346575	60681544	53713295	conserved hypothetical protein	n/a
Bd1253	Bv3860	29345931	60682159	53713955	putative acetyltransferase	R
Bd1256	Bv3873	29346588	60681539	53713290	conserved hypothetical protein	n/a
Bd1258	Bv3876	29346591	60681536	53713287	glycosyltransferase family 2, related to beta-glycosyltransferases	M
Bd1267	Bv3857	29346576	60681543	53713294	glycosyltransferase family 2, related to beta-glycosyltransferases	R
Bd1269	Bv3882c	29346468	60681669	53713422	two-component system sensor histidine kinase	TT
Bd1270	Bv2027	29349861	60682862	53714881	6-pyruvoyl-tetrahydropterin synthase	H
Bd1271	Bv2028	29349862	60682861	53714880	conserved hypothetical protein	O
Bd1272c	Bv2199c	29345660	60682435	53714416	dihydroorotase (DHOase)	F
Bd1273c	Bv2200c	29345661	60682434	53714415	glycosyltransferase family 2, candidate beta-glycosyltransferase	M
Bd1280c	Bv3971c	29347105	60682621	53714599	outer membrane efflux protein	MU
Bd1281c	Bv3972c	29347104	60682620	53714598	transporter, AcrB/D/F family	V
Bd1282c	Bv3973c	29347103	60682619	53714597	periplasmic linker protein, putative multidrug resistance protein	M
Bd1283c	Bv3948	29345842	60681198	53712986	putative endonuclease BB0411	F
Bd1297	Bv0307	29348259	60683575	53715633	putative two-component system sensor histidine kinase	T
Bd1303c	Bv3247	29345974	60682126	53713919	putative ABC transporter permease protein	V
Bd1311c	Bv2384c	29350086	60680739	53712557	L-serine dehydratase	E
Bd1333	Bv3485	29349231	60682741	53714735	conserved hypothetical protein	R
Bd1337	Bv1627c	29345838	60681194	53712982	phenylacetate-coenzyme A ligase	H
Bd1343	Bv2831c	29346648	60681362	53713095	putative RNA-binding protein	K
Bd1344	Bv2972	29345989	60682112	53713903	putative transcriptional regulator	S
Bd1346	Bv3144c	29346740	60682250	53714056	conserved hypothetical protein	n/a
Bd1347	Bv3974	29346500	60681571	53713322	transcriptional regulator	K
Bd1348	Bv2008c	29349344	60683160	53715226	prephenate dehydratase	E
Bd1349	Bv2007c	29349343	60683161	53715227	putative aminotransferase	E
Bd1350	Bv2006c	29349342	60683162	53715228	putative phospho-2-dehydro-3-deoxyheptonate aldolase	E
Bd1351	Bv2005c	29349341	60683163	53715229	chorismate mutase/prephenate dehydratase (tyrA)	E
Bd1354	Bv0370	29349212	60683363	53715395	putative multidrug efflux protein	V
Bd1356	Bv3377	29346100	60681686	53713439	two-component system response regulator	T
Bd1357	Bv3378	29346099	60681685	53713438	putative two-component system sensor histidine kinase	TT
Bd1358	Bv2676	29346039	60682065	53713852	Mn2+ and Fe2+ transport protein	P
Bd1359	Bv3837	29346735	60682245	53714050	glutaminyl-tRNA synthetase	J

Bd1360	Bv3838	29346736	60682246	53714051	putative N-acetylglucosamine transferase	R
Bd1362	Bv2254c	29345741	60681150	53712938	ketoisovalerate oxidoreductase subunit VorB	C
Bd1363	Bv2252c	29345740	60681148	53712936	ketoisovalerate oxidoreductase subunit VorA	C
Bd1364	Bv2251c	29345739	60681147	53712935	ketoisovalerate oxidoreductase subunit VorA	C
Bd1365	Bv2250c	29345738	60681146	53712934	conserved hypothetical protein	n/a
Bd1366	Bv2249c	29345737	60681145	53712933	glutamine ABC transporter, periplasmic glutamine-binding protein (glnH)	ET
Bd1368	Bv3077c	29346241	60681868	53713588	ATP-dependent RNA helicase	LKJ
Bd1370c	Bv2815	29347003	60680868	53712709	putative cell-cycle protein	D
Bd1371c	Bv3230c	29346293	60681954	53713681	conserved hypothetical protein	R
Bd1372c	Bv3231c	29346292	60681953	53713680	conserved hypothetical protein	SS
Bd1373c	Bv3138	29346282	60681943	53713670	aspartyl-tRNA synthetase	J
Bd1400	Bv2216	29346487	60681591	53713346	putative aspartate aminotransferase	E
Bd1401	Bv2901c	29346488	60681590	53713345	putative transcriptional regulator	K
Bd1402c	Bv2900	29346489	60681589	53713344	putative acetyltransferase	J
Bd1404c	Bv2898	29346491	60681587	53713342	recombination protein recR	L
Bd1406	Bv2690c	29346496	60681574	53713327	putative oligopeptide transporter	S
Bd1407	Bv2535c	29349029	60679961	53711723	amidase enhancer precursor	D
Bd1410	Bv2896c	29346492	60681584	53713338	Na+/solute symporter	ER
Bd1411	Bv2895c	29346493	60681583	53713337	putative GTP-binding protein	R
Bd1415	Bv2829c	29346639	60681373	53713107	putative membrane protein	R
Bd1418c	Bv1279	29348417	60683732	53715803	conserved hypothetical protein	n/a
Bd1420	Bv2320c	29350020	60680707	53712507	conserved hypothetical protein	E
Bd1421	Bv2319c	29350019	60680706	53712506	putative bacterioferritin co-migratory protein	O
Bd1422	Bv2318c	29350018	60680705	53712505	RecA	L
Bd1433c	Bv3074	29346239	60681866	53713586	UDP-glucose 6-dehydrogenase	M
Bd1444	Bv0197c	29347915	60680061	53711833	conserved hypothetical protein	S
Bd1445c	Bv3801c	29346042	60682060	53713847	GTP-binding protein, membrane GTPase lepA	M
Bd1446	Bv2209c	29347085	60682580	53714559	conserved hypothetical protein	R
Bd1448c	Bv2308	29345626	60682488	53714465	putative rubrerythrin	C
Bd1450c	Bv3270	29345997	60682106	53713897	prolyl oligopeptidase family protein	E
Bd1458c	Bv1988	29347822	60680118	53711890	putative collagenase	O
Bd1459c	Bv1987	29347823	60680117	53711889	homoserine O-succinyltransferase	E
Bd1460c	Bv3688	29345716	60682415	53714395	outer membrane protein oprM precursor	MU
Bd1461c	Bv3687	29345715	60682416	53714396	putative aminoglycoside efflux pump (Acriflavine resistance protein)	V
Bd1462c	Bv3686	29345714	60682417	53714397	acriflavine resistance protein A precursor	M
Bd1464c	Bv0374c	29349180	60683368	53715401	ribosomal large subunit pseudouridine synthase D	J
Bd1465c	Bv0375c	29349179	60683369	53715402	3-oxoacyl-[acyl-carrier protein] reductase	IQR

Bd1467c	Bv0376c	29349178	60683370	53715403	transcriptional regulator	n/a
Bd1470c	Bv2471c	29345837	60681193	53712981	xanthine phosphoribosyltransferase	F
Bd1471c	Bv2470c	29349881	60682846	53714864	putative purine permease	F
Bd1473c	Bv1654c	29347225	60682681	53714670	low-specificity L-threonine aldolase	E
Bd1487c	Bv2227	29345717	60682408	53714387	phosphofructokinase	G
Bd1489	Bv3466	29346702	60682212	53714016	thiol:disulfide interchange protein	OC
Bd1491	Bv2454c	29345584	60682515	53714493	conserved hypothetical protein	n/a
Bd1492	Bv2453c	29345583	60682516	53714494	conserved hypothetical protein	n/a
Bd1495	Bv0255	29346227	60682659	53714645	lipoprotein, function unknown	n/a
Bd1496	Bv1130	29346953	60680932	53712783	putative ATP-binding ABC transporter protein	R
Bd1498c	Bv2816c	29346769	60682322	53714266	tetratricopeptide repeat family protein	R
Bd1500c	Bv3478c	29346367	60682011	53713791	conserved hypothetical protein	V
Bd1501c	Bv3479c	29346366	60682010	53713790	putative membrane protein	V
Bd1502c	Bv3480c	29346365	60682009	53713789	conserved hypothetical protein, putative membrane protein	M
Bd1503c	Bv3481c	29346364	60682008	53713788	conserved hypothetical protein	MU
Bd1506c	Bv3072c	29346236	60681863	53713583	conserved hypothetical protein	n/a
Bd1511c	Bv0460	29349325	60683186	53715252	putative inorganic polyphosphate/ATP-NAD kinase	G
Bd1513	Bv0459c	29349326	60683185	53715251	pyridoxal phosphate biosynthetic protein pdxJ	H
Bd1514	Bv0458c	29349327	60683184	53715250	putative biopolymer transport protein ExbB	U
Bd1515	Bv0457c	29349328	60683183	53715249	conserved hypothetical protein	n/a
Bd1517	Bv0455c	29349330	60683181	53715247	putative ThiJ family intracellular protease	R
Bd1525c	Bv0818c	29349021	60682616	53714594	transcriptional regulator	K
Bd1526	Bv1347c	29349110	60682615	53714593	outer membrane protein, probably involved in nutrient binding	n/a
Bd1531c	Bv1978	29349219	60683355	53715386	putative endothelin-converting enzyme	O
Bd1533	Bv0364c	29349216	60683359	53715391	conserved hypothetical protein	n/a
Bd1538	Bv0368	29349210	60683365	53715397	peptidyl-prolyl cis-trans isomerase, cyclophilin-type	O
Bd1539	Bv1850c	29349817	60680582	53712423	DNA damage-inducible protein	L
Bd1541c	Bv1570	29347538	60682086	53713878	conserved hypothetical protein	R
Bd1543	Bv0452c	29349333	60683178	53715244	nucleoside diphosphate kinase	F
Bd1571	Bv2724c	29346965	60680917	53712767	putative dioxygenase	R
Bd1572c	Bv1982	29347450	60682971	53715008	conserved hypothetical protein	n/a
Bd1573c	Bv1981	29347449	60682970	53715007	AcrB/AcrD/AcrF family cation efflux system protein	P
Bd1574c	Bv1980	29347448	60682969	53715006	putative metal resistance related exported protein	M
Bd1576	Bv3742	29345570	60680864	53712705	conserved hypothetical protein	n/a
Bd1584c	Bv0266	29347503	60683046	53715087	conserved hypothetical protein	n/a
Bd1588c	Bv0606c	29349398	60680241	53712046	glycoside hydrolase family 92, related to an ill-defined alpha-1,2-mannosidase	G
Bd1599	Bv0717	29349804	60680410	53712231	transcriptional regulator	K
Bd1611	Bv2187	29346977	60680907	53712757	conserved hypothetical protein	S

Bd1612c	Bv1269c	29347952	60683570	53715627	putative haloacid dehalogenase-like hydrolase	R
Bd1613c	Bv3462c	29346698	60682208	53714012	putative ABC transport system, lipoprotein	E
Bd1614c	Bv3463c	29346699	60682209	53714013	putrescine transport system permease protein PotI	E
Bd1615c	Bv3464c	29346700	60682210	53714014	putrescine transport system permease protein PotH	E
Bd1616c	Bv3465c	29346701	60682211	53714015	spermidine/putrescine transport ATP-binding protein	E
Bd1627c	Bv2009c	29346672	60682195	53713995	putative transcriptional regulator	K
Bd1632	Bv2268c	29345782	60681157	53712945	aldose 1-epimerase precursor	G
Bd1633	Bv2267c	29345781	60681156	53712944	glucose/galactose transporter	G
Bd1634	Bv2266c	29345780	60681155	53712943	galactokinase	G
Bd1636	Bv2259c	29345757	60681154	53712942	transketolase	G
Bd1637	Bv2258c	29345756	60681153	53712941	ribose 5-phosphate isomerase B	G
Bd1655	Bv1905c	29347499	60683041	53715082	putative DNA topoisomerase	L
Bd1656	Bv0331	29349380	60683104	53715169	putative NTP pyrophosphohydrolase	n/a
Bd1659c	Bv0046c	29349358	60683117	53715182	phosphoglucomutase/phosphomannomutase	G
Bd1660c	Bv0047c	29349357	60683118	53715183	conserved hypothetical protein	n/a
Bd1661c	Bv0048c	29349356	60683119	53715184	putative exopolyphosphatase-related protein	R
Bd1663c	Bv0050c	29349354	60683121	53715186	ribulose-phosphate 3-epimerase	G
Bd1665c	Bv2246	29345726	60681138	53712923	conserved hypothetical exported protein	n/a
Bd1667	Bv0202c	29349046	60679970	53711736	putative Na+/H+ antiporter	P
Bd1669c	Bv2993c	29346232	60681860	53713580	putative metallo-beta-lactamase superfamily hydrolase	R
Bd1670c	Bv3989	29345936	60682154	53713950	L-asparaginase I	EJ
Bd1671c	Bv3265c	29346611	60681438	53713177	conserved hypothetical protein	S
Bd1673c	Bv0299c	29347445	60682968	53715002	putative ABC transporter ATP-binding protein	R
Bd1679	Bv0335c	29347511	60683047	53715088	putative transmembrane cation efflux protein	V
Bd1680	Bv0334c	29347512	60683048	53715089	putative regulatory protein	n/a
Bd1683c	Bv2908	29346098	60681684	53713437	transcriptional regulator	T
Bd1684	Bv2907c	29346097	60681683	53713436	prismane protein	C
Bd1685	Bv0843	29349866	60682857	53714876	pyridoxine kinase	H
Bd1686	Bv1172c	29348526	60683777	53715859	putative oxidoreductase	H
Bd1687	Bv0825	29347810	60683149	53715214	DNA-3-methyladenine glycosylase I	L
Bd1688c	Bv1657	29345783	60681158	53712946	mannose-6-phosphate isomerase	G
Bd1689c	Bv3237c	29346181	60681816	53713532	glycoside hydrolase family 13, candidate 1,4-alpha-glucan branching enzyme	G
Bd1691	Bv2474c	29345591	60682508	53714485	putative NADH dehydrogenase/NAD(P)H nitroreductase	C
Bd1693c	Bv0469c	29345654	60682452	53714434	putative exonuclease	L
Bd1696	Bv0237	29347379	60682870	53714890	NADP-dependent malate dehydrogenase	CC
Bd1699	Bv0311c	29346294	60681955	53713682	outer membrane efflux protein oprM precursor	MU
Bd1700	Bv0310c	29346295	60681956	53713683	putative cation efflux pump	M
Bd1701	Bv0309c	29346296	60681957	53713684	putative cation efflux pump	V

Bd1702c	Bv1135	29345575	60681559	53713310	peptidyl-prolyl cis-trans isomerase	O
Bd1705c	Bv0054c	29349353	60683122	53715187	methionyl-tRNA formyltransferase	J
Bd1706c	Bv0055c	29349352	60683123	53715188	putative chloride channel protein	P
Bd1708c	Bv0056c	29349351	60683124	53715189	conserved hypothetical protein, putative translation factor	J
Bd1709	Bv2016c	29349347	60683150	53715216	putative single-stranded-DNA-specific exonuclease	L
Bd1710	Bv2015c	29349346	60683151	53715217	ATP-dependent DNA helicase RecQ	L
Bd1717c	Bv1992c	29347817	60680122	53711894	NAD-utilizing dehydrogenase	R
Bd1718c	Bv1994c	29347816	60680124	53711896	putative DNA repair protein	O
Bd1720c	Bv0610c	29349402	60680245	53712050	glycoside hydrolase family 92, related to an ill-defined alpha-1,2-mannosidase	G
Bd1722c	Bv3712c	29349511	60680366	53712183	Sec-independent protein translocase	U
Bd1724	Bv3885	29346199	60681832	53713547	malonyl CoA-acyl carrier protein transacylase	I
Bd1731c	Bv1818c	29347599	60680216	53712016	aminopeptidase	R
Bd1732c	Bv0333c	29347091	60682594	53714572	conserved hypothetical protein	P
Bd1733c	Bv0536	29349539	60680459	53712281	conserved hypothetical protein	R
Bd1738c	Bv3690	29347156	60682635	53714616	conserved hypothetical protein	R
Bd1739c	Bv0261c	29349300	60683217	53715281	branched-chain amino acid aminotransferase	EH
Bd1741	Bv1784c	29349229	60683345	53715376	5,10-methylenetetrahydrofolate reductase	E
Bd1742	Bv1783c	29349228	60683346	53715377	putative DNA polymerase III, delta subunit	LL
Bd1744	Bv1937	29349597	60680445	53712265	conserved hypothetical protein	n/a
Bd1745	Bv2365	29350058	60680715	53712532	putative transporter periplasmic binding protein	P
Bd1746c	Bv2375	29346983	60680905	53712755	conserved hypothetical protein	n/a
Bd1754c	Bv2660	29346646	60681364	53713097	2',3'-cyclic-nucleotide 2'-phosphodiesterase precursor	F
Bd1758c	Bv2215	29345839	60681195	53712983	indolepyruvate oxidoreductase subunit IorB	C
Bd1759c	Bv2214	29345840	60681196	53712984	indolepyruvate oxidoreductase subunit IorA	C
Bd1760c	Bv3950	29345841	60681197	53712985	putative aminodeoxychorismate lyase	R
Bd1776c	Bv3251c	29346153	60681755	53713516	glycosyltransferase family 51, candidate bifunctional family GT51 beta-glycosyltransferase/PBP transpeptidase (candidate murein polymerase)	M
Bd1778c	Bv3717c	29350007	60680699	53712499	putative transcriptional regulator	K
Bd1780	Bv2982	29345559	60680852	53712693	peptidase	O
Bd1782	Bv3516c	29349737	60680492	53712318	conserved hypothetical protein	S
Bd1784	Bv3515c	29349736	60680491	53712317	conserved hypothetical protein	S
Bd1787c	Bv3491	29349673	60680438	53712261	glutaminne-hydrolyzing GMP synthase	FF
Bd1790	Bv0290	29347426	60682962	53714996	dTDP-glucose 4,6-dehydratase	M
Bd1804	Bv0445	29349340	60683165	53715231	DNA primase	L
Bd1807	Bv1873c	29349416	60680262	53712068	conserved hypothetical protein	S
Bd1808	Bv1872c	29349415	60680261	53712067	AAA-metalloprotease FtsH with ATPase domain	O
Bd1809	Bv1871c	29349414	60680260	53712066	phosphatidate cytidylyltransferase	I

Bd1811c	Bv3726	29349998	60680690	53712490	conserved hypothetical protein	n/a
Bd1812	Bv3725c	29349999	60680691	53712491	putative N utilization substance protein	K
Bd1813	Bv3724c	29350000	60680692	53712492	conserved hypothetical protein with conserved domain	U
Bd1814	Bv3723c	29350001	60680693	53712493	conserved hypothetical protein	n/a
Bd1815	Bv3722c	29350002	60680694	53712494	putative dephospho-CoA kinase	H
Bd1816	Bv3721c	29350003	60680695	53712495	conserved hypothetical protein	n/a
Bd1828c	Bv3083c	29346247	60681873	53713593	ATP-dependent protease	O
Bd1829	Bv3084	29346248	60681874	53713594	conserved hypothetical protein	R
Bd1830c	Bv3085c	29346281	60681942	53713669	conserved hypothetical protein	IR
Bd1831	Bv3086	29346280	60681941	53713668	8-amino-7-oxononanoate synthase	H
Bd1832	Bv1782c	29349227	60683347	53715378	conserved hypothetical protein	S
Bd1834c	Bv1780	29349225	60683349	53715380	rod shape-determining protein	D
Bd1835c	Bv1779	29349224	60683350	53715381	penicillin-binding protein 2	M
Bd1836c	Bv1778	29349223	60683351	53715382	conserved hypothetical protein	n/a
Bd1837c	Bv1777	29349222	60683352	53715383	rod shape-determining protein	n/a
Bd1838c	Bv1776	29349221	60683353	53715384	rod shape-determining protein	D
Bd1839c	Bv1775	29349220	60683354	53715385	phosphoribosylaminoimidazole carboxy formyl formyltransferase	F
Bd1843	Bv2272	29346652	60681312	53713033	ABC transporter ATP-binding protein	R
Bd1844	Bv1048c	29347263	60682721	53714716	putative sulfatase	M
Bd1845c	Bv2023c	29349825	60682867	53714887	HD superfamily hydrolase	R
Bd1846c	Bv2024c	29349826	60682868	53714888	conserved hypothetical protein	n/a
Bd1847c	Bv2025c	29349827	60682869	53714889	conserved hypothetical protein	n/a
Bd1849c	Bv1644	29349798	60680577	53712415	conserved hypothetical protein	S
Bd1850c	Bv1643	29349797	60680576	53712414	conserved hypothetical protein	S
Bd1851c	Bv1642	29349796	60680575	53712413	GTP-binding protein	R
Bd1852c	Bv1641	29349795	60680574	53712412	adenylate kinase (ATP-AMP transphosphatase)	F
Bd1853c	Bv1640	29349794	60680573	53712411	putative hypoxanthine guanine phosphoribosyltransferase	F
Bd1854	Bv1639c	29349791	60680549	53712380	putative sugar kinase	SG
Bd1864	Bv1638c	29349790	60680548	53712379	conserved hypothetical protein	n/a
Bd1865	Bv2973	29345990	60682111	53713902	putative H+/peptide symporter	E
Bd1869	Bv2714	29346853	60681112	53712893	8-amino-7-oxononanoate synthase	H
Bd1872	Bv2717	29346856	60681110	53712891	dethiobiotin synthetase	H
Bd1874	Bv2628	29347774	60679937	53711698	putative outer membrane protein, probably involved in nutrient binding	n/a
Bd1875	Bv2629	29347775	60679938	53711699	putative outer membrane protein, probably involved in nutrient binding	n/a
Bd1876c	Bv2670	29346951	60680934	53712785	conserved hypothetical protein	S
Bd1877c	Bv2669	29346950	60680935	53712786	transcriptional regulator	K
Bd1878c	Bv1276	29346866	60682167	53713964	thioredoxin (TRX)	OC

Bd1884	Bv2911	29346338	60682225	53714028	two-component system response regulator	TK
Bd1885	Bv2912	29346337	60682226	53714029	two-component system sensor histidine kinase	T
Bd1887	Bv1906	29346332	60682228	53714031	conserved hypothetical protein	n/a
Bd1888c	Bv1628	29349647	60680400	53712220	conserved hypothetical protein	n/a
Bd1889c	Bv3707c	29349646	60680399	53712219	putative permease	n/a
Bd1890c	Bv2485	29349645	60680398	53712218	tRNA pseudouridine synthase A	J
Bd1891	Bv2625	29345910	60682175	53713972	putative transport protein	R
Bd1894	Bv0485	29346131	60681715	53713469	DNA repair and recombination protein, putative helicase	L
Bd1895c	Bv1152c	29347410	60682946	53714979	MFS Superfamily multidrug efflux transporter	GEPR
Bd1896c	Bv2317	29346494	60681582	53713336	conserved hypothetical protein	n/a
Bd1898c	Bv1630	29347101	60682607	53714585	fructose-bisphosphate aldolase	G
Bd1899	Bv1629c	29347102	60682609	53714587	50S ribosomal protein L31 type B	J
Bd1900	Bv1773c	29349648	60680401	53712221	conserved hypothetical protein, with a phosphotransferase enzyme family domain	n/a
Bd1902c	Bv2296	29346938	60680956	53712809	two-component system sensor histidine kinase	T
Bd1904c	Bv2294	29346940	60680955	53712807	putative outer membrane protein OprM precursor	n/a
Bd1918c	Bv2287	29346945	60680949	53712801	ABC transporter ATP-binding protein	V
Bd1925c	Bv2284	29346946	60680939	53712790	ABC transporter permease	M
Bd1930c	Bv4029c	29345871	60681307	53713027	glycoside hydrolase family 2, candidate beta-galactosidase	G
Bd1931	Bv2725	29348165	60683472	53715499	anaerobic C4-dicarboxylate transporter dcuB	R
Bd1939c	Bv2484	29349627	60680393	53712213	conserved hypothetical protein	n/a
Bd1940	Bv2483c	29349626	60680392	53712212	phosphate starvation-inducible protein PhoH	T
Bd1941	Bv2482c	29349625	60680391	53712211	phosphoribosylamidoimidazole-succinocarboxamide synthase	F
Bd1942	Bv2481c	29349624	60680390	53712210	ubiquinone/menaquinone biosynthesis methyltransferase	H
Bd1943	Bv2480c	29349623	60680389	53712209	shikimate 5-dehydrogenase	E
Bd1944c	Bv3993c	29346905	60681074	53712850	conserved hypothetical protein	n/a
Bd1946c	Bv3994c	29346906	60681073	53712849	putative transmembrane CorC/HlyC family transporter associated protein	R
Bd1947c	Bv3995c	29346907	60681072	53712848	single-strand binding protein	L
Bd1948c	Bv3996c	29346908	60681070	53712846	A/G-specific adenine glycosylase	L
Bd1949	Bv3997	29346909	60681069	53712845	DNA-binding protein HU	L
Bd1950	Bv3998	29346910	60681067	53712844	ribonuclease G	J
Bd1951	Bv2323	29350023	60680714	53712517	chaperone protein DnaK	O
Bd1954c	Bv2534c	29349028	60679960	53711722	beta-lactamase induction signal transducer	GEPR
Bd1955c	Bv2213	29345669	60682421	53714401	putative amidohydrolase	R
Bd1956c	Bv2225c	29345670	60682420	53714400	acyltransferase family protein	I
Bd1957c	Bv2226c	29345671	60682419	53714399	conserved hypothetical protein	n/a
Bd1958	Bv3469c	29346704	60682215	53714019	glycosyltransferase family 3, candidate alpha-glycosyltransferase; related to glycogen synthases	n/a

Bd1959	Bv3468c	29346703	60682214	53714018	glycosyltransferase family 35, candidate alpha-glucan	G
Bd1960c	Bv2155c	29346984	60680904	53712753	putative outer membrane protein precursor	n/a
Bd1968	Bv3747	29346790	60682361	53714343	imidazole glycerol phosphate synthase subunit hisH	E
Bd1969	Bv3748	29346789	60682360	53714342	imidazole-4-carboxamide isomerase	E
Bd1970	Bv3749	29346788	60682359	53714341	imidazole glycerol phosphate synthase subunit hisF	E
Bd1971	Bv3750	29346787	60682358	53714340	phosphoribosyl-AMP cyclohydrolase/phosphoribosyl-ATP pyrophosphohydrolase	EE
Bd1972	Bv3751	29346786	60682357	53714339	putative ATP-binding protein involved in cell division	D
Bd1973	Bv3752	29346785	60682356	53714338	aspartokinase	E
Bd1974	Bv3753	29346784	60682355	53714337	diaminopimelate decarboxylase	E
Bd1975	Bv3756	29346882	60681099	53712881	glycosyltransferase family 2, distantly related to glycosyltransferases	n/a
Bd1977c	Bv3760c	29346885	60681098	53712880	ABC transporter permease protein	M
Bd1978c	Bv3761c	29346886	60681097	53712879	aspartate aminotransferase	E
Bd1985c	Bv2866	29346889	60681092	53712874	putative ATP-binding component of ABC transporter	R
Bd1991	Bv0613	29346840	60682366	53714348	transcriptional regulator	n/a
Bd1996c	Bv2034	29349902	60682404	53714383	conserved hypothetical protein	R
Bd2003c	Bv2696	29346111	60681703	53713456	conserved hypothetical protein	n/a
Bd2004	Bv2695c	29346110	60681702	53713455	GTP pyrophosphokinase	TK
Bd2005c	Bv1615c	29347385	60682903	53714925	putative polyA polymerase	J
Bd2009c	Bv1944c	29347799	60680134	53711907	putative transport protein	M
Bd2010	Bv1956	29347261	60682719	53714714	conserved hypothetical protein	R
Bd2011c	Bv1953	29347267	60682733	53714729	3-isopropylmalate dehydrogenase	CE
Bd2013c	Bv1952	29347268	60682734	53714730	2-isopropylmalate synthase	E
Bd2014c	Bv1951	29347269	60682735	53714731	3-isopropylmalate dehydratase small subunit	E
Bd2015c	Bv1950	29347270	60682736	53714732	3-isopropylmalate dehydratase large subunit	E
Bd2016c	Bv1949	29347271	60682737	53714733	2-isopropylmalate synthase	E
Bd2021c	Bv2497	29347484	60683004	53715042	ketol-acid reductoisomerase	EH
Bd2022c	Bv2496	29347485	60683006	53715044	acyl-ACP thioesterase	n/a
Bd2023c	Bv2495	29347486	60683007	53715045	acetohydroxyacid synthase small subunit	E
Bd2024c	Bv2494	29347487	60683008	53715046	acetolactate synthase large subunit	EH
Bd2025c	Bv2493	29347488	60683009	53715047	dihydroxy-acid dehydratase	EG
Bd2032	Bv2011	29349546	60680460	53712282	putative aminotransferase	E
Bd2033	Bv2491c	29347489	60683016	53715054	peptidyl-prolyl cis-trans isomerase	O
Bd2034	Bv2476c	29347494	60683037	53715076	chorismate synthase	E
Bd2036c	Bv2315c	29346972	60680910	53712760	conserved hypothetical protein	S
Bd2038	Bv2316	29346970	60680912	53712762	nicotinate-nucleotide pyrophosphorylase	H
Bd2040c	Bv2190c	29347020	60682540	53714518	putative DNA polymerase III	L
Bd2042	Bv2192	29347022	60682542	53714520	conserved hypothetical protein	n/a

Bd2043c	Bv2311c	29347023	60682546	53714524	conserved hypothetical protein	n/a
Bd2044	Bv2313	29347025	60682548	53714526	aminoacyl-histidine dipeptidase	E
Bd2046	Bv2314	29347026	60682549	53714527	conserved hypothetical protein	n/a
Bd2048	Bv3710c	29346960	60680919	53712769	conserved hypothetical protein	M
Bd2049	Bv3708c	29346958	60680921	53712771	putative phosphoglucomutase	G
Bd2050c	Bv2275c	29346949	60680936	53712787	cytidine deaminase	F
Bd2051	Bv2928c	29346992	60680894	53712741	putative methyltransferase	n/a
Bd2052	Bv2283	29346948	60680937	53712788	glycoside hydrolase family 73, related to peptidoglycan hydrolases	NU
Bd2053	Bv0092	29349619	60680384	53712204	putative phosphoribosylformylglycinamidine cyclo-ligase	F
Bd2054	Bv0094	29349618	60680383	53712203	peptide chain release factor 1	J
Bd2055	Bv0095	29349617	60680382	53712202	orotidine 5'-phosphate decarboxylase	F
Bd2056	Bv0097	29349615	60680380	53712200	UDP-3-O-[3-hydroxymyristoyl] glucosamine N-acyltransferase	M
Bd2057	Bv0098	29349614	60680379	53712199	UDP-3-O-[3-hydroxymyristoyl] N-acetylglucosamine deacetylase	MI
Bd2058	Bv0099	29349613	60680378	53712198	acyl-[acyl-carrier-protein]--UDP-N-acetylglucosamine O-acyltransferase	M
Bd2062	Bv0100	29349612	60680377	53712197	conserved hypothetical protein	n/a
Bd2063	Bv0101	29349611	60680376	53712196	tRNA delta(2)-isopentenylpyrophosphate transferase	J
Bd2065c	Bv3549c	29349966	60680672	53712471	conserved hypothetical protein	D
Bd2066	Bv3550	29349965	60680671	53712470	conserved hypothetical protein	n/a
Bd2067	Bv3551	29349964	60680670	53712469	conserved hypothetical protein	n/a
Bd2068	Bv3552	29349963	60680669	53712468	conserved hypothetical protein	n/a
Bd2069	Bv3553	29349962	60680668	53712467	purine nucleoside phosphorylase II	F
Bd2071c	Bv0002c	29347554	60683068	53715110	putative oxidoreductase	C
Bd2074	Bv3555	29349959	60680664	53712463	putative GTPase, ThdF family	R
Bd2076	Bv3627	29349957	60680662	53712460	integrase	n/a
Bd2081	Bv3577	29345639	60683500	53715553	conserved hypothetical protein	n/a
Bd2122c	Bv3674c	29349920	60680795	53712582	putative zinc-binding alcohol dehydrogenase	ER
Bd2126	Bv3677	29346425	60680599	53712437	putative oxidoreductase	IQR
Bd2130	Bv3453	29349913	60682041	53713823	conserved hypothetical protein	n/a
Bd2132	Bv3683	29349909	60680595	53712433	ribosomal large subunit pseudouridine synthase A	J
Bd2137c	Bv1938	29349598	60680446	53712266	conserved hypothetical protein	R
Bd2138	Bv1904	29347452	60682975	53715011	tRNA and rRNA cytosine-C5-methylase	J
Bd2141c	Bv0288c	29347429	60682965	53714999	polyphosphate kinase	P
Bd2142	Bv0289	29347428	60682964	53714998	putative phosphoesterase	R
Bd2144c	Bv1940c	29349600	60680448	53712268	putative lipoic acid synthetase	H
Bd2145c	Bv1941c	29349601	60680449	53712269	dipeptidyl peptidase IV	E
Bd2146	Bv1942	29349533	60680452	53712274	conserved hypothetical protein	R
Bd2147c	Bv0581	29346610	60681439	53713178	conserved hypothetical protein	n/a
Bd2150	Bv1270c	29347951	60683571	53715628	putative transmembrane calcium-transporting ATPase	P

Bd2155c	Bv2588c	29346634	60681378	53713112	GDP-mannose 4,6-dehydratase	M
Bd2201c	Bv2017c	29347199	60682664	53714650	aminopeptidase C	n/a
Bd2202	Bv0571c	29349165	60680051	53711821	pyrroline-5-carboxylate reductase	E
Bd2206	Bv0557c	29349164	60680050	53711820	transcriptional regulator	K
Bd2207	Bv0556c	29349163	60680049	53711819	acetyl-coenzyme A synthetase	I
Bd2208	Bv0535c	29349127	60680021	53711790	glutamate 5-kinase	E
Bd2209	Bv0534c	29349126	60680020	53711789	gamma-glutamyl phosphate reductase	E
Bd2211	Bv0533c	29349125	60680017	53711785	ornithine carbamoyltransferase	E
Bd2212c	Bv0790c	29348141	60683443	53715471	conserved hypothetical protein	n/a
Bd2215c	Bv0792c	29348142	60683449	53715476	DNA-directed RNA polymerase beta' chain	K
Bd2216c	Bv0793c	29348143	60683450	53715477	DNA-directed RNA polymerase beta chain	K
Bd2217c	Bv0794c	29348144	60683451	53715478	50S ribosomal protein L7/L12	J
Bd2218c	Bv0795c	29348145	60683452	53715479	50S ribosomal protein L10	J
Bd2219c	Bv0796c	29348146	60683453	53715480	50S ribosomal protein L1	J
Bd2220c	Bv0797c	29348147	60683454	53715481	50S ribosomal protein L11	J
Bd2221c	Bv0798c	29348148	60683455	53715482	transcription anti-termination protein	K
Bd2223c	Bv0799c	29348149	60683457	53715484	elongation factor Tu	J
Bd2224c	Bv0800c	29348150	60683458	53715485	sigma-54 modulation protein	J
Bd2225c	Bv0801c	29348151	60683459	53715486	tyrosine type site-specific recombinase	L
Bd2230	Bv0803	29348152	60683461	53715488	putative aminopeptidase	E
Bd2231	Bv0804	29348153	60683462	53715489	acetyltransferase	R
Bd2234	Bv3146	29346571	60681548	53713299	putative aminopeptidase C	E
Bd2235c	Bv1441	29348156	60683463	53715490	glycosyltransferase family 30, related to KDO-transferases	M
Bd2236c	Bv1440	29348157	60683464	53715491	glutamyl-tRNA synthetase	J
Bd2237	Bv1439c	29348158	60683465	53715492	putative membrane-associated HD superfamily hydrolase	R
Bd2244c	Bv1438	29348159	60683466	53715493	putative protein-tyrosine-phosphatase	T
Bd2245c	Bv1436	29348161	60683468	53715495	putative primosomal protein N'	L
Bd2246c	Bv1435	29348162	60683469	53715496	conserved hypothetical protein	n/a
Bd2249c	Bv0302	29347500	60683043	53715084	methylmalonyl-CoA mutase large subunit	II
Bd2250c	Bv0301	29347501	60683044	53715085	putative methylmalonyl-CoA mutase small subunit	II
Bd2252	Bv3193c	29347229	60682685	53714674	conserved hypothetical protein	n/a
Bd2257c	Bv1946c	29349538	60680458	53712280	putative helicase	LR
Bd2274c	Bv3704c	82775345	60679697	53711380	two-component system response regulator	KT
Bd2277c	Bv2196c	29345653	60682453	53714435	putative 1-acyl-sn-glycerol-3-phosphate acyltransferase	I
Bd2284c	Bv0143c	29347849	60680086	53711858	glycoside hydrolase family 3, candidate beta-glycosidase	GV
Bd2287	Bv0146	29347899	60680082	53711854	50S ribosomal protein L19	J
Bd2299c	Bv0759c	29348109	60683410	53715438	50S ribosomal protein L17	J
Bd2300c	Bv0760c	29348110	60683411	53715439	DNA-directed RNA polymerase alpha chain	K
Bd2301c	Bv0761c	29348111	60683412	53715440	30S ribosomal protein S4	J

Bd2302c	Bv0762c	29348112	60683413	53715441	30S ribosomal protein S11	J
Bd2303c	Bv0763c	29348113	60683414	53715442	30S ribosomal protein S13	J
Bd2305c	Bv0764c	29348115	60683417	53715445	methionine aminopeptidase	J
Bd2306c	Bv0765c	29348116	60683418	53715446	preprotein translocase SecY subunit	U
Bd2307c	Bv0766c	29348117	60683419	53715447	50S ribosomal protein L15	J
Bd2308c	Bv0767c	29348118	60683420	53715448	50S ribosomal protein L30	J
Bd2309c	Bv0768c	29348119	60683421	53715449	30S ribosomal protein S5	J
Bd2310c	Bv0769c	29348120	60683422	53715450	50S ribosomal protein L18	J
Bd2311c	Bv0770c	29348121	60683423	53715451	50S ribosomal protein L6	J
Bd2312c	Bv0771c	29348122	60683424	53715452	30S ribosomal protein S8	J
Bd2313c	Bv0772c	29348123	60683425	53715453	chloroplast 30S ribosomal protein S14	J
Bd2314c	Bv0773c	29348124	60683426	53715454	50S ribosomal protein L5	J
Bd2315c	Bv0774c	29348125	60683427	53715455	50S ribosomal protein L24	J
Bd2316c	Bv0775c	29348126	60683428	53715456	50S ribosomal protein L14	J
Bd2317c	Bv0776c	29348127	60683429	53715457	30S ribosomal protein S17	J
Bd2318c	Bv0777c	29348128	60683430	53715458	50S ribosomal protein L29	n/a
Bd2319c	Bv0778c	29348129	60683431	53715459	50S ribosomal protein L16	J
Bd2320c	Bv0779c	29348130	60683432	53715460	30S ribosomal protein S3	J
Bd2321c	Bv0780c	29348131	60683433	53715461	50S ribosomal protein L22	J
Bd2322c	Bv0781c	29348132	60683434	53715462	30S ribosomal protein S19	J
Bd2323c	Bv0782c	29348133	60683435	53715463	50S ribosomal protein L2	J
Bd2324c	Bv0783c	29348134	60683436	53715464	50S ribosomal protein L23	J
Bd2325c	Bv0784c	29348135	60683437	53715465	50S ribosomal protein L4	J
Bd2326c	Bv0785c	29348136	60683438	53715466	50S ribosomal protein L3	J
Bd2327c	Bv0786c	29348137	60683439	53715467	30S ribosomal protein S10	J
Bd2328c	Bv0787c	29348138	60683440	53715468	elongation factor G	J
Bd2329c	Bv0788c	29348139	60683441	53715469	30S ribosomal protein S7	J
Bd2330c	Bv0789c	29348140	60683442	53715470	30S ribosomal protein S12	J
Bd2333c	Bv0409c	29349259	60683322	53715353	DNA mismatch repair protein mutL	L
Bd2335c	Bv0411c	29349257	60683324	53715355	conserved hypothetical protein	n/a
Bd2336c	Bv0412c	29349256	60683325	53715356	peptidyl-prolyl cis-trans isomerase	O
Bd2337c	Bv0413c	29349255	60683326	53715357	conserved hypothetical protein	n/a
Bd2339c	Bv0415c	29349253	60683328	53715359	inosine-5'-monophosphate dehydrogenase	FR
Bd2340c	Bv0429c	29349252	60683329	53715360	ATP-dependent DNA helicase recQ	L
Bd2341c	Bv0430c	29349251	60683330	53715361	ATP-dependent Clp protease ATP-binding subunit	O
Bd2342c	Bv0431c	29349250	60683331	53715362	ATP-dependent Clp protease proteolytic subunit 2	OU
Bd2343c	Bv0432c	29349249	60683332	53715363	FKBP-type peptidyl-prolyl cis-transisomerase	O
Bd2345c	Bv0434c	29349245	60683335	53715367	ABC transporter ATP-binding protein	R
Bd2347	Bv0435	29349246	60683336	53715368	conserved hypothetical protein	Q

Bd2348	Bv0436	29349247	60683337	53715369	ABC transporter ATP-binding protein	Q
Bd2349c	Bv1153	29348628	60679668	53711352	S-adenosylmethionine synthetase	H
Bd2350c	Bv1143	29348625	60679667	53711350	2-amino-4-hydroxy-6- hydroxymethyldihydropteridine	H
Bd2351c	Bv1142	29348623	60679666	53711349	S-adenosylmethionine:tRNA ribosyltransferase-isomerase	J
Bd2352c	Bv1141	29348622	60679665	53711348	tRNA pseudouridine synthase B	J
Bd2353c	Bv1140	29348621	60679664	53711347	putative undecaprenol kinase	V
Bd2354c	Bv1139	29348620	60679663	53711346	putative membrane protein	n/a
Bd2355c	Bv1138	29348619	60679662	53711345	putative cell division protein FtsX	D
Bd2361c	Bv1134	29348604	60679624	53711319	conserved hypothetical protein	J
Bd2363	Bv1132c	29348602	60679622	53711317	putative coenzyme A transferase	C
Bd2364	Bv3835c	29346733	60682243	53714048	putative ABC transporter permease protein	P
Bd2365	Bv3834c	29346732	60682242	53714047	putative ABC transporter permease protein	P
Bd2366	Bv3833c	29346731	60682241	53714046	putative phosphate transport ATP-binding protein	P
Bd2367	Bv3832c	29346730	60682240	53714045	putative transcriptional regulator	P
Bd2368	Bv2727	29348167	60683474	53715501	conserved hypothetical protein	n/a
Bd2370c	Bv1041c	29348795	60679783	53711520	ABC transporter ATP-binding protein	V
Bd2373	Bv2141c	29348160	60683467	53715494	conserved hypothetical protein	n/a
Bd2380c	Bv0437c	29349244	60683339	53715371	putative phosphoglycerate dehydrogenase	R
Bd2381c	Bv0438c	29349243	60683340	53715372	putative GTP-binding protein	R
Bd2382c	Bv0439c	29349242	60683341	53715373	3-oxoacyl-[acyl-carrier-protein] synthase III	I
Bd2383c	Bv0440c	29349240	60683342	53715375	conserved hypothetical protein	n/a
Bd2419	Bv1854	29349385	60680233	53712038	conserved hypothetical protein	n/a
Bd2420	Bv0016c	29347576	60683085	53715149	two-component system sensor histidine kinase	T
Bd2421	Bv0015c	29347575	60683084	53715148	two-component system response regulator	TK
Bd2422c	Bv0088c	29347152	60682631	53714609	carbon starvation protein A	T
Bd2423c	Bv1358c	29348852	60679842	53711592	conserved hypothetical protein	S
Bd2424c	Bv1359c	29348853	60679843	53711593	cell division protein FtsZ	D
Bd2425c	Bv1360c	29348854	60679844	53711594	cell division protein FtsA	D
Bd2426c	Bv1361c	29348855	60679845	53711595	putative cell division protein	n/a
Bd2427c	Bv1362c	29348856	60679846	53711596	UDP-N-acetylmuramate--alanine ligase	M
Bd2428c	Bv1363c	29348857	60679847	53711597	glycosyltransferase family 28, candidate undecaprenyldiphospho-muramoylpentapeptide beta-N-acetylglucosaminyltransferase	M
Bd2429c	Bv1364c	29348858	60679848	53711598	rod shape-determining protein rodA	D
Bd2430c	Bv1365c	29348859	60679849	53711599	UDP-N-acetylmuramoylalanine--D-glutamate ligase	M
Bd2431c	Bv1366c	29348860	60679850	53711600	phospho-N-acetylmuramoyl-pentapeptide-transferase	M
Bd2432c	Bv1367c	29348861	60679851	53711601	UDP-N-acetylmuramoylalanyl-D-glutamate--2, 6-diaminopimelate ligase	M
Bd2433c	Bv1368c	29348862	60679852	53711602	penicillin-binding protein	M
Bd2434c	Bv1369c	29348863	60679853	53711603	conserved hypothetical protein	n/a

Bd2435c	Bv1370c	29348864	60679854	53711604	S-adenosyl-methyltransferase MraW	M
Bd2436	Bv1372	29348867	60679862	53711612	conserved hypothetical protein	n/a
Bd2437	Bv1373	29348868	60679863	53711613	hemolysin A	n/a
Bd2441	Bv1855	29349386	60680234	53712039	putative cardiolipin synthetase	I
Bd2442c	Bv1856c	29349387	60680235	53712040	putative methyltransferase	L
Bd2443c	Bv1857c	29349388	60680236	53712041	conserved hypothetical protein	n/a
Bd2444c	Bv1858c	29349389	60680237	53712042	conserved hypothetical protein	n/a
Bd2445	Bv1859	29349390	60680238	53712043	putative ATP-dependent exodeoxyribonuclease	L
Bd2447	Bv1790	29347683	60680151	53711951	methyltransferase	R
Bd2448	Bv1791	29347682	60680152	53711952	conserved hypothetical protein	n/a
Bd2449	Bv1792	29347681	60680153	53711953	haloacid dehalogenase-like hydrolase	R
Bd2451c	Bv0074c	29347139	60680815	53712603	peptide chain release factor 3 (RF-3)	J
Bd2452c	Bv0075c	29347140	60680814	53712602	putative dTDP-4-dehydrorhamnose reductase	M
Bd2453c	Bv0076c	29347141	60680813	53712601	conserved hypothetical protein	n/a
Bd2462	Bv4033c	29345869	60681303	53713023	glycoside hydrolase family 20, candidate beta-N-acetylhexosaminidase	G
Bd2466	Bv0078	29347143	60682623	53714601	putative phosphoribosylformylglycinamidine synthase	FF
Bd2470	Bv0739c	29348957	60681379	53713113	putative ATPase	R
Bd2482c	Bv1852c	29349383	60680231	53712036	3-dehydroquinate synthase	E
Bd2483c	Bv1860c	29349403	60680248	53712054	alanyl-tRNA synthetase	J
Bd2484	Bv1861	29349404	60680249	53712055	putative peptidase	M
Bd2485	Bv1862	29349405	60680250	53712056	transcriptional regulator	K
Bd2486	Bv2822	29346775	60682328	53714272	conserved hypothetical protein	n/a
Bd2487	Bv1201	29348428	60683743	53715822	putative Na+/Ca2+ exchanger protein	P
Bd2490c	Bv1863c	29349406	60680251	53712057	GTP pyrophosphokinase	TK
Bd2492	Bv2645	29347797	60680858	53712699	O-acetylhomoserine (thiol)-lyase	E
Bd2496c	Bv1864c	29349407	60680252	53712058	glycoside hydrolase family 23, related to peptidoglycan lytic transglycosylases	M
Bd2497c	Bv1865c	29349408	60680253	53712059	conserved hypothetical protein	n/a
Bd2498c	Bv1866c	29349409	60680254	53712060	putative chromosome partitioning protein ParB	K
Bd2499c	Bv1867c	29349410	60680255	53712061	ParA family ATPase	D
Bd2503c	Bv1830c	29347648	60680194	53711993	putative biotin-(acetyl-CoA carboxylase) synthetase	H
Bd2504c	Bv1831c	29347647	60680195	53711994	conserved hypothetical protein	S
Bd2505c	Bv1832c	29347646	60680196	53711995	conserved hypothetical protein	L
Bd2507	Bv1834	29347644	60680198	53711997	putative cytosine/adenosine deaminase	FJ
Bd2509c	Bv1836c	29347642	60680200	53711999	CDP-diacylglycerol-serine O-phosphatidyltransferase	I
Bd2510c	Bv1837c	29347641	60680201	53712000	phosphatidylserine decarboxylase	I
Bd2511c	Bv3379	29346464	60681671	53713424	ATP-dependent helicase	L
Bd2512c	Bv1595c	29349287	60683228	53715293	putative translation initiation factor SUI1	J

Bd2514c	Bv1598	29349292	60683225	53715290	2-C-methyl-D-erythritol 2,4-cyclodiphosphate synthase	I
Bd2517c	Bv1597	29349291	60683226	53715291	2-hydroxyhepta-2,4-diene-1,7-dioate isomerase	Q
Bd2518c	Bv1596	29349290	60683227	53715292	putative DNA binding protein	R
Bd2522c	Bv1878	29349453	60680267	53712073	aminoacyl-histidine dipeptidase	E
Bd2523c	Bv1877	29349452	60680266	53712072	putative dolichol-P-glucose synthetase	n/a
Bd2524	Bv1876c	29349451	60680265	53712071	dimethyladenosine transferase	J
Bd2525	Bv1875c	29349450	60680264	53712070	putative Mg2+ transporter MgtE	P
Bd2527	Bv0503c	29349098	60679997	53711764	putative membrane protein	M
Bd2528	Bv0511c	29349117	60680009	53711777	elongation factor P	J
Bd2529	Bv0509c	29349103	60680007	53711774	putative DNA repair protein	L
Bd2530	Bv0508c	29349104	60680005	53711772	conserved hypothetical protein	R
Bd2531	Bv0507c	29349105	60680006	53711773	UDP-2,3-diacylglucosamine hydrolase	S
Bd2532c	Bv3836	29346734	60682244	53714049	phosphate ABC transporter, phosphate-binding protein	P
Bd2537c	Bv2805	29345930	60682160	53713956	transcriptional regulator	TK
Bd2538c	Bv2804	29345929	60682161	53713957	conserved hypothetical protein	n/a
Bd2549	Bv2909c	29350039	60681071	53712847	putative arylsulfatase precursor	P
Bd2550	Bv3692	29347328	60679677	53711361	choline-sulfatase	P
Bd2551	Bv0077c	29347142	60680812	53712600	conserved hypothetical protein	n/a
Bd2552	Bv2282	29348744	60679748	53711478	conserved hypothetical protein	n/a
Bd2554	Bv2511c	29347227	60682683	53714672	RNA polymerase ECF-type sigma factor	K
Bd2559	Bv2523c	29349753	60680506	53712335	30S ribosomal protein S1	J
Bd2561	Bv1922c	29349592	60680353	53712170	phosphoglycolate phosphatase	R
Bd2562	Bv1851c	29349382	60680226	53712031	putative transcriptional regulator	K
Bd2575	Bv1868	29349411	60680257	53712063	stationary-phase survival protein SurE	R
Bd2576	Bv1869	29349412	60680258	53712064	glycosyltransferase family 19, candidate lipid-alpha-disaccharide synthase	M
Bd2588	Bv2733c	29346620	60681426	53713165	cytochrome D ubiquinol oxidase subunit I	C
Bd2589	Bv2732c	29346619	60681427	53713166	cytochrome D ubiquinol oxidase subunit II	C
Bd2591	Bv2521c	29349754	60680507	53712336	conserved hypothetical protein	R
Bd2595c	Bv1343	29348836	60679837	53711586	conserved hypothetical protein	n/a
Bd2596c	Bv1342	29348828	60679834	53711583	2,3-bisphosphoglycerate-independent phosphoglycerate mutase	G
Bd2597	Bv1309	29348808	60679798	53711537	conserved hypothetical protein	R
Bd2600c	Bv1617c	29347388	60682940	53714974	Holliday junction DNA helicase ruvA	L
Bd2603c	Bv1716c	29349506	60680349	53712165	Trk system K+ uptake protein TrkA	P
Bd2604c	Bv1717c	29349507	60680350	53712166	1-deoxy-D-xylulose 5-phosphate synthase	HI
Bd2607c	Bv3240c	29348210	60683608	53715669	inositol-1-monophosphatase	G
Bd2609	Bv0366c	29349214	60683361	53715393	conserved hypothetical protein	n/a
Bd2610	Bv0365c	29349215	60683360	53715392	conserved hypothetical protein	n/a
Bd2614c	Bv0530	29349122	60680014	53711782	conserved hypothetical protein	n/a

Bd2615c	Bv0529	29349121	60680013	53711781	D-alanine--D-alanine ligase	M
Bd2616c	Bv0528	29349120	60680012	53711780	ribosomal large subunit pseudouridine synthase D	J
Bd2617c	Bv0527	29349119	60680011	53711779	conserved hypothetical protein	n/a
Bd2621c	Bv2013c	29349345	60683152	53715218	conserved hypothetical protein	R
Bd2622	Bv1924	29347108	60682292	53714229	conserved hypothetical protein	n/a
Bd2623	Bv1925	29347107	60682291	53714228	pyruvate carboxylase subunit B	CC
Bd2624	Bv1926	29347106	60682290	53714227	oxaloacetate decarboxylase beta chain	C
Bd2626	Bv3689c	29347155	60682634	53714613	conserved hypothetical protein	n/a
Bd2633c	Bv0446c	29349339	60683172	53715238	GTP cyclohydrolase I	H
Bd2634c	Bv0447c	29349338	60683173	53715239	conserved hypothetical protein	n/a
Bd2635c	Bv0448c	29349337	60683174	53715240	triosephosphate isomerase	G
Bd2636c	Bv0449c	29349336	60683175	53715241	conserved hypothetical protein	n/a
Bd2637c	Bv0450c	29349335	60683176	53715242	conserved hypothetical protein	n/a
Bd2638c	Bv0451c	29349334	60683177	53715243	putative peptidase	M
Bd2639c	Bv0453c	29349332	60683179	53715245	ATP-dependent DNA helicase RecG	LK
Bd2640c	Bv0454c	29349331	60683180	53715246	2-C-methyl-D-erythritol 4-phosphate cytidylyltransferase	I
Bd2641c	Bv0262c	29349299	60683218	53715282	conserved hypothetical protein	n/a
Bd2642c	Bv0263c	29349298	60683219	53715283	putative exodeoxyribonuclease VII large subunit	L
Bd2643c	Bv0264c	29349297	60683220	53715284	putative exported serine protease, subtilase family	O
Bd2644c	Bv1296c	29348387	60683722	53715795	transcriptional regulator	K
Bd2646c	Bv1393c	29348385	60683720	53715793	putative FkbP-type 22 kDa peptidyl-prolyl cis-trans isomerase	O
Bd2650	Bv1394	29348384	60683719	53715792	putative nicotinate mononucleotide:5,6-dimethylbenzimidazole phosphoribosyltransferase	K
Bd2657	Bv3145	29345568	60680862	53712703	Na+/H+ antiporter	C
Bd2658	Bv2728c	29346633	60681407	53713142	Na+/H+-dicarboxylate symporter	C
Bd2659c	Bv2962	29346841	60680667	53712466	putative exonuclease/DNA polymerase III epsilon c	L
Bd2665c	Bv3705c	29348666	60679696	53711379	putative two-component system sensor kinase	T
Bd2667c	Bv2974c	29346132	60681742	53713499	putative surface protein	S
Bd2671	Bv0607c	29349399	60680242	53712047	glycoside hydrolase family 92, related to an ill-defined alpha-1,2-mannosidase	G
Bd2672	Bv2276	29348819	60679820	53711561	conserved hypothetical protein	S
Bd2674	Bv1214	29350121	60682670	53714657	glycoside hydrolase family 29, related to alpha-L-fucosidases	G
Bd2687c	Bv1450	29348961	60679910	53711665	peptide chain release factor RF-2	J
Bd2688c	Bv1449	29348959	60679909	53711664	putative long-chain-fatty-acid--CoA ligase	I
Bd2689	Bv2700	29345992	60682109	53713900	conserved hypothetical protein	K
Bd2690	Bv2701	29345993	60682108	53713899	conserved hypothetical protein	n/a
Bd2691c	Bv1400	29347976	60683547	53715603	histidine triad (HIT) family protein	FGR
Bd2692c	Bv1399	29347975	60683548	53715604	transcription elongation factor GreA	K
Bd2693c	Bv1398	29347974	60683549	53715605	conserved hypothetical protein	n/a

Bd2694	Bv1397c	29347973	60683550	53715606	polyribonucleotide nucleotidyltransferase	J
Bd2699c	Bv1812	29347666	60680167	53711966	anaerobic fumarate hydratase class I	CC
Bd2701	Bv0850	29348253	60683695	53715764	putative plasminogen binding protein precursor	n/a
Bd2703c	Bv0945	29348196	60683676	53715742	GTP-binding elongation factor family protein TypA/BipA	T
Bd2704	Bv0867c	29348195	60683677	53715743	30S ribosomal protein S15	J
Bd2705	Bv2654c	29348192	60683678	53715744	transcriptional regulator	K
Bd2706	Bv2653c	29348191	60683679	53715745	putative long-chain-fatty-acid--CoA ligase	IQ
Bd2711c	Bv0864c	29348205	60683682	53715748	conserved hypothetical protein	n/a
Bd2712c	Bv0863	29348356	60683718	53715791	glycosyltransferase family 2, related to beta-glycosyltransferases	M
Bd2714c	Bv0862	29348355	60683717	53715790	glycosyltransferase family 4, related to alpha-glycosyltransferases	M
Bd2722c	Bv0854	29348343	60683710	53715783	putative lipopolysaccharide biosynthesis protein	R
Bd2723c	Bv2248c	29345732	60681144	53712931	putative GFO/IDH/MocA-family oxidoreductase	R
Bd2725c	Bv0853	29348342	60683709	53715782	Methionyl-tRNA synthetase	JR
Bd2737c	Bv0852	29348255	60683697	53715766	magnesium chelatase, subunit ChlI	O
Bd2739c	Bv0953c	29348199	60683662	53715725	phosphoenolpyruvate carboxykinase	C
Bd2740c	Bv0955c	29348246	60683658	53715720	2-oxoglutarate synthase subunit KorB	C
Bd2741c	Bv0956c	29348245	60683657	53715719	2-oxoglutarate synthase subunit korA	CC
Bd2743c	Bv1303	29348802	60679787	53711526	shikimate kinase	E
Bd2748	Bv1049c	29348800	60679785	53711522	putative ribonuclease H1	n/a
Bd2752c	Bv1034	29348773	60679778	53711515	conserved hypothetical protein	n/a
Bd2758	Bv2178c	29347080	60682574	53714553	putative transmembrane transport protein	GEPR
Bd2759	Bv1029c	29348770	60679776	53711513	erythronate-4-phosphate dehydrogenase	HE
Bd2760c	Bv1021	29348769	60679775	53711512	phosphoribosylglycinamide formyltransferase	F
Bd2761	Bv1019c	29348767	60679773	53711510	3-oxoacyl-[acyl-carrier-protein] synthase II	IQ
Bd2762	Bv1018c	29348766	60679772	53711509	putative ribonuclease III	K
Bd2764c	Bv1009	29348765	60679771	53711508	6-phosphofructokinase	G
Bd2765	Bv1008c	29348764	60679770	53711507	putative auxin-regulated protein	n/a
Bd2768c	Bv3097	29346265	60681929	53713655	two-component system sensor histidine kinase	TT
Bd2771	Bv0978	29348760	60679757	53711487	cysteinyl-tRNA synthetase	J
Bd2772	Bv0322c	29348752	60679756	53711486	putative phenylacetic acid degradation protein	n/a
Bd2773c	Bv1006c	29348751	60679755	53711485	conserved hypothetical protein	n/a
Bd2774c	Bv1007c	29348750	60679754	53711484	conserved hypothetical protein	n/a
Bd2780c	Bv1224c	29348730	60679744	53711474	conserved hypothetical protein	n/a
Bd2781	Bv1225	29348729	60679743	53711473	dihydrodipicolinate reductase	E
Bd2782	Bv1226	29348728	60679742	53711472	signal peptidase I	U
Bd2784	Bv1228	29348726	60679740	53711470	conserved hypothetical protein	n/a
Bd2789c	Bv1774c	29347430	60682966	53715000	conserved hypothetical protein	n/a
Bd2800	Bv2609	29346578	60682256	53714062	N-acetylmuramoyl-L-alanine amidase	n/a

Bd2810	Bv0201c	29349047	60679971	53711737	ThiF family protein, putative dinucleotide-utilizing enzyme involved in molybdopterin and thiamine biosynthesis	H
Bd2811	Bv0200c	29349048	60679972	53711738	lipoprotein releasing system ATP-binding protein	V
Bd2819	Bv1455	29349050	60679974	53711740	Na+-dependent transporter	R
Bd2822c	Bv1162c	29345995	60679612	53711307	conserved hypothetical protein	n/a
Bd2824	Bv2031c	29349884	60682843	53714861	amino acid carrier protein	E
Bd2826c	Bv1237c	29348717	60679735	53711465	putative zinc protease	R
Bd2829	Bv1239	29348715	60679733	53711463	putative sugar isomerase	M
Bd2830	Bv1240	29348714	60679732	53711462	fructokinase	G
Bd2835	Bv1261c	29348712	60679730	53711460	ATP-dependent RNA helicase	LKJ
Bd2842c	Bv1260	29348698	60679727	53711457	conserved hypothetical protein	n/a
Bd2843c	Bv1259	29348697	60679726	53711456	putative integral membrane protein	S
Bd2845c	Bv1258	29348696	60679725	53711455	polysaccharide biosynthesis protein	R
Bd2846c	Bv1257	29348694	60679724	53711454	Holliday junction DNA helicase RuvB	L
Bd2869	Bv4011c	29345843	60681205	53712994	putative xylose repressor	KG
Bd2876	Bv4037c	29345866	60681299	53713019	glycoside hydrolase family 20, related to beta-N-acetylhexosaminidases	G
Bd2877	Bv4035c	29345867	60681301	53713021	sialate O-acetylesterase	n/a
Bd2885c	Bv1154	29348633	60679669	53711353	putative lysine decarboxylase	R
Bd2886c	Bv4017c	29347309	60681996	53713727	conserved hypothetical protein	n/a
Bd2889	Bv1186c	29348693	60679721	53711452	conserved hypothetical protein	RS
Bd2890	Bv1185c	29348692	60679714	53711447	conserved hypothetical protein with conserved domain	R
Bd2894	Bv1182c	29348677	60679709	53711391	glucose-inhibited division protein A	D
Bd2896	Bv1180c	29348675	60679707	53711389	excinuclease ABC subunit C	L
Bd2897	Bv1179c	29348674	60679706	53711388	putative D-tyrosyl-tRNA deacylase	J
Bd2898	Bv1178c	29348673	60679705	53711387	conserved hypothetical protein	R
Bd2899	Bv1177c	29348672	60679704	53711386	putative deoxyribose-phosphate aldolase	F
Bd2900c	Bv1176	29348670	60679701	53711383	octaprenyl-diphosphate synthase	H
Bd2901	Bv1175c	29348669	60679700	53711382	DNA polymerase I	LL
Bd2916	Bv1173c	29348525	60683776	53715858	GTP-binding protein	J
Bd2927c	Bv1163	29348530	60683780	53715862	putative DNA mismatch repair protein MutS	L
Bd2928c	Bv3248	29347068	60682551	53714529	transaldolase	G
Bd2937	Bv1118c	29348535	60683783	53715865	leucyl-tRNA synthetase	J
Bd2939	Bv1115c	29348537	60683785	53715867	putative xanthosine triphosphate pyrophosphatase	F
Bd2943c	Bv1114	29348573	60683786	53715869	quinolinate synthetase A	H
Bd2944	Bv1112c	29348574	60679598	53711292	tRNA/rRNA methyltransferase	J
Bd2945c	Bv1111	29348575	60679609	53711304	conserved hypothetical protein	n/a
Bd2948	Bv1110c	29348576	60679610	53711305	conserved hypothetical protein	n/a
Bd2949	Bv1109c	29348577	60679611	53711306	putative Fe-S oxidoreductase	C

Bd2952c	Bv2153	29349027	60679957	53711719	putative transmembrane protein	n/a
Bd2961	Bv1106c	29348591	60679617	53711312	rubrerythrin	C
Bd2962c	Bv1082	29348593	60679618	53711313	putative exported L-aspartate oxidase	H
Bd2982	Bv1155	29348634	60679670	53711354	conserved hypothetical protein	n/a
Bd2983	Bv1156	29348635	60679671	53711355	possible uroporphyrinogen-III synthase	n/a
Bd2984	Bv1157	29348636	60679672	53711356	putative ribonuclease P protein component	n/a
Bd2985	Bv1158	29348637	60679673	53711357	conserved hypothetical protein	S
Bd2986	Bv1159	29348638	60679674	53711358	TatD-related DNase	L
Bd2987	Bv1160	29348639	60679675	53711359	putative tyrosyl-tRNA synthetase 1	J
Bd3001	Bv3777c	29349650	60680403	53712223	putative transporter	S
Bd3002c	Bv3533	29349728	60680484	53712310	putative zinc protease	R
Bd3005	Bv1072	29348656	60679683	53711367	conserved hypothetical protein	n/a
Bd3006	Bv1073	29348655	60679682	53711366	polysaccharide deacetylase	G
Bd3008	Bv1431c	29347846	60680092	53711864	putative secreted tripeptidyl aminopeptidase	n/a
Bd3009	Bv1070	29348658	60679688	53711371	putative metal ABC transporter substrate-binding precursor	P
Bd3010	Bv1071	29348657	60679687	53711370	putative metal ABC transporter, ATP-binding protein	P
Bd3011	Bv1058c	29348484	60683766	53715847	NAD-dependent epimerase	MG
Bd3020	Bv1061	29348665	60679695	53711378	serine acetyltransferase	E
Bd3021	Bv1062	29348664	60679694	53711377	putative N6-adenine-specific DNA methylase	L
Bd3022	Bv1063	29348663	60679693	53711376	dipeptidyl peptidase IV	E
Bd3023	Bv1064	29348662	60679692	53711375	phosphoribosylamine--glycine ligase	F
Bd3024	Bv1065	29348661	60679691	53711374	conserved hypothetical protein	n/a
Bd3025	Bv1066	29348660	60679690	53711373	putative integral membrane protein	S
Bd3027c	Bv1448c	29348958	60679908	53711663	putative peptidase/deacetylase	E
Bd3028c	Bv1268c	29347953	60683569	53715626	putative riboflavin kinase	H
Bd3073	Bv2424	29345562	60680855	53712696	acetyl esterase	R
Bd3074	Bv1846c	29347596	60680225	53712030	3-phosphoshikimate 1-carboxyvinyltransferase	E
Bd3075	Bv1845c	29347615	60680215	53712015	conserved hypothetical protein	n/a
Bd3076	Bv1844c	29347616	60680214	53712014	zinc ABC transporter permease	P
Bd3078c	Bv3194	29346048	60682055	53713842	putative methionine aminopeptidase A	J
Bd3079c	Bv2012c	29346881	60681100	53712882	putative transmembrane protein	R
Bd3082c	Bv1389	29347938	60683573	53715631	putative Na+-dependent phosphate transporter	P
Bd3083	Bv1388c	29347937	60683574	53715632	uridine kinase (uridine monophosphokinase)	FJ
Bd3097	Bv1385c	29347931	60683581	53715641	RNA polymerase sigma-54	K
Bd3098	Bv1384c	29347930	60683582	53715642	conserved hypothetical protein	n/a
Bd3099	Bv1383c	29347929	60683583	53715643	putative glycine cleavage system H protein	E
Bd3100	Bv1382c	29347928	60683584	53715644	phosphoribosylaminoimidazole carboxylase	F
Bd3101	Bv1381c	29347927	60683585	53715645	1-hydroxy-2-methyl-2-(E)-butenyl 4-diphosphate synthase	I
Bd3102	Bv1375	29348870	60679865	53711615	deoxyuridine 5'-triphosphate nucleotidohydrolase	F

Bd3103	Bv1376	29348871	60679866	53711616	TPR domain protein	R
Bd3105	Bv1378	29348873	60679868	53711618	putative peptidase	n/a
Bd3112c	Bv2996c	29346233	60681862	53713582	uronate isomerase	G
Bd3113c	Bv2981	29345899	60682191	53713991	putative gluconate aldolase	G
Bd3116c	Bv1054	29348482	60683764	53715845	putative TIM-barrel enzyme, possible dehydrogenase, contains a highly conserved dihydrouridine synthase domain	J
Bd3119	Bv1053c	29348481	60683762	53715843	collagenase precursor	O
Bd3120	Bv1216c	29348480	60683761	53715842	conserved hypothetical protein	R
Bd3121	Bv1215c	29348479	60683760	53715841	putative DNA processing Smf-like protein	LU
Bd3122c	Bv0208	29348986	60679939	53711700	conserved hypothetical protein	n/a
Bd3123c	Bv0207	29348987	60679940	53711701	conserved hypothetical protein	n/a
Bd3124c	Bv0206	29348988	60679941	53711702	topoisomerase IV subunit A	L
Bd3128	Bv0205c	29349019	60679942	53711703	glycyl-tRNA synthetase	J
Bd3135c	Bv1209	29348464	60683752	53715832	fumarate reductase iron-sulfur cluster protein subunit	C
Bd3136c	Bv1208	29348463	60683751	53715831	fumarate reductase flavoprotein subunit	C
Bd3137c	Bv1207	29348462	60683750	53715830	putative cytochrome B subunit	n/a
Bd3141	Bv0256	29349307	60683211	53715275	transcriptional regulator	n/a
Bd3145	Bv2961c	29345713	60682418	53714398	putative patatin-like phospholipase	R
Bd3146	Bv3239c	29346179	60681815	53713531	conserved hypothetical protein	S
Bd3193c	Bv2379c	29350072	60680717	53712534	tryptophan synthase beta chain	R
Bd3195	Bv2150c	29348734	60679747	53711477	conserved hypothetical outer membrane protein	n/a
Bd3196c	Bv0203	29349044	60679969	53711735	aspartate-semialdehyde dehydrogenase	E
Bd3203	Bv1984	29346416	60682652	53714637	putative nitroreductase	C
Bd3209c	Bv0082c	29347147	60682627	53714605	putative secreted protein	S
Bd3210	Bv0083	29347149	60682628	53714606	UvrABC SOS-repair system protein excinuclease A	L
Bd3215	Bv1809	29347668	60680164	53711962	putative GTP-binding protein	R
Bd3216	Bv1810	29347667	60680165	53711963	conserved hypothetical protein	n/a
Bd3227c	Bv0849	29348252	60683694	53715763	integrase	L
Bd3228	Bv0848c	29348251	60683693	53715762	putative type II 3-dehydroquinate dehydratase	E
Bd3229	Bv0847c	29348250	60683692	53715761	pyruvate kinase	G
Bd3230	Bv0846c	29348249	60683691	53715760	O-methyltransferase	R
Bd3231	Bv0845c	29348248	60683689	53715758	ribosome-binding factor A	J
Bd3232	Bv0844c	29348247	60683688	53715757	conserved hypothetical protein	M
Bd3233c	Bv1401c	29348190	60683546	53715602	putative mannose-1-phosphate guanylyltransferase	M
Bd3240	Bv2421	29347777	60679901	53711655	acyltransferase	I
Bd3242	Bv2668c	29346674	60682196	53713997	two-component system sensor histidine kinase, with response regulator receiver domain	TT
Bd3243c	Bv3179	29346112	60681967	53713696	chloramphenicol acetyltransferase (CAT-III)	V
Bd3244c	Bv1424c	29347820	60682638	53714619	putative acetyltransferase	KR

Bd3246	Bv0737c	29346957	60682172	53713969	conserved hypothetical protein	S
Bd3252c	Bv1434	29348168	60683505	53715558	conserved hypothetical protein	n/a
Bd3253c	Bv1433	29348169	60683506	53715559	conserved hypothetical protein	S
Bd3254c	Bv1432	29348170	60683507	53715560	conserved hypothetical protein	n/a
Bd3255c	Bv1815c	29347663	60680171	53711971	putative outer membrane protein TolC	MU
Bd3256c	Bv1816c	29347662	60680172	53711972	multidrug efflux membrane fusion protein	V
Bd3257c	Bv1817c	29347661	60680173	53711973	putative membrane fusion protein precursor	M
Bd3258c	Bv3795c	29349820	60680584	53712425	conserved hypothetical protein	n/a
Bd3259	Bv2677c	29346095	60681681	53713434	putative Na+/H+ exchanger	P
Bd3265c	Bv1734	29347872	60680072	53711845	putative anti-sigma factor	n/a
Bd3266c	Bv1733	29347873	60680073	53711846	putative ECF-type RNA polymerase sigma factor	n/a
Bd3269	Bv1843c	29347617	60680213	53712013	putative ATPase/GTPase	R
Bd3270c	Bv1060	29348486	60683768	53715849	ribonuclease R	K
Bd3272	Bv1202	29348441	60683744	53715823	conserved hypothetical protein	n/a
Bd3273	Bv1203	29348442	60683745	53715824	DNA topoisomerase IV subunit B	L
Bd3274	Bv1204	29348443	60683746	53715825	phosphopantetheine adenylyltransferase	H
Bd3275	Bv1205	29348444	60683747	53715826	carboxy-terminal processing protease precursor	M
Bd3278	Bv2734	29346622	60681425	53713163	outer membrane efflux protein	MU
Bd3279	Bv2735	29346623	60681424	53713162	periplasmic component of efflux system	M
Bd3280	Bv2736	29346624	60681423	53713161	putative ABC transporter ATP-binding protein	V
Bd3281	Bv2737	29346625	60681422	53713160	ABC transporter permease protein	V
Bd3282	Bv2738	29346626	60681421	53713159	putative two-component system sensor histidine kinase	T
Bd3283	Bv2739	29346627	60681420	53713158	two-component system response regulator	KT
Bd3284c	Bv1077c	29348596	60679621	53711316	putative exported D-alanyl-D-alanine carboxypeptidase penicillin-binding protein	M
Bd3285	Bv1788c	29347686	60680148	53711948	putative permease	R
Bd3291c	Bv0498c	29349052	60679976	53711742	UDP-N-acetylmuramoylalanyl-D-glutamyl-2, 6-diaminopimelate-D-alanyl-D-alanyl ligase	M
Bd3292	Bv0499	29349054	60679981	53711748	dihydropteroate synthase	H
Bd3293	Bv0500	29349055	60679982	53711749	conserved hypothetical protein	S
Bd3294	Bv0505	29349100	60680002	53711769	phosphate acetyltransferase	C
Bd3295	Bv0506	29349101	60680003	53711770	acetate kinase	C
Bd3296	Bv1826c	29347652	60680189	53711986	uridylate kinase	F
Bd3300	Bv1623	29347168	60682645	53714627	putative sugar transport-related, membrane protein	G
Bd3301	Bv1820c	29347659	60680182	53711978	ribosome recycling factor	J
Bd3302	Bv1819c	29347660	60680181	53711977	putative GTPase	R
Bd3303c	Bv1374c	29348869	60679864	53711614	dGTP triphosphohydrolase	F
Bd3307c	Bv1945c	29349536	60680456	53712278	conserved hypothetical protein	J
Bd3308c	Bv1936	29349596	60680444	53712264	enoyl-[acyl-carrier-protein] reductase	I

Bd3309c	Bv1719	29349509	60680368	53712185	putative alanine racemase and UDP-N-acetylmuramoylalanyl-D-glutamyl, 6-diaminopimelate-D-alanyl-D-alanyl ligase fusion protein	MM
Bd3310c	Bv1718	29349508	60680369	53712186	conserved hypothetical protein	n/a
Bd3317	Bv2810c	29350146	60680841	53712630	formate acetyltransferase	C
Bd3318	Bv2809c	29350145	60680840	53712629	putative pyruvate formate-lyase 1 activating enzyme	O
Bd3320	Bv2577c	29349980	60680680	53712480	enolase	G
Bd3321	Bv0820c	29348221	60680733	53712551	alkyl hydroperoxide reductase subunit C	O
Bd3322	Bv0819c	29348220	60680732	53712550	alkyl hydroperoxide reductase subunit F	O
Bd3326	Bv0824c	29347475	60683755	53715835	putative TonB-linked outer membrane receptor	H
Bd3331c	Bv1612c	29347409	60682945	53714978	anaerobic ribonucleoside-triphosphate reductase activating protein	O
Bd3332c	Bv1613c	29347408	60682944	53714977	anaerobic ribonucleoside-triphosphate reductase	F
Bd3342	Bv2002c	29346899	60681082	53712861	outer membrane vitamin B12 receptor protein	n/a
Bd3350c	Bv0397	29349274	60683252	53715319	glutamine-dependent carbamyl phosphate synthetase	EF
Bd3358c	Bv0058c	29346082	60682454	53714436	drug efflux protein	V
Bd3359c	Bv0059c	29346081	60682455	53714437	putative outer membrane protein TolC	n/a
Bd3360c	Bv0060c	29346080	60682456	53714438	putative cation efflux system	V
Bd3361c	Bv0061c	29346079	60682457	53714439	putative cation efflux system protein	M
Bd3362c	Bv0062c	29346072	60682458	53714440	putative membrane protein	n/a
Bd3375c	Bv3162	29347658	60682395	53714374	putative integral membrane protein	GER
Bd3377	Bv0395c	29349272	60683253	53715320	tryptophanyl-tRNA synthetase	J
Bd3401	Bv2830c	29346954	60680931	53712782	NADH pyrophosphatase	L
Bd3403	Bv3096	29346266	60681930	53713656	putative two-component system response regulator	T
Bd3407	Bv1067	29348659	60679689	53711372	conserved hypothetical protein	S
Bd3408c	Bv1839	29347639	60680203	53712002	thioredoxin (thioredoxin M)	OC
Bd3409c	Bv1838	29347640	60680202	53712001	DNA polymerase III alpha subunit	L
Bd3412	Bv1591	29349283	60683232	53715297	50S ribosomal protein L13	J
Bd3413	Bv1592	29349284	60683231	53715296	30S ribosomal protein S9	J
Bd3414	Bv1593	29349285	60683230	53715295	30S ribosomal protein S2 (BS1)	J
Bd3415	Bv1594	29349286	60683229	53715294	elongation factor Ts (EF-Ts)	J
Bd3416c	Bv0142	29347825	60680114	53711886	aspartate aminotransferase	E
Bd3417c	Bv0141	29347826	60680113	53711885	GTP cyclohydrolase II	HH
Bd3418c	Bv0140	29347827	60680112	53711884	conserved hypothetical protein	n/a
Bd3419c	Bv0139	29347828	60680111	53711883	conserved hypothetical protein	n/a
Bd3453c	Bv3540c	29347807	60680131	53711904	conserved hypothetical protein	n/a
Bd3454c	Bv3541c	29347806	60680132	53711905	putative membrane transporter involved in nicotinamide mononucleotide transport	n/a
Bd3456	Bv1320	29348811	60679811	53711552	conserved hypothetical protein	S
Bd3457	Bv1321	29348812	60679812	53711553	putative nitrogen utilization substance protein	K

Bd3458	Bv1322	29348813	60679813	53711554	translation initiation factor IF-2	J
Bd3460	Bv1324	29348815	60679815	53711556	ABC transporter permease	O
Bd3461	Bv1325	29348816	60679817	53711558	ABC transporter ATP-binding protein	O
Bd3462	Bv1326	29348817	60679818	53711559	conserved hypothetical protein, putative ABC transporter permease component	O
Bd3464c	Bv1349c	29349112	60682603	53714581	glycoside hydrolase family 13, candidate alpha-glycosidase	G
Bd3467	Bv1327	29348818	60679819	53711560	aminotransferase	E
Bd3468	Bv1454c	29349051	60679975	53711741	conserved hypothetical protein	n/a
Bd3469	Bv1338	29348821	60679832	53711581	ribonuclease HII	L
Bd3470c	Bv1283c	29347957	60683563	53715621	conserved hypothetical protein	R
Bd3471c	Bv1284c	29347958	60683562	53715620	leucine aminopeptidase precursor	R
Bd3474c	Bv1355c	29348838	60679839	53711588	DNA gyrase B subunit	L
Bd3475c	Bv1356c	29348839	60679840	53711589	ribosomal protein S20	n/a
Bd3476c	Bv2440	29346782	60682335	53714279	conserved hypothetical protein	n/a
Bd3477	Bv1357	29348840	60679841	53711591	DNA repair protein	n/a
Bd3486	Bv1618	29347389	60682941	53714975	putative oxidoreductase	n/a
Bd3488	Bv1943	29349534	60680453	53712275	flavoprotein	C
Bd3489	Bv4015	29349535	60680454	53712276	glucosamine-6-phosphate isomerase	G
Bd3493c	Bv0194	29347908	60680076	53711848	putative outer membrane efflux protein	MU
Bd3494c	Bv0193	29347907	60680077	53711849	membrane fusion efflux protein	M
Bd3495c	Bv0192	29347906	60680078	53711850	putative ABC transporter ATP-binding protein	V
Bd3497c	Bv0191	29347905	60680079	53711851	ABC transporter putative permease	V
Bd3498c	Bv0190	29347904	60680080	53711852	ABC transporter ATP-binding protein	V
Bd3499	Bv2702	29346114	60681705	53713459	phosphoribosylglycinamide formyltransferase 2	F
Bd3508	Bv2863	29346988	60680898	53712745	putative metal dependent hydrolase	n/a
Bd3509	Bv2864	29346990	60680896	53712743	lactoylglutathione lyase	E
Bd3510	Bv0189c	29347903	60680081	53711853	ROK family transcriptional repressor, with glucokinase domain	KG
Bd3511	Bv1299	29348224	60683634	53715696	putative mutT family protein	LF
Bd3512	Bv1300	29348225	60683635	53715697	conserved hypothetical protein	n/a
Bd3513	Bv1301	29348226	60683636	53715698	putative TonB-dependent receptor	n/a
Bd3519c	Bv2256c	29345744	60681152	53712940	conserved hypothetical protein	n/a
Bd3520	Bv1302	29348236	60683637	53715699	DNA topoisomerase I	RL
Bd3522c	Bv1050c	29348238	60683639	53715701	arginyl-tRNA synthetase	J
Bd3523c	Bv0965	29348239	60683640	53715702	DNA-binding protein HU-beta	L
Bd3524	Bv0964c	29348240	60683641	53715703	putative transmembrane rhomboid family protein	R
Bd3525	Bv0963c	29348241	60683642	53715704	conserved hypothetical protein	R
Bd3526	Bv0962c	29348242	60683643	53715705	conserved hypothetical protein	n/a
Bd3529	Bv0960c	29348244	60683645	53715707	protein-export membrane protein SecD/SecF	UU
Bd3565	Bv0325	29348103	60683405	53715432	putative urocanate hydratase	E

Bd3566	Bv0326	29348102	60683404	53715431	formiminotransferase-cyclodeaminase	E
Bd3567	Bv0328	29348101	60683403	53715430	imidazolonepropionase	Q
Bd3568	Bv0329	29348100	60683402	53715429	putative methenyltetrahydrofolate cyclohydrolase	E
Bd3569	Bv0330	29348099	60683401	53715428	histidine ammonia-lyase	E
Bd3570	Bv3355	29347665	60680168	53711967	conserved hypothetical protein	n/a
Bd3571c	Bv0258c	29349303	60683214	53715278	Mrp/Nbp35 family ATP-binding protein	D
Bd3572c	Bv0259c	29349302	60683215	53715279	putative methyltransferase	R
Bd3574	Bv0811c	29348208	60683594	53715655	putative PfkB family carbohydrate kinase	G
Bd3575	Bv0810c	29348207	60683593	53715654	carboxy-terminal processing protease precursor	M
Bd3586	Bv1452	29348974	60679915	53711669	putative TonB-dependent receptor	n/a
Bd3589	Bv2828c	29346638	60681374	53713108	fructose-1,6-bisphosphatase	G
Bd3600	Bv3544	29349718	60680475	53712297	putative NADPH-dependent glutamate synthase	ERHC
Bd3604c	Bv0829c	29349141	60680034	53711803	argininosuccinate lyase	E
Bd3605c	Bv0830c	29349140	60680033	53711802	conserved hypothetical protein	n/a
Bd3606c	Bv0831c	29349139	60680032	53711801	orotate phosphoribosyltransferase	F
Bd3607c	Bv0832c	29348209	60683607	53715668	putative amidophosphoribosyl-transferase	R
Bd3608c	Bv0833c	29349138	60680031	53711800	putative regulatory protein	n/a
Bd3609c	Bv0834c	29349137	60680030	53711799	putative protoporphyrinogen oxidase	J
Bd3610	Bv0835	29349136	60680029	53711798	riboflavin biosynthesis protein ribD	HH
Bd3611	Bv0836	29349135	60680028	53711797	conserved hypothetical protein	n/a
Bd3613	Bv0837	29349134	60680027	53711796	undecaprenyl pyrophosphate synthetase	I
Bd3615	Bv0840	29349132	60680025	53711794	cationic outer membrane protein precursor	n/a
Bd3616	Bv0841	29349131	60680024	53711793	putative outer membrane protein OmpH	n/a
Bd3617	Bv0842	29349130	60680023	53711792	glutamate racemase	M
Bd3618c	Bv2229	29350016	60681994	53713724	conserved hypothetical protein	S
Bd3624c	Bv4045	29347545	60679965	53711729	conserved hypothetical protein	S
Bd3629	Bv4044c	29347544	60683060	53715102	conserved hypothetical protein	R
Bd3630	Bv4043c	29347543	60683059	53715101	conserved hypothetical exported protein	n/a
Bd3631c	Bv4042	29347540	60683058	53715100	uracil-DNA glycosylase	L
Bd3632c	Bv4041	29347539	60683057	53715099	aspartate-ammonia ligase	E
Bd3635	Bv1920c	29346226	60681856	53713573	conserved hypothetical protein	S
Bd3636	Bv1585	29349279	60683237	53715302	adenylosuccinate lyase	F
Bd3637	Bv1586	29349280	60683236	53715301	ribosomal large subunit pseudouridine synthase B	J
Bd3638	Bv1587	29349281	60683235	53715300	asparaginyl-tRNA synthetase	J
Bd3639c	Bv1898	29347459	60682983	53715020	transcriptional regulator	K
Bd3640	Bv1896c	29347461	60679737	53711467	conserved hypothetical protein	n/a
Bd3641c	Bv1895	29347462	60682985	53715023	conserved hypothetical protein	n/a
Bd3642c	Bv1894	29347463	60682986	53715024	conserved hypothetical protein	n/a
Bd3643c	Bv1893	29347464	60682987	53715025	conserved hypothetical protein	n/a

Bd3644c	Bv1892	29347465	60682988	53715026	putative biopolymer transport protein	U
Bd3645c	Bv1891	29347466	60682989	53715027	conserved hypothetical protein	L
Bd3646c	Bv1890	29347468	60682991	53715029	putative isoprenoid biosynthesis related protein	H
Bd3647c	Bv1889	29347469	60682992	53715030	outer membrane protein TonB	n/a
Bd3649	Bv1888c	29347470	60682993	53715031	cytidylate kinase	F
Bd3650	Bv1887c	29347471	60682994	53715032	penicillin tolerance protein LytB	IM
Bd3651	Bv1886c	29347472	60682995	53715033	6-phosphofructokinase	G
Bd3654c	Bv1885	29349457	60680277	53712082	putative ArgK protein	E
Bd3655c	Bv1884	29349456	60680276	53712081	conserved hypothetical protein	n/a
Bd3657c	Bv1882	29349455	60680272	53712079	conserved hypothetical protein	R
Bd3658c	Bv1899c	29347458	60682982	53715019	dihydrofolate reductase	H
Bd3659c	Bv1900c	29347457	60682981	53715018	putative thymidylate synthase B	F
Bd3669c	Bv0324	29349381	60683103	53715168	S-adenosylmethionine:tRNA ribosyltransferase-isomerase	J
Bd3670	Bv0323c	29347589	60683102	53715167	putative DNA mismatch repair protein	n/a
Bd3671	Bv1451	29348963	60679913	53711667	conserved hypothetical protein	S
Bd3672	Bv2193c	29345646	60682481	53714459	conserved hypothetical protein	n/a
Bd3674c	Bv0018	29347578	60683089	53715153	coproporphyrinogen III oxidase	H
Bd3675	Bv0017c	29347577	60683086	53715151	putative elongation factor G	J
Bd3677	Bv2915	29345645	60682483	53714460	putative glycosyltransferase	n/a
Bd3678c	Bv0750	29347333	60682816	53714830	O-acetylhomoserine (thiol)-lyase	E
Bd3679c	Bv3257	29346603	60681449	53713188	conserved hypothetical protein	V
Bd3682c	Bv2220c	29348820	60679829	53711580	pyrophosphate-energized vacuolar membrane proton pump	C
Bd3683	Bv3886	29346184	60681819	53713535	conserved hypothetical protein	R
Bd3684	Bv3887	29346183	60681817	53713533	glycoside hydrolase family 13, candidate alpha-glycosidase	G
Bd3687	Bv3460	29346158	60681758	53713520	ribose-phosphate pyrophosphokinase	FE
Bd3688c	Bv0393	29345565	60680859	53712700	putative tRNA (5-methylaminomethyl-2-thiouridylate)-methyltransferase	J
Bd3689	Bv1960	29347256	60682716	53714710	putative dipeptidyl-peptidase III	n/a
Bd3690	Bv1961	29347255	60682715	53714709	conserved hypothetical protein	n/a
Bd3691	Bv1962	29347254	60682713	53714707	putative iron uptake regulation protein	P
Bd3692	Bv1963	29347253	60682712	53714706	adenylosuccinate synthetase	F
Bd3693	Bv0362c	29347251	60682710	53714704	conserved hypothetical protein	R
Bd3694	Bv0342c	29347250	60682707	53714701	histidyl-tRNA synthetase	J
Bd3696	Bv0337c	29347240	60682692	53714682	10 kDa chaperonin (groES)	O
Bd3697	Bv0336c	29347239	60682691	53714681	60 kDa chaperonin (groEL)	O
Bd3698	Bv2520c	29349755	60680508	53712337	RNA polymerase ECF-type sigma factor	K
Bd3700	Bv2518c	29349757	60680510	53712339	conserved hypothetical protein	n/a
Bd3701c	Bv2514	29349758	60680511	53712340	conserved hypothetical protein	R
Bd3702c	Bv2036c	29345596	60682503	53714480	putative exported cytochrome C biogenesis-related protein	OC

Bd3703c	Bv3271c	29346052	60682049	53713831	putative RNA pseudouridylate synthase	J
Bd3704	Bv2513c	29349761	60680513	53712342	valyl-tRNA synthetase	J
Bd3707	Bv2508	29349769	60680528	53712359	conserved hypothetical protein	n/a
Bd3709	Bv2957	29347075	60682571	53714549	conserved hypothetical protein	n/a
Bd3710	Bv2958	29347074	60682570	53714548	crossover junction endodeoxyribonuclease RuvC	L
Bd3711c	Bv0243	29347383	60682898	53714920	NAD-specific glutamate dehydrogenase 1	E
Bd3712	Bv0242c	29347382	60682897	53714918	phosphoenolpyruvate synthase/pyruvate phosphate dikinase	n/a
Bd3716c	Bv0238	29347380	60682871	53714892	NADP-specific glutamate dehydrogenase	E
Bd3747c	Bv1990c	29347819	60680120	53711892	putative TonB-dependent outer membrane protein	n/a
Bd3748c	Bv1991c	29347818	60680121	53711893	transcriptional regulator	TK
Bd3760c	Bv1108	29348590	60679616	53711311	putative sulfate transporter, permease	P
Bd3763c	Bv3188	29347161	60682641	53714622	glycine betaine transport ATP-binding protein	E
Bd3811c	Bv3191	29348532	60681450	53713189	conserved hypothetical protein	GER
Bd3816	Bv2188	29346976	60680908	53712758	putative aluminum resistance protein	R
Bd3817	Bv2189	29346974	60680909	53712759	conserved hypothetical protein	R
Bd3819c	Bv2486c	29349891	60682838	53714855	conserved hypothetical protein	n/a
Bd3820c	Bv2487c	29349890	60682839	53714856	conserved hypothetical protein	R
Bd3827	Bv3445	29346195	60681821	53713538	glutamine synthetase I	E
Bd3828	Bv0619	29347310	60682778	53714786	BexA, putative cation effux pump	V
Bd3834	Bv3185c	29347835	60680097	53711869	potassium-transporting ATPase A chain	P
Bd3835	Bv3184c	29347834	60680098	53711870	potassium-transporting ATPase B chain	P
Bd3836	Bv3183c	29347833	60680099	53711871	potassium-transporting ATPase C chain	P
Bd3838	Bv3170c	29347831	60680101	53711873	osmosensitive K+ channel histidine kinase	T
Bd3844c	Bv1565c	29347292	60682745	53714739	putative thiamine-monophosphate kinase	H
Bd3845c	Bv1567c	29347290	60682743	53714737	tetraacyldisaccharide 4'-kinase	M
Bd3846c	Bv1568c	29347289	60682742	53714736	protease IV	OU
Bd3865	Bv0013c	29347573	60683082	53715146	30S ribosomal protein S6	J
Bd3866	Bv0012c	29347572	60683081	53715145	30S ribosomal protein S18	J
Bd3867	Bv0011c	29347571	60683080	53715144	putative 50S ribosomal protein L9	J
Bd3868	Bv4087	29347551	60683065	53715107	putative N-acetylmuramoyl-L-alanine amidase amiA	M
Bd3869	Bv4088	29347552	60683066	53715108	conserved hypothetical protein	n/a

Legend: For an explanation of COG-based functional codes, see **Figure S1**.
