# Supplementary material for: Evolution of Symbiotic Bacteria in the Distal Human Intestine
Source: PLoS Biol. 2007 Jun 19;5(7):e156. doi: 10.1371/journal.pbio.0050156 (PMC1892571; doi:10.1371/journal.pbio.0050156)
Supplement: Supplementary file 7 [file pbio.0050156.st003.pdf]

Table S3. Glycoside hydrolases found in *B. distasonis* ATCC 8503, *B. vulgatus* ATCC 8482, *B. thetaiotaomicron* ATCC 29148 and *B. fragilis* strains NCTC 9343 and YCH 46.

Family ID	*B. distasonis* ATCC 8503	*B. vulgatus* ATCC 8482	*B. thetaiotaomicron* ATCC 29148	*B. fragilis* NCTC 9343	*B. fragilis* YCH 46
**Glycosidases and Transglycosidases**					
**2**	11	25	33	15	16
**3**	8	5	10	10	10
**5**	0	2	1	1	1
**10**	0	1	0	0	0
**13**	9	4	7	6	6
**15**	1	1	0	0	0
**16**	1	1	3	6	6
**18**	0	2	7	2	2
**20**	6	9	14	12	13
**23**	3	3	3	3	3
**24**	1	2	0	0	1
**25**	0	1	1	1	1
**26**	1	0	0	2	2
**27**	0	1	5	3	3
**28**	1	13	9	0	0
**29**	1	10	9	9	9
**30**	2	2	2	0	0
**31**	1	4	6	4	4
**32**	1	1	4	2	2
**33**	1	3	1	3	3
**35**	1	1	3	4	4
**36**	2	2	3	3	3
**38**	1	0	2	1	1
**42**	0	1	1	0	0
**43**	7	22	32	9	9
**51**	3	3	4	2	2
**53**	0	0	1	0	0
**57**	1	1	1	1	1
**63**	1	2	0	0	1
**65**	0	0	0	1	1
**66**	0	0	1	0	0
**67**	0	1	1	0	0
**73**	3	1	1	1	2
**76**	1	0	10	3	3
**77**	1	1	1	1	1
**78**	7	5	6	2	2
**84**	1	1	1	1	1
**88**	0	3	4	1	1
**89**	0	1	3	1	1
**92**	14	9	23	8	9
**93**	0	0	1	0	0
**95**	1	4	5	4	4
**97**	3	9	10	4	4
**99**	0	0	1	0	0
**Unclassified**	2	2	0	0	0
**Sub-total**	**97**	**159**	**226**	**126**	**132**
**Polysaccharide Lyases**					
1	0	1	5	0	0
4	0	1	0	0	0
8	0	0	3	1	1
9	0	2	2	0	0
10	0	1	1	0	0
11	0	2	1	0	0
12	0	0	2	0	0
13	0	0	1	0	0
**Sub-total**	**0**	**7**	**15**	**1**	**1**

Legend: The classification scheme used is described in the Carbohydrate-Active Enzymes database (CAZy) at http://afmb.cnrs-mrs.fr/CAZY/.
