# Supplementary material for: Evolution of Symbiotic Bacteria in the Distal Human Intestine
Source: PLoS Biol. 2007 Jun 19;5(7):e156. doi: 10.1371/journal.pbio.0050156 (PMC1892571; doi:10.1371/journal.pbio.0050156)
Supplement: Supplementary file 8 [file pbio.0050156.st004.pdf]

Table S4. List of putative xenologs in *B. distasonis* ATCC 8503 (A), *B. vulgatus* ATCC 8482 (B), *B. thetaiotaomicron* ATCC 29148 (C), *B. fragilis* NCTC 9343 (D), and *B. fragilis* YCH 46 (E).

(A). Putative xenologs in *B. distasonis* ATCC 8503

Gene	Annotation	COG	LGT
Bd0026	nucleoside-diphosphate-sugar epimerase	MG	LGT-in
Bd0034	conserved hypothetical protein, putative glycosyltransferase	-	LGT-in
Bd0050c	glycoside hydrolase family 20, candidate beta-N-acetylhexosaminidase	G	LGT-unresolved
Bd0053	TonB-dependent receptor, putative	-	LGT-unresolved
Bd0118c	putative sugar nucleotide epimerase	M	LGT-in
Bd0126	putative capsular polysaccharide biosynthesis protein	MG	LGT-in
Bd0167c	dihydroxyacid dehydratase	EG	LGT-in
Bd0236	helicase domain protein	-	LGT-in
Bd0237	conserved hypothetical protein	-	LGT-in
Bd0238	conserved hypothetical protein	R	LGT-in
Bd0267	conserved hypothetical protein	-	LGT-unresolved
Bd0292	glycosyltransferase family 2, related to beta-glycosyltransferases	R	LGT-out
Bd0297c	conserved hypothetical protein	-	LGT-unresolved
Bd0321	conserved hypothetical protein	S	LGT-in
Bd0325c	putative short-chain dehydrogenase	R	LGT-in
Bd0326c	putative NADH:flavin oxidoreductase	C	LGT-in
Bd0327c	putative cation efflux transporter	V	LGT-in
Bd0341	uncharacterized Fe-S center protein, putative ferredoxin	R	LGT-unresolved
Bd0357	TRAP-type C4-dicarboxylate transport system, periplasmic component	G	LGT-unresolved
Bd0358	TRAP-type C4-dicarboxylate transport system, small permease component	G	LGT-in
Bd0359	conserved hypothetical protein	G	LGT-unresolved
Bd0365	AcrB/AcrD family multidrug resistance protein	V	LGT-in
Bd0366	AcrB/AcrD family multidrug resistance protein	V	LGT-in
Bd0400c	conserved hypothetical protein	-	LGT-unresolved
Bd0414	1-aminocyclopropane-1-carboxylate deaminase	E	LGT-unresolved
Bd0415	carbamoylphosphate synthase large subunit (split gene in MJ)	EF	LGT-unresolved
Bd0417	NAD/NADP octopine/nopaline dehydrogenas	-	LGT-unresolved
Bd0422	hypothetical protein	-	LGT-in
Bd0424	putative CPS repeating unit transporter	R	LGT-in
Bd0425	uridine diphosphate glucose dehydrogenase	M	LGT-unresolved
Bd0448c	putative permease	E	LGT-out
Bd0449c	conserved hypothetical protein	-	LGT-unresolved
Bd0491	two-component sensor histidine kinase with response regulator receiver domain	TT	LGT-in
Bd0507c	conserved hypothetical protein, putative membrane-bound serine protease (ClpP class)	O	LGT-unresolved
Bd0540	exopolysaccharide biosynthesis protein	-	LGT-out
Bd0544	conserved hypothetical protein	-	LGT-in
Bd0545	putative xylanase/chitin deacetylase	-	LGT-in
Bd0553	glycosyltransferase family 4, distantly related to alpha-glycosyltransferases	-	LGT-unresolved
Bd0583	conserved hypothetical protein	-	LGT-unresolved
Bd0586	transcriptional regulator, putative nickel responsive regulator	K	LGT-unresolved
Bd0603c	two-component sensor histidine kinase, with response regulator receiver	TT	LGT-in
Bd0604	transcriptional regulator	K	LGT-in
Bd0608	conserved hypothetical protein	S	LGT-in
Bd0610	hypothetical protein	-	LGT-in
Bd0611	conserved hypothetical protein	G	LGT-unresolved
Bd0612	dihydroxyacetone kinase	G	LGT-in
Bd0613	conserved hypothetical protein	G	LGT-in
Bd0631	conserved hypothetical protein	-	LGT-in

Bd0634	GDP-D-mannose dehydratase	M	LGT-unresolved
Bd0640	pyruvate/oxaloacetate carboxyltransferase	C	LGT-in
Bd0647	glycoside hydrolase family 20, candidate beta-N-acetylhexosaminidase	G	LGT-unresolved
Bd0681c	two-component system response regulator	KT	LGT-in
Bd0711	putative DNA-binding protein	-	LGT-in
Bd0717	DNA primase TraC	-	LGT-in
Bd0732	hypothetical protein	-	LGT-in
Bd0733	hypothetical protein	-	LGT-in
Bd0737	serine protease	O	LGT-in
Bd0744c	two-component system sensor histidine kinase, with response regulator receiver domain	TT	LGT-in
Bd0754c	ferredoxin-type protein	CC	LGT-out
Bd0763	xylose-proton symporter	GEPR	LGT-out
Bd0781	putative transport-related, membrane protein	P	LGT-out
Bd0789c	hypothetical protein	-	LGT-unresolved
Bd0822c	conserved hypothetical protein	-	LGT-in
Bd0830	conserved hypothetical protein	-	LGT-in
Bd0837	conserved hypothetical protein	-	LGT-unresolved
Bd0839	site-specific DNA-methyltransferase	L	LGT-in
Bd0840	methyltransferase	L	LGT-in
Bd0844	putative prophage Lp2 protein 26	-	LGT-in
Bd0854	putative DNA methylase (ParB family)	-	LGT-unresolved
Bd0856	putative phage large subunit terminase	-	LGT-in
Bd0893	putative cation transport ATPase	-	LGT-in
Bd0899c	ABC transporter, ATP-binding protein-related protein	-	LGT-unresolved
Bd0904	putative Na+/H+ exchange protein	-	LGT-out
Bd0906c	conserved hypothetical protein	-	LGT-in
Bd0907c	glycoside hydrolase family 13, candidate alpha-glucosidase	G	LGT-unresolved
Bd0934	conserved hypothetical protein	R	LGT-unresolved
Bd0941	hypothetical protein	-	LGT-in
Bd0961c	conserved hypothetical protein	GEPR	LGT-in
Bd0963	cytochrome c peroxidase	P	LGT-in
Bd0965c	putative glucose-fructose oxidoreductase	R	LGT-unresolved
Bd0973	outer membrane protein, putative OmpA domain protein	M	LGT-unresolved
Bd1002	conserved hypothetical protein	-	LGT-in
Bd1019	NADP-reducing hydrogenase B chain - Desulfovibrio fructosovorans gi|466364|gb|AAA87055.1| potential NAD-reducing hydrogenase subunit	-	LGT-unresolved
Bd1066c	conserved hypothetical protein	-	LGT-in
Bd1067c	predicted metal-dependent phosphoesterase (PHP family)	-	LGT-unresolved
Bd1069c	ferredoxin 2	C	LGT-in
Bd1070c	putative anti-sigma regulatory factor (Ser/Thr protein kinase)	T	LGT-in
Bd1102c	guanine aminohydrolase	FR	LGT-in
Bd1116	glycoside hydrolase family 84, related to beta-N-acetylhexosaminidases	-	LGT-out
Bd1152	putative helicase	R	LGT-unresolved
Bd1153	conserved hypothetical protein	-	LGT-unresolved
Bd1154	conserved hypothetical protein	-	LGT-unresolved
Bd1155	uncharacterized protein predicted to be involved in DNA repair	L	LGT-unresolved
Bd1156	exonuclease, RecB family	-	LGT-unresolved
Bd1157	conserved hypothetical protein	L	LGT-unresolved
Bd1158	uncharacterized protein predicted to be involved in DNA repair	L	LGT-in
Bd1188	conserved hypothetical protein	-	LGT-out
Bd1221	4-carboxymuconolactone decarboxylase	S	LGT-in
Bd1223c	transcriptional regulator	TK	LGT-unresolved
Bd1224	aminotransferase (subgroup I)	KE	LGT-unresolved

Bd1225c	transcriptional regulator	KE	LGT-in
Bd1227c	hypothetical protein	-	LGT-in
Bd1264	glycoside hydrolase family 73, related to peptidoglycan hydrolases	-	LGT-in
Bd1279c	UDP-N-acetyl-D-mannosaminuronate dehydrogenase	M	LGT-unresolved
Bd1284c	conserved hypothetical protein	-	LGT-unresolved
Bd1298c	putative peptidase	E	LGT-unresolved
Bd1299c	D-threonine aldolase, metal-activated pyridoxal enzyme	E	LGT-unresolved
Bd1300c	putative translation initiation inhibitor	J	LGT-unresolved
Bd1302c	H+/gluconate symporter and related permease	GE	LGT-unresolved
Bd1313c	conserved hypothetical protein	-	LGT-out
Bd1316c	Fe-S oxidoreductase	C	LGT-in
Bd1319c	putative glycero-phosphotransferase	M	LGT-in
Bd1320c	ribulose-phosphate 3-epimerase	G	LGT-unresolved
Bd1321c	conserved hypothetical protein	ER	LGT-unresolved
Bd1340	conserved hypothetical protein	-	LGT-in
Bd1341	putative ABC transporter	PE	LGT-in
Bd1352c	putative transmembrane cation efflux pump	V	LGT-unresolved
Bd1355c	putative transport protein	GEPR	LGT-unresolved
Bd1377c	glycosyltransferase family 4, related to alpha-glycosyltransferases	M	LGT-unresolved
Bd1379c	acetyltransferase (isoleucine patch superfamily)	R	LGT-unresolved
Bd1383c	exopolysaccharide biosynthesis protein, putative galactosyltransferase	-	LGT-in
Bd1394c	excinuclease ABC, subunit A, daunorubicin resistance protein	L	LGT-in
Bd1395c	conserved hypothetical protein	-	LGT-unresolved
Bd1397	glycoside hydrolase family 20, distantly related to beta-N-acetylhexosaminidases	-	LGT-unresolved
Bd1412	Fe-S oxidoreductase	C	LGT-unresolved
Bd1416	ABC-type multidrug transport system, ATPase component	V	LGT-in
Bd1425	conserved hypothetical protein	S	LGT-unresolved
Bd1428	conserved hypothetical protein	-	LGT-in
Bd1434	putative ornithine cyclodeaminase	E	LGT-in
Bd1436	O-antigen transporter	R	LGT-unresolved
Bd1437c	putative cell wall surface anchor family protein	-	LGT-unresolved
Bd1447	acetyltransferase	J	LGT-in
Bd1452c	putative fructokinase	G	LGT-out
Bd1455c	alginate O-acetylation protein, putative alginate o-acetyltransferase	M	LGT-unresolved
Bd1469c	uncharacterized protein, possibly involved in aromatic compounds catabolism	Q	LGT-in
Bd1472c	conserved hypothetical protein	R	LGT-out
Bd1474c	conserved hypothetical protein	-	LGT-out
Bd1481	glycoside hydrolase family 73, related to peptidoglycan hydrolases	-	LGT-unresolved
Bd1490	conserved hypothetical protein	O	LGT-unresolved
Bd1492	conserved hypothetical protein	-	LGT-out
Bd1504c	putative fosmidomycin resistance protein	GEPR	LGT-in
Bd1512c	conserved hypothetical protein	-	LGT-unresolved
Bd1544c	glycosyltransferase family 4, distantly related to alpha-glycosyltransferases	M	LGT-out
Bd1547c	conserved hypothetical protein, putative prenyltransferase and squalene oxidase	-	LGT-in
Bd1548c	putative zinc-binding dehydrogenase	R	LGT-in
Bd1581c	beta-glucosidase, partial	-	LGT-in
Bd1593c	conserved hypothetical protein	-	LGT-in
Bd1616c	spermidine/putrescine transport ATP-binding protein	E	LGT-out
Bd1618c	fumarase	C	LGT-in
Bd1624c	putative large exoprotein involved in heme utilization or adhesion	-	LGT-in
Bd1631c	putative outer membrane protein/protective antigen OMA87	-	LGT-unresolved
Bd1641	glycoside hydrolase family 76, related to an ill-defined alpha-mannanase	-	LGT-unresolved
Bd1643c	arylsulfatase A	P	LGT-unresolved
Bd1653	TPR-domain containing protein	-	LGT-in

Bd1667	Na+/H+ antiporter	P	LGT-unresolved
Bd1675c	putative metal-binding protein	-	LGT-unresolved
Bd1678c	conserved hypothetical protein	GER	LGT-in
Bd1711c	conserved hypothetical protein	-	LGT-unresolved
Bd1712c	ROK family transcriptional repressor	KG	LGT-out
Bd1721	arginase	E	LGT-in
Bd1734	putative oxidoreductase MocA family	C	LGT-in
Bd1736c	pyruvate-flavodoxin oxidoreductase, partial	CC	LGT-in
Bd1737c	pyruvate-flavodoxin oxidoreductase, partial	CC	LGT-in
Bd1740c	conserved hypothetical protein, putative Superfamily I DNA helicase	-	LGT-in
Bd1755c	glycoside hydrolase family 78, related to alpha-L-rhamnosidases	-	LGT-unresolved
Bd1763c	hydrogenase accessory protein HypB	OK	LGT-unresolved
Bd1764c	hydrogenase expression/formation protein HupA	R	LGT-in
Bd1765c	hydrogenase maturation factor	O	LGT-in
Bd1766	hydrogenase expression/formation protein; HypC	O	LGT-in
Bd1767	hydrogenase isoenzymes formation protein; HypD	O	LGT-unresolved
Bd1768	hydrogenase expression/formation protein HypE	O	LGT-in
Bd1769	putative uptake hydrogenase small subunit precursor (hydrogenlyase)	C	LGT-unresolved
Bd1770	stable NiFe hydrogenase large subunit	C	LGT-in
Bd1771	NiFe hydrogenase small subunit, HupC	C	LGT-in
Bd1772	Ni,Fe-hydrogenase maturation factor	C	LGT-in
Bd1773c	glycerol uptake facilitator protein	G	LGT-unresolved
Bd1775c	glycerol-3-phosphate dehydrogenase	C	LGT-unresolved
Bd1783	nucleoside transporter	F	LGT-unresolved
Bd1796c	lipopolysaccharide biosynthesis protein	-	LGT-unresolved
Bd1798c	glycosyltransferase family 4, related to alpha-glycosyltransferases	M	LGT-in
Bd1801c	polysaccharide pyruvyl transferase YvfF	-	LGT-in
Bd1817	transcriptional regulator/glucokinase	KG	LGT-unresolved
Bd1818	putative N-acetylmannosamine-6-phosphate 2-epimerase (ManNAc-6-P epimerase)	G	LGT-unresolved
Bd1821	putative thiogalactoside transacetylase	R	LGT-unresolved
Bd1829	conserved hypothetical protein	R	LGT-in
Bd1842	conserved hypothetical protein, putative ATPase involved in DNA repair	-	LGT-unresolved
Bd1860c	RNA polymerase ECF-type sigma factor	-	LGT-in
Bd1901c	conserved hypothetical protein	-	LGT-unresolved
Bd1908	putative phosphoesterase, PAP2 family	-	LGT-unresolved
Bd1917	hypothetical protein	-	LGT-out
Bd1925c	ABC transporter permease	M	LGT-unresolved
Bd1963	conserved hypothetical protein	-	LGT-in
Bd1979c	conserved hypothetical protein	-	LGT-in
Bd1999c	putative NADH-dependent dyhydrogenase	R	LGT-unresolved
Bd2017c	hypothetical protein	-	LGT-in
Bd2019c	hypothetical protein	-	LGT-in
Bd2035	putative multidrug resistance protein	P	LGT-in
Bd2073	uncharacterized Fe-S center protein, putative ferredoxin	R	LGT-in
Bd2078c	conserved hypothetical protein	S	LGT-unresolved
Bd2096	conserved hypothetical protein	R	LGT-in
Bd2097	conserved hypothetical protein	-	LGT-unresolved
Bd2103	conserved hypothetical protein	S	LGT-unresolved
Bd2104	conserved hypothetical protein, putative ATPase involved in DNA repair	-	LGT-unresolved
Bd2153c	mannose-6-phosphate isomerase (phosphomannose isomerase) ; mannose-1-phosphate guanylyl transferase (GDP-mannose pyrophosphorylase)	GM	LGT-unresolved
Bd2154c	GDP-4-keto-6-deoxy-D-mannose-3,5-epimerase	MG	LGT-unresolved
Bd2155c	GDP-mannose 4,6-dehydratase	M	LGT-out

Bd2157c	acetyltransferase (isoleucine patch superfamily)	R	LGT-unresolved
Bd2158c	glycosyltransferase family 4, distantly related to alpha-glycosyltransferases	M	LGT-unresolved
Bd2168c	glycosyltransferase family 4, distantly related to alpha-glycosyltransferases	M	LGT-in
Bd2185	transposase	-	LGT-out
Bd2227c	translation initiation factor eIF-2B alpha subunit	J	LGT-unresolved
Bd2232	putative beta-lactamase	V	LGT-unresolved
Bd2233	conserved hypothetical protein	-	LGT-in
Bd2251	conserved hypothetical protein	-	LGT-in
Bd2253	D-tagatose 3-epimerase	G	LGT-unresolved
Bd2260c	arylsulfatase A	P	LGT-in
Bd2267c	arylsulfatase A	P	LGT-unresolved
Bd2275	conserved hypothetical protein, putative lysophospholipase	-	LGT-unresolved
Bd2294c	conserved hypothetical protein	-	LGT-out
Bd2356c	putative beta-agarase precursor	-	LGT-in
Bd2358	putative outer membrane protein, probably involved in nutrient binding	-	LGT-unresolved
Bd2359	putative outer membrane protein, probably involved in nutrient binding	-	LGT-unresolved
Bd2360	glycoside hydrolase family 78, related to alpha-L-rhamnosidases	-	LGT-unresolved
Bd2376	conserved hypothetical protein	-	LGT-in
Bd2386	conserved hypothetical protein	-	LGT-in
Bd2389	conserved hypothetical protein	-	LGT-in
Bd2390	conserved hypothetical protein	-	LGT-in
Bd2391	conserved hypothetical protein	-	LGT-unresolved
Bd2392	conserved hypothetical protein	-	LGT-in
Bd2393	conserved hypothetical protein, putative phage baseplate assembly protein W	-	LGT-unresolved
Bd2395	conserved hypothetical protein	-	LGT-in
Bd2396	hypothetical protein	-	LGT-in
Bd2399c	ATPase of the AAA+ class	O	LGT-unresolved
Bd2400c	putative DNA segregation ATPase FtsK/SpoIIIE and related proteins	-	LGT-in
Bd2406c	conserved hypothetical protein	-	LGT-unresolved
Bd2454c	UDP-N-acetylmuramate-alanine ligase	M	LGT-in
Bd2533c	hypothetical protein	-	LGT-unresolved
Bd2569c	conserved hypothetical protein	-	LGT-in
Bd2579	hypothetical protein	-	LGT-in
Bd2601c	gluconolactonase precursor	G	LGT-in
Bd2612c	arginase	E	LGT-unresolved
Bd2652c	adenine-specific DNA methylase	L	LGT-unresolved
Bd2654	putative glycosyl hydrolase	-	LGT-in
Bd2671	glycoside hydrolase family 92, related to an ill-defined alpha-1,2-mannosidase	G	LGT-out
Bd2673	conserved hypothetical protein	-	LGT-in
Bd2675c	putative signal transduction histidine kinase	T	LGT-in
Bd2676c	conserved hypothetical protein	-	LGT-unresolved
Bd2710	glycosyltransferase family 4, distantly related to alpha-glycosyltransferases	-	LGT-unresolved
Bd2742c	alanine dehydrogenase	E	LGT-unresolved
Bd2750	glycosyltransferase family 4, related to alpha-glycosyltransferases	M	LGT-unresolved
Bd2756c	two-component system sensor histidine kinase	T	LGT-in
Bd2804	putative dehydrogenase	R	LGT-in
Bd2806	putative galactosamine-6-phosphate isomerase	G	LGT-unresolved
Bd2821	conserved hypothetical protein	R	LGT-unresolved
Bd2833c	putative serine protease	-	LGT-in
Bd2851	glycosyltransferase family 32, related to alpha-glycosyltransferases	-	LGT-in
Bd2852	hypothetical protein	-	LGT-unresolved
Bd2858	glycosyltransferase family 2, related to beta-glycosyltransferases	M	LGT-in
Bd2860	glycosyltransferase family 2, will be annotated when complete	M	LGT-in
Bd2880	2-amino-3-ketobutyrate CoA ligase	H	LGT-unresolved
Bd2884c	conserved hypothetical protein	R	LGT-unresolved

Bd2895	adenine deaminase	F	LGT-in
Bd2905c	putative dinitrogenase reductase activating glycohydrolase	O	LGT-unresolved
Bd2908c	Type III restriction-modification enzyme, helicase subunit	-	LGT-in
Bd2909c	adenine specific DNA methylase (Mod-related)	L	LGT-unresolved
Bd2917	periplasmic component of the Tol biopolymer transport system	U	LGT-unresolved
Bd2929c	conserved hypothetical protein	-	LGT-in
Bd2930c	site-specific DNA methylase	L	LGT-unresolved
Bd2941	acyltransferase	-	LGT-unresolved
Bd2946	conserved hypothetical protein	-	LGT-in
Bd2974	hypothetical protein	-	LGT-in
Bd2993	glycoside hydrolase family 43, candidate beta-glycosidase; central CBM32 module	-	LGT-in
Bd2996	glycoside hydrolase family 3, candidate beta-glucosidase	G	LGT-unresolved
Bd3046	conserved hypothetical protein	-	LGT-in
Bd3056	conserved hypothetical protein	-	LGT-unresolved
Bd3089	conserved hypothetical protein	-	LGT-in
Bd3106c	putative secreted protein	-	LGT-unresolved
Bd3112c	uronate isomerase	G	LGT-in
Bd3133c	glycosyltransferase family 1, candidate beta-glycosyltransferase	GC	LGT-in
Bd3147c	putative biotin apo-protein ligase-related protein	-	LGT-in
Bd3153c	mannose-6-phosphate isomerase (phosphomannose isomerase) ; mannose-1-phosphate guanylyl transferase (GDP-mannose pyrophosphorylase)	GM	LGT-unresolved
Bd3158c	glycosyltransferase family 4, related to alpha-glycosyltransferases	M	LGT-in
Bd3161c	glycosyltransferase family 8, related to alpha-glycosyltransferases	M	LGT-unresolved
Bd3162c	putative O-acetyltransferase	-	LGT-in
Bd3179	putative DNA-methyltransferase	L	LGT-unresolved
Bd3180	Type II restriction enzyme EcoRI	-	LGT-in
Bd3182	putative transcriptional regulator	-	LGT-unresolved
Bd3197	conserved hypothetical protein	-	LGT-in
Bd3218	putative glucose-6-phosphate isomerase	-	LGT-in
Bd3220	putative thermophilic glucose-6-phosphate isomerase and related metalloenzymes	-	LGT-unresolved
Bd3234	conserved hypothetical protein	J	LGT-in
Bd3245	putative hydrolase	R	LGT-unresolved
Bd3273	DNA topoisomerase IV subunit B	L	LGT-unresolved
Bd3299	putative fructokinase	G	LGT-out
Bd3306	glycoside hydrolase family 2, candidate beta-glycosidase; related to beta-mannosidases	G	LGT-unresolved
Bd3313c	putative outer membrane protein, probably involved in nutrient binding	-	LGT-unresolved
Bd3317	formate acetyltransferase	C	LGT-unresolved
Bd3338c	haloacid dehalogenase-like hydrolase	R	LGT-unresolved
Bd3346c	conserved hypothetical protein	R	LGT-in
Bd3354	conserved hypothetical protein	-	LGT-in
Bd3355	conserved hypothetical protein	-	LGT-unresolved
Bd3357c	conserved hypothetical protein	-	LGT-unresolved
Bd3376	conserved hypothetical protein	-	LGT-in
Bd3388	hypothetical protein	-	LGT-unresolved
Bd3391	conserved hypothetical protein	-	LGT-in
Bd3421	two-component system sensor histidine kinase/response regulator, hybrid ('one-component system')	TK	LGT-unresolved
Bd3434	putative thiol-disulfide isomerase and thioredoxins	OC	LGT-in
Bd3435	conserved hypothetical protein	R	LGT-in
Bd3448	conserved hypothetical protein	-	LGT-in
Bd3483	putative dehydrogenase	R	LGT-in

Bd3484	sugar phosphate isomerase/epimerase	G	LGT-unresolved
Bd3490c	conserved hypothetical protein	E	LGT-unresolved
Bd3499	phosphoribosylglycinamide formyltransferase	F	LGT-in
Bd3540	conserved hypothetical protein	-	LGT-unresolved
Bd3542	putative flippase, involved in lipopolysaccharide biosynthesis	R	LGT-unresolved
Bd3544	hypothetical protein	-	LGT-in
Bd3546	2-C-methyl-D-erythritol 4-phosphate cytidylyltransferase	I	LGT-unresolved
Bd3548	lipopolysaccharide biosynthesis protein	M	LGT-unresolved
Bd3549	putative acetyltransferase	R	LGT-unresolved
Bd3554	putative xylanase/chitin deacetylase	G	LGT-unresolved
Bd3564	putative DNA binding protein	-	LGT-unresolved
Bd3583	conserved hypothetical protein	-	LGT-unresolved
Bd3619c	membrane protein related to metalloendopeptidase	M	LGT-in
Bd3675	translation elongation factor G	J	LGT-in
Bd3695	glycoside hydrolase family 30, candidate beta-glycosidase	-	LGT-out
Bd3730	conserved hypothetical protein, putative glycosyltransferase	-	LGT-unresolved
Bd3734	conserved hypothetical protein	-	LGT-in
Bd3742	conserved hypothetical protein	-	LGT-unresolved
Bd3744	diaminopimelate decarboxylase	E	LGT-out
Bd3746	UDP-glucose 4-epimerase (galactowaldenase)	MG	LGT-unresolved
Bd3756c	metallo-beta-lactamase superfamily protein	Q	LGT-in
Bd3759c	conserved hypothetical protein	-	LGT-unresolved
Bd3764	femAB family protein	V	LGT-unresolved
Bd3823	arylsulfatase A	P	LGT-in
Bd3829c	NhaP-type Na+/H+ and K+/H+ antiporters	P	LGT-unresolved
Bd3851c	conserved hypothetical protein	-	LGT-in
Bd3856c	putative pseudouridine synthase family 1 protein	J	LGT-in

(B). Putative xenologs in *B. vulgatus* ATCC 8482

Gene	Annotation	COG	LGT
Bv0006c	putative DNA repair photolyase	L	LGT-unresolved
Bv0017c	putative elongation factor G	J	LGT-in
Bv0071	conserved hypothetical protein	R	LGT-unresolved
Bv0091c	conserved hypothetical protein	-	LGT-in
Bv0119c	putative beta-lactamase class C and other penicillin binding proteins	V	LGT-in
Bv0121c	glycoside hydrolase family 28, distantly related to polygalacturonases	-	LGT-out
Bv0153c	putative H-repeat protein	-	LGT-in
Bv0154c	transposase	-	LGT-unresolved
Bv0159c	polysaccharide lyase family 11, candidate rhamnogalacturonan lyase	-	LGT-in
Bv0174	polysaccharide lyase family 11, related to rhamnogalacturonan lyases	-	LGT-unresolved
Bv0177c	glycoside hydrolase family 42, related to beta-galactosidases	G	LGT-unresolved
Bv0178c	glycoside hydrolase family 43, modular protein with tandem of two domains distantly related to beta-glycosidases and C-terminal CBM32 module	-	LGT-unresolved
Bv0250	putative alkaline protease AprF, partial	-	LGT-in
Bv0251	putative alkaline protease AprF, partial	MU	LGT-in
Bv0277c	putative moaA/nifB/pqqE family protein, involved in molybdenum cofactor biosynthesis	R	LGT-in
Bv0319	conserved hypothetical protein	-	LGT-unresolved
Bv0321	putative histidine decarboxylase	-	LGT-in
Bv0343c	putative ABC transporter, periplasmic sugar-binding protein	-	LGT-in
Bv0345c	ABC-type sugar transport system, periplasmic component	G	LGT-in
Bv0347c	conserved hypothetical protein	G	LGT-unresolved
Bv0348c	glycerol kinase 2 (ATP:glycerol 3-phosphotransferase 2)	C	LGT-unresolved
Bv0349c	transketolase, C-terminal subunit	G	LGT-unresolved
Bv0350c	transketolase, N-terminal subunit	G	LGT-unresolved
Bv0351c	transcriptional regulator of sugar metabolism	KG	LGT-in
Bv0352c	3,4-dihydroxy-2-butanone 4-phosphate synthase (cyclohydrolase II)	H	LGT-unresolved
Bv0353c	conserved hypothetical protein	-	LGT-unresolved
Bv0359c	conserved hypothetical protein	-	LGT-unresolved
Bv0382	glycoside hydrolase family 20, distantly related to beta-N-acetylhexosaminidases	G	LGT-unresolved
Bv0385	hypothetical protein	-	LGT-in
Bv0418	putative site-specific recombinase	-	LGT-unresolved
Bv0539c	glycoside hydrolase family 29, candidate alpha-L-fucosidase (C-terminal	-	LGT-in
Bv0565c	arylsulfate sulfotransferase	-	LGT-in
Bv0582c	conserved hypothetical protein, putative ABC-type xylose transporter	-	LGT-unresolved
Bv0651	conserved hypothetical protein	S	LGT-in
Bv0663c	helicase, putative	KL	LGT-in
Bv0705c	DNA primase	-	LGT-in
Bv0707	conserved hypothetical protein	-	LGT-in
Bv0711c	conserved hypothetical protein	E	LGT-in
Bv0740	alcohol dehydrogenase	C	LGT-unresolved
Bv0806	acetyltransferase	J	LGT-in
Bv0838	putative outer membrane protein	M	LGT-in
Bv0921	putative oxidoreductase (putative secreted protein)	R	LGT-unresolved
Bv0981	3-oxoacyl-[acyl-carrier-protein] synthase I	IQ	LGT-in
Bv0983	putative acyltransferase	-	LGT-unresolved
Bv0985	conserved hypothetical protein, putative 3-hydroxymyristoyl/3-hydroxydecanoyl-(acyl carrier protein) dehydratase	-	LGT-unresolved
Bv0986	putative thioesterase	R	LGT-unresolved
Bv0989	3-oxoacyl-[acyl-carrier-protein] synthase	IQ	LGT-out
Bv0992	putative xylanase/chitin deacetylase	G	LGT-unresolved
Bv0994	hypothetical protein	-	LGT-in

Bv0998	glycosyltransferase family 2, related to beta-glycosyltransferases	M	LGT-unresolved
Bv0999	putative exporter	I	LGT-in
Bv1001	putative choloylglycine hydrolase	-	LGT-in
Bv1003	conserved hypothetical protein	-	LGT-in
Bv1084c	conserved hypothetical protein	-	LGT-unresolved
Bv1090c	peptidase	E	LGT-unresolved
Bv1120	glycoside hydrolase family 2, candidate beta-glycosidase	G	LGT-out
Bv1128	sulfatase	P	LGT-unresolved
Bv1188	conserved hypothetical protein	-	LGT-unresolved
Bv1241	glycoside hydrolase family 18, related to chitinases	G	LGT-unresolved
Bv1255c	putative transcriptional regulator	-	LGT-in
Bv1272c	hypothetical protein	-	LGT-in
Bv1401c	putative mannose-1-phosphate guanylyltransferase	M	LGT-in
Bv1409c	conserved hypothetical protein	-	LGT-in
Bv1410c	conserved hypothetical protein	-	LGT-unresolved
Bv1420c	transcriptional regulator	-	LGT-unresolved
Bv1425c	putative streptothricin acetyltransferase	-	LGT-in
Bv1446	capsular polysaccharide biosynthesis protein D	MG	LGT-unresolved
Bv1461c	conserved hypothetical protein	-	LGT-in
Bv1468c	conserved hypothetical protein	-	LGT-out
Bv1516	helicase, putative	KL	LGT-in
Bv1583c	putative aminopeptidase	R	LGT-out
Bv1649c	putative transposase	-	LGT-in
Bv1691c	putative ABC transporter subunit	S	LGT-in
Bv1692c	putative cell surface protein, putative surface antigen BspA	-	LGT-in
Bv1701c	glycoside hydrolase family 28, candidate polygalacturonase	M	LGT-unresolved
Bv1743	aldehyde dehydrogenase A	C	LGT-unresolved
Bv1753	putative outer membrane protein, probably involved in nutrient binding	-	LGT-out
Bv1754	putative outer membrane protein, probably involved in nutrient binding	-	LGT-out
Bv1755	glycoside hydrolase family 33, distantly related to sialidases	S	LGT-in
Bv1757	carbohydrate esterase family 8, distantly related to pectin methylesterases	-	LGT-out
Bv1762	glycoside hydrolase family 2, candidate beta-glycosidase	G	LGT-out
Bv1766c	putative pectin degradation protein	S	LGT-in
Bv1767	polysaccharide lyase family 10, related to pectate lyases	-	LGT-in
Bv1806	putative outer membrane protein, probably involved in nutrient binding	-	LGT-unresolved
Bv1811c	glutaryl-7-ACA acylase precursor	R	LGT-unresolved
Bv1824c	conserved hypothetical protein	-	LGT-unresolved
Bv1841	conserved hypothetical protein	-	LGT-unresolved
Bv1849	putative ADP-ribosylglycohydrolase	-	LGT-in
Bv1955c	peptide methionine sulfoxide reductase	O	LGT-in
Bv1970	putative pectinesterase precursor, partial	-	LGT-in
Bv1993c	conserved hypothetical protein	-	LGT-unresolved
Bv2045c	conserved hypothetical protein	-	LGT-in
Bv2049c	conserved hypothetical protein	-	LGT-in
Bv2098	conserved hypothetical protein	-	LGT-unresolved
Bv2111	glycoside hydrolase family 28, related to polygalacturonases	GM	LGT-in
Bv2169	putative outer membrane protein, probably involved in nutrient binding	-	LGT-unresolved
Bv2170	putative outer membrane protein, probably involved in nutrient binding	-	LGT-unresolved
Bv2185	conserved hypothetical protein	-	LGT-unresolved
Bv2186	ornithine/acetylornithine aminotransferase	E	LGT-unresolved
Bv2219	conserved hypothetical protein	-	LGT-in
Bv2233	site-specific recombinase	-	LGT-unresolved
Bv2324	conserved hypothetical protein	-	LGT-in
Bv2378c	conserved hypothetical protein	-	LGT-in
Bv2574	ATP-dependent DNA helicase	L	LGT-unresolved

Bv2590c	glycosyltransferase family 2, related to beta-glycosyltransferases	M	LGT-in
Bv2596c	acylneuraminate cytidylyltransferase, putative	M	LGT-unresolved
Bv2597c	putative aldolase	E	LGT-out
Bv2604c	conserved hypothetical protein	-	LGT-in
Bv2622c	conserved hypothetical protein	-	LGT-in
Bv2657c	conserved hypothetical protein	-	LGT-out
Bv2667	conserved hypothetical protein	-	LGT-out
Bv2686	conserved hypothetical protein	-	LGT-out
Bv2698c	conserved hypothetical protein	-	LGT-unresolved
Bv2713	adenosylmethionine-8-amino-7-oxononanoate aminotransferase, partial	H	LGT-unresolved
Bv2761c	conserved hypothetical protein	-	LGT-in
Bv2768c	conserved hypothetical protein	-	LGT-in
Bv2770c	putative terminase	-	LGT-in
Bv2771c	conserved hypothetical protein	-	LGT-in
Bv2774c	hypothetical protein	-	LGT-in
Bv2778c	putative sulfate adenylate transferase, subunit 2	EH	LGT-in
Bv2779c	putative type I restriction-modification system methyltransferase subunit	-	LGT-in
Bv2795c	conserved hypothetical protein	-	LGT-unresolved
Bv2802	conserved hypothetical protein	-	LGT-in
Bv2803	putative outer membrane protein	T	LGT-in
Bv2812	transposase	-	LGT-in
Bv2834c	conserved hypothetical protein	-	LGT-in
Bv2835c	conserved hypothetical protein	-	LGT-in
Bv2876	conserved hypothetical protein, putative polysaccharide polymerase	-	LGT-in
Bv2879	putative epimerase/dehydratase	MG	LGT-in
Bv2894	putative helicase	KL	LGT-in
Bv2952	lipoate-protein ligase B	HKR	LGT-in
Bv3022c	conserved hypothetical protein	-	LGT-unresolved
Bv3059c	conserved hypothetical protein	-	LGT-in
Bv3095c	TPR repeat protein	R	LGT-in
Bv3119	putative cation channel family protein	P	LGT-in
Bv3120	transposase	-	LGT-in
Bv3131	putative cation channel family protein	P	LGT-unresolved
Bv3299	conserved hypothetical protein	-	LGT-in
Bv3304	putative DNA mismatch repair protein mutS	L	LGT-in
Bv3324c	ATP-dependent exoDNAse (exonuclease V), alpha subunit - helicase superfamily I member	L	LGT-unresolved
Bv3325c	type I restriction enzyme HsdR, putative	V	LGT-in
Bv3327c	type I restriction-modification system S subunit	V	LGT-in
Bv3328c	type I restriction-modification system M subunit	V	LGT-unresolved
Bv3338	putative SOS-response transcriptional repressor (RecA-mediated autopeptidase)	KT	LGT-in
Bv3342	hypothetical protein	-	LGT-in
Bv3344c	transcriptional regulator	-	LGT-unresolved
Bv3357c	glycosyltransferase family 26, related to beta-glycosyltransferases	M	LGT-in
Bv3359c	putative glycosyltransferase	R	LGT-unresolved
Bv3373	conserved hypothetical protein	-	LGT-in
Bv3414c	transposase	-	LGT-in
Bv3435c	ferritin A	-	LGT-in
Bv3457c	glutathionylspermidine synthase	E	LGT-in
Bv3499c	conserved hypothetical protein	-	LGT-in
Bv3501	putative transmembrane AcrB/D/F-family transporter	V	LGT-in
Bv3502	transporter, AcrB/D/F family	V	LGT-in
Bv3521c	aldose 1-epimerase precursor, partial	G	LGT-unresolved
Bv3522c	aldose 1-epimerase precursor, partial	G	LGT-in
Bv3560	hypothetical protein	-	LGT-out

Bv3563	putative transposase	L	LGT-in
Bv3564	putative transposase	L	LGT-in
Bv3565	putative transposase	L	LGT-in
Bv3566c	conserved hypothetical protein	-	LGT-in
Bv3568c	conserved hypothetical protein	-	LGT-unresolved
Bv3569c	conserved hypothetical protein	-	LGT-unresolved
Bv3570c	conserved hypothetical protein	-	LGT-in
Bv3571c	conserved hypothetical protein	-	LGT-in
Bv3588	type I restriction endonuclease S subunit	-	LGT-in
Bv3594	conserved hypothetical protein	-	LGT-in
Bv3643c	conserved hypothetical protein	-	LGT-in
Bv3645	conserved hypothetical protein	-	LGT-in
Bv3646	putative N6-adeinine specific methyltransferase	-	LGT-unresolved
Bv3648	putative ATP-dependent helicase	KL	LGT-in
Bv3649	conserved hypothetical protein	-	LGT-out
Bv3656	conserved hypothetical protein	-	LGT-in
Bv3657	conserved hypothetical protein	-	LGT-in
Bv3658	conserved hypothetical protein	-	LGT-unresolved
Bv3660	conserved hypothetical protein	-	LGT-out
Bv3668c	conserved hypothetical protein	-	LGT-in
Bv3671	putative transposase	L	LGT-unresolved
Bv3698c	conserved hypothetical protein	-	LGT-in
Bv3699c	hypothetical protein	-	LGT-in
Bv3700c	hypothetical protein	-	LGT-in
Bv3703c	conserved hypothetical protein	-	LGT-in
Bv3774	conserved hypothetical protein	-	LGT-unresolved
Bv3861	glycosyltransferase	-	LGT-unresolved
Bv3865c	ABC-type polysaccharide/polyol phosphate transport system, ATPase component	GM	LGT-unresolved
Bv3866c	ABC-type polysaccharide/polyol phosphate export systems, permease component	GM	LGT-unresolved
Bv3888c	type II restriction enzyme HpaII	-	LGT-out
Bv3908c	putative helicase	KL	LGT-in
Bv3917c	transposase	-	LGT-in
Bv3922c	putative pyridoxal phosphate-dependent enzyme apparently involved in regulation of cell wall biogenesis	M	LGT-in
Bv3926c	putative pyridoxal phosphate-dependent enzyme apparently involved in regulation of cell wall biogenesis	M	LGT-unresolved
Bv3929c	conserved hypothetical protein	-	LGT-in
Bv3931c	putative 4-hydroxy-2-ketovalerate aldolase	E	LGT-out
Bv4001	conserved hypothetical protein	-	LGT-out
Bv4048c	ATP-binding protein of ABC transporter	V	LGT-unresolved
Bv4051c	putative cell surface protein	-	LGT-in
Bv4060	conserved hypothetical protein	-	LGT-in
Bv4061	conserved hypothetical protein	-	LGT-unresolved
Bv4064	conserved hypothetical protein	S	LGT-unresolved
Bv4065	conserved hypothetical protein	-	LGT-in
Bv4083c	TonB-dependent outer membrane receptor	-	LGT-in
Bv4084c	conserved hypothetical protein	-	LGT-in

(C). Putative xenologs in *B. thetaiotaomicron* ATCC 29148

Gene	Annotation	COG	LGT
gi|29345415|ref|NP_808918.1|	30S ribosomal protein S17	J	LGT-in
gi|29345419|ref|NP_808922.1|	phosphate-binding periplasmic protein precursor, putative ABC transporter	P	LGT-unresolved
gi|29345434|ref|NP_808937.1|	transposase	L	LGT-in
gi|29345437|ref|NP_808940.1|	putative transposase	L	LGT-in
gi|29345439|ref|NP_808942.1|	putative outer membrane protein, probably involved in nutrient binding	-	LGT-out
gi|29345440|ref|NP_808943.1|	putative outer membrane protein, probably involved in nutrient binding	-	LGT-out
gi|29345457|ref|NP_808960.1|	putative glycosyltransferase	M	LGT-in
gi|29345464|ref|NP_808967.1|	putative glycosyltransferase	-	LGT-in
gi|29345465|ref|NP_808968.1|	putative glycosyltransferase	M	LGT-unresolved
gi|29345479|ref|NP_808982.1|	hypothetical protein BT0069	L	LGT-in
gi|29345480|ref|NP_808983.1|	hypothetical protein BT0070	L	LGT-unresolved
gi|29345493|ref|NP_808996.1|	hypothetical protein BT0083	-	LGT-in
gi|29345553|ref|NP_809056.1|	putative transmembrane protein	-	LGT-in
gi|29345555|ref|NP_809058.1|	putative beta-xylosidase	G	LGT-in
gi|29345602|ref|NP_809105.1|	cell surface protein	S	LGT-in
gi|29345606|ref|NP_809109.1|	putative hexose phosphate transport protein	G	LGT-unresolved
gi|29345614|ref|NP_809117.1|	hypothetical protein BT0204	-	LGT-unresolved
gi|29345617|ref|NP_809120.1|	putative outer membrane protein, probably involved in nutrient binding	-	LGT-out
gi|29345620|ref|NP_809123.1|	leucine-rich repeat protein, function unknown	-	LGT-in
gi|29345675|ref|NP_809178.1|	hypothetical protein BT0265	-	LGT-unresolved
gi|29345685|ref|NP_809188.1|	hypothetical protein BT0275	-	LGT-in
gi|29345688|ref|NP_809191.1|	hypothetical protein BT0278	-	LGT-in
gi|29345690|ref|NP_809193.1|	transposase for insertion sequence element ISRM3	L	LGT-out
gi|29345695|ref|NP_809198.1|	putative tolQ-type transport protein	U	LGT-unresolved
gi|29345697|ref|NP_809200.1|	putative biopolymer transmembrane protein	U	LGT-in
gi|29345779|ref|NP_809282.1|	endo-1,4-beta-xylanase D precursor	-	LGT-unresolved
gi|29345802|ref|NP_809305.1|	lipopolysaccharide biosynthesis RfbU-related protein	-	LGT-in
gi|29345804|ref|NP_809307.1|	capsular polysaccharide biosynthesis glycosyltransferase	M	LGT-unresolved
gi|29345844|ref|NP_809347.1|	hypothetical protein BT0434	-	LGT-unresolved
gi|29345847|ref|NP_809350.1|	N-acylglucosamine 2-epimerase	-	LGT-unresolved
gi|29345852|ref|NP_809355.1|	glycerophosphoryl diester phosphodiesterase	-	LGT-in
gi|29345863|ref|NP_809366.1|	N-acylglucosamine 2-epimerase	G	LGT-unresolved
gi|29345864|ref|NP_809367.1|	arabinose-proton symporter (Arabinose transporter)	GEPR	LGT-in
gi|29345882|ref|NP_809385.1|	putative acyltransferase in colanic acid biosynthesis	R	LGT-unresolved
gi|29345884|ref|NP_809387.1|	D-glycero-D-manno-heptose 1-phosphate kinase	R	LGT-unresolved
gi|29345885|ref|NP_809388.1|	putative phosphoheptose isomerase	G	LGT-unresolved
gi|29345924|ref|NP_809427.1|	hypothetical protein BT0514	-	LGT-in
gi|29345925|ref|NP_809428.1|	terminal quinol oxidase, subunit, putative (doxD-like)	-	LGT-out
gi|29346012|ref|NP_809515.1|	UDP-N-acetyl-D-mannosaminuronic acid dehydrogenase	M	LGT-in
gi|29346017|ref|NP_809520.1|	serine O-acetyltransferase	-	LGT-unresolved
gi|29346140|ref|NP_809643.1|	putative mRNA-binding protein	MG	LGT-in
gi|29346168|ref|NP_809671.1|	acetyltransferase	R	LGT-unresolved
gi|29346175|ref|NP_809678.1|	putative protease	O	LGT-unresolved
gi|29346246|ref|NP_809749.1|	ATP-dependent protease	O	LGT-in
gi|29346247|ref|NP_809750.1|	ATP-dependent protease	O	LGT-in
gi|29346334|ref|NP_809837.1|	hypothetical protein BT0924	S	LGT-in
gi|29346335|ref|NP_809838.1|	ABC transporter, ATP-binding protein	V	LGT-in
gi|29346336|ref|NP_809839.1|	ABC-type transport, permease protein	R	LGT-unresolved

gi|29346352|ref|NP_809855.1|	putative dinitrogenase reductase activating glycohydrolase	O	LGT-unresolved
gi|29346381|ref|NP_809884.1|	hypothetical protein BT0971	-	LGT-in
gi|29346382|ref|NP_809885.1|	putative oxidoreductase	IQR	LGT-in
gi|29346401|ref|NP_809904.1|	hypothetical protein BT0991	S	LGT-in
gi|29346443|ref|NP_809946.1|	hypothetical protein BT1033	G	LGT-unresolved
gi|29346451|ref|NP_809954.1|	integrase	-	LGT-unresolved
gi|29346503|ref|NP_810006.1|	conserved hypothetical protein, putative TonB-dependent outer membrane receptor protein	-	LGT-in
gi|29346506|ref|NP_810009.1|	transposase	-	LGT-unresolved
gi|29346523|ref|NP_810026.1|	flavodoxin	-	LGT-in
gi|29346526|ref|NP_810029.1|	hypothetical protein BT1116	R	LGT-in
gi|29346551|ref|NP_810054.1|	cation efflux family protein	P	LGT-unresolved
gi|29346581|ref|NP_810084.1|	hypothetical protein BT1171	-	LGT-in
gi|29346582|ref|NP_810085.1|	DNA primase/helicase	-	LGT-out
gi|29346597|ref|NP_810100.1|	transcriptional regulator	-	LGT-in
gi|29346628|ref|NP_810131.1|	conserved hypothetical protein, with conserved domain	S	LGT-in
gi|29346656|ref|NP_810159.1|	transposase	-	LGT-in
gi|29346666|ref|NP_810169.1|	hypothetical protein BT1256	-	LGT-in
gi|29346667|ref|NP_810170.1|	hypothetical protein BT1257	-	LGT-unresolved
gi|29346671|ref|NP_810174.1|	peptidase	E	LGT-unresolved
gi|29346695|ref|NP_810198.1|	endo-beta-N-acetylglucosaminidase	-	LGT-unresolved
gi|29346720|ref|NP_810223.1|	hypothetical protein BT1310	-	LGT-unresolved
gi|29346755|ref|NP_810258.1|	glycosyltransferase	-	LGT-unresolved
gi|29346758|ref|NP_810261.1|	CDP-abequose synthase	MG	LGT-unresolved
gi|29346793|ref|NP_810296.1|	oxidoreductase, aldo/keto reductase	R	LGT-unresolved
gi|29346794|ref|NP_810297.1|	hypothetical protein BT1384	-	LGT-out
gi|29346797|ref|NP_810300.1|	putative Na+-driven multidrug efflux pump	V	LGT-unresolved
gi|29346800|ref|NP_810303.1|	hypothetical protein BT1390	-	LGT-in
gi|29346804|ref|NP_810307.1|	hypothetical protein BT1394	-	LGT-unresolved
gi|29346807|ref|NP_810310.1|	hypothetical protein BT1397	GER	LGT-unresolved
gi|29346811|ref|NP_810314.1|	putative sugar transport protein	G	LGT-in
gi|29346815|ref|NP_810318.1|	hypothetical protein BT1405	-	LGT-unresolved
gi|29346817|ref|NP_810320.1|	activator of (R)-2-hydroxyglutaryl-CoA dehydratase	SSI	LGT-in
gi|29346819|ref|NP_810322.1|	hypothetical protein BT1409	-	LGT-out
gi|29346832|ref|NP_810335.1|	hypothetical protein BT1422	-	LGT-in
gi|29346833|ref|NP_810336.1|	hypothetical protein BT1423	S	LGT-in
gi|29346836|ref|NP_810339.1|	hypothetical protein BT1426	S	LGT-in
gi|29346846|ref|NP_810349.1|	hypothetical protein BT1436	-	LGT-in
gi|29346848|ref|NP_810351.1|	putative membrane efflux protein	GEPR	LGT-in
gi|29346873|ref|NP_810376.1|	putative acetyltransferase	R	LGT-in
gi|29346883|ref|NP_810386.1|	hypothetical protein BT1473	R	LGT-in
gi|29346884|ref|NP_810387.1|	ribosomal protein S6 modification protein-related protein	HJ	LGT-in
gi|29346932|ref|NP_810435.1|	putative aureobasidin A resistance protein	-	LGT-in
gi|29346933|ref|NP_810436.1|	CDP-diacylglycerol-inositol 3-phosphatidyltransferase	I	LGT-in
gi|29346935|ref|NP_810438.1|	phosphatidylglycerophosphatase A	I	LGT-in
gi|29346992|ref|NP_810495.1|	putative methyltransferase	-	LGT-in
gi|29346997|ref|NP_810500.1|	hypothetical protein BT1587	-	LGT-unresolved
gi|29347012|ref|NP_810515.1|	transposase	-	LGT-unresolved
gi|29347039|ref|NP_810542.1|	hypothetical protein BT1629	-	LGT-in
gi|29347042|ref|NP_810545.1|	chitinase	-	LGT-unresolved
gi|29347059|ref|NP_810562.1|	hypothetical protein BT1649	-	LGT-in
gi|29347126|ref|NP_810629.1|	putative teichoic acid biosynthesis protein B precursor	M	LGT-in
gi|29347176|ref|NP_810679.1|	putative ribonucleoprotein-related protein	-	LGT-unresolved
gi|29347177|ref|NP_810680.1|	hypothetical protein BT1767	-	LGT-unresolved
gi|29347186|ref|NP_810689.1|	beta-galactosidase	G	LGT-unresolved

gi|29347190|ref|NP_810693.1|	periplasmic beta-glucosidase precursor	G	LGT-in
gi|29347191|ref|NP_810694.1|	xylosidase/arabinosidase	-	LGT-in
gi|29347217|ref|NP_810720.1|	conserved protein, with rhomboid family domain	R	LGT-in
gi|29347218|ref|NP_810721.1|	hypothetical protein BT1808	-	LGT-in
gi|29347219|ref|NP_810722.1|	conserved protein, with weak BamHI domain	-	LGT-unresolved
gi|29347230|ref|NP_810733.1|	pyruvate dehydrogenase	EH	LGT-in
gi|29347232|ref|NP_810735.1|	pyruvate dehydrogenase [cytochrome]	EH	LGT-in
gi|29347233|ref|NP_810736.1|	conserved hypothetical protein, putative membrane protein	-	LGT-in
gi|29347235|ref|NP_810738.1|	hypothetical protein BT1825	-	LGT-in
gi|29347317|ref|NP_810820.1|	putative RNA polymerase sigma factor RpoS	-	LGT-in
gi|29347343|ref|NP_810846.1|	transposase	-	LGT-in
gi|29347366|ref|NP_810869.1|	putative cell surface protein	-	LGT-out
gi|29347394|ref|NP_810897.1|	transcriptional regulator	K	LGT-unresolved
gi|29347404|ref|NP_810907.1|	hypothetical protein BT1994	-	LGT-in
gi|29347406|ref|NP_810909.1|	transposase	L	LGT-out
gi|29347436|ref|NP_810939.1|	endonuclease	-	LGT-in
gi|29347473|ref|NP_810976.1|	conserved hypothetical protein, putative iron uptake factor	S	LGT-unresolved
gi|29347513|ref|NP_811016.1|	transcriptional regulator	K	LGT-in
gi|29347515|ref|NP_811018.1|	putative alpha-1,2-mannosidase	G	LGT-in
gi|29347517|ref|NP_811020.1|	putative outer membrane protein, probably involved in nutrient binding	-	LGT-out
gi|29347523|ref|NP_811026.1|	hypothetical protein BT2113	-	LGT-in
gi|29347558|ref|NP_811061.1|	glycosyltransferase	-	LGT-unresolved
gi|29347577|ref|NP_811080.1|	elongation factor G	J	LGT-in
gi|29347584|ref|NP_811087.1|	ABC transporter ATP-binding protein	R	LGT-in
gi|29347600|ref|NP_811103.1|	beta-galactosidase	G	LGT-unresolved
gi|29347653|ref|NP_811156.1|	hypothetical protein BT2243	-	LGT-in
gi|29347740|ref|NP_811243.1|	hypothetical protein BT2330	-	LGT-in
gi|29347749|ref|NP_811252.1|	hypothetical protein BT2339	-	LGT-in
gi|29347750|ref|NP_811253.1|	hypothetical protein BT2340	-	LGT-in
gi|29347751|ref|NP_811254.1|	hypothetical protein BT2341	-	LGT-in
gi|29347752|ref|NP_811255.1|	transcription regulator	-	LGT-unresolved
gi|29347755|ref|NP_811258.1|	hypothetical protein BT2345	-	LGT-in
gi|29347756|ref|NP_811259.1|	iron-sulfur cluster-binding protein	-	LGT-in
gi|29347763|ref|NP_811266.1|	hypothetical protein BT2353	-	LGT-in
gi|29347765|ref|NP_811268.1|	site-specific DNA-methyltransferase	L	LGT-unresolved
gi|29347781|ref|NP_811284.1|	putative protease/amidase	R	LGT-in
gi|29347789|ref|NP_811292.1|	hypothetical protein BT2379	-	LGT-in
gi|29347791|ref|NP_811294.1|	putative acetyltransferase	-	LGT-in
gi|29347795|ref|NP_811298.1|	hypothetical protein BT2385	-	LGT-unresolved
gi|29347841|ref|NP_811344.1|	hypothetical protein BT2431	-	LGT-in
gi|29347865|ref|NP_811368.1|	reverse transcriptase/maturase family protein	L	LGT-in
gi|29347866|ref|NP_811369.1|	putative reverse transcriptase/maturase	L	LGT-in
gi|29347868|ref|NP_811371.1|	putative pyridine nucleotide-disulphide oxidoreductase	-	LGT-in
gi|29347892|ref|NP_811395.1|	transcriptional regulator	K	LGT-in
gi|29347912|ref|NP_811415.1|	hypothetical protein BT2502	-	LGT-in
gi|29347918|ref|NP_811421.1|	hypothetical protein BT2508	-	LGT-unresolved
gi|29347968|ref|NP_811471.1|	transposase	-	LGT-unresolved
gi|29347989|ref|NP_811492.1|	hypothetical protein BT2579	-	LGT-in
gi|29348000|ref|NP_811503.1|	hypothetical protein BT2590	-	LGT-out
gi|29348025|ref|NP_811528.1|	reverse transcriptase	L	LGT-in
gi|29348027|ref|NP_811530.1|	reverse transcriptase	L	LGT-unresolved
gi|29348031|ref|NP_811534.1|	hypothetical protein BT2621	-	LGT-unresolved
gi|29348037|ref|NP_811540.1|	putative cell surface protein, have conserved domain	-	LGT-in
gi|29348044|ref|NP_811547.1|	putative integrase/recombinase	L	LGT-in

gi|29348045|ref|NP_811548.1|	putative integrase/recombinase	-	LGT-in
gi|29348046|ref|NP_811549.1|	hypothetical protein BT2636	-	LGT-in
gi|29348049|ref|NP_811552.1|	hypothetical protein BT2639	-	LGT-out
gi|29348163|ref|NP_811666.1|	hypothetical protein BT2754	-	LGT-unresolved
gi|29348172|ref|NP_811675.1|	transposase	L	LGT-out
gi|29348180|ref|NP_811683.1|	putative serine/threonine-protein kinase pknB	RTKL	LGT-in
gi|29348181|ref|NP_811684.1|	putative protein phosphatase	T	LGT-unresolved
gi|29348182|ref|NP_811685.1|	hypothetical protein BT2773	-	LGT-unresolved
gi|29348183|ref|NP_811686.1|	putative adenylate cyclase	T	LGT-unresolved
gi|29348204|ref|NP_811707.1|	outer membrane efflux protein precursor	MU	LGT-unresolved
gi|29348217|ref|NP_811720.1|	putative inosine-uridine preferring nucleoside hydrolase	-	LGT-unresolved
gi|29348261|ref|NP_811764.1|	putative beta-xylosidase	G	LGT-out
gi|29348268|ref|NP_811771.1|	putative outer membrane protein, probably involved in nutrient binding	-	LGT-out
gi|29348273|ref|NP_811776.1|	putative glycosyltransferase involved in lipopolysaccharide biosynthesis	M	LGT-unresolved
gi|29348274|ref|NP_811777.1|	putative glucose transferase	M	LGT-unresolved
gi|29348281|ref|NP_811784.1|	putative capsular polysaccharide synthesis protein	-	LGT-unresolved
gi|29348292|ref|NP_811795.1|	phosphoribosylglycinamide formyltransferase	F	LGT-unresolved
gi|29348293|ref|NP_811796.1|	hypothetical protein BT2884	-	LGT-in
gi|29348301|ref|NP_811804.1|	hypothetical protein BT2892	-	LGT-unresolved
gi|29348314|ref|NP_811817.1|	putative outer membrane protein, probably involved in nutrient binding	-	LGT-out
gi|29348318|ref|NP_811821.1|	putative L-arabinose isomerase	-	LGT-unresolved
gi|29348329|ref|NP_811832.1|	putative outer membrane protein, probably involved in nutrient binding	-	LGT-out
gi|29348330|ref|NP_811833.1|	hypothetical protein BT2921	R	LGT-out
gi|29348352|ref|NP_811855.1|	putative hexapeptide transferase family protein	R	LGT-in
gi|29348363|ref|NP_811866.1|	hypothetical protein BT2954	-	LGT-in
gi|29348366|ref|NP_811869.1|	transcriptional regulator	-	LGT-unresolved
gi|29348373|ref|NP_811876.1|	chondroitinase AC precursor	-	LGT-unresolved
gi|29348377|ref|NP_811880.1|	putative outer membrane protein, probably involved in nutrient binding	-	LGT-out
gi|29348401|ref|NP_811904.1|	hypothetical protein BT2992	R	LGT-in
gi|29348408|ref|NP_811911.1|	helicase IV	L	LGT-unresolved
gi|29348410|ref|NP_811913.1|	ATP-dependent DNA helicase	-	LGT-unresolved
gi|29348413|ref|NP_811916.1|	hypothetical protein BT3004	-	LGT-in
gi|29348416|ref|NP_811919.1|	D-alanyl-D-alanine dipeptidase	M	LGT-out
gi|29348449|ref|NP_811952.1|	hypothetical protein BT3040	R	LGT-unresolved
gi|29348490|ref|NP_811993.1|	putative pectin degradation protein	S	LGT-in
gi|29348496|ref|NP_811999.1|	cycloisomaltooligosaccharide glucanotransferase	-	LGT-in
gi|29348501|ref|NP_812004.1|	beta-galactosidase	G	LGT-unresolved
gi|29348503|ref|NP_812006.1|	putative secreted xylosidase	-	LGT-unresolved
gi|29348509|ref|NP_812012.1|	lipase, putative esterase	-	LGT-unresolved
gi|29348510|ref|NP_812013.1|	N-sulphoglucosamine sulphohydrolase precursor	P	LGT-in
gi|29348515|ref|NP_812018.1|	arylsulfatase (aryl-sulfate sulphohydrolase)	P	LGT-unresolved
gi|29348555|ref|NP_812058.1|	hypothetical protein BT3146	-	LGT-in
gi|29348561|ref|NP_812064.1|	ATP-dependent DNA helicase	K	LGT-unresolved
gi|29348565|ref|NP_812068.1|	putative outer membrane protein, probably involved in nutrient binding	-	LGT-out
gi|29348569|ref|NP_812072.1|	hypothetical protein BT3160	-	LGT-out
gi|29348570|ref|NP_812073.1|	hypothetical protein BT3161	-	LGT-in
gi|29348579|ref|NP_812082.1|	hypothetical protein BT3170	-	LGT-in
gi|29348585|ref|NP_812088.1|	hypothetical protein BT3176	-	LGT-in

<table>
<tr><td>gi|29348610|ref|NP_812113.1|</td><td>hypothetical protein BT3201</td><td>-</td><td>LGT-unresolved</td></tr>
<tr><td>gi|29348611|ref|NP_812114.1|</td><td>cell well associated RhsD protein precursor</td><td>-</td><td>LGT-unresolved</td></tr>
<tr><td>gi|29348624|ref|NP_812127.1|</td><td>hypothetical protein BT3215</td><td>-</td><td>LGT-in</td></tr>
<tr><td>gi|29348773|ref|NP_812276.1|</td><td>hypothetical protein BT3364</td><td>-</td><td>LGT-in</td></tr>
<tr><td>gi|29348775|ref|NP_812278.1|</td><td>putative glycosyltransferase yibD</td><td>M</td><td>LGT-unresolved</td></tr>
<tr><td>gi|29348776|ref|NP_812279.1|</td><td>putative glycosyltransferase</td><td>-</td><td>LGT-unresolved</td></tr>
<tr><td>gi|29348777|ref|NP_812280.1|</td><td>putative glycosyltransferase</td><td>-</td><td>LGT-unresolved</td></tr>
<tr><td>gi|29348782|ref|NP_812285.1|</td><td>spermidine n1-acetyltransferase</td><td>-</td><td>LGT-unresolved</td></tr>
<tr><td>gi|29348788|ref|NP_812291.1|</td><td>putative glycosyltransferase</td><td>M</td><td>LGT-in</td></tr>
<tr><td>gi|29348877|ref|NP_812380.1|</td><td>hypothetical protein BT3468</td><td>H</td><td>LGT-unresolved</td></tr>
<tr><td>gi|29348880|ref|NP_812383.1|</td><td>hypothetical protein BT3471</td><td>-</td><td>LGT-unresolved</td></tr>
<tr><td>gi|29348894|ref|NP_812397.1|</td><td>hypothetical protein BT3485</td><td>-</td><td>LGT-unresolved</td></tr>
<tr><td>gi|29348895|ref|NP_812398.1|</td><td>N-acetylgalactosamine-6-sulfatase precursor</td><td>P</td><td>LGT-unresolved</td></tr>
<tr><td>gi|29348906|ref|NP_812409.1|</td><td>hypothetical protein BT3497</td><td>-</td><td>LGT-in</td></tr>
<tr><td>gi|29348908|ref|NP_812411.1|</td><td>hypothetical protein BT3499</td><td>-</td><td>LGT-unresolved</td></tr>
<tr><td>gi|29348924|ref|NP_812427.1|</td><td>putatuve glycosylhydrolase</td><td>R</td><td>LGT-unresolved</td></tr>
<tr><td>gi|29348938|ref|NP_812441.1|</td><td>aldose 1-epimerase precursor</td><td>G</td><td>LGT-unresolved</td></tr>
<tr><td>gi|29348944|ref|NP_812447.1|</td><td>hypothetical protein BT3535</td><td>-</td><td>LGT-in</td></tr>
<tr><td>gi|29348956|ref|NP_812459.1|</td><td>hypothetical protein BT3547</td><td>-</td><td>LGT-unresolved</td></tr>
<tr><td>gi|29348970|ref|NP_812473.1|</td><td>hypothetical protein BT3561</td><td>-</td><td>LGT-unresolved</td></tr>
<tr><td>gi|29348988|ref|NP_812491.1|</td><td>DNA topoisomerase IV subunit A</td><td>L</td><td>LGT-in</td></tr>
<tr><td>gi|29348990|ref|NP_812493.1|</td><td>hypothetical protein BT3582</td><td>-</td><td>LGT-unresolved</td></tr>
<tr><td>gi|29348991|ref|NP_812494.1|</td><td>putative dehydrogenase and relate proteins</td><td>R</td><td>LGT-in</td></tr>
<tr><td>gi|29348992|ref|NP_812495.1|</td><td>thiamine biosynthesis lipoprotein ApbE</td><td>H</td><td>LGT-in</td></tr>
<tr><td>gi|29348993|ref|NP_812496.1|</td><td>putative oxidoreductase</td><td>-</td><td>LGT-unresolved</td></tr>
<tr><td>gi|29348994|ref|NP_812497.1|</td><td>putative dehydrogenase</td><td>-</td><td>LGT-unresolved</td></tr>
<tr><td>gi|29349000|ref|NP_812503.1|</td><td>alpha-galactosidase precursor</td><td>-</td><td>LGT-unresolved</td></tr>
<tr><td>gi|29349001|ref|NP_812504.1|</td><td>hypothetical protein BT3593</td><td>-</td><td>LGT-in</td></tr>
<tr><td>gi|29349008|ref|NP_812511.1|</td><td>ROK family transcriptional repressor</td><td>KG</td><td>LGT-in</td></tr>
<tr><td>gi|29349012|ref|NP_812515.1|</td><td>putative outer membrane protein, probably involved in nutrient binding</td><td>-</td><td>LGT-out</td></tr>
<tr><td>gi|29349069|ref|NP_812572.1|</td><td>alpha-glucosidase</td><td>-</td><td>LGT-in</td></tr>
<tr><td>gi|29349076|ref|NP_812579.1|</td><td>hypothetical protein BT3668</td><td>-</td><td>LGT-in</td></tr>
<tr><td>gi|29349082|ref|NP_812585.1|</td><td>hypothetical protein BT3674</td><td>S</td><td>LGT-unresolved</td></tr>
<tr><td>gi|29349084|ref|NP_812587.1|</td><td>hypothetical protein BT3676</td><td>-</td><td>LGT-unresolved</td></tr>
<tr><td>gi|29349127|ref|NP_812630.1|</td><td>gamma-glutamyl kinase</td><td>E</td><td>LGT-unresolved</td></tr>
<tr><td>gi|29349141|ref|NP_812644.1|</td><td>argininosuccinate lyase</td><td>E</td><td>LGT-in</td></tr>
<tr><td>gi|29349183|ref|NP_812686.1|</td><td>hypothetical protein BT3775</td><td>-</td><td>LGT-unresolved</td></tr>
<tr><td>gi|29349218|ref|NP_812721.1|</td><td>putative endothelin-converting enzyme</td><td>O</td><td>LGT-unresolved</td></tr>
<tr><td>gi|29349269|ref|NP_812772.1|</td><td>hypothetical protein BT3861</td><td>-</td><td>LGT-unresolved</td></tr>
<tr><td>gi|29349287|ref|NP_812790.1|</td><td>putative translation initiation factor SUI1</td><td>J</td><td>LGT-in</td></tr>
<tr><td>gi|29349313|ref|NP_812816.1|</td><td>putative alkaline protease aprF</td><td>MU</td><td>LGT-unresolved</td></tr>
<tr><td>gi|29349314|ref|NP_812817.1|</td><td>hypothetical protein BT3906</td><td>-</td><td>LGT-in</td></tr>
<tr><td>gi|29349315|ref|NP_812818.1|</td><td>hypothetical protein BT3907</td><td>-</td><td>LGT-in</td></tr>
<tr><td>gi|29349316|ref|NP_812819.1|</td><td>hypothetical protein BT3908</td><td>R</td><td>LGT-in</td></tr>
<tr><td>gi|29349317|ref|NP_812820.1|</td><td>hypothetical protein BT3909</td><td>-</td><td>LGT-unresolved</td></tr>
<tr><td>gi|29349367|ref|NP_812870.1|</td><td>conserved hypothetical protein, putative outer membrane protein</td><td>-</td><td>LGT-unresolved</td></tr>
<tr><td>gi|29349394|ref|NP_812897.1|</td><td>putative patatin-like protein</td><td>-</td><td>LGT-out</td></tr>
<tr><td>gi|29349417|ref|NP_812920.1|</td><td>integrase</td><td>-</td><td>LGT-in</td></tr>
<tr><td>gi|29349418|ref|NP_812921.1|</td><td>hypothetical protein BT4010</td><td>-</td><td>LGT-unresolved</td></tr>
<tr><td>gi|29349419|ref|NP_812922.1|</td><td>DNA-methyltransferase</td><td>L</td><td>LGT-in</td></tr>
<tr><td>gi|29349443|ref|NP_812946.1|</td><td>hypothetical protein BT4035</td><td>-</td><td>LGT-in</td></tr>
<tr><td>gi|29349457|ref|NP_812960.1|</td><td>arginine/ornithine transport system ATPase</td><td>E</td><td>LGT-out</td></tr>
</table>

<table>
<tr><td>gi|29349486|ref|NP_812989.1|</td><td>hypothetical protein BT4078</td><td>-</td><td>LGT-in</td></tr>
<tr><td>gi|29349522|ref|NP_813025.1|</td><td>putative outer membrane protein, probably involved in nutrient binding</td><td>-</td><td>LGT-unresolved</td></tr>
<tr><td>gi|29349542|ref|NP_813045.1|</td><td>putative outer membrane protein, probably involved in nutrient binding</td><td>-</td><td>LGT-out</td></tr>
<tr><td>gi|29349551|ref|NP_813054.1|</td><td>putative iron-sulfur cluster binding protein</td><td>C</td><td>LGT-unresolved</td></tr>
<tr><td>gi|29349556|ref|NP_813059.1|</td><td>hypothetical protein BT4148</td><td>-</td><td>LGT-unresolved</td></tr>
<tr><td>gi|29349561|ref|NP_813064.1|</td><td>Exopolygalacturonase precursor</td><td>M</td><td>LGT-unresolved</td></tr>
<tr><td>gi|29349570|ref|NP_813073.1|</td><td>hypothetical protein BT4162</td><td>R</td><td>LGT-in</td></tr>
<tr><td>gi|29349571|ref|NP_813074.1|</td><td>hypothetical protein BT4163</td><td>-</td><td>LGT-unresolved</td></tr>
<tr><td>gi|29349572|ref|NP_813075.1|</td><td>putative outer membrane protein, probably involved in nutrient binding</td><td>-</td><td>LGT-unresolved</td></tr>
<tr><td>gi|29349573|ref|NP_813076.1|</td><td>putative outer membrane protein, probably involved in nutrient binding</td><td>-</td><td>LGT-unresolved</td></tr>
<tr><td>gi|29349575|ref|NP_813078.1|</td><td>hypothetical protein BT4167</td><td>-</td><td>LGT-unresolved</td></tr>
<tr><td>gi|29349576|ref|NP_813079.1|</td><td>putative outer membrane protein, probably involved in nutrient binding</td><td>-</td><td>LGT-unresolved</td></tr>
<tr><td>gi|29349577|ref|NP_813080.1|</td><td>hypothetical protein BT4169</td><td>-</td><td>LGT-in</td></tr>
<tr><td>gi|29349587|ref|NP_813090.1|</td><td>polysaccharide deacetylase</td><td>-</td><td>LGT-in</td></tr>
<tr><td>gi|29349590|ref|NP_813093.1|</td><td>two-component system sensor histidine kinase/response regulator, hybrid ('one-component system')</td><td>TKT</td><td>LGT-unresolved</td></tr>
<tr><td>gi|29349629|ref|NP_813132.1|</td><td>hypothetical protein BT4221</td><td>-</td><td>LGT-unresolved</td></tr>
<tr><td>gi|29349686|ref|NP_813189.1|</td><td>transposase</td><td>L</td><td>LGT-unresolved</td></tr>
<tr><td>gi|29349687|ref|NP_813190.1|</td><td>hypothetical protein BT4279</td><td>-</td><td>LGT-in</td></tr>
<tr><td>gi|29349723|ref|NP_813226.1|</td><td>HPr(Ser) phosphatase</td><td>R</td><td>LGT-unresolved</td></tr>
<tr><td>gi|29349751|ref|NP_813254.1|</td><td>hypothetical protein BT4343</td><td>L</td><td>LGT-in</td></tr>
<tr><td>gi|29349759|ref|NP_813262.1|</td><td>hypothetical protein BT4351</td><td>-</td><td>LGT-in</td></tr>
<tr><td>gi|29349792|ref|NP_813295.1|</td><td>hypothetical protein BT4384</td><td>-</td><td>LGT-unresolved</td></tr>
<tr><td>gi|29349799|ref|NP_813302.1|</td><td>putative metalloendopeptidase</td><td>M</td><td>LGT-unresolved</td></tr>
<tr><td>gi|29349815|ref|NP_813318.1|</td><td>hypothetical protein BT4407</td><td>-</td><td>LGT-in</td></tr>
<tr><td>gi|29349828|ref|NP_813331.1|</td><td>conserved hypothetical protein, with a conserved domain</td><td>-</td><td>LGT-in</td></tr>
<tr><td>gi|29349830|ref|NP_813333.1|</td><td>hypothetical protein BT4422</td><td>-</td><td>LGT-unresolved</td></tr>
<tr><td>gi|29349832|ref|NP_813335.1|</td><td>sorbitol dehydrogenase</td><td>ER</td><td>LGT-unresolved</td></tr>
<tr><td>gi|29349847|ref|NP_813350.1|</td><td>hypothetical protein BT4439</td><td>-</td><td>LGT-in</td></tr>
<tr><td>gi|29349850|ref|NP_813353.1|</td><td>hypothetical protein BT4442</td><td>-</td><td>LGT-unresolved</td></tr>
<tr><td>gi|29349852|ref|NP_813355.1|</td><td>hypothetical protein BT4444</td><td>-</td><td>LGT-unresolved</td></tr>
<tr><td>gi|29349887|ref|NP_813390.1|</td><td>integrase protein</td><td>-</td><td>LGT-unresolved</td></tr>
<tr><td>gi|29349909|ref|NP_813412.1|</td><td>ribosomal large subunit pseudouridine synthase A</td><td>J</td><td>LGT-unresolved</td></tr>
<tr><td>gi|29349915|ref|NP_813418.1|</td><td>beta-lactamase precursor</td><td>-</td><td>LGT-in</td></tr>
<tr><td>gi|29349923|ref|NP_813426.1|</td><td>hypothetical protein BT4515</td><td>-</td><td>LGT-in</td></tr>
<tr><td>gi|29349937|ref|NP_813440.1|</td><td>hypothetical protein BT4529</td><td>-</td><td>LGT-unresolved</td></tr>
<tr><td>gi|29349938|ref|NP_813441.1|</td><td>ATP-dependent DNA helicase recG</td><td>K</td><td>LGT-in</td></tr>
<tr><td>gi|29349940|ref|NP_813443.1|</td><td>hypothetical protein BT4532</td><td>-</td><td>LGT-in</td></tr>
<tr><td>gi|29349943|ref|NP_813446.1|</td><td>Type I restriction enzyme EcoR124II R protein</td><td>V</td><td>LGT-unresolved</td></tr>
<tr><td>gi|29349949|ref|NP_813452.1|</td><td>putative Type I restriction modification DNA specificity protein</td><td>-</td><td>LGT-in</td></tr>
<tr><td>gi|29350009|ref|NP_813512.1|</td><td>hypothetical protein BT4601</td><td>-</td><td>LGT-unresolved</td></tr>
<tr><td>gi|29350014|ref|NP_813517.1|</td><td>hypothetical protein BT4606</td><td>-</td><td>LGT-in</td></tr>
<tr><td>gi|29350059|ref|NP_813562.1|</td><td>endonuclease IV</td><td>L</td><td>LGT-in</td></tr>
<tr><td>gi|29350064|ref|NP_813567.1|</td><td>N-acetylglucosamine-6-sulfatase precursor</td><td>P</td><td>LGT-out</td></tr>
<tr><td>gi|29350082|ref|NP_813585.1|</td><td>hypothetical protein BT4674</td><td>-</td><td>LGT-in</td></tr>
<tr><td>gi|29350124|ref|NP_813627.1|</td><td>redox-sensitive transcriptional activator</td><td>K</td><td>LGT-unresolved</td></tr>
<tr><td>gi|29350135|ref|NP_813638.1|</td><td>glycerophosphoryl diester phosphodiesterase</td><td>C</td><td>LGT-in</td></tr>
</table>

gi|29350152|ref|NP_813655.1|	putative multiple inositol polyphosphate histidine phosphatase 1	-	LGT-in
gi|29350160|ref|NP_813663.1|	hypothetical protein BT4752	-	LGT-in
gi|29350161|ref|NP_813664.1|	hypothetical protein BT4753	-	LGT-unresolved

(D). Putative xenologs in *B. fragilis* NCTC 9343

Gene	Annotation	COG	LGT
gi|60679615|ref|YP_209759.1|	putative iron transport receptor protein	P	LGT-in
gi|60679717|ref|YP_209861.1|	hypothetical protein BF0121	-	LGT-unresolved
gi|60679762|ref|YP_209906.1|	hypothetical Kelch-like protein	-	LGT-unresolved
gi|60679781|ref|YP_209925.1|	putative glycosyltransferase	M	LGT-unresolved
gi|60679788|ref|YP_209932.1|	hypothetical protein BF0193	R	LGT-in
gi|60679789|ref|YP_209933.1|	putative heat-shock protein	O	LGT-unresolved
gi|60679790|ref|YP_209934.1|	hypothetical protein BF0195	-	LGT-unresolved
gi|60679830|ref|YP_209974.1|	hypothetical protein BF0237	-	LGT-in
gi|60679881|ref|YP_210025.1|	putative TonB dependent receptor outer membrane protein	-	LGT-out
gi|60679882|ref|YP_210026.1|	putative lipoprotein	-	LGT-out
gi|60679926|ref|YP_210070.1|	hypothetical protein BF0337	-	LGT-unresolved
gi|60679954|ref|YP_210098.1|	putative beta-lactamase/acylhydrolase	V	LGT-in
gi|60679958|ref|YP_210102.1|	putative FAD-binding oxidoreductase	CC	LGT-unresolved
gi|60680035|ref|YP_210179.1|	putative bacteriophage resolvase/recombinase	L	LGT-in
gi|60680066|ref|YP_210210.1|	putative mismatch repair-like protein	L	LGT-in
gi|60680142|ref|YP_210286.1|	putative glycosyl hydrolase	G	LGT-unresolved
gi|60680186|ref|YP_210330.1|	putative chaperone protein	O	LGT-unresolved
gi|60680246|ref|YP_210390.1|	putative efflux transporter bicyclomycin resistance protein	GEPR	LGT-unresolved
gi|60680281|ref|YP_210425.1|	possible HipA-like protein	-	LGT-unresolved
gi|60680292|ref|YP_210436.1|	putative sugar-phosphate nucleotidyl transferase	MJ	LGT-in
gi|60680298|ref|YP_210442.1|	possible capsular polysaccharide related protein	-	LGT-unresolved
gi|60680301|ref|YP_210445.1|	putative transposase_11 DDE family protein	-	LGT-unresolved
gi|60680302|ref|YP_210446.1|	hypothetical protein BF0747	-	LGT-unresolved
gi|60680303|ref|YP_210447.1|	putative glycosyltransferase protein	M	LGT-unresolved
gi|60680305|ref|YP_210449.1|	putative acetyltransferase	R	LGT-in
gi|60680306|ref|YP_210450.1|	putative FIC family protein	D	LGT-in
gi|60680321|ref|YP_210465.1|	possible methyltransferase	-	LGT-in
gi|60680322|ref|YP_210466.1|	putative B12-binding methyltransferase	R	LGT-in
gi|60680333|ref|YP_210477.1|	hypothetical protein BF0779	-	LGT-unresolved
gi|60680351|ref|YP_210495.1|	hypothetical protein BF0799	R	LGT-unresolved
gi|60680406|ref|YP_210550.1|	hypothetical protein BF0855	-	LGT-unresolved
gi|60680461|ref|YP_210605.1|	putative O-acetylhomoserine synthase	E	LGT-unresolved
gi|60680566|ref|YP_210710.1|	putative glycosyltransferase	M	LGT-unresolved
gi|60680568|ref|YP_210712.1|	putative epimerase/dehydratase	MG	LGT-in
gi|60680590|ref|YP_210734.1|	putative 3,4-dihydroxy-2-butanone 4-phosphate/shikimate 5-dehydrogenase fusion protein	HE	LGT-unresolved
gi|60680593|ref|YP_210737.1|	hypothetical protein BF1054	-	LGT-unresolved
gi|60680596|ref|YP_210740.1|	putative restriction enzyme of type III restriction-modification system	V	LGT-unresolved
gi|60680597|ref|YP_210741.1|	putative modification enzyme of type III restriction-modification system	L	LGT-unresolved
gi|60680603|ref|YP_210747.1|	putative ATP/GTP-binding protein	-	LGT-in
gi|60680614|ref|YP_210758.1|	putative modification protein of type I restriction-modification system	V	LGT-out
gi|60680615|ref|YP_210759.1|	putative type I restriction enzyme	V	LGT-out
gi|60680619|ref|YP_210763.1|	hypothetical protein BF1083	-	LGT-in
gi|60680622|ref|YP_210766.1|	hypothetical protein BF1090	K	LGT-in
gi|60680633|ref|YP_210777.1|	conserved hypothetical protein with RecF/RecN/SMC N-terminal domain	L	LGT-in
gi|60680634|ref|YP_210778.1|	putative ATP-dependent DNA helicase	L	LGT-in
gi|60680646|ref|YP_210790.1|	hypothetical protein BF1117	KL	LGT-unresolved
gi|60680647|ref|YP_210791.1|	hypothetical protein BF1118	-	LGT-unresolved

<table>
<tr><td>gi|60680648|ref|YP_210792.1|</td><td>hypothetical protein BF1119</td><td>-</td><td>LGT-in</td></tr>
<tr><td>gi|60680655|ref|YP_210799.1|</td><td>hypothetical protein BF1126</td><td>R</td><td>LGT-in</td></tr>
<tr><td>gi|60680721|ref|YP_210865.1|</td><td>putative alpha-glucosidase, glycosylhydrolase</td><td>G</td><td>LGT-in</td></tr>
<tr><td>gi|60680750|ref|YP_210894.1|</td><td>hypothetical protein BF1230</td><td>-</td><td>LGT-in</td></tr>
<tr><td>gi|60680771|ref|YP_210915.1|</td><td>hypothetical protein BF1251</td><td>-</td><td>LGT-in</td></tr>
<tr><td>gi|60680849|ref|YP_210993.1|</td><td>putative alpha-glucosidase II</td><td>G</td><td>LGT-unresolved</td></tr>
<tr><td>gi|60680872|ref|YP_211016.1|</td><td>possible cell division protein</td><td>-</td><td>LGT-unresolved</td></tr>
<tr><td>gi|60680887|ref|YP_211031.1|</td><td>hypothetical protein BF1373</td><td>-</td><td>LGT-in</td></tr>
<tr><td>gi|60680889|ref|YP_211033.1|</td><td>putative glycosyltransferase</td><td>M</td><td>LGT-unresolved</td></tr>
<tr><td>gi|60680930|ref|YP_211074.1|</td><td>putative histidine decarboxylase</td><td>-</td><td>LGT-unresolved</td></tr>
<tr><td>gi|60680961|ref|YP_211105.1|</td><td>putative type I RM modification enzyme</td><td>V</td><td>LGT-unresolved</td></tr>
<tr><td>gi|60680962|ref|YP_211106.1|</td><td>putative DNA-binding protein</td><td>-</td><td>LGT-unresolved</td></tr>
<tr><td>gi|60680963|ref|YP_211107.1|</td><td>hypothetical protein BF1460</td><td>-</td><td>LGT-unresolved</td></tr>
<tr><td>gi|60680964|ref|YP_211108.1|</td><td>putative type I restriction enzyme specificity protein</td><td>-</td><td>LGT-out</td></tr>
<tr><td>gi|60681037|ref|YP_211181.1|</td><td>hypothetical protein BF1536</td><td>-</td><td>LGT-in</td></tr>
<tr><td>gi|60681062|ref|YP_211206.1|</td><td>putative transmembrane protein</td><td>-</td><td>LGT-in</td></tr>
<tr><td>gi|60681065|ref|YP_211209.1|</td><td>putative DNTP-hexose dehydratase-epimerase</td><td>MG</td><td>LGT-unresolved</td></tr>
<tr><td>gi|60681122|ref|YP_211266.1|</td><td>hypothetical protein BF1626</td><td>Q</td><td>LGT-unresolved</td></tr>
<tr><td>gi|60681226|ref|YP_211370.1|</td><td>hypothetical protein BF1734</td><td>-</td><td>LGT-in</td></tr>
<tr><td>gi|60681232|ref|YP_211376.1|</td><td>hypothetical protein BF1740</td><td>-</td><td>LGT-in</td></tr>
<tr><td>gi|60681238|ref|YP_211382.1|</td><td>hypothetical protein BF1746</td><td>-</td><td>LGT-in</td></tr>
<tr><td>gi|60681243|ref|YP_211387.1|</td><td>hypothetical protein BF1751</td><td>-</td><td>LGT-in</td></tr>
<tr><td>gi|60681246|ref|YP_211390.1|</td><td>hypothetical protein BF1754</td><td>-</td><td>LGT-in</td></tr>
<tr><td>gi|60681248|ref|YP_211392.1|</td><td>hypothetical protein BF1756</td><td>-</td><td>LGT-in</td></tr>
<tr><td>gi|60681319|ref|YP_211463.1|</td><td>hypothetical protein BF1827</td><td>-</td><td>LGT-out</td></tr>
<tr><td>gi|60681328|ref|YP_211472.1|</td><td>hypothetical protein BF1837</td><td>S</td><td>LGT-unresolved</td></tr>
<tr><td>gi|60681331|ref|YP_211475.1|</td><td>putative type I restriction endonuclease specificity subunit, partial</td><td>-</td><td>LGT-in</td></tr>
<tr><td>gi|60681334|ref|YP_211478.1|</td><td>putative phage integrase/recombinase</td><td>L</td><td>LGT-out</td></tr>
<tr><td>gi|60681400|ref|YP_211544.1|</td><td>putative LPS biosynthesis related epimerase</td><td>M</td><td>LGT-in</td></tr>
<tr><td>gi|60681414|ref|YP_211558.1|</td><td>putative urea transport protein</td><td>-</td><td>LGT-in</td></tr>
<tr><td>gi|60681417|ref|YP_211561.1|</td><td>putative RumB/ImpB like DNA repair protein</td><td>L</td><td>LGT-unresolved</td></tr>
<tr><td>gi|60681428|ref|YP_211572.1|</td><td>hypothetical protein BF1939</td><td>-</td><td>LGT-unresolved</td></tr>
<tr><td>gi|60681431|ref|YP_211575.1|</td><td>coproporphyrinogen III oxidase</td><td>H</td><td>LGT-unresolved</td></tr>
<tr><td>gi|60681470|ref|YP_211614.1|</td><td>putative MarR-family regulatory protein</td><td>KKR</td><td>LGT-in</td></tr>
<tr><td>gi|60681479|ref|YP_211623.1|</td><td>putative TonB-dependent outer membrane receptor protein</td><td>P</td><td>LGT-unresolved</td></tr>
<tr><td>gi|60681500|ref|YP_211644.1|</td><td>hypothetical protein BF2012</td><td>-</td><td>LGT-in</td></tr>
<tr><td>gi|60681511|ref|YP_211655.1|</td><td>putative ATP-dependent protease</td><td>O</td><td>LGT-in</td></tr>
<tr><td>gi|60681517|ref|YP_211661.1|</td><td>putative ATP-binding protein</td><td>-</td><td>LGT-in</td></tr>
<tr><td>gi|60681518|ref|YP_211662.1|</td><td>putative tautomerase</td><td>-</td><td>LGT-in</td></tr>
<tr><td>gi|60681521|ref|YP_211665.1|</td><td>hypothetical protein BF2035</td><td>-</td><td>LGT-in</td></tr>
<tr><td>gi|60681593|ref|YP_211737.1|</td><td>putative RNA-directed DNA polymerase from retron</td><td>-</td><td>LGT-in</td></tr>
<tr><td>gi|60681604|ref|YP_211748.1|</td><td>putative AraC family transcriptional regulator</td><td>K</td><td>LGT-unresolved</td></tr>
<tr><td>gi|60681613|ref|YP_211757.1|</td><td>putative aldo/keto reductase</td><td>C</td><td>LGT-in</td></tr>
<tr><td>gi|60681614|ref|YP_211758.1|</td><td>putative amidohydrolase</td><td>-</td><td>LGT-unresolved</td></tr>
<tr><td>gi|60681615|ref|YP_211759.1|</td><td>short chain dehydrogenase</td><td>IQR</td><td>LGT-unresolved</td></tr>
<tr><td>gi|60681620|ref|YP_211764.1|</td><td>putative acetyltransferase</td><td>R</td><td>LGT-in</td></tr>
<tr><td>gi|60681625|ref|YP_211769.1|</td><td>putative RibD-family protein</td><td>H</td><td>LGT-unresolved</td></tr>
<tr><td>gi|60681654|ref|YP_211798.1|</td><td>hypothetical protein BF2175</td><td>-</td><td>LGT-in</td></tr>
<tr><td>gi|60681768|ref|YP_211912.1|</td><td>hypothetical protein BF2290</td><td>-</td><td>LGT-in</td></tr>
<tr><td>gi|60681777|ref|YP_211921.1|</td><td>hypothetical protein BF2299</td><td>-</td><td>LGT-in</td></tr>
<tr><td>gi|60681805|ref|YP_211949.1|</td><td>putative lipoprotein</td><td>-</td><td>LGT-in</td></tr>
<tr><td>gi|60681818|ref|YP_211962.1|</td><td>putative oxidoreductase</td><td>R</td><td>LGT-unresolved</td></tr>
<tr><td>gi|60681820|ref|YP_211964.1|</td><td>putative fosmidomycin resistance protein</td><td>GEPR</td><td>LGT-unresolved</td></tr>
<tr><td>gi|60681898|ref|YP_212042.1|</td><td>putative proteinase</td><td>OU</td><td>LGT-unresolved</td></tr>
</table>

gi|60681969|ref|YP_212113.1|	hypothetical protein BF2489	S	LGT-unresolved
gi|60682023|ref|YP_212167.1|	putative cobalamin biosynthesis-related membrane protein	H	LGT-in
gi|60682031|ref|YP_212175.1|	putative lipoprotein	-	LGT-in
gi|60682071|ref|YP_212215.1|	putative LPS biosynthesis related DNTP-hexose dehydratase-epimerase	MG	LGT-unresolved
gi|60682072|ref|YP_212216.1|	putative LPS biosynthesis related glycosyltransferase	M	LGT-unresolved
gi|60682073|ref|YP_212217.1|	putative LPS biosynthesis related glycosyltransferase	M	LGT-unresolved
gi|60682074|ref|YP_212218.1|	putative LPS biosynthesis related glycosyltransferase	M	LGT-unresolved
gi|60682079|ref|YP_212223.1|	DNTP-hexose dehydratase-epimerase	MG	LGT-unresolved
gi|60682080|ref|YP_212224.1|	putative LPS biosynthesis related DNTP-hexose dehydratase-epimerase	MG	LGT-unresolved
gi|60682081|ref|YP_212225.1|	putative LPS biosynthesis related DNTP-hexose dehydratase-epimerase	MG	LGT-unresolved
gi|60682083|ref|YP_212227.1|	putative LPS biosynthesis related DNTP-hexose dehydratase-epimerase	M	LGT-in
gi|60682168|ref|YP_212312.1|	putative exported methylamine utilization protein	P	LGT-in
gi|60682169|ref|YP_212313.1|	hypothetical protein BF2696	E	LGT-in
gi|60682173|ref|YP_212317.1|	hypothetical protein BF2700	GEPR	LGT-in
gi|60682174|ref|YP_212318.1|	hypothetical protein BF2701	V	LGT-unresolved
gi|60682197|ref|YP_212341.1|	hypothetical protein BF2724	-	LGT-unresolved
gi|60682198|ref|YP_212342.1|	hypothetical protein BF2725	-	LGT-in
gi|60682199|ref|YP_212343.1|	putative peptide chain release factor	J	LGT-in
gi|60682275|ref|YP_212419.1|	putative glycosyltransferase	M	LGT-unresolved
gi|60682284|ref|YP_212428.1|	putative transmembrane protein	-	LGT-in
gi|60682298|ref|YP_212442.1|	putative phage-related protein	-	LGT-unresolved
gi|60682302|ref|YP_212446.1|	hypothetical protein BF2831	-	LGT-unresolved
gi|60682304|ref|YP_212448.1|	hypothetical protein BF2833	-	LGT-in
gi|60682310|ref|YP_212454.1|	putative two-component system, histidine kinase	T	LGT-unresolved
gi|60682311|ref|YP_212455.1|	hypothetical protein BF2841	-	LGT-in
gi|60682363|ref|YP_212507.1|	hypothetical protein BF2893	SS	LGT-in
gi|60682370|ref|YP_212514.1|	putative exported hydrolase	G	LGT-unresolved
gi|60682373|ref|YP_212517.1|	putative dimethylamine corrinoid protein	R	LGT-in
gi|60682374|ref|YP_212518.1|	putative methyltransferase	-	LGT-unresolved
gi|60682375|ref|YP_212519.1|	putative exported hydrolase	G	LGT-unresolved
gi|60682387|ref|YP_212530.1|	putative restriction enzyme related protein	V	LGT-unresolved
gi|60682388|ref|YP_212532.1|	hypothetical protein BF2918	-	LGT-unresolved
gi|60682400|ref|YP_212544.1|	putative antibiotic resistance transport membrane protein	GEPR	LGT-unresolved
gi|60682424|ref|YP_212568.1|	hypothetical protein BF2954	T	LGT-in
gi|60682444|ref|YP_212588.1|	hypothetical protein BF2974	-	LGT-unresolved
gi|60682448|ref|YP_212592.1|	putative ABC transport system, ATP-binding protein	V	LGT-in
gi|60682449|ref|YP_212593.1|	putative ABC transport system, membrane protein	V	LGT-unresolved
gi|60682490|ref|YP_212634.1|	hypothetical protein BF3020	S	LGT-in
gi|60682519|ref|YP_212663.1|	putative ribonuclease	S	LGT-unresolved
gi|60682520|ref|YP_212664.1|	hypothetical protein BF3050	-	LGT-in
gi|60682543|ref|YP_212687.1|	hypothetical protein BF3074	-	LGT-in
gi|60682561|ref|YP_212705.1|	hypothetical protein BF3092	-	LGT-in
gi|60682584|ref|YP_212728.1|	putative non ribosome peptide synthase related, AMP-binding protein	Q	LGT-in
gi|60682611|ref|YP_212755.1|	hypothetical protein BF3142	-	LGT-in
gi|60682618|ref|YP_212762.1|	putative glycosyl hydrolase	G	LGT-in
gi|60682636|ref|YP_212780.1|	hypothetical protein BF3167	-	LGT-in
gi|60682650|ref|YP_212794.1|	putative lipoprotein	-	LGT-in
gi|60682687|ref|YP_212831.1|	putative flavoprotein	R	LGT-in
gi|60682698|ref|YP_212842.1|	hypothetical protein BF3229	-	LGT-out

gi|60682703|ref|YP 212847.1|	hypothetical protein BF3234	-	LGT-unresolved
gi|60682704|ref|YP_212848.1|	putative glycosyl transferase	M	LGT-in
gi|60682714|ref|YP_212858.1|	hypothetical protein BF3245	M	LGT-unresolved
gi|60682730|ref|YP 212874.1|	hypothetical protein BF3260	M	LGT-unresolved
gi|60682731|ref|YP 212875.1|	putative transport-related, membrane protein	S	LGT-in
gi|60682732|ref|YP_212876.1|	putative glycosyl transferase	M	LGT-unresolved
gi|60682758|ref|YP_212902.1|	putative AraC-family regulatory protein	K	LGT-unresolved
gi|60682761|ref|YP 212905.1|	hypothetical protein BF3293	-	LGT-in
gi|60682764|ref|YP 212908.1|	hypothetical protein BF3296	-	LGT-in
gi|60682786|ref|YP_212930.1|	hypothetical protein BF3318	S	LGT-in
gi|60682807|ref|YP 212951.1|	hypothetical protein BF3340	-	LGT-unresolved
gi|60682808|ref|YP 212952.1|	putative exported beta-lactamase protein	VS	LGT-in
gi|60682810|ref|YP_212954.1|	hypothetical protein BF3345	S	LGT-in
gi|60682819|ref|YP_212963.1|	hypothetical protein BF3354	-	LGT-unresolved
gi|60682824|ref|YP 212968.1|	putative chaperone/membrane protein	P	LGT-unresolved
gi|60682880|ref|YP 213024.1|	putative DNA methylase	L	LGT-unresolved
gi|60682881|ref|YP_213025.1|	hypothetical protein BF3419	-	LGT-in
gi|60682901|ref|YP 213045.1|	hypothetical protein BF3440	-	LGT-in
gi|60682909|ref|YP 213053.1|	hypothetical protein BF3448	-	LGT-unresolved
gi|60682910|ref|YP_213054.1|	cold shock-like protein	K	LGT-in
gi|60682921|ref|YP_213065.1|	putative LPS biosynthesis related glycosyltransferase	-	LGT-in
gi|60682922|ref|YP 213066.1|	hypothetical protein BF3461	-	LGT-in
gi|60682923|ref|YP 213067.1|	hypothetical protein BF3462	-	LGT-unresolved
gi|60682924|ref|YP_213068.1|	putative LPS biosynthesis related polysaccharide transporter	R	LGT-in
gi|60682936|ref|YP 213080.1|	putative acetyltransferase	M	LGT-in
gi|60682938|ref|YP 213082.1|	hypothetical protein BF3478	E	LGT-in
gi|60682973|ref|YP_213117.1|	hypothetical protein BF3515	-	LGT-unresolved
gi|60683011|ref|YP_213155.1|	hypothetical protein BF3553	-	LGT-in
gi|60683086|ref|YP 213230.1|	putative elongation factor G	J	LGT-in
gi|60683129|ref|YP 213273.1|	DNTP-hexose dehydratase-epimerase	MG	LGT-out
gi|60683137|ref|YP_213281.1|	hypothetical protein BF3691	-	LGT-in
gi|60683138|ref|YP_213282.1|	possible flippase	-	LGT-out
gi|60683139|ref|YP 213283.1|	hypothetical protein BF3693	-	LGT-in
gi|60683140|ref|YP_213284.1|	putative nucleotidyltransferase	-	LGT-in
gi|60683142|ref|YP_213286.1|	putative haloacid dehalogenase-like hydrolase	R	LGT-in
gi|60683143|ref|YP 213287.1|	hypothetical protein BF3697	-	LGT-in
gi|60683159|ref|YP_213303.1|	putative GntR-family regulatory protein	K	LGT-in
gi|60683166|ref|YP_213310.1|	putative exported sulfatase	P	LGT-unresolved
gi|60683195|ref|YP_213339.1|	hypothetical protein BF3749	-	LGT-unresolved
gi|60683238|ref|YP 213382.1|	putative exported beta-galactosidase	G	LGT-unresolved
gi|60683245|ref|YP_213389.1|	putative outer membrane protein	-	LGT-unresolved
gi|60683293|ref|YP_213437.1|	hypothetical protein BF3851	M	LGT-unresolved
gi|60683294|ref|YP 213438.1|	putative transport related membrane protein	V	LGT-in
gi|60683295|ref|YP_213439.1|	putative outer membrane protein	-	LGT-in
gi|60683297|ref|YP_213441.1|	putative regulatory protein	-	LGT-in
gi|60683305|ref|YP_213449.1|	putative copper transport-related membrane protein	P	LGT-in
gi|60683316|ref|YP 213460.1|	putative transposase	L	LGT-in
gi|60683320|ref|YP 213464.1|	putative ubiquitin	-	LGT-in
gi|60683338|ref|YP_213482.1|	putative LuxR-family regulatory protein	TK	LGT-in
gi|60683489|ref|YP 213633.1|	putative beta-galactosidase	G	LGT-unresolved
gi|60683516|ref|YP_213660.1|	hypothetical protein BF4092	-	LGT-in
gi|60683526|ref|YP_213670.1|	putative two-component sensor histidine kinase transcriptional regulator	-	LGT-in
gi|60683533|ref|YP_213677.1|	putative thiogalactoside transacetylase	R	LGT-out
gi|60683603|ref|YP 213747.1|	putative transmembrane sugar transporter	GEPR	LGT-unresolved

gi|60683606|ref|YP 213750.1| hypothetical protein BF4185	S	LGT-in
gi|60683616|ref|YP_213760.1| hypothetical protein BF4195	ME	LGT-out
gi|60683622|ref|YP_213766.1| putative glycosyl transferase	M	LGT-in
gi|60683660|ref|YP 213804.1| putative glycerophosphoryl diester phosphodiesterase	C	LGT-unresolved
gi|60683712|ref|YP 213856.1| putative exopolysaccharide biosynthesis protein	-	LGT-unresolved
gi|60683753|ref|YP_213897.1| putative iron-regulated transmembrane protein	S	LGT-in
gi|60683763|ref|YP_213907.1| hypothetical protein BF4352	-	LGT-unresolved

(E). Putative xenologs in *B. fragilis* YCH 46

Gene	Annotation	COG	LGT
gi|53711310|ref|YP_097302.1|	ferrichrome-iron receptor	P	LGT-in
gi|53711426|ref|YP_097418.1|	hypothetical protein BF0135	R	LGT-in
gi|53711430|ref|YP_097422.1|	truncated tetracycline resistance element regulator RteA	T	LGT-in
gi|53711431|ref|YP_097423.1|	truncated tetracycline resistance element regulator RteA	T	LGT-in
gi|53711450|ref|YP_097442.1|	hypothetical protein BF0159	-	LGT-unresolved
gi|53711496|ref|YP_097488.1|	hypothetical protein BF0205	-	LGT-in
gi|53711499|ref|YP_097491.1|	hypothetical protein BF0208	-	LGT-unresolved
gi|53711518|ref|YP_097510.1|	probable glycosyltransferase	M	LGT-unresolved
gi|53711527|ref|YP_097519.1|	hypothetical protein BF0236	R	LGT-in
gi|53711528|ref|YP_097520.1|	heat shock protein HtpG	O	LGT-unresolved
gi|53711529|ref|YP_097521.1|	hypothetical protein BF0238	-	LGT-unresolved
gi|53711569|ref|YP_097561.1|	hypothetical protein BF0278	-	LGT-in
gi|53711575|ref|YP_097567.1|	hypothetical protein BF0284	-	LGT-in
gi|53711631|ref|YP_097623.1|	putative outer membrane protein probably involved in nutrient binding	-	LGT-out
gi|53711632|ref|YP_097624.1|	putative outer membrane protein	-	LGT-out
gi|53711716|ref|YP_097708.1|	putative esterase	V	LGT-in
gi|53711720|ref|YP_097712.1|	putative D-lactate dehydrogenase	CC	LGT-unresolved
gi|53711804|ref|YP_097796.1|	serine type site-specific recombinase	-	LGT-unresolved
gi|53711828|ref|YP_097820.1|	hypothetical protein BF0537	-	LGT-in
gi|53711839|ref|YP_097831.1|	putative mutS-like mismatch repair protein	L	LGT-unresolved
gi|53711911|ref|YP_097903.1|	hypothetical protein BF0620	-	LGT-unresolved
gi|53711915|ref|YP_097907.1|	putative beta-galactosidase	G	LGT-unresolved
gi|53711926|ref|YP_097918.1|	hypothetical protein BF0635	-	LGT-in
gi|53711927|ref|YP_097919.1|	hypothetical protein BF0636	-	LGT-in
gi|53711928|ref|YP_097920.1|	hypothetical protein BF0637	-	LGT-unresolved
gi|53711929|ref|YP_097921.1|	hypothetical protein BF0638	-	LGT-unresolved
gi|53711930|ref|YP_097922.1|	DNA methyltransferase	-	LGT-unresolved
gi|53711932|ref|YP_097924.1|	hypothetical protein BF0641	-	LGT-in
gi|53711982|ref|YP_097974.1|	chaperone protein DnaK	O	LGT-unresolved
gi|53712052|ref|YP_098044.1|	putative bicyclomycin resistance protein	GEPR	LGT-unresolved
gi|53712089|ref|YP_098081.1|	hypothetical protein BF0796	-	LGT-unresolved
gi|53712102|ref|YP_098094.1|	putative dNDP-4-keto-6-deoxy-glucose-2,3- dehydratase	-	LGT-in
gi|53712103|ref|YP_098095.1|	probable NDP-hexose-3-ketoreductase	R	LGT-unresolved
gi|53712104|ref|YP_098096.1|	aminotransferase	M	LGT-in
gi|53712133|ref|YP_098125.1|	endo-1,4-beta-mannosidase	-	LGT-unresolved
gi|53712135|ref|YP_098127.1|	hypothetical protein BF0842	-	LGT-in
gi|53712136|ref|YP_098128.1|	corrinoid methyltransferase protein	R	LGT-in
gi|53712148|ref|YP_098140.1|	hypothetical protein BF0855	-	LGT-unresolved
gi|53712168|ref|YP_098160.1|	hypothetical protein BF0875	R	LGT-unresolved
gi|53712227|ref|YP_098219.1|	hypothetical protein BF0935	-	LGT-in
gi|53712283|ref|YP_098275.1|	O-acetylhomoserine sulfhydrylase	E	LGT-unresolved
gi|53712389|ref|YP_098381.1|	putative glycosyltransferase	-	LGT-in
gi|53712397|ref|YP_098389.1|	putative glycosyltransferase	-	LGT-unresolved
gi|53712401|ref|YP_098393.1|	putative glycosyltransferase	M	LGT-unresolved
gi|53712403|ref|YP_098395.1|	putative acetyltransferase	R	LGT-in
gi|53712431|ref|YP_098423.1|	hypothetical protein BF1139	-	LGT-unresolved
gi|53712434|ref|YP_098426.1|	type III restriction-modification system restriction subunit	V	LGT-unresolved
gi|53712435|ref|YP_098427.1|	type III DNA modification enzyme	L	LGT-unresolved
gi|53712444|ref|YP_098436.1|	hypothetical protein BF1152	-	LGT-out
gi|53712449|ref|YP_098441.1|	hypothetical protein BF1157	R	LGT-unresolved
gi|53712452|ref|YP_098444.1|	hypothetical protein BF1160	-	LGT-in

<table>
<tr><td>gi|53712519|ref|YP_098511.1|</td><td>hypothetical protein BF1227</td><td>-</td><td>LGT-in</td></tr>
<tr><td>gi|53712523|ref|YP_098515.1|</td><td>hypothetical protein BF1231</td><td>-</td><td>LGT-unresolved</td></tr>
<tr><td>gi|53712528|ref|YP_098520.1|</td><td>hypothetical protein BF1236</td><td>-</td><td>LGT-in</td></tr>
<tr><td>gi|53712530|ref|YP_098522.1|</td><td>hypothetical protein BF1238</td><td>-</td><td>LGT-in</td></tr>
<tr><td>gi|53712539|ref|YP_098531.1|</td><td>putative alpha-xylosidase</td><td>G</td><td>LGT-in</td></tr>
<tr><td>gi|53712560|ref|YP_098552.1|</td><td>hypothetical protein BF1268</td><td>-</td><td>LGT-unresolved</td></tr>
<tr><td>gi|53712678|ref|YP_098670.1|</td><td>hypothetical protein BF1385</td><td>-</td><td>LGT-unresolved</td></tr>
<tr><td>gi|53712683|ref|YP_098675.1|</td><td>tyrosine type site-specific recombinase</td><td>L</td><td>LGT-in</td></tr>
<tr><td>gi|53712690|ref|YP_098682.1|</td><td>hypothetical protein BF1397</td><td>G</td><td>LGT-unresolved</td></tr>
<tr><td>gi|53712711|ref|YP_098703.1|</td><td>hypothetical protein BF1418</td><td>-</td><td>LGT-unresolved</td></tr>
<tr><td>gi|53712729|ref|YP_098721.1|</td><td>mannose-1-phosphate guanyltransferase</td><td>MJ</td><td>LGT-in</td></tr>
<tr><td>gi|53712735|ref|YP_098727.1|</td><td>glycosyltransferase</td><td>M</td><td>LGT-unresolved</td></tr>
<tr><td>gi|53712780|ref|YP_098772.1|</td><td>histidine decarboxylase</td><td>-</td><td>LGT-unresolved</td></tr>
<tr><td>gi|53712816|ref|YP_098808.1|</td><td>hypothetical protein BF1523</td><td>-</td><td>LGT-in</td></tr>
<tr><td>gi|53712822|ref|YP_098814.1|</td><td>putative dehydrogenase</td><td>-</td><td>LGT-unresolved</td></tr>
<tr><td>gi|53712823|ref|YP_098815.1|</td><td>hypothetical protein BF1531</td><td>Q</td><td>LGT-unresolved</td></tr>
<tr><td>gi|53712827|ref|YP_098819.1|</td><td>hypothetical protein BF1535</td><td>-</td><td>LGT-in</td></tr>
<tr><td>gi|53712828|ref|YP_098820.1|</td><td>CDP-glucose-4,6-dehydratase</td><td>MG</td><td>LGT-unresolved</td></tr>
<tr><td>gi|53712830|ref|YP_098822.1|</td><td>hypothetical protein BF1538</td><td>-</td><td>LGT-in</td></tr>
<tr><td>gi|53712839|ref|YP_098831.1|</td><td>putative glycosyltransferase</td><td>M</td><td>LGT-in</td></tr>
<tr><td>gi|53712840|ref|YP_098832.1|</td><td>glycosyltransferase</td><td>M</td><td>LGT-unresolved</td></tr>
<tr><td>gi|53712842|ref|YP_098834.1|</td><td>putative UDP-galactose 4-epimerase</td><td>MG</td><td>LGT-unresolved</td></tr>
<tr><td>gi|53712904|ref|YP_098896.1|</td><td>hypothetical protein BF1614</td><td>Q</td><td>LGT-unresolved</td></tr>
<tr><td>gi|53713039|ref|YP_099031.1|</td><td>1-phosphatidylinositol phosphodiesterase precursor</td><td>-</td><td>LGT-in</td></tr>
<tr><td>gi|53713053|ref|YP_099045.1|</td><td>hypothetical protein BF1764</td><td>-</td><td>LGT-out</td></tr>
<tr><td>gi|53713059|ref|YP_099051.1|</td><td>hypothetical protein BF1770</td><td>-</td><td>LGT-unresolved</td></tr>
<tr><td>gi|53713065|ref|YP_099057.1|</td><td>hypothetical protein BF1776</td><td>-</td><td>LGT-out</td></tr>
<tr><td>gi|53713134|ref|YP_099126.1|</td><td>putative epimerase</td><td>M</td><td>LGT-in</td></tr>
<tr><td>gi|53713149|ref|YP_099141.1|</td><td>probable urea transport protein</td><td>-</td><td>LGT-in</td></tr>
<tr><td>gi|53713152|ref|YP_099144.1|</td><td>SOS mutagenesis and repair protein UmuC homolog</td><td>L</td><td>LGT-unresolved</td></tr>
<tr><td>gi|53713167|ref|YP_099159.1|</td><td>hypothetical protein BF1877</td><td>-</td><td>LGT-unresolved</td></tr>
<tr><td>gi|53713170|ref|YP_099162.1|</td><td>coproporphyrinogen III oxidase</td><td>H</td><td>LGT-unresolved</td></tr>
<tr><td>gi|53713213|ref|YP_099205.1|</td><td>putative TonB-dependent outer membrane protein</td><td>P</td><td>LGT-unresolved</td></tr>
<tr><td>gi|53713232|ref|YP_099224.1|</td><td>hypothetical protein BF1942</td><td>-</td><td>LGT-in</td></tr>
<tr><td>gi|53713233|ref|YP_099225.1|</td><td>hypothetical protein BF1943</td><td>-</td><td>LGT-in</td></tr>
<tr><td>gi|53713244|ref|YP_099236.1|</td><td>endopeptidase Clp ATP-binding chain B</td><td>O</td><td>LGT-in</td></tr>
<tr><td>gi|53713251|ref|YP_099243.1|</td><td>hypothetical protein BF1961</td><td>-</td><td>LGT-in</td></tr>
<tr><td>gi|53713252|ref|YP_099244.1|</td><td>probabale oxalocrotonate tautomerase</td><td>-</td><td>LGT-in</td></tr>
<tr><td>gi|53713256|ref|YP_099248.1|</td><td>tyrosine type site-specific recombinase</td><td>L</td><td>LGT-in</td></tr>
<tr><td>gi|53713269|ref|YP_099261.1|</td><td>putative ATPase involved in DNA repair</td><td>-</td><td>LGT-unresolved</td></tr>
<tr><td>gi|53713359|ref|YP_099351.1|</td><td>putative transcriptional regulator</td><td>K</td><td>LGT-unresolved</td></tr>
<tr><td>gi|53713360|ref|YP_099352.1|</td><td>hypothetical protein BF2071</td><td>-</td><td>LGT-in</td></tr>
<tr><td>gi|53713366|ref|YP_099358.1|</td><td>aldo/keto reductase family oxidoreductase</td><td>R</td><td>LGT-in</td></tr>
<tr><td>gi|53713372|ref|YP_099364.1|</td><td>putative 5-amino-6-(5-phosphoribosylamino) uracil</td><td>H</td><td>LGT-unresolved</td></tr>
<tr><td>gi|53713405|ref|YP_099397.1|</td><td>hypothetical protein BF2116</td><td>-</td><td>LGT-in</td></tr>
<tr><td>gi|53713534|ref|YP_099526.1|</td><td>probable oxidoreductase</td><td>R</td><td>LGT-unresolved</td></tr>
<tr><td>gi|53713537|ref|YP_099529.1|</td><td>putative membrane efflux protein</td><td>GEPR</td><td>LGT-unresolved</td></tr>
<tr><td>gi|53713561|ref|YP_099553.1|</td><td>hypothetical protein BF2272</td><td>-</td><td>LGT-in</td></tr>
<tr><td>gi|53713611|ref|YP_099603.1|</td><td>hypothetical protein BF2322</td><td>-</td><td>LGT-in</td></tr>
<tr><td>gi|53713622|ref|YP_099614.1|</td><td>probable protease</td><td>OU</td><td>LGT-in</td></tr>
<tr><td>gi|53713698|ref|YP_099690.1|</td><td>hypothetical protein BF2407</td><td>S</td><td>LGT-unresolved</td></tr>
<tr><td>gi|53713751|ref|YP_099743.1|</td><td>putative terminase large subunit</td><td>R</td><td>LGT-in</td></tr>
<tr><td>gi|53713753|ref|YP_099745.1|</td><td>conserved hypothetical protein related to phage</td><td>-</td><td>LGT-unresolved</td></tr>
<tr><td>gi|53713761|ref|YP_099753.1|</td><td>putative methylase</td><td>L</td><td>LGT-unresolved</td></tr>
<tr><td>gi|53713771|ref|YP_099763.1|</td><td>hypothetical protein BF2480</td><td>-</td><td>LGT-in</td></tr>
</table>

gi|53713779|ref|YP_099771.1|	phosphoglycerate mutase	G	LGT-in
gi|53713805|ref|YP_099797.1|	cobalamin biosynthesis protein CobN	H	LGT-unresolved
gi|53713819|ref|YP_099811.1|	ThiS protein involved in thiamine biosynthesis	-	LGT-in
gi|53713844|ref|YP_099836.1|	hypothetical protein BF2553	-	LGT-out
gi|53713858|ref|YP_099850.1|	putative UDP-galactose 4-epimerase	MG	LGT-unresolved
gi|53713859|ref|YP_099851.1|	putative glycosyltransferase	M	LGT-unresolved
gi|53713861|ref|YP_099853.1|	putative epimerase	M	LGT-in
gi|53713868|ref|YP_099860.1|	putative alcohol dehydrogenase	C	LGT-unresolved
gi|53713872|ref|YP_099864.1|	putative carbamoylphosphate synthase large subunit short form	-	LGT-unresolved
gi|53713965|ref|YP_099957.1|	cytochrome c peroxidase	P	LGT-in
gi|53713966|ref|YP_099958.1|	hypothetical protein BF2674	E	LGT-in
gi|53713970|ref|YP_099962.1|	hypothetical protein BF2678	GEPR	LGT-in
gi|53713971|ref|YP_099963.1|	hypothetical protein BF2679	V	LGT-unresolved
gi|53714000|ref|YP_099992.1|	hypothetical protein BF2708	R	LGT-unresolved
gi|53714001|ref|YP_099993.1|	hypothetical protein BF2709	-	LGT-in
gi|53714002|ref|YP_099994.1|	putative peptide chain release factor	J	LGT-in
gi|53714076|ref|YP_100068.1|	putative glycosyltransferase	M	LGT-unresolved
gi|53714077|ref|YP_100069.1|	serine acetyltransferase	E	LGT-unresolved
gi|53714093|ref|YP_100085.1|	putative site-specific DNA-methyltransferase	L	LGT-unresolved
gi|53714099|ref|YP_100091.1|	hypothetical protein BF2807	-	LGT-unresolved
gi|53714114|ref|YP_100106.1|	putative partitioning protein	K	LGT-unresolved
gi|53714127|ref|YP_100119.1|	ATP-dependent Clp protease ATP-binding subunit	O	LGT-in
gi|53714138|ref|YP_100130.1|	hypothetical protein BF2846	-	LGT-in
gi|53714139|ref|YP_100131.1|	putative Rhs protein	M	LGT-in
gi|53714141|ref|YP_100133.1|	putative RhsD protein	M	LGT-in
gi|53714143|ref|YP_100135.1|	putative RhsD protein	M	LGT-in
gi|53714145|ref|YP_100137.1|	hypothetical protein BF2854	-	LGT-unresolved
gi|53714148|ref|YP_100140.1|	hypothetical protein BF2857	-	LGT-unresolved
gi|53714149|ref|YP_100141.1|	putative DNA methylase	KLL	LGT-unresolved
gi|53714152|ref|YP_100144.1|	hypothetical protein BF2861	-	LGT-in
gi|53714169|ref|YP_100161.1|	hypothetical protein BF2878	-	LGT-in
gi|53714173|ref|YP_100165.1|	hypothetical protein BF2882	M	LGT-in
gi|53714177|ref|YP_100169.1|	putative hemagglutinin	NU	LGT-in
gi|53714180|ref|YP_100172.1|	putative DNA primase	L	LGT-in
gi|53714199|ref|YP_100191.1|	hypothetical protein BF2908	-	LGT-in
gi|53714222|ref|YP_100214.1|	hypothetical protein BF2931	-	LGT-in
gi|53714234|ref|YP_100226.1|	hypothetical protein BF2944	-	LGT-unresolved
gi|53714235|ref|YP_100227.1|	hypothetical protein BF2945	-	LGT-unresolved
gi|53714241|ref|YP_100233.1|	putative phage-related protein	-	LGT-unresolved
gi|53714245|ref|YP_100237.1|	hypothetical protein BF2955	-	LGT-unresolved
gi|53714247|ref|YP_100239.1|	hypothetical protein BF2957	-	LGT-in
gi|53714254|ref|YP_100246.1|	hypothetical protein BF2964	T	LGT-unresolved
gi|53714255|ref|YP_100247.1|	hypothetical protein BF2965	-	LGT-in
gi|53714297|ref|YP_100289.1|	hypothetical protein BF3009	-	LGT-in
gi|53714320|ref|YP_100312.1|	putative cell surface protein	-	LGT-out
gi|53714344|ref|YP_100336.1|	hypothetical protein BF3056	SS	LGT-in
gi|53714352|ref|YP_100344.1|	periplasmic beta-glucosidase precursor	G	LGT-unresolved
gi|53714355|ref|YP_100347.1|	trimethylamine corrinoid protein	R	LGT-in
gi|53714356|ref|YP_100348.1|	putative methyltransferase CmuC	-	LGT-unresolved
gi|53714357|ref|YP_100349.1|	periplasmic beta-glucosidase precursor	G	LGT-unresolved
gi|53714379|ref|YP_100371.1|	putative membrane transport protein	GEPR	LGT-unresolved
gi|53714404|ref|YP_100396.1|	putative protein-tyrosine phosphatase	T	LGT-in
gi|53714426|ref|YP_100418.1|	hypothetical protein BF3139	-	LGT-unresolved
gi|53714431|ref|YP_100423.1|	ABC transporter ATP-binding protein	V	LGT-in

gi|53714432|ref|YP_100424.1|	multidrug resistance ABC transporter	V	LGT-unresolved
gi|53714467|ref|YP_100459.1|	putative aminopeptidase C	S	LGT-in
gi|53714497|ref|YP_100489.1|	putative serum resistance protein BrkB	S	LGT-unresolved
gi|53714498|ref|YP_100490.1|	hypothetical protein BF3211	-	LGT-in
gi|53714521|ref|YP_100513.1|	hypothetical protein BF3234	-	LGT-in
gi|53714539|ref|YP_100531.1|	hypothetical protein BF3253	-	LGT-in
gi|53714562|ref|YP_100554.1|	putative peptide synthetase	Q	LGT-in
gi|53714589|ref|YP_100581.1|	hypothetical protein BF3303	-	LGT-in
gi|53714591|ref|YP_100583.1|	hypothetical protein BF3305	-	LGT-out
gi|53714596|ref|YP_100588.1|	trehalose/maltose hydrolase	G	LGT-in
gi|53714617|ref|YP_100609.1|	hypothetical protein BF3331	-	LGT-unresolved
gi|53714676|ref|YP_100668.1|	iron-sulfur flavoprotein	R	LGT-in
gi|53714697|ref|YP_100689.1|	hypothetical protein BF3411	-	LGT-unresolved
gi|53714698|ref|YP_100690.1|	putative glycosyltransferase	M	LGT-in
gi|53714708|ref|YP_100700.1|	putative glycosyltransferase	M	LGT-unresolved
gi|53714726|ref|YP_100718.1|	dolichyl-phosphate-mannose-protein mannosyltransferase family protein	M	LGT-unresolved
gi|53714727|ref|YP_100719.1|	putative lipid-A-disaccharide synthase	S	LGT-in
gi|53714728|ref|YP_100720.1|	dolichol-phosphate mannosyltransferase	M	LGT-unresolved
gi|53714741|ref|YP_100733.1|	hypothetical protein BF3456	-	LGT-in
gi|53714759|ref|YP_100751.1|	hypothetical protein BF3474	-	LGT-in
gi|53714770|ref|YP_100762.1|	hypothetical protein BF3485	K	LGT-in
gi|53714794|ref|YP_100786.1|	hypothetical protein BF3509	S	LGT-in
gi|53714817|ref|YP_100809.1|	putative isochorismatase	-	LGT-unresolved
gi|53714819|ref|YP_100811.1|	putative peptidase	VS	LGT-in
gi|53714822|ref|YP_100814.1|	hypothetical protein BF3537	S	LGT-in
gi|53714833|ref|YP_100825.1|	hypothetical protein BF3548	-	LGT-unresolved
gi|53714852|ref|YP_100844.1|	putative DNA helicase	L	LGT-unresolved
gi|53714923|ref|YP_100915.1|	hypothetical protein BF3638	-	LGT-unresolved
gi|53714935|ref|YP_100927.1|	glycosyltransferase	-	LGT-unresolved
gi|53714938|ref|YP_100930.1|	putative O-acetyltransferase	R	LGT-in
gi|53714942|ref|YP_100934.1|	putative glycosyltransferase	M	LGT-unresolved
gi|53714945|ref|YP_100937.1|	hypothetical protein BF3660	J	LGT-in
gi|53714967|ref|YP_100959.1|	type II restriction endonuclease	-	LGT-in
gi|53714970|ref|YP_100962.1|	putative acetyltransferase	M	LGT-in
gi|53714972|ref|YP_100964.1|	putative transcriptional regulator	E	LGT-in
gi|53715123|ref|YP_101115.1|	putative adenosine deaminase	F	LGT-in
gi|53715126|ref|YP_101118.1|	hypothetical protein BF3842	-	LGT-unresolved
gi|53715151|ref|YP_101143.1|	elongation factor G	J	LGT-unresolved
gi|53715194|ref|YP_101186.1|	putative phage-related protein	-	LGT-in
gi|53715197|ref|YP_101189.1|	hypothetical protein BF3913	-	LGT-in
gi|53715199|ref|YP_101191.1|	putative glycosyltransferase	M	LGT-unresolved
gi|53715225|ref|YP_101217.1|	putative transcriptional regulator	K	LGT-in
gi|53715232|ref|YP_101224.1|	putative secreted sulfatase ydeN precursor	P	LGT-unresolved
gi|53715258|ref|YP_101250.1|	hypothetical protein BF3974	-	LGT-unresolved
gi|53715303|ref|YP_101295.1|	beta-galactosidase precursor	G	LGT-unresolved
gi|53715344|ref|YP_101336.1|	probable transposase	L	LGT-in
gi|53715370|ref|YP_101362.1|	putative transcriptional regulator	TK	LGT-in
gi|53715408|ref|YP_101400.1|	hypothetical protein BF4124	-	LGT-in
gi|53715508|ref|YP_101500.1|	hypothetical protein BF4223	-	LGT-in
gi|53715509|ref|YP_101501.1|	hypothetical protein BF4224	-	LGT-in
gi|53715510|ref|YP_101502.1|	truncated IS116 family transposase	-	LGT-in
gi|53715518|ref|YP_101510.1|	hypothetical protein BF4233	-	LGT-in
gi|53715536|ref|YP_101528.1|	beta-galactosidase precursor	G	LGT-unresolved
gi|53715544|ref|YP_101536.1|	hypothetical protein BF4261	-	LGT-in

gi|53715545|ref|YP 101537.1|	probable truncated transposase	-	LGT-in
gi|53715569|ref|YP_101561.1|	hypothetical protein BF4287	-	LGT-in
gi|53715582|ref|YP_101574.1|	putative maltose O-acetyltransferase	R	LGT-unresolved
gi|53715664|ref|YP 101656.1|	xylose permease	GEPR	LGT-unresolved
gi|53715667|ref|YP 101659.1|	hypothetical protein BF4387	S	LGT-in
gi|53715677|ref|YP_101669.1|	hemolysin erythrocyte lysis protein	ME	LGT-out
gi|53715723|ref|YP_101715.1|	putative glycerophosphoryl diester phosphodiesterase	C	LGT-unresolved
gi|53715833|ref|YP 101825.1|	putative sulfite reductase flavoprotein component	S	LGT-in
gi|53715844|ref|YP 101836.1|	hypothetical protein BF4565	-	LGT-unresolved

Legend: For an explanation of COG-based functional codes, see **Figure S1**. The LGT column defines the predicted evolutionary history of the coding sequence: LGT-in, laterally transferred into the genome; LGT-out, laterally transferred out of the genome; LGT-unresolved, laterally transferred but direction unknown. See **Materials and Methods** for detailed explanations.
