# Supplementary material for: Evolution of Symbiotic Bacteria in the Distal Human Intestine
Source: PLoS Biol. 2007 Jun 19;5(7):e156. doi: 10.1371/journal.pbio.0050156 (PMC1892571; doi:10.1371/journal.pbio.0050156)
Supplement: Supplementary file 9 [file pbio.0050156.st005.pdf]

Table S5. ***CPS*** **loci of** ***B. distasonis*** **ATCC 8503 (A),** ***B. vulgatus*** **ATCC 8482 (B),** ***B. thetaiotaomicron*** **ATCC 29148 (C),** ***B. fragilis*** **NCTC 9343 (D) and** ***B. fragilis*** **YCH 46 (E).**

(A). *CPS* loci of *B. distasonis* ATCC 8503

**Locus 1**			
**Bd0013**	**integrase**	**41.4**	NO
Bd0014c	hypothetical protein	29.2	NOVEL
**Bd0015**	**putative transcriptional regulator UpxY homolog**	**43.6**	NO
Bd0016	undecaprenyl-phosphate alpha-N-acetylglucosaminyltransferase	43.8	NO
Bd0017	polysaccharide export protein, BexD/CtrA/VexA family	47.5	NO
Bd0018	capsular polysaccharide transport protein, putative	46.1	NO
Bd0019	capsular polysaccharide biosynthesis protein, putative	42.9	NO
Bd0020	conserved hypothetical protein	52.2	NO
Bd0021c	***N-acetylmuramoyl-L-alanine amidase, putative***	***52.2***	NO
Bd0022c	***conserved hypothetical protein***	***50.5***	UNRESOLVED
Bd0023c	***conserved hypothetical protein***	***42.2***	NO
Bd0024	hypothetical protein	46.0	NOVEL
Bd0025	putative UDP-ManNAc dehydrogenase	43.5	NO
**Bd0026**	**nucleoside-diphosphate-sugar epimerase**	**42.8**	LGT-in
Bd0027	putative UDP-glucose-6 dehydrogenase	39.6	NO
Bd0028	conserved hypothetical protein, putative glycosyltransferase	31.4	UNRESOLVED
Bd0029	hypothetical protein	33.6	NO
Bd0030	putative acetyltransferase (isoleucine patch superfamily)	33.2	UNRESOLVED
Bd0031	O-antigen repeat unit transporter	34.6	UNRESOLVED
Bd0032	acetyltransferase	34.4	NO
Bd0033	glycosyltransferase family 4, distantly related to alpha-glycosyltransferases	33.5	UNRESOLVED
**Bd0034**	**conserved hypothetical protein, putative glycosyltransferase**	**34.4**	LGT-in
Bd0035	hypothetical protein	33.6	NOVEL
Bd0036	hypothetical protein	31.1	NOVEL
Bd0037	hypothetical protein	35.0	NOVEL
Bd0038	conserved hypothetical protein	35.5	UNRESOLVED
Bd0039	glycosyltransferase family 4, related to alpha-glycosyltransferases	33.6	UNRESOLVED
Bd0040	putative serine acetyltransferase, putative hexapeptide transferase family protein	36.0	NO
Bd0041	conserved hypothetical protein	39.4	NO
Bd0042	glycosyltransferase family 26, related to beta-N-acetyl mannosaminuronic acid transferase	41.8	NO
Bd0043	putative UDP-GlcNAc 2-epimerase	43.1	NO
Bd0044	conserved hypothetical protein, putative glycosyltransferase	33.3	NOVEL
Bd0045	conserved hypothetical protein	34.3	UNRESOLVED
**Locus 2**			
**Bd0119**	**integrase / site-specific recombinase**	**46.9**	NO
Bd0120	hypothetical protein	46.7	NOVEL
**Bd0121**	**putative transcriptional regulator UpxY homolog**	**60.1**	NO
Bd0122	hypothetical protein	58.2	NO
Bd0123	conserved hypothetical protein	56.0	UNRESOLVED
Bd0124	polysaccharide export outer membrane protein	57.4	NO
Bd0125	tyrosine-protein kinase	57.9	NO
**Bd0126**	**putative capsular polysaccharide biosynthesis protein**	**44.3**	LGT-in
Bd0127	putative aminotransferase	47.5	NO
Bd0128	putative nucleotide-diphosphate sugar epimerase	50.3	NO
Bd0129c	hypothetical protein	40.1	NOVEL

Bd0130	hypothetical protein	49.8	NOVEL
Bd0131	conserved hypothetical protein	49.4	UNRESOLVED
**Locus 3**			
**Bd0287**	**integrase**	**54.5**	NO
**Bd0288**	**putative transcriptional regulator UpxY homolog**	**57.6**	NO
Bd0289	hypothetical protein	58.5	NO
Bd0290	undecaprenyl-phosphate alpha-N-acetylglucosaminyltransferase	49.8	NO
Bd0291	putative LPS biosynthesis related flippase	40.2	UNRESOLVED
**Bd0292**	**glycosyltransferase family 2, related to beta-glycosyltransferases**	**30.8**	LGT-out
Bd0293	putative lipopolysaccharide biosynthesis related polysaccharide polymerase	30.7	NOVEL
Bd0294	hypothetical protein	28.5	NOVEL
**Locus 4**			
**Bd0404**	**integrase**	43.5	NO
**Bd0405**	**putative transcriptional regulator UpxY homolog**	**48.9**	NO
Bd0406	hypothetical protein	47.4	NO
Bd0407	putative nucleoside-diphosphate sugar epimerase/dehydrase	46.9	NO
Bd0408	conserved hypothetical protein	45.5	UNRESOLVED
Bd0409	polysaccharide export outer membrane protein	47.2	NO
Bd0410	putative EPS related membrane protein	44.9	NO
Bd0411	aminotransferase family protein	44.9	UNRESOLVED
Bd0412	putative UDP-galactose phosphate transferase involved in lipopolysaccharide synthesis	37.1	UNRESOLVED
Bd0413	putative acetyltransferase, GNAT family	35.2	UNRESOLVED
**Bd0414**	**1-aminocyclopropane-1-carboxylate deaminase**	**36.0**	LGT-unresolved
**Bd0415**	**carbamoylphosphate synthase large subunit (split gene in MJ)**	**35.8**	LGT-unresolved
Bd0416	putative hexapeptide transferase family protein	39.1	UNRESOLVED
**Bd0417**	**NAD/NADP octopine/nopaline dehydrogenas**	**39.3**	LGT-unresolved
Bd0418	putative hexapeptide transferase family protein	33.1	UNRESOLVED
Bd0419	capsule biosynthesis protein	34.4	UNRESOLVED
Bd0420	putative capsule biosynthesis protein	34.9	UNRESOLVED
Bd0421	glycosyltransferase family 4, related to alpha-glycosyltransferases	36.0	UNRESOLVED
**Bd0422**	**hypothetical protein**	**29.1**	LGT-in
Bd0423	putative glycosyltransferase	32.2	UNRESOLVED
**Bd0424**	**putative CPS repeating unit transporter**	**30.8**	LGT-in
**Bd0425**	**uridine diphosphate glucose dehydrogenase**	**38.8**	LGT-unresolved
**Locus 5**			
**Bd0528**	**integrase / site-specific recombinase**	**46.5**	NO
Bd0529	hypothetical protein	45.5	NOVEL
**Bd0530**	**putative transcriptional regulator UpxY homolog**	**59.1**	NO
Bd0531	hypothetical protein	54.5	NO
Bd0532	putative capsular polysaccharide biosynthesis glycosyltransferase	51.5	NO
Bd0533c	***putative N-acetylmuramoyl-L-alanine amidase***	***54.5***	NO
Bd0534c	***hypothetical protein***	***50.2***	NOVEL
Bd0535c	***hypothetical protein***	***50.0***	NOVEL
Bd0536c	***conserved hypothetical protein***	***58.1***	NO
Bd0537c	***conserved hypothetical protein***	***55.6***	NO
Bd0538	hypothetical protein	58.4	NOVEL
Bd0539	hypothetical protein	43.8	NOVEL
**Bd0540**	**exopolysaccharide biosynthesis protein**	**41.0**	LGT-out
Bd0541	putative ATP-grasp enzyme	34.2	UNRESOLVED

Bd0542	hypothetical protein	43.8	NOVEL
Bd0543	putative O-antigen polymerase	35.4	NOVEL
**Bd0544**	**conserved hypothetical protein**	**43.4**	LGT-in
**Bd0545**	**putative xylanase/chitin deacetylase**	**41.6**	LGT-in
Bd0546	hypothetical protein	44.2	NOVEL
Bd0547	putative acyl carrier protein	38.2	UNRESOLVED
Bd0548	3-oxoacyl-[acyl-carrier-protein] reductase	39.1	NO
Bd0549	3-oxoacyl-[acyl-carrier-protein] synthase	43.3	UNRESOLVED
Bd0550	putative acyl carrier protein	40.6	NO
Bd0551	conserved hypothetical protein	40.2	NO
Bd0552	3-oxoacyl-[acyl-carrier-protein] reductase	39.7	NO
**Bd0553**	**glycosyltransferase family 4, distantly related to alpha-glycosyltransferases**	**41.1**	LGT-unresolved
Bd0554	putative dehydratase	43.3	NO
Bd0555	putative reductase	35.5	NO
Bd0556	putative UDP-N-acetylglucosamine 2-epimerase	36.1	UNRESOLVED
Bd0557	glycosyltransferase family 4, distantly related to alpha-glycosyltransferases	35.4	NO
Bd0558	conserved hypothetical protein	44.8	NO
Bd0559c	hypothetical protein	41.3	NOVEL
Bd0560	amidophosphoribosyltransferase	49.9	NO
Bd0561	carbamoyl phosphate synthetase III (glutamine-hydrolyzing)	51.4	NO
Bd0562	carbamyl phosphate synthetase	53.4	NO
Bd0563	conserved hypothetical protein	49.6	NO
Bd0564	oxidoreductase, putative glycolate oxidase	51.4	NO
Bd0565	putative electron transport protein	51.4	NO
Bd0566	conserved hypothetical protein	52.1	NO
**Locus 6**			
Bd1310c	glycoside hydrolase family 43, candidate beta-glycosidase	49.4	NO
Bd1311c	L-serine dehydratase, iron-sulfur-dependent, single chain form	52.5	NO
Bd1312c	putative transmembrane protein	44.5	NO
**Bd1313c**	**conserved hypothetical protein**	**47.9**	LGT-out
Bd1314c	conserved hypothetical protein with a conserved domain	41.6	NO
Bd1315c	glycosyltransferase family 2, related to beta-glycosyltransferases	41.2	NO
**Bd1316c**	**Fe-S oxidoreductase**	**43.8**	LGT-in
Bd1317c	conserved hypothetical protein	38.1	UNRESOLVED
Bd1318c	conserved hypothetical protein	31.0	UNRESOLVED
**Bd1319c**	**putative glycero-phosphotransferase**	**30.6**	LGT-in
**Bd1320c**	**ribulose-phosphate 3-epimerase**	**35.3**	LGT-unresolved
**Bd1321c**	**conserved hypothetical protein**	**35.0**	LGT-unresolved
Bd1322c	nucleotidyltransferase family protein	33.5	UNRESOLVED
Bd1323c	hypothetical protein	40.8	NOVEL
Bd1324c	putative polysaccharide transporter/flippase	45.9	UNRESOLVED
Bd1325c	conserved hypothetical protein	54.3	UNRESOLVED
Bd1326c	putative capsular polysaccharide biosynthesis glycosyltransferase	52.0	NO
Bd1327c	hypothetical protein	43.4	NOVEL
Bd1328c	hypothetical protein	53.3	NO
**Bd1329c**	**putative transcriptional regulator UpxY homolog**	**56.2**	NO
**Bd1330c**	**integrase**	**48.3**	NO
**Locus 7**			
**Bd1377c**	**glycosyltransferase family 4, related to alpha-glycosyltransferases**	**33.5**	LGT-unresolved
Bd1378c	hypothetical protein	28.0	NO

**Bd1379c**	**acetyltransferase (isoleucine patch superfamily)**	**35.3**	LGT-unresolved
Bd1380c	glycosyltransferase family 4, related to alpha-glycosyltransferases	32.4	UNRESOLVED
Bd1381c	conserved hypothetical protein	28.2	NO
Bd1382c	glycosyltransferase family 2, related to beta-glycosyltransferases	31.3	UNRESOLVED
**Bd1383c**	**exopolysaccharide biosynthesis protein, putative galactosyltransferase**	**28.6**	LGT-in
Bd1384c	putative cholinephosphotransferase	28.6	UNRESOLVED
Bd1385c	putative O-antigen transporter	28.4	UNRESOLVED
Bd1386c	hemolysin erythrocyte lysis protein 2	38.0	NO
Bd1387c	hypothetical protein	36.9	NOVEL
Bd1388c	conserved hypothetical protein	54.6	UNRESOLVED
Bd1389c	putative LPS biosynthesis related glycosyltransferase	49.7	NO
Bd1390c	hypothetical protein	53.0	NO
**Bd1391c**	**putative transcriptional regulator UpxY homolog**	**56.3**	NO
**Bd1392c**	**integrase**	**48.3**	NO
**Locus 8**			
**Bd1544c**	**glycosyltransferase family 4, distantly related to alpha-glycosyltransferases**	**49.7**	LGT-out
Bd1545c	putative UDP-GlcNAc 2-epimerase	52.2	NO
Bd1546c	putative UDP-ManNAc dehydrogenase	53.8	NO
**Bd1547c**	**conserved hypothetical protein, putative prenyltransferase and squalene oxidase**	**44.1**	LGT-in
**Bd1548c**	**putative zinc-binding dehydrogenase**	**50.1**	LGT-in
Bd1549c	conserved hypothetical protein, putative glycosyltransferase group 1	29.1	UNRESOLVED
Bd1550c	glycosyltransferase family 4, related to alpha-glycosyltransferases	34.2	UNRESOLVED
Bd1551c	hypothetical protein	33.7	NOVEL
Bd1552c	glycosyltransferase family 2, related to beta-glycosyltransferases	39.4	NO
Bd1553c	putative flippase	35.9	NO
Bd1554c	conserved hypothetical protein, putative nucleotidyltransferase	36.3	UNRESOLVED
Bd1555c	conserved hypothetical protein, putative nucleotidyltransferase	40.5	UNRESOLVED
Bd1556c	hypothetical protein	54.9	NO
Bd1557	***conserved hypothetical protein***	***52.9***	NO
Bd1558	***conserved hypothetical protein***	***55.6***	NO
Bd1559	***hypothetical protein***	***49.3***	NOVEL
Bd1560	***conserved hypothetical protein***	***56.4***	NO
Bd1561	***putative N-acetylmuramoyl-L-alanine amidase***	***53.2***	UNRESOLVED
Bd1562c	putative LPS biosynthesis related glycosyltransferase	49.4	NO
Bd1563c	tyrosine-protein kinase	57.9	NO
Bd1564c	polysaccharide export outer membrane protein	57.4	NO
Bd1565c	conserved hypothetical protein	56.0	UNRESOLVED
Bd1566c	hypothetical protein	58.2	NO
**Bd1567c**	**putative transcriptional regulator UpxY homolog**	**60.1**	NO
**Bd1568c**	**integrase**	**44.5**	NO
**Locus 9**			
Bd1791c	outer membrane assembly protein	45.5	NO
Bd1792c	putative nucleotide-diphosphate sugar epimerase	50.3	NO
Bd1793c	glycosyltransferase	36.6	NO
Bd1794c	UDP-bacillosamine synthetase	44.7	NO
Bd1795c	glycosyltransferase family 2, related to beta-glycosyltransferases	39.8	UNRESOLVED
**Bd1796c**	**lipopolysaccharide biosynthesis protein**	**34.5**	LGT-unresolved
Bd1797c	pyrophosphorylase	41.2	NO
**Bd1798c**	**glycosyltransferase family 4, related to alpha-glycosyltransferases**	**34.0**	LGT-in

Bd1799c	conserved hypothetical protein	27.1	NO
Bd1800c	glycosyltransferase family 2, related to beta-glycosyltransferases	34.8	UNRESOLVED
**Bd1801c**	**polysaccharide pyruvyl transferase YvfF**	**32.5**	LGT-in
Bd1802c	conserved hypothetical transmembrane protein; putative transmembrane protein	43.3	NO
**Bd1803c**	**putative transcriptional regulator Updx homolog**	**48.7**	UNRESOLVED
**Locus 10**			
Bd2152c	putative transcriptional regulator UpxY homolog	45.4	NO
**Bd2153c**	**mannose-6-phosphate isomerase (phosphomannose isomerase) ; mannose-1-phosphate guanylyl transferase (GDP-mannose pyrophosphorylase)**	**49.2**	LGT-unresolved
**Bd2154c**	**GDP-4-keto-6-deoxy-D-mannose-3,5-epimerase**	**52.6**	LGT-unresolved
**Bd2155c**	**GDP-mannose 4,6-dehydratase**	**48.3**	LGT-out
Bd2156c	glycosyltransferase family 2, related to beta-glycosyltransferases	38.7	NO
**Bd2157c**	**acetyltransferase (isoleucine patch superfamily)**	**39.3**	LGT-unresolved
**Bd2158c**	**glycosyltransferase family 4, distantly related to alpha-glycosyltransferases**	**36.8**	LGT-unresolved
Bd2159c	glycosyltransferase family 4, related to alpha-glycosyltransferases	37.1	NO
Bd2160c	hypothetical protein	32.8	NOVEL
Bd2161c	hypothetical protein	35.7	NOVEL
Bd2162c	hypothetical protein	36.5	NOVEL
Bd2163c	glycosyltransferase family 2, related to beta-glycosyltransferases	31.3	UNRESOLVED
Bd2164c	conserved hypothetical protein	35.5	UNRESOLVED
Bd2165c	glycosyltransferase family 2, related to beta-glycosyltransferases	33.3	UNRESOLVED
Bd2166c	glycosyltransferase family 2, related to beta-glycosyltransferases	33.7	UNRESOLVED
Bd2167c	UDP-glucose 6-dehydrogenase	39.8	UNRESOLVED
**Bd2168c**	**glycosyltransferase family 4, distantly related to alpha-glycosyltransferases**	**32.9**	LGT-in
Bd2169c	conserved hypothetical protein	33.7	UNRESOLVED
Bd2170c	capsular polysaccharide repeat unit transporter	34.4	UNRESOLVED
Bd2171c	putative UDP-glucose-6 dehydrogenase	43.3	NO
Bd2172c	hypothetical protein	35.5	NOVEL
Bd2173c	hypothetical protein	54.7	NOVEL
Bd2174c	capsular polysaccharide biosythesis protein, putative	47.5	NO
Bd2175c	putative tyrosine-protein kinase ptk	49.2	NO
Bd2176c	polysaccharide export protein, BexD/CtrA/VexA family	49.0	NO
Bd2177c	putative LPS biosynthesis related glycosyltransferase	47.1	NO
**Bd2178c**	**putative transcriptional regulator UpxY homolog**	**49.5**	NO
Bd2179c	hypothetical protein	39.1	NOVEL
Bd2180	hypothetical protein	32.6	NOVEL
**Bd2181c**	**transposase**	**56.7**	NO
**Locus 11**			
Bd3151c	hypothetical protein	39.0	NOVEL
Bd3152c	hypothetical protein	41.4	NOVEL
**Bd3153c**	**mannose-6-phosphate isomerase (phosphomannose isomerase) ; mannose-1-phosphate guanylyl transferase (GDP-mannose pyrophosphorylase)**	**48.8**	LGT-unresolved
Bd3154c	glycosyltransferase family 2, related to beta-glycosyltransferases	34.6	UNRESOLVED
Bd3155c	glycosyltransferase family 4, related to alpha-glycosyltransferases	33.4	NOVEL
Bd3156c	glycosyltransferase family 2, related to beta-glycosyltransferases	37.8	UNRESOLVED
Bd3157c	putative O-antigen polymerase (wzy)	36.7	NOVEL

**Bd3158c**	**glycosyltransferase family 4, related to alpha-glycosyltransferases**	**41.4**	LGT-in
Bd3159c	glycosyltransferase family 2, related to beta-glycosyltransferases	41.1	UNRESOLVED
Bd3160c	glycosyltransferase family 4, distantly related to alpha-glycosyltransferases	38.6	UNRESOLVED
**Bd3161c**	**glycosyltransferase family 8, related to alpha-glycosyltransferases**	**38.3**	LGT-unresolved
**Bd3162c**	**putative O-acetyltransferase**	**38.9**	LGT-in
Bd3163c	putative flippase	35.0	NO
Bd3164c	glycosyltransferase family 4, related to alpha-glycosyltransferases	40.1	UNRESOLVED
Bd3165c	UDP-glucose 6-dehydrogenase	42.7	UNRESOLVED
Bd3166c	putative capsular polysaccharide biosythesis protein	46.0	NO
Bd3167c	putative tyrosine protein kinase	48.8	NO
Bd3168c	polysaccharide export protein, BexD/CtrA/VexA family	47.8	NO
Bd3169c	putative LPS biosynthesis related glycosyltransferase	47.8	NO
**Bd3170c**	**putative transcriptional regulator UpxY homolog**	**49.3**	NO
Bd3171c	hypothetical protein	38.7	NOVEL
**Bd3172c**	**transposase**	**56.3**	NO
**Locus 12**			
**Bd3530**	**integrase**	**44.9**	NO
**Bd3531**	**putative transcriptional regulator UpxY homolog**	**48.6**	NO
Bd3532	hypothetical protein	49.1	NO
Bd3533c	***putative N-acetylmuramoyl-L-alanine amidase***	***52.2***	NO
Bd3534c	***conserved hypothetical protein***	***47.9***	NO
Bd3535	hypothetical protein	*50.4*	NOVEL
Bd3536	putative nucleoside-diphosphate sugar epimerase/dehydrase	*51.2*	NO
Bd3537	dTDP-glucose 4,6-dehydratase	*41.7*	NO
Bd3538	conserved hypothetical protein	39.6	NO
Bd3539	conserved hypothetical protein	39.8	NO
**Bd3540**	**conserved hypothetical protein**	**31.2**	LGT-unresolved
Bd3541	putative aminotransferase	40.3	NO
**Bd3542**	**putative flippase, involved in lipopolysaccharide biosynthesis**	**37.9**	LGT-unresolved
Bd3543	hypothetical protein	28.8	NOVEL
**Bd3544**	**hypothetical protein**	**30.7**	LGT-in
Bd3545	conserved hypothetical protein	35.4	NO
**Bd3546**	**2-C-methyl-D-erythritol 4-phosphate cytidylyltransferase**	**37.6**	LGT-unresolved
Bd3547	putative nucleotide-sugar dehydratase	39.1	UNRESOLVED
**Bd3548**	**lipopolysaccharide biosynthesis protein**	**32.6**	LGT-unresolved
**Bd3549**	**putative acetyltransferase**	**34.1**	LGT-unresolved
Bd3550	putative acyl carrier protein	41.0	UNRESOLVED
Bd3551	3-oxoacyl-[acyl-carrier-protein] reductase	42.3	NO
Bd3552	3-oxoacyl-[acyl-carrier-protein] synthase III	41.4	NO
Bd3553	hypothetical protein	37.2	NOVEL
**Bd3554**	**putative xylanase/chitin deacetylase**	**39.0**	LGT-unresolved
Bd3555	putative undecaprenyl-phosphate glycosyl-1-phosphate transferase	40.0	NO
Bd3556+A309	probable serine O-acetyltransferase	32.2	NO
Bd3557	putative lactoylglutathione lyase	36.5	NO
Bd3558	conserved hypothetical protein	35.0	NO
Bd3559	conserved hypothetical protein, putative acyl carrier protein	36.0	NO
Bd3560	conserved hypothetical protein	41.7	UNRESOLVED
Bd3561	conserved hypothetical protein	40.1	NOVEL
Bd3562	putative aminotransferase	49.7	NO
Bd3563	hypothetical protein	37.1	NOVEL
**Bd3564**	**putative DNA binding protein**	**32.4**	LGT-unresolved

Bd3565	putative urocanate hydratase	48.7	NO
Bd3566	formiminotransferase-cyclodeaminase	48.3	NO
Bd3567	imidazolonepropionase	48.7	UNRESOLVED
Bd3568	putative formiminotransferase-cyclodeaminase	48.6	NO
Bd3569	histidine ammonia-lyase	48.2	NO
Bd3570	conserved hypothetical protein	42.9	NO
**Locus 13**			
**Bd3718**	**integrase**	**49.0**	NO
Bd3719c	putative transposase	42.2	NO
Bd3720c	transposase	47.1	NO
**Bd3721**	**putative transcriptional regulator UpxY homolog**	**51.2**	NO
Bd3722	hypothetical protein	56.3	NO
Bd3723	putative nucleotide-diphosphate sugar epimerase	55.4	NO
Bd3724c	***conserved hypothetical protein***	***56.0***	NO
Bd3725c	***conserved hypothetical protein***	***54.5***	NO
Bd3726c	***conserved hypothetical protein***	***56.3***	UNRESOLVED
Bd3727c	***conserved hypothetical protein***	***54.6***	NO
Bd3728	conserved hypothetical protein	47.2	NO
Bd3729	putative nucleotide-sugar dehydrogenase	46.3	NO
**Bd3730**	**conserved hypothetical protein, putative glycosyltransferase**	**34.2**	LGT-unresolved
Bd3731	putative flippase	50.1	UNRESOLVED
Bd3732	conserved hypothetical protein	34.9	NOVEL
Bd3733	hypothetical protein	30.4	NOVEL
**Bd3734**	**conserved hypothetical protein**	**31.5**	LGT-in
Bd3735	putative CDP-ribitol pyrophosphorylase	38.7	NO
Bd3736	putative dTDP-glucose 4-6-dehydratase	35.6	NO
Bd3737	putative teichoic acid biosynthesis protein F	35.7	NO
Bd3738	putative dehydratase	48.0	NO
Bd3739	capsular polysaccharide biosynthesis protein Cps4K	43.7	NO
Bd3740	putative UDP-N-acetylglucosamine 2-epimerase	44.4	UNRESOLVED
Bd3741	putative lipopolysaccharide biosynthesis protein	30.7	NO
**Bd3742**	**conserved hypothetical protein**	**45.8**	LGT-unresolved
Bd3743	UDP-galactose phosphate transferase	40.5	UNRESOLVED
**Bd3744**	**diaminopimelate decarboxylase**	**38.0**	LGT-out
Bd3745	putative carbamoylphosphate synthase large subunit, short form	35.8	UNRESOLVED
**Bd3746**	**UDP-glucose 4-epimerase (galactowaldenase)**	**34.8**	LGT-unresolved

(B). *CPS* loci of *B. vulgatus* ATCC 8482

**Locus 1**			
Bv0666c	putative UDP-GlcNAc 2-epimerase	43.4	NO
Bv0667c	glycosyltransferase family 26, related to beta-N-acetyl mannosaminuronic acid transferase	43.0	NO
Bv0668c	conserved hypothetical protein	45.8	NO
Bv0669c	putative serine acetyltransferase family protein	32.8	NO
Bv0670c	hypothetical protein	50.6	UNRESOLVED
Bv0671c	hypothetical protein	29.4	NOVEL
Bv0672c	putative UDP-N-acetylglucosamine 2-epimerase	45.3	NO
Bv0673c	conserved hypothetical protein	47.2	NO
Bv0674c	putative epimerase/dehydratase, involved in capsular polysaccharide biosynthesis	44.8	UNRESOLVED
Bv0675c	putative dehydratase	45.1	NO
Bv0676c	putative glycosyltransferase	29.0	UNRESOLVED
Bv0677c	hypothetical protein	28.8	NOVEL
Bv0678c	putative LPS biosynthesis related polysaccharide transporter/flippase	32.1	NO
Bv0679c	hypothetical protein	29.3	NOVEL
Bv0680c	putative UDP-ManNAc dehydrogenase	44.8	NO
Bv0681c	capsular polysaccharide biosythesis protein, putative	36.6	NO
Bv0682c	tyrosine-protein kinase ptk	40.7	NO
Bv0683c	polysaccharide export protein, BexD/CtrA/VexA family	45.5	NO
Bv0684c	undecaprenyl-phosphate alpha-N-acetylglucosaminyltransferase	42.8	NO
**Bv0685c**	**putative transcriptional regulator**	**46.9**	NO
**Bv0686c**	**integrase**	**51.2**	NO
**Locus 2**			
**Bv1492**	**integrase**	**51.8**	NO
Bv1493	hypothetical protein	42.3	NOVEL
**Bv1494**	**putative transcriptional regulator**	**46.9**	NO
Bv1495	undecaprenyl-phosphate alpha-N-acetylglucosaminyltransferase	42.4	NO
Bv1496	polysaccharide export protein, BexD/CtrA/VexA family	43.2	NO
Bv1497	tyrosine-protein kinase ptk	40.9	NO
Bv1498	capsular polysaccharide biosythesis protein, putative	35.6	NO
Bv1499	putative UDP-ManNAc dehydrogenase	44.9	NO
Bv1500	hypothetical protein	29.3	NOVEL
Bv1501	putative LPS biosynthesis related polysaccharide transporter/flippase	32.1	NO
Bv1502	hypothetical protein	28.8	NOVEL
Bv1503	putative glycosyltransferase	29.0	UNRESOLVED
Bv1504	putative dehydratase	45.1	NO
Bv1505	putative epimerase/dehydratase	44.8	UNRESOLVED
Bv1506	conserved hypothetical protein	47.2	UNRESOLVED
Bv1507	putative UDP-N-acetylglucosamine 2-epimerase	45.3	NO
Bv1508	hypothetical protein	29.4	NOVEL
Bv1509	hypothetical protein	50.6	UNRESOLVED
Bv1510	putative serine transferase family protein	32.8	NO
Bv1511	conserved hypothetical protein	45.8	NO
Bv1512	glycosyltransferase family 26, related to beta-N-acetyl mannosaminuronic acid transferase	43.0	NO
Bv1513	putative UDP-GlcNAc 2-epimerase	43.4	NO
**Locus 3**			
Bv1674	hypothetical protein	44.6	NOVEL

**Bv1675**	**transposase**	**50.5**	NO
**Bv1676**	**putative transcriptional regulator UpxY homolog**	**42.2**	NO
Bv1677	hypothetical protein	40.8	NO
Bv1678	putative LPS biosynthesis related polysaccharide transporter/flippase	35.5	NO
Bv1679	glycosyltransferase family 2, related to beta-glycosyltransferase	32.0	UNRESOLVED
Bv1680	hypothetical protein	28.7	NOVEL
Bv1681	glycosyltransferase family 4, related to alpha-glycosyltransferases	29.7	NO
Bv1682	glycosyltransferase family 4, related to alpha-glycosyltransferases	29.0	UNRESOLVED
Bv1683	glycosyltransferase family 14, related to beta-glycosyltransferases	28.8	UNRESOLVED
Bv1684	glycosyltransferase family 2, related to beta-glycosyltransferases	32.1	NO
Bv1685	undecaprenyl-phosphate alpha-N-acetylglucosaminyltransferase	37.8	NO
Bv1686	hypothetical protein	34.1	NOVEL
Bv1687	conserved hypothetical protein	39.1	NO
**Locus 4**			
**Bv2327**	**transposase**	**40.8**	UNRESOLVED
**Bv2328**	**putative transcriptional regulator UpxY homolog**	**41.0**	NO
Bv2329	conserved hypothetical protein	47.9	UNRESOLVED
Bv2330	polysaccharide export outer membrane protein	44.2	NO
Bv2331	putative EPS related membrane protein	43.9	NO
Bv2332	conserved hypothetical transmembrane protein; putative transmembrane protein	38.7	NO
Bv2333	F420H2-dehydrogenase, beta subunit	36.8	UNRESOLVED
Bv2334	conserved hypothetical protein	32.7	UNRESOLVED
Bv2335	conserved hypothetical protein	28.4	NO
Bv2336	glycosyltransferase family 2, related to beta-glycosyltransferases	26.5	UNRESOLVED
Bv2337	glycosyltransferase family 4, related to alpha-glycosyltransferases	34.5	UNRESOLVED
Bv2338	iron-sulfur cluster-binding protein/coenzyme F420-reducing hydrogenase, beta subunit, putative	42.8	UNRESOLVED
Bv2339	conserved hypothetical protein	40.3	UNRESOLVED
Bv2340	glycosyltransferase family 4, related to alpha-glycosyltransferases	40.9	NO
Bv2341	glycosyltransferase family 4, related to alpha-glycosyltransferases	43.5	NO
Bv2342	conserved hypothetical protein	37.9	UNRESOLVED
Bv2343	glucose-1-phosphate thymidyltransferase	46.1	NO
Bv2344	dTDP-4-dehydrorhamnose 3,5-epimerase	43.3	NO
Bv2345	dTDP-4-dehydrorhamnose reductase	41.7	NO
Bv2346	dTDP-glucose 4,6-dehydratase	40.9	NO
Bv2347	glycosyltransferase	48.9	NO
Bv2348c	***N-acetylmuramoyl-L-alanine amidase***	***54.8***	NO
Bv2349c	***conserved hypothetical protein***	***57.2***	NO
Bv2350c	***conserved hypothetical protein***	***56.1***	NO
Bv2351c	***conserved hypothetical protein***	***48.5***	NO
**Locus 5**			
Bv2585c	conserved hypothetical protein	42.3	UNRESOLVED
Bv2586c	mannose-1-phosphate guanylyltransferase	47.8	NO
Bv2587c	GDP-4-keto-6-deoxy-D-mannose-3, 5-epimerase-4-reductase	48.9	NO
Bv2588c	GDP-mannose 4,6-dehydratase	47.8	NO
Bv2589c	glycosyltransferase family 2, related to beta-glycosyltransferases	43.9	UNRESOLVED
**Bv2590c**	**glycosyltransferase family 2, related to beta-glycosyltransferases**	**41.9**	LGT-in
Bv2591c	putative acyltransferase transmembrane protein	32.2	NO
Bv2592c	acetyltransferase (isoleucine patch superfamily)	42.3	UNRESOLVED
Bv2593c	glycosyltransferase family 4, related to alpha-glycosyltransferases	35.3	UNRESOLVED

Bv2594c	hypothetical protein	32.2	NOVEL
Bv2595c	putative gluconate kinase	34.3	UNRESOLVED
**Bv2596c**	**acylneuraminate cytidylyltransferase, putative**	**32.8**	LGT-unresolved
**Bv2597c**	**putative aldolase**	**32.7**	LGT-out
Bv2598c	glycosyltransferase family 4, related to alpha-glycosyltransferases	27.9	NO
Bv2599c	hypothetical protein	29.1	NOVEL
Bv2600c	conserved hypothetical protein	30.7	UNRESOLVED
Bv2601c	glycosyltransferase family 4, distantly related to alpha-glycosyltransferases	31.6	UNRESOLVED
Bv2602c	putative O-acetyltransferase Cps9vM	28.7	UNRESOLVED
Bv2603c	conserved hypothetical transmembrane protein; putative transmembrane protein	33.8	NO
**Bv2604c**	**conserved hypothetical protein**	**44.7**	LGT-in
**Bv2605c**	**putative transcriptional regulator UpxY homolog**	**50.8**	NO
Bv2606	***conserved hypothetical protein***	***53.6***	NO
Bv2607	***hypothetical protein***	***38.9***	NOVEL
Bv2608	***conserved hypothetical protein***	***49.8***	UNRESOLVED
Bv2609	***N-acetylmuramoyl-L-alanine amidase***	***50.3***	NO
**Locus 6**			
**Bv2867**	**integrase**	**45.9**	NO
**Bv2868**	**conserved hypothetical protein, putative transcriptional regulatory protein**	**44.8**	NO
Bv2869	hypothetical protein	32.1	NOVEL
Bv2870	conserved hypothetical protein	36.7	UNRESOLVED
Bv2871	conserved hypothetical transmembrane protein; putative transmembrane protein	38.1	NO
Bv2872	F420H2:quinone oxidoreductase	41.4	UNRESOLVED
Bv2873	UDP-N-acetylglucosamine 2-epimerase	39.0	NO
Bv2874	conserved hypothetical protein	35.3	UNRESOLVED
Bv2875	conserved hypothetical protein	32.5	UNRESOLVED
**Bv2876**	**conserved hypothetical protein, putative polysaccharide polymerase**	**28.9**	LGT-in
Bv2877	glycosyltransferase family 2, related to beta-glycosyltransferases	31.6	UNRESOLVED
Bv2878	2-C-methyl-D-erythritol 4-phosphate cytidylyltransferase	38.2	NO
**Bv2879**	**putative epimerase/dehydratase**	**36.3**	LGT-in
Bv2880	lipopolysaccharide biosynthesis protein	36.6	UNRESOLVED
Bv2881	glycosyltransferase family 2, related to beta-glycosyltransferases	37.7	NO
Bv2882	putative UDP-galactose 4-epimerase	49.9	NO
Bv2883	conserved hypothetical protein	46.2	UNRESOLVED
Bv2884	conserved hypothetical protein	49.6	NO
Bv2885c	***putative N-acetylmuramoyl-L-alanine amidase***	***55.2***	NO
Bv2886	***hypothetical protein***	***40.8***	NOVEL
Bv2887c	***conserved hypothetical protein***	***54.9***	NO
Bv2888c	***hypothetical protein***	***55.8***	NOVEL
Bv2889c	***conserved hypothetical protein***	***55.7***	NO
Bv2890c	***conserved hypothetical protein***	***55.2***	NO
**Locus 7**			
Bv3356c	conserved hypothetical protein	42.6	UNRESOLVED
**Bv3357c**	**glycosyltransferase family 26, related to beta-glycosyltransferases**	**41.2**	LGT-in
Bv3358c	glycosyltransferase family 4, related to alpha-glycosyltransferases	34.5	UNRESOLVED
**Bv3359c**	**putative glycosyltransferase**	**28.9**	LGT-unresolved
Bv3360c	transposase	40.8	NO
Bv3361c	glycosyltransferase family 2, related to beta-glycosyltransferases	32.7	NO

Bv3362c	putative acetyl transferase	36.6	NO
Bv3363c	glycosyltransferase family 11, related to alpha-fucosyltransferases	29.9	UNRESOLVED
Bv3364c	hypothetical protein	29.0	NOVEL
Bv3365c	glycosyltransferase family 2, related to beta-glycosyltransferases	28.4	NO
Bv3366c	conserved hypothetical protein	30.4	NO
Bv3367	hypothetical protein	48.4	NOVEL
Bv3368c	hypothetical protein	46.7	NO
**Bv3369c**	**putative transcriptional regulator UpxY homolog**	**48.0**	NO
**Locus 8**			
Bv3808	***conserved hypothetical protein***	***55.0***	NO
Bv3809	***conserved hypothetical protein***	***54.7***	NO
Bv3810	***hypothetical protein***	***47.6***	NOVEL
Bv3811	***conserved hypothetical protein***	***56.2***	NO
Bv3812	***putative N-acetylmuramoyl-L-alanine amidase***	***55.6***	NO
Bv3813	***hypothetical protein***	***51.6***	NOVEL
Bv3814c	DNA primase/helicase	56.4	NO
Bv3815c	conserved hypothetical protein	43.8	NOVEL
Bv3816c	hypothetical protein	49.0	NOVEL
Bv3817c	conserved hypothetical protein, putative nucleic acid-binding protein, contains PIN domain	37.0	NO
Bv3818	hypothetical protein	31.5	NOVEL
Bv3819c	conserved hypothetical protein	44.4	UNRESOLVED
Bv3820c	glycosyltransferase family 26, related to beta-glycosyltransferases	47.2	NO
Bv3821c	glycosyltransferase family 4, related to alpha-glycosyltransferases	34.6	NO
Bv3822c	putative glycosyltransferase	34.6	UNRESOLVED
Bv3823c	glycosyltransferase family 4, related to alpha-glycosyltransferases	29.1	NO
Bv3824c	glycosyltransferase family 2, related to beta-glycosyltransferases	29.5	UNRESOLVED
Bv3825c	hypothetical protein	28.3	NOVEL
Bv3826c	glycosyltransferase family 4, related to alpha-glycosyltransferases	31.6	NO
Bv3827c	putative LPS biosynthesis related polysaccharide transporter/flippase	28.1	NO
**Bv3828c**	**putative transcriptional regulator UpxY homolog**	**51.3**	NO
**Locus 9**			
**Bv3908c**	**putative helicase**	**52.4**	LGT-in
Bv3909c	conserved hypothetical protein	36.9	UNRESOLVED
Bv3910c	conserved hypothetical protein	35.4	UNRESOLVED
Bv3911c	hypothetical protein	33.6	NOVEL
Bv3912c	conserved hypothetical protein	40.4	UNRESOLVED
Bv3913c	UDP-GlcNAc 4-epimerase	40.6	NO
Bv3914c	glycosyltransferase family 4, related to alpha-glycosyltransferases	37.2	NO
Bv3915c	glycosyltransferase family 4, distantly related to alpha-glycosyltransferases	30.3	UNRESOLVED
Bv3916c	hypothetical protein	29.1	NOVEL
**Bv3917c**	**transposase**	**41.8**	LGT-in
Bv3918c	hypothetical protein	45.4	NOVEL
Bv3919c	hypothetical protein	40.2	NOVEL
Bv3920c	hypothetical protein	38.1	NOVEL
Bv3921c	glycosyltransferase family 2, related to beta-glycosyltransferases	29.3	UNRESOLVED
**Bv3922c**	**putative pyridoxal phosphate-dependent enzyme apparently involved in regulation of cell wall biogenesis**	**37.1**	LGT-in
Bv3923c	glucose-1-phosphate cytidylyltransferase	35.0	NO
Bv3924c	conserved hypothetical protein	36.9	UNRESOLVED
Bv3925c	CDP-glucose 4,6-dehydratase	37.9	NO

**Bv3926c**	**putative pyridoxal phosphate-dependent enzyme apparently involved in regulation of cell wall biogenesis**	**33.9**	LGT-unresolved
Bv3927c	putative lipopolysaccharide biosynthesis related polysaccharide transporter/flippase	32.5	NOVEL
Bv3928c	hypothetical protein	34.5	NOVEL
**Bv3929c**	**conserved hypothetical protein**	**33.0**	LGT-in
Bv3930c	putative inositol monophosphatase CysQ	38.0	UNRESOLVED
**Bv3931c**	**putative 4-hydroxy-2-ketovalerate aldolase**	**38.9**	LGT-out
Bv3932c	putative CMP-KDO synthetase	36.3	UNRESOLVED
Bv3933c	nucleotidyltransferase family protein	41.0	NO
Bv3934c	CMP-N-acetylneuraminic acid synthetase	38.0	NO
Bv3935c	sialic acid synthase	40.4	UNRESOLVED
Bv3936c	putative carbamoylphosphate synthase, large subunit	35.4	UNRESOLVED
Bv3937c	diaminopimelate decarboxylase	33.0	UNRESOLVED
Bv3938c	UDP-N-acetylglucosamine 2-epimerase	37.6	UNRESOLVED
Bv3939c	putative aminotransferase	46.3	NO
Bv3940c	predicted nucleoside-diphosphate sugar epimerase	43.5	NO
Bv3941c	hypothetical protein	45.6	NOVEL
Bv3942	***conserved hypothetical protein***	***48.2***	NO
Bv3943	***conserved hypothetical protein***	***50.5***	UNRESOLVED
Bv3944	***putative N-acetylmuramoyl-L-alanine amidase***	***50.1***	NO
**Bv3945c**	**putative transcriptional regulator UpxY homolog**	**46.7**	NO

(C). *CPS* loci of *B. thetaiotaomicron* ATCC 29148

**Locus 1**			
**gi|29345443|ref|NP_808946.1|**	**integrase**	**52.6**	NO
**gi|29345444|ref|NP_808947.1|**	**putative transcriptional regulatory protein**	**42.8**	NOVEL
gi|29345445|ref|NP_808948.1|	hypothetical protein	32.0	NOVEL
gi|29345446|ref|NP_808949.1|	conserved hypothetical protein	45.5	UNRESOLVED
gi|29345447|ref|NP_808950.1|	hypothetical protein	47.3	NO
gi|29345448|ref|NP_808951.1|	F420H2:quinone oxidoreductase	47.4	NO
gi|29345449|ref|NP_808952.1|	conserved hypothetical protein	38.2	UNRESOLVED
gi|29345450|ref|NP_808953.1|	putative glycosyltransferase	29.9	NOVEL
gi|29345451|ref|NP_808954.1|	putative glycosyltransferase	36.8	UNRESOLVED
gi|29345452|ref|NP_808955.1|	putative polysaccharide biosynthesis protein	33.8	UNRESOLVED
gi|29345453|ref|NP_808956.1|	putative glycosyltransferase	44.7	UNRESOLVED
**gi|29345454|ref|NP_808957.1|**	**putative glycosyltransferase**	**38.6**	LGT-in
gi|29345455|ref|NP_808958.1|	hypothetical protein	36.1	NOVEL
gi|29345456|ref|NP_808959.1|	putative glycosyltransferase	42.3	UNRESOLVED
gi|29345457|ref|NP_808960.1|	putative glycosyltransferase	44.1	UNRESOLVED
gi|29345458|ref|NP_808961.1|	putative glycosyltransferase	44.1	UNRESOLVED
gi|29345459|ref|NP_808962.1|	hypothetical protein	40.3	NO
gi|29345460|ref|NP_808963.1|	glycosyltransferase	46.7	UNRESOLVED
**gi|29345461|ref|NP_808964.1|**	**putative glycosyltransferase**	**45.3**	LGT-in
**gi|29345462|ref|NP_808965.1|**	**putative glycosyltransferase**	**44.1**	LGT-unresolved
gi|29345463|ref|NP_808966.1|	putative glycosyltransferase	42.5	NO
gi|29345464|ref|NP_808967.1|	hypothetical protein	59.0	NO
gi|29345465|ref|NP_808968.1|	hypothetical protein	62.9	UNRESOLVED
gi|29345466|ref|NP_808969.1|	hypothetical protein	63.5	UNRESOLVED
gi|29345467|ref|NP_808970.1|	putative polysaccharide export protein	60.2	UNRESOLVED
**Locus 2**			
**gi|29345785|ref|NP_809288.1|**	**integrase**	**46.8**	NOVEL
**gi|29345786|ref|NP_809289.1|**	**putative transcriptional regulator**	**44.9**	NO
gi|29345787|ref|NP_809290.1|	conserved hypothetical protein	38.1	NO
gi|29345788|ref|NP_809291.1|	capsular polysaccharide biosynthesis protein capD	43.6	NO
gi|29345789|ref|NP_809292.1|	UDP-glucose 6-dehydrogenase	40.5	NO
gi|29345790|ref|NP_809293.1|	nucleotide sugar epimerase	39.9	NO
gi|29345791|ref|NP_809294.1|	capsular polysaccharide biosynthesis protein capD	42.8	NO
gi|29345792|ref|NP_809295.1|	capsular polysaccharide biosynthesis protein Cps4K	41.5	NO
gi|29345793|ref|NP_809296.1|	putative UDP-N-acetylglucosamine 2-epimerase	44.2	NO
gi|29345794|ref|NP_809297.1|	hypothetical protein	33.6	NOVEL
gi|29345795|ref|NP_809298.1|	conserved hypothetical protein	32.4	UNRESOLVED
gi|29345796|ref|NP_809299.1|	putative F420H2-dehydrogenase	36.3	UNRESOLVED
gi|29345797|ref|NP_809300.1|	NADH dehydrogenase subunit 2	31.6	NOVEL
gi|29345798|ref|NP_809301.1|	galactoside O-acetyltransferase	37.3	UNRESOLVED
gi|29345799|ref|NP_809302.1|	putative galactoside acetyltransferase	37.0	UNRESOLVED
gi|29345800|ref|NP_809303.1|	putative O-antigen export protein	30.8	UNRESOLVED
gi|29345801|ref|NP_809304.1|	putative protein involved in capsular polysaccharide biosynthesis	34.5	UNRESOLVED
**gi|29345802|ref|NP_809305.1|**	**lipopolysaccharide biosynthesis RfbU-related protein**	**40.3**	LGT-in
gi|29345803|ref|NP_809306.1|	serine acetyltransferase	39.9	NO
**gi|29345804|ref|NP_809307.1|**	**capsular polysaccharide biosynthesis glycosyltransferase**	**39.6**	LGT-unresolved

gi|29345805|ref|NP_809308.1|	hypothetical protein	34.2	NOVEL
gi|29345806|ref|NP_809309.1|	putative UDP-GlcNAc:undecaprenylphosphate GlcNAc-1-phosphate transferase	37.4	NO
gi|29345807|ref|NP_809310.1|	conserved hypothetical protein	38.7	NO
gi|29345808|ref|NP_809311.1|	polysaccharide export outer membrane protein	39.9	NO
gi|29345809|ref|NP_809312.1|	tyrosine-protein kinase ptk involved in exopolysaccharide biosynthesis	40.1	NOVEL
gi|29345810|ref|NP_809313.1|	putative tyrosine-protein kinase in capsular polysaccharide biosynthesis region	42.5	NO
gi|29345811|ref|NP_809314.1|	hypothetical protein	32.4	NOVEL
gi|29345812|ref|NP_809315.1|	hypothetical protein	44.7	NO
**Locus 3**			
**gi|29345872|ref|NP_809375.1|**	**putative transcriptional regulator**	**41.4**	NO
gi|29345873|ref|NP_809376.1|	glucose-1-phosphate thymidylyltransferase	39.4	NO
gi|29345874|ref|NP_809377.1|	dTDP-4-dehydrorhamnose 3,5-epimerase	36.3	NO
gi|29345875|ref|NP_809378.1|	dTDP-4-dehydrorhamnose reductase	32.3	NO
gi|29345876|ref|NP_809379.1|	dTDP-glucose 4,6-dehydratase	36.5	NO
gi|29345877|ref|NP_809380.1|	conserved hypothetical protein	33.2	NO
gi|29345878|ref|NP_809381.1|	putative F420H2-dehydrogenase 40 kDa subunit	35.0	UNRESOLVED
gi|29345879|ref|NP_809382.1|	hypothetical protein	29.7	NO
gi|29345880|ref|NP_809383.1|	hypothetical protein	32.0	NOVEL
gi|29345881|ref|NP_809384.1|	glycosyltransferase	33.0	NO
**gi|29345882|ref|NP_809385.1|**	**putative acyltransferase in colanic acid biosynthesis**	**35.4**	LGT-unresolved
gi|29345883|ref|NP_809386.1|	putative glycosyltransferase	33.2	NO
**gi|29345884|ref|NP_809387.1|**	**D-glycero-D-manno-heptose 1-phosphate kinase**	**39.0**	LGT-unresolved
**gi|29345885|ref|NP_809388.1|**	**putative phosphoheptose isomerase**	**37.9**	LGT-unresolved
gi|29345886|ref|NP_809389.1|	D-mannose-1-phosphate guanyltransferase	34.6	NO
gi|29345887|ref|NP_809390.1|	putative phosphatase	32.9	NO
gi|29345888|ref|NP_809391.1|	conserved hypothetical protein	30.7	UNRESOLVED
gi|29345889|ref|NP_809392.1|	putative glycosyltransferase	31.0	UNRESOLVED
gi|29345890|ref|NP_809393.1|	glycosyltransferase	36.5	NO
gi|29345891|ref|NP_809394.1|	polysaccharide export outer membrane protein	35.7	NO
gi|29345892|ref|NP_809395.1|	tyrosine-protein kinase ptk	40.1	NO
**Locus 4**			
**gi|29346005|ref|NP_809508.1|**	**integrase**	**43.1**	NO
**gi|29346006|ref|NP_809509.1|**	**putative transcriptional regulator**	**43.7**	NO
gi|29346007|ref|NP_809510.1|	conserved hypothetical protein	39.0	NO
gi|29346008|ref|NP_809511.1|	putative nucleoside-diphosphate sugar epimerases/dehydrase	43.1	NO
gi|29346009|ref|NP_809512.1|	UDP-glucose 6-dehydrogenase	40.6	NO
gi|29346010|ref|NP_809513.1|	nucleotide sugar epimerase	40.5	NO
gi|29346011|ref|NP_809514.1|	UDP-N-acetylglucosamine 2-epimerase	45.0	NO
**gi|29346012|ref|NP_809515.1|**	**UDP-N-acetyl-D-mannosaminuronic acid dehydrogenase**	**45.4**	LGT-in
gi|29346013|ref|NP_809516.1|	conserved hypothetical protein	34.6	UNRESOLVED
gi|29346014|ref|NP_809517.1|	putative coenzyme F420-reducing hydrogenase	34.6	UNRESOLVED
gi|29346015|ref|NP_809518.1|	putative polysaccharide export protein	32.9	UNRESOLVED
gi|29346016|ref|NP_809519.1|	hypothetical protein	32.0	NOVEL
**gi|29346017|ref|NP_809520.1|**	**serine O-acetyltransferase**	**34.2**	LGT-unresolved
gi|29346018|ref|NP_809521.1|	glycosyltransferase	33.8	UNRESOLVED
gi|29346019|ref|NP_809522.1|	glycosyltransferase	29.9	NO

gi|29346020|ref|NP_809523.1|	lipopolysaccharide biosynthesis protein, putative glycosyltransferase	36.7	UNRESOLVED
gi|29346021|ref|NP_809524.1|	glycosyltransferase	41.4	NO
gi|29346022|ref|NP_809525.1|	putative aminotransferase	43.5	NO
gi|29346023|ref|NP_809526.1|	putative membrane protein involved in polysaccharide export	37.9	NO
gi|29346024|ref|NP_809527.1|	putative tyrosine-protein kinase in cps region	42.1	NO
**Locus 5**			
gi|29346745|ref|NP_810248.1|	folylpolyglutamate synthase	44.3	NO
gi|29346746|ref|NP_810249.1|	putative translation initiation inhibitor	46.9	NO
gi|29346747|ref|NP_810250.1|	putative aminopeptidase C	46.4	UNRESOLVED
gi|29346748|ref|NP_810251.1|	dTDP-4-dehydrorhamnose 3,5-epimerase	34.6	NO
gi|29346749|ref|NP_810252.1|	undecaprenyl-phosphate alpha-N-acetylglucosaminyltransferase	32.5	NO
gi|29346750|ref|NP_810253.1|	putative lipopolysaccharide biosynthesis glycosyltransferase	30.4	NO
gi|29346751|ref|NP_810254.1|	UDP-glucose 6-dehydrogenase	36.5	NO
gi|29346752|ref|NP_810255.1|	putative UDP-glucuronic acid epimerase	35.2	NO
gi|29346753|ref|NP_810256.1|	putative capsule biosynthesis protein	29.6	NOVEL
gi|29346754|ref|NP_810257.1|	putative glycosyltransferase	32.9	UNRESOLVED
**gi|29346755|ref|NP_810258.1|**	**glycosyltransferase**	**30.0**	LGT-unresolved
gi|29346756|ref|NP_810259.1|	capsule biosynthesis protein capA	28.8	UNRESOLVED
gi|29346757|ref|NP_810260.1|	conserved hypothetical protein	28.9	UNRESOLVED
**gi|29346758|ref|NP_810261.1|**	**CDP-abequose synthase**	**29.9**	LGT-unresolved
gi|29346759|ref|NP_810262.1|	conserved hypothetical protein	36.5	NO
gi|29346760|ref|NP_810263.1|	CDP-glucose 4,6-dehydratase	36.0	NO
gi|29346761|ref|NP_810264.1|	glucose-1-phosphate cytidylyltransferase	35.4	NO
gi|29346762|ref|NP_810265.1|	putative glycosyltransferase HI1698	30.1	NO
gi|29346763|ref|NP_810266.1|	glycosyltransferase	28.5	UNRESOLVED
gi|29346764|ref|NP_810267.1|	putative flippase	28.5	NO
gi|29346765|ref|NP_810268.1|	hypothetical protein	35.4	NO
gi|29346766|ref|NP_810269.1|	putative capsule polysaccharide export protein	48.4	NO
gi|29346767|ref|NP_810270.1|	conserved hypothetical protein	43.4	NO
**gi|29346768|ref|NP_810271.1|**	**putative transcriptional regulator**	**42.8**	NO
**Locus 6**			
gi|29347047|ref|NP_810550.1|	***hypothetical protein***	***49.0***	NO
gi|29347048|ref|NP_810551.1|	***hypothetical protein***	***50.0***	NO
gi|29347049|ref|NP_810552.1|	***hypothetical protein***	***50.2***	NO
gi|29347050|ref|NP_810553.1|	***hypothetical protein***	***49.7***	NO
gi|29347051|ref|NP_810554.1|	***N-acetylmuramoyl-L-alanine amidase***	***49.5***	NO
gi|29347052|ref|NP_810555.1|	conserved hypothetical protein	40.2	NO
gi|29347053|ref|NP_810556.1|	hypothetical protein	26.3	NOVEL
gi|29347054|ref|NP_810557.1|	putative CPS biosynthesis glycosyltransferase	39.6	NO
gi|29347055|ref|NP_810558.1|	putative glycosyltransferase	34.1	NO
gi|29347056|ref|NP_810559.1|	putative glycosyltransferase	30.6	UNRESOLVED
gi|29347057|ref|NP_810560.1|	conserved hypothetical protein, putative integral membrane protein	28.6	NOVEL
gi|29347058|ref|NP_810561.1|	putative glycosyltransferase	31.4	NO
**gi|29347059|ref|NP_810562.1|**	**conserved hypothetical protein**	**37.2**	LGT-in
gi|29347060|ref|NP_810563.1|	putative teichoic acid biosynthesis protein F	29.8	NO
gi|29347061|ref|NP_810564.1|	pyrophosphorylase	34.7	NO

gi|29347062|ref|NP_810565.1|	lipopolysaccharide biosynthesis protein	34.3	NO
gi|29347063|ref|NP_810566.1|	hypothetical protein	36.4	NO
gi|29347064|ref|NP_810567.1|	polysialic acid transport protein kpsD precursor	46.5	NO
gi|29347065|ref|NP_810568.1|	conserved hypothetical protein	44.7	NO
**gi|29347066|ref|NP_810569.1|**	**putative transcriptional regulator**	**47.5**	NO
**gi|29347067|ref|NP_810570.1|**	**integrase**	**51.2**	NO
**Locus 7**			
gi|29347111|ref|NP_810614.1|	***hypothetical protein***	***36.0***	NOVEL
gi|29347112|ref|NP_810615.1|	***hypothetical protein***	***47.4***	NO
gi|29347113|ref|NP_810616.1|	***hypothetical protein***	***48.9***	NO
gi|29347114|ref|NP_810617.1|	***hypothetical protein***	***47.9***	NO
gi|29347115|ref|NP_810618.1|	***hypothetical protein***	***50.9***	NO
gi|29347116|ref|NP_810619.1|	***putative N-acetylmuramoyl-L-alanine amidase***	***47.8***	NO
gi|29347117|ref|NP_810620.1|	conserved hypothetical protein	35.4	NO
gi|29347118|ref|NP_810621.1|	putative capsular polysaccharide biosynthesis glycosyltransferase	39.7	UNRESOLVED
gi|29347119|ref|NP_810622.1|	putative glycosyltransferase	33.7	NO
gi|29347120|ref|NP_810623.1|	conserved hypothetical protein	30.0	UNRESOLVED
gi|29347121|ref|NP_810624.1|	hypothetical protein	28.8	NO
gi|29347122|ref|NP_810625.1|	glycosyltransferase	32.2	UNRESOLVED
gi|29347123|ref|NP_810626.1|	putative acylneuraminate cytidylyltransferase	34.3	UNRESOLVED
gi|29347124|ref|NP_810627.1|	sialic acid synthase (N-acetylneuraminate synthase)	34.7	UNRESOLVED
gi|29347125|ref|NP_810628.1|	putative polysaccharide biosynthesis protein	32.7	UNRESOLVED
**gi|29347126|ref|NP_810629.1|**	**putative teichoic acid biosynthesis protein B precursor**	**26.9**	LGT-in
gi|29347127|ref|NP_810630.1|	putative lipopolysaccharide biosynthesis protein	29.4	UNRESOLVED
gi|29347128|ref|NP_810631.1|	putative 2-aminoethylphosphonate pyruvate aminotransferase	36.9	NO
gi|29347129|ref|NP_810632.1|	sulfopyruvate decarboxylase subunit beta	36.7	NO
gi|29347130|ref|NP_810633.1|	phosphoenolpyruvate phosphomutase precursor	36.9	UNRESOLVED
gi|29347131|ref|NP_810634.1|	putative glucose-1-P-cytidylyltransferase	35.8	NO
gi|29347132|ref|NP_810635.1|	putative protein involved in capsular polysaccharide biosynthesis	39.5	NO
gi|29347133|ref|NP_810636.1|	polysialic acid transport protein kpsD precursor	46.4	NO
gi|29347134|ref|NP_810637.1|	conserved hypothetical protein	45.0	NO
**gi|29347135|ref|NP_810638.1|**	**putative transcriptional regulator**	**47.8**	NO
**gi|29347136|ref|NP_810639.1|**	**integrase**	**51.9**	NO
**Locus 8**			
gi|29348270|ref|NP_811773.1|	putative outer membrane protein	43.8	NO
gi|29348271|ref|NP_811774.1|	tyrosine-protein kinase ptk	41.9	NO
gi|29348272|ref|NP_811775.1|	putative polysaccharide export protein	38.3	UNRESOLVED
**gi|29348273|ref|NP_811776.1|**	**putative glycosyltransferase involved in lipopolysaccharide biosynthesis**	**40.9**	LGT-unresolved
**gi|29348274|ref|NP_811777.1|**	**putative glucose transferase**	**40.4**	LGT-unresolved
gi|29348275|ref|NP_811778.1|	putative glycosyltransferase	40.5	NO
gi|29348276|ref|NP_811779.1|	conserved hypothetical protein	36.7	NO
gi|29348277|ref|NP_811780.1|	putative glycosyltransferase yibD	36.3	UNRESOLVED
gi|29348278|ref|NP_811781.1|	putative succinyltransferase involved in succinoglycan biosynthesis	34.9	UNRESOLVED
gi|29348279|ref|NP_811782.1|	putative glycosyltransferase	38.4	NO
gi|29348280|ref|NP_811783.1|	putative glycosyltransferase	34.8	NO

**gi|29348281|ref|NP_811784.1|**	**putative capsular polysaccharide synthesis protein**	**36.9**	LGT-unresolved
gi|29348282|ref|NP_811785.1|	putative stress protein	35.6	UNRESOLVED
gi|29348283|ref|NP_811786.1|	putative colanic acid biosynthesis glycosyltransferase	34.6	UNRESOLVED
gi|29348284|ref|NP_811787.1|	serine O-acetyltransferase	35.9	UNRESOLVED
gi|29348285|ref|NP_811788.1|	putative glycosyltransferase	33.4	UNRESOLVED
gi|29348286|ref|NP_811789.1|	putative acetyltransferase	36.0	UNRESOLVED
gi|29348287|ref|NP_811790.1|	lipopolysaccharide biosynthesis protein	35.6	UNRESOLVED
gi|29348288|ref|NP_811791.1|	conserved hypothetical protein	34.1	NO
gi|29348289|ref|NP_811792.1|	putative nucleotide-sugar dehydratase	40.0	NO
gi|29348290|ref|NP_811793.1|	2-C-methyl-D-erythritol 4-phosphate cytidylyltransferase	39.3	UNRESOLVED
gi|29348291|ref|NP_811794.1|	glycosyltransferase	34.5	UNRESOLVED
**gi|29348292|ref|NP_811795.1|**	**phosphoribosylglycinamide formyltransferase**	**34.9**	LGT-unresolved
**gi|29348293|ref|NP_811796.1|**	**conserved hypothetical protein**	**32.5**	LGT-in
gi|29348294|ref|NP_811797.1|	nucleotide sugar transaminase	40.6	NO
**gi|29348295|ref|NP_811798.1|**	**putative transcriptional regulator**	**47.7**	NO

(D). *CPS* loci of *B. fragilis* NCTC 9343

**Locus 1**			
**gi|60680287|ref|YP_210431.1|**	**putative transcriptional regulator**	**43.0**	NO
gi|60680288|ref|YP_210432.1|	putative transcriptional regulator	49.1	NO
gi|60680289|ref|YP_210433.1|	putative capsular polysaccharide biosynthesis protein	42.0	NO
gi|60680290|ref|YP_210434.1|	putative DegT/DnrJ/EryC1/StrS aminotransferase family O-antigen related protein	36.2	UNRESOLVED
gi|60680291|ref|YP_210435.1|	putative capsular polysaccharide related hexapeptide transferase family protein	36.6	UNRESOLVED
**gi|60680292|ref|YP_210436.1|**	**putative sugar-phosphate nucleotidyl transferase**	**31.7**	LGT-in
gi|60680293|ref|YP_210437.1|	putative polysaccharide transporter/flippase	25.1	UNRESOLVED
gi|60680294|ref|YP_210438.1|	possible glycosyltransferase protein	29.4	UNRESOLVED
gi|60680295|ref|YP_210439.1|	possible O-antigen related transmembrane protein	26.6	NOVEL
gi|60680296|ref|YP_210440.1|	possible glycosyltransferase	30.6	NOVEL
gi|60680297|ref|YP_210441.1|	hypothetical protein	30.5	UNRESOLVED
**gi|60680298|ref|YP_210442.1|**	**possible capsular polysaccharide related protein**	**29.0**	LGT-unresolved
gi|60680299|ref|YP_210443.1|	putative UDP-ManNAc dehydrogenase	37.3	NO
gi|60680300|ref|YP_210444.1|	putative UDP-GlcNAc 2-epimerase	37.8	NO
**gi|60680301|ref|YP_210445.1|**	**putative transposase_11 DDE family protein**	**38.0**	LGT-unresolved
**gi|60680302|ref|YP_210446.1|**	**conserved hypothetical protein**	**34.9**	LGT-unresolved
**gi|60680303|ref|YP_210447.1|**	**putative glycosyltransferase protein**	**38.0**	LGT-unresolved
gi|60680304|ref|YP_210448.1|	putative UDP-galactose phosphate transferase	36.1	NO
**gi|60680305|ref|YP_210449.1|**	**putative acetyltransferase**	**45.1**	LGT-in
**Locus 2**			
**gi|60680552|ref|YP_210696.1|**	**putative transcriptional regulator**	**45.3**	UNRESOLVED
gi|60680553|ref|YP_210697.1|	putative transcriptional regulator	39.9	NO
gi|60680554|ref|YP_210698.1|	glucose-1-phosphate thymidyl transferase	38.5	NO
gi|60680555|ref|YP_210699.1|	dTDP-6-deoxy-D-glucose-3,5 epimerase	37.0	NO
gi|60680556|ref|YP_210700.1|	putative acetyl transferase	28.9	UNRESOLVED
gi|60680557|ref|YP_210701.1|	putative O-antigen flippase	28.0	NO
gi|60680558|ref|YP_210702.1|	putative fucosyl transferase	31.0	UNRESOLVED
gi|60680559|ref|YP_210703.1|	putative glycosyltransferase	28.1	UNRESOLVED
gi|60680560|ref|YP_210704.1|	putative acetyltransferase	30.7	UNRESOLVED
gi|60680561|ref|YP_210705.1|	putative polysaccharide polymerase	26.7	NOVEL
gi|60680562|ref|YP_210706.1|	putative glycosyltransferase	27.3	UNRESOLVED
gi|60680563|ref|YP_210707.1|	putative UDP-glucose-6 dehydrogenase	38.5	NO
gi|60680564|ref|YP_210708.1|	putative glycosyltransferase	34.8	UNRESOLVED
gi|60680565|ref|YP_210709.1|	putative deacetylase	35.8	UNRESOLVED
**gi|60680566|ref|YP_210710.1|**	**putative glycosyltransferase**	**36.5**	LGT-unresolved
gi|60680567|ref|YP_210711.1|	putative glycosyltransferase	32.8	NO
**gi|60680568|ref|YP_210712.1|**	**putative epimerase/dehydratase**	**39.9**	LGT-in
gi|60680569|ref|YP_210713.1|	putative phosphate transferase	37.4	NO
**Locus 3**			
**gi|60680881|ref|YP_211025.1|**	**putative LPS biosynthesis related transcriptional regulatory protein**	**42.0**	UNRESOLVED
gi|60680882|ref|YP_211026.1|	putative LPS biosynthesis related transcriptional regulatory protein	42.4	NO
gi|60680883|ref|YP_211027.1|	putative LPS biosynthesis related flippase	30.1	UNRESOLVED
gi|60680884|ref|YP_211028.1|	putative LPS biosynthesis related UDP-galactopyranose mutase	32.2	NO
gi|60680885|ref|YP_211029.1|	putative LPS biosynthesis related glycosyltransferase	30.3	NO

gi|60680886|ref|YP_211030.1|	putative LPS biosynthesis related integral membrane protein	27.7	NOVEL
**gi|60680887|ref|YP_211031.1|**	**unknown**	**30.0**	LGT-in
gi|60680888|ref|YP_211032.1|	putative glycosyltransferase	28.8	UNRESOLVED
**gi|60680889|ref|YP_211033.1|**	**putative glycosyltransferase**	**33.5**	LGT-unresolved
gi|60680890|ref|YP_211034.1|	putative DegT/DnrJ/EryC1/StrS family amino sugar synthetase	44.7	UNRESOLVED
gi|60680891|ref|YP_211035.1|	undecaprenyl-phosphate galactose phosphotransferase	38.9	NO
gi|60680892|ref|YP_211036.1|	conserved hypothetical protein	46.1	NO
gi|60680893|ref|YP_211037.1|	putative transmembrane sodium/sulfate transporter	47.7	NO
**Locus 4**			
**gi|60681050|ref|YP_211194.1|**	**putative transcriptional regulator**	**49.5**	NO
gi|60681051|ref|YP_211195.1|	putative transcriptional regulator	47.8	NO
gi|60681052|ref|YP_211196.1|	putative glucose-1-phosphate thymidyl transferase	44.7	NO
gi|60681053|ref|YP_211197.1|	putative dTDP-4-dehydrorhamnose 3,5 epimerase	37.2	NO
gi|60681054|ref|YP_211198.1|	hypothetical protein	37.1	NOVEL
gi|60681055|ref|YP_211199.1|	putative nucleotide-sugar dehydrogenase	43.8	NO
gi|60681056|ref|YP_211200.1|	putative dNTP-hexose dehydratase-epimerase	39.3	NO
gi|60681057|ref|YP_211201.1|	putative polysaccharide transporter/flippase	32.5	NO
gi|60681058|ref|YP_211202.1|	putative glucosyltransferase	30.0	UNRESOLVED
gi|60681059|ref|YP_211203.1|	putative glyocosyltransferase protein	31.9	UNRESOLVED
gi|60681060|ref|YP_211204.1|	hypothetical protein	34.5	NOVEL
gi|60681061|ref|YP_211205.1|	putative glycosyltransferase	31.1	UNRESOLVED
**gi|60681062|ref|YP_211206.1|**	**putative transmembrane protein**	**28.6**	LGT-in
gi|60681063|ref|YP_211207.1|	putative glycosyltransferase O-antigen related protein	33.2	UNRESOLVED
gi|60681064|ref|YP_211208.1|	putative lipopolysaccharide biosynthesis glycosyltransferase	30.5	NO
gi|60681065|ref|YP_211209.1|	putative DNTP-hexose dehydratase-epimerase	38.3	LGT-unresolved
gi|60681066|ref|YP_211210.1|	putative glycosyltransferase	40.9	NO
**Locus 5**			
**gi|60681383|ref|YP_211527.1|**	**putative LPS biosynthesis related transcriptional regulatory protein**	**40.6**	UNRESOLVED
gi|60681384|ref|YP_211528.1|	putative LPS biosynthesis related transcriptional regulatory protein	40.7	NO
gi|60681385|ref|YP_211529.1|	putative LPS biosynthesis related membrane protein	43.1	UNRESOLVED
gi|60681386|ref|YP_211530.1|	putative glucose-1-P-cytidylyltransferase	49.0	NO
gi|60681387|ref|YP_211531.1|	putative LPS biosynthesis related phosphoenolpyruvate phosphomutase	51.5	UNRESOLVED
gi|60681388|ref|YP_211532.1|	putative LPS biosynthesis related phosphoenolpyruvate decarboxylase	51.5	NO
gi|60681389|ref|YP_211533.1|	putative LPS biosynthesis related 2-aminoethylphosphonate pyruvate aminotransferase	47.2	NO
gi|60681390|ref|YP_211534.1|	putative LPS biosynthesis related flippase	25.9	NOVEL
gi|60681391|ref|YP_211535.1|	hypothetical protein	29.9	NOVEL
gi|60681392|ref|YP_211536.1|	putative LPS biosynthesis related alpha-1,2-fucosyltransferase	32.0	UNRESOLVED
gi|60681393|ref|YP_211537.1|	putative LPS biosynthesis related UDP-glucuronic acid epimerase	42.0	NO
gi|60681394|ref|YP_211538.1|	putative LPS biosynthesis related UDP-glucose dehydrogenase	42.1	NO
gi|60681395|ref|YP_211539.1|	putative LPS biosynthesis related glycosyltransferase	27.7	UNRESOLVED

gi|60681396|ref|YP_211540.1|	putative LPS biosynthesis related glycosyltransferase	29.7	UNRESOLVED
gi|60681397|ref|YP_211541.1|	putative LPS biosynthesis related polymerase	27.1	NOVEL
gi|60681398|ref|YP_211542.1|	putative LPS biosynthesis related glycosyltransferase	27.3	UNRESOLVED
gi|60681399|ref|YP_211543.1|	putative LPS biosynthesis related dehydratase	42.1	NO
**gi|60681400|ref|YP_211544.1|**	**putative LPS biosynthesis related epimerase**	**40.8**	LGT-in
gi|60681401|ref|YP_211545.1|	putative LPS biosynthesis related reductase	41.8	NO
gi|60681402|ref|YP_211546.1|	putative LPS biosynthesis related glycosyltransferase	34.5	NO
gi|60681403|ref|YP_211547.1|	putative LPS biosynthesis related dehydratase	38.7	UNRESOLVED
gi|60681404|ref|YP_211548.1|	putative LPS biosynthesis related UndPP-QuiNAc-P-transferase	40.9	NO
**Locus 6**			
gi|60682070|ref|YP_212214.1|	putative LPS biosynthesis related glycosyltransferase	37.8	NO
**gi|60682071|ref|YP_212215.1|**	**putative LPS biosynthesis related DNTP-hexose dehydratase-epimerase**	**37.8**	LGT-unresolved
**gi|60682072|ref|YP_212216.1|**	**putative LPS biosynthesis related glycosyltransferase**	**28.3**	LGT-unresolved
**gi|60682073|ref|YP_212217.1|**	**putative LPS biosynthesis related glycosyltransferase**	**29.0**	LGT-unresolved
**gi|60682074|ref|YP_212218.1|**	**putative LPS biosynthesis related glycosyltransferase**	**30.5**	LGT-unresolved
gi|60682075|ref|YP_212219.1|	putative LPS biosynthesis related polysaccharide polymerase	28.4	NOVEL
gi|60682076|ref|YP_212220.1|	putative LPS biosynthesis related glycosyltransferase	28.4	UNRESOLVED
gi|60682077|ref|YP_212221.1|	putative LPS biosynthesis related polysaccharide transporter/flippase	30.3	NO
gi|60682078|ref|YP_212222.1|	putative LPS biosynthesis related glycosyltransferase	28.5	UNRESOLVED
**gi|60682079|ref|YP_212223.1|**	**DNTP-hexose dehydratase-epimerase**	**33.4**	LGT-unresolved
**gi|60682080|ref|YP_212224.1|**	**putative LPS biosynthesis related DNTP-hexose dehydratase-epimerase**	**31.6**	LGT-unresolved
**gi|60682081|ref|YP_212225.1|**	**putative LPS biosynthesis related DNTP-hexose dehydratase-epimerase**	**40.3**	LGT-unresolved
gi|60682082|ref|YP_212226.1|	putative LPS biosynthesis-related sugar-phosphate nucleotidyltransferase	36.6	NO
**gi|60682083|ref|YP_212227.1|**	**putative LPS biosynthesis related DNTP-hexose dehydratase-epimerase**	**39.7**	LGT-in
gi|60682084|ref|YP_212228.1|	putative transcriptional regulatory protein	38.9	NO
**gi|60682085|ref|YP_212229.1|**	**putative transcriptional regulatory protein**	**42.2**	UNRESOLVED
**Locus 7**			
**gi|60682261|ref|YP_212405.1|**	**putative transcriptional regulator (pseudogene)**	**59.3**	NO
gi|60682262|ref|YP_212406.1|	conserved hypothetical transmembrane protein; putative transmembrane protein	35.5	NO
gi|60682263|ref|YP_212407.1|	putative transmembrane protein	34.9	NO
gi|60682264|ref|YP_212408.1|	conserved hypothetical protein	38.3	UNRESOLVED
gi|60682265|ref|YP_212409.1|	conserved hypothetical protein	39.9	UNRESOLVED
gi|60682266|ref|YP_212410.1|	putative glycosyltransferase protein	38.3	UNRESOLVED
gi|60682267|ref|YP_212411.1|	putative glycosyltransferase	38.7	NO
gi|60682268|ref|YP_212412.1|	putative glycosyltransferase	34.4	UNRESOLVED
gi|60682269|ref|YP_212413.1|	putative acetyltransferase	37.7	UNRESOLVED
gi|60682270|ref|YP_212414.1|	possible glycosyltransferase	35.3	NOVEL
gi|60682271|ref|YP_212415.1|	putative transmembrane protein	34.1	NOVEL
gi|60682272|ref|YP_212416.1|	putative glycosyltransferase protein	34.3	UNRESOLVED
gi|60682273|ref|YP_212417.1|	putative acyltransferase transmembrane protein	35.2	NO
gi|60682274|ref|YP_212418.1|	putative acetyltransferase	32.3	UNRESOLVED
**gi|60682275|ref|YP_212419.1|**	**putative glycosyltransferase**	**33.4**	LGT-unresolved

gi|60682276|ref|YP_212420.1|	putative 4Fe-4S binding protein	36.3	UNRESOLVED
gi|60682277|ref|YP_212421.1|	conserved hypothetical protein	35.2	UNRESOLVED
gi|60682278|ref|YP_212422.1|	putative glycosyltransferase protein	37.4	NO
gi|60682279|ref|YP_212423.1|	putative acetyltransferase/glucosyltransferase	45.1	NO
gi|60682280|ref|YP_212424.1|	putative exported protein	43.5	NO
gi|60682281|ref|YP_212425.1|	putative exported protein	49.9	NO
gi|60682282|ref|YP_212426.1|	putative exported protein	46.8	NO
gi|60682283|ref|YP_212427.1|	putative exported protein	45.5	NOVEL
**gi|60682284|ref|YP_212428.1|**	**putative transmembrane protein**	**39.2**	LGT-in
gi|60682285|ref|YP_212429.1|	hypothetical transmembrane protein	45.3	NO
gi|60682286|ref|YP_212430.1|	putative exported protein	43.4	UNRESOLVED
gi|60682287|ref|YP_212431.1|	conserved hypothetical protein	45.5	NO
gi|60682288|ref|YP_212432.1|	putative nucleotidyltransferase	43.6	NO
**Locus 8**			
gi|60682911|ref|YP_213055.1|	hypothetical protein	?	NOVEL
gi|60682912|ref|YP_213056.1|	putative LPS biosynthesis related conserved hypothetical protein	45.5	UNRESOLVED
gi|60682913|ref|YP_213057.1|	putative LPS biosynthesis related dTDP-4-dehydrorhamnose 3,5-epimerase	42.0	NO
gi|60682914|ref|YP_213058.1|	putative LPS biosynthesis related glucose-1-phosphate thymidylyltransferase	43.2	NO
gi|60682915|ref|YP_213059.1|	putative LPS biosynthesis related glycosyltransferase	31.7	NO
gi|60682916|ref|YP_213060.1|	putative LPS biosynthesis related glycosyltransferase	27.7	UNRESOLVED
gi|60682917|ref|YP_213061.1|	putative LPS biosynthesis related polysaccharide polymerase	25.1	NOVEL
gi|60682918|ref|YP_213062.1|	putative LPS biosynthesis related glycosyltransferase	31.5	UNRESOLVED
gi|60682919|ref|YP_213063.1|	putative LPS biosynthesis related glycosyltransferase	28.6	UNRESOLVED
**gi|60682920|ref|YP_213064.1|**	**putative LPS biosynthesis related glycosyltransferase**	**29.3**	UNRESOLVED
**gi|60682921|ref|YP_213065.1|**	**putative LPS biosynthesis related glycosyltransferase**	**29.0**	LGT-in
**gi|60682922|ref|YP_213066.1|**	**conserved hypothetical protein**	**29.0**	LGT-in
**gi|60682923|ref|YP_213067.1|**	**hypothetical protein**	**31.2**	LGT-unresolved
**gi|60682924|ref|YP_213068.1|**	**putative LPS biosynthesis related polysaccharide transporter**	**28.5**	LGT-in
gi|60682925|ref|YP_213069.1|	putative LPS biosynthesis related glycosyltransferase	48.8	NO
gi|60682926|ref|YP_213070.1|	putative LPS biosynthesis related transcriptional regulatory protein	51.6	NO
**gi|60682927|ref|YP_213071.1|**	**putative LPS biosynthesis related transcriptional regulatory protein**	**52.2**	NO
**Locus 9**			
**gi|60683129|ref|YP_213273.1|**	**DNTP-hexose dehydratase-epimerase**	**36.8**	LGT-out
gi|60683130|ref|YP_213274.1|	putative glycosyltransferase protein	31.6	NO
gi|60683131|ref|YP_213275.1|	putative glycosyltransferase protein	33.3	UNRESOLVED
gi|60683132|ref|YP_213276.1|	putative polysaccharide polymerase	27.8	UNRESOLVED
gi|60683133|ref|YP_213277.1|	putative glycosyltransferase protein	30.3	UNRESOLVED
gi|60683134|ref|YP_213278.1|	putative alpha-1,2-fucosyltransferase	31.3	UNRESOLVED
gi|60683135|ref|YP_213279.1|	putative transferase	29.6	UNRESOLVED
gi|60683136|ref|YP_213280.1|	putative O-acetyl transferase (capsular polysaccharide synthesis enzyme o-acetyl transferase)	31.4	UNRESOLVED
**gi|60683137|ref|YP_213281.1|**	**hypothetical protein**	**29.6**	LGT-in
**gi|60683138|ref|YP_213282.1|**	**possible flippase**	**29.4**	LGT-out
**gi|60683139|ref|YP_213283.1|**	**conserved hypothetical protein**	**35.8**	LGT-in

**gi|60683140|ref|YP_213284.1|**	**putative nucleotidyltransferase**	**32.9**	LGT-in
gi|60683141|ref|YP_213285.1|	putative nucleotidyltransferase	32.5	UNRESOLVED
**gi|60683142|ref|YP_213286.1|**	**putative haloacid dehalogenase-like hydrolase**	**35.9**	LGT-in
**gi|60683143|ref|YP_213287.1|**	**conserved hypothetical protein**	**32.9**	LGT-in
gi|60683144|ref|YP_213288.1|	putative transcriptional regulator	46.3	NO
**gi|60683145|ref|YP_213289.1|**	**putative transcriptional regulator**	**46.9**	NO

(E). *CPS* loci of *B. fragilis* YCH 46

**Locus 1**			
**gi|53712096|ref|YP_098088.1|**	**putative transcriptional regulator UpxY homolog**	**43.2**	NO
gi|53712097|ref|YP_098089.1|	conserved hypothetical protein UpxZ homolog	49.7	NO
gi|53712098|ref|YP_098090.1|	glucose-1-phosphate thymidyltransferase	40.5	NO
gi|53712099|ref|YP_098091.1|	dTDP-4-dehydrorhamnose 3,5-epimerase	33.9	NO
gi|53712100|ref|YP_098092.1|	dTDP-glucose 4,6-dehydratase	36.9	NO
gi|53712101|ref|YP_098093.1|	O-antigen repeat unit transporter	34.4	UNRESOLVED
**gi|53712102|ref|YP_098094.1|**	**putative dNDP-4-keto-6-deoxy-glucose-2,3- dehydratase**	**34.7**	LGT-in
**gi|53712103|ref|YP_098095.1|**	**probable NDP-hexose-3-ketoreductase**	**37.9**	LGT-unresolved
**gi|53712104|ref|YP_098096.1|**	**aminotransferase**	**37.1**	LGT-in
gi|53712105|ref|YP_098097.1|	hypothetical protein	32.4	NOVEL
gi|53712106|ref|YP_098098.1|	probable glycosyltransferase	30.3	NO
gi|53712107|ref|YP_098099.1|	probable glycosyltransferase	33.3	UNRESOLVED
gi|53712108|ref|YP_098100.1|	putative polysaccharide polymerase	32.1	UNRESOLVED
gi|53712109|ref|YP_098101.1|	putative glycosyltransferase	32.8	UNRESOLVED
gi|53712110|ref|YP_098102.1|	putative undecaprenyl-phosphate glycosyl-1-phosphate transferase	37.3	NO
gi|53712111|ref|YP_098103.1|	probable serine O-acetyltransferase	32.7	NO
gi|53712112|ref|YP_098104.1|	putative acyl carrier protein	31.2	UNRESOLVED
gi|53712113|ref|YP_098105.1|	probable 3-oxoacyl-[acyl carrier protein] synthase	37.3	NO
gi|53712114|ref|YP_098106.1|	3-oxoacyl-[acyl-carrier-protein] reductase	36.9	NO
gi|53712115|ref|YP_098107.1|	3-oxoacyl-[acyl-carrier-protein] synthase	37.4	UNRESOLVED
gi|53712116|ref|YP_098108.1|	putative lactoylglutathione lyase	35.3	NO
gi|53712117|ref|YP_098109.1|	conserved hypothetical protein	35.2	NO
gi|53712118|ref|YP_098110.1|	acyl carrier protein	36.6	NO
gi|53712119|ref|YP_098111.1|	conserved hypothetical protein	34.8	UNRESOLVED
gi|53712120|ref|YP_098112.1|	putative aminotransferase	48.8	NO
**Locus 2**			
**gi|53712384|ref|YP_098376.1|**	**putative transcriptional regulator UpxY homolog**	**46.5**	NOVEL
gi|53712385|ref|YP_098377.1|	conserved hypothetical protein UpxZ homolog	39.9	NO
gi|53712386|ref|YP_098378.1|	glucose-1-phosphate thymidyltransferase	38.1	NO
gi|53712387|ref|YP_098379.1|	putative flippase	28.9	UNRESOLVED
gi|53712388|ref|YP_098380.1|	conserved hypothetical protein	30.2	NOVEL
**gi|53712389|ref|YP_098381.1|**	**putative glycosyltransferase**	**31.3**	LGT-in
gi|53712390|ref|YP_098382.1|	putative polymerase	28.3	NOVEL
gi|53712391|ref|YP_098383.1|	UDP-ManNAc dehydrogenase	34.8	NO
gi|53712392|ref|YP_098384.1|	UDP-GlcNAc 2-epimerase	35.7	NO
gi|53712393|ref|YP_098385.1|	putative glycosyltransferase	30.4	UNRESOLVED
gi|53712394|ref|YP_098386.1|	conserved hypothetical protein	32.9	NO
gi|53712395|ref|YP_098387.1|	conserved hypothetical protein	32.2	UNRESOLVED
gi|53712396|ref|YP_098388.1|	putative aminotransferase	32.9	NO
**gi|53712397|ref|YP_098389.1|**	**putative glycosyltransferase**	**29.6**	LGT-unresolved
gi|53712398|ref|YP_098390.1|	putative dehydratase	41.6	NO
gi|53712399|ref|YP_098391.1|	putative epimerase	40.6	UNRESOLVED
gi|53712400|ref|YP_098392.1|	putative epimerase	43.5	UNRESOLVED
**gi|53712401|ref|YP_098393.1|**	**putative glycosyltransferase**	**37.6**	LGT-unresolved
gi|53712402|ref|YP_098394.1|	putative undecaprenyl-phosphate glycosyl-1-phosphate transferase	39.4	NO
**gi|53712403|ref|YP_098395.1|**	**putative acetyltransferase**	**44.6**	LGT-in
gi|53712404|ref|YP_098396.1|	putative aminotransferase	50.4	NO
gi|53712405|ref|YP_098397.1|	conserved hypothetical protein	38.3	NOVEL

**Locus 3**			
**gi|53712721|ref|YP_098713.1|**	**putative transcriptional regulatory protein UpxY homolog**	**41.2**	UNRESOLVED
gi|53712722|ref|YP_098714.1|	conserved hypothetical protein UpxZ homolog	42.0	NO
gi|53712723|ref|YP_098715.1|	predicted nucleoside-diphosphate sugar epimerase	41.8	NO
gi|53712724|ref|YP_098716.1|	putative aminotransferase	44.4	NO
gi|53712725|ref|YP_098717.1|	putative acyl carrier protein	28.8	NO
gi|53712726|ref|YP_098718.1|	conserved hypothetical protein	31.7	NO
gi|53712727|ref|YP_098719.1|	3-oxoacyl-[acyl-carrier-protein] reductase	33.5	NO
**gi|53712728|ref|YP_098720.1|**	**putative acetyl transferase**	**31.9**	UNRESOLVED
**gi|53712729|ref|YP_098721.1|**	**mannose-1-phosphate guanyltransferase**	**31.3**	LGT-in
gi|53712730|ref|YP_098722.1|	hypothetical protein	27.9	NOVEL
gi|53712731|ref|YP_098723.1|	hypothetical protein	28.5	NOVEL
gi|53712732|ref|YP_098724.1|	oligosaccharide repeat unit transporter	28.3	NOVEL
gi|53712733|ref|YP_098725.1|	putative capsular polysaccharide polymerase	27.6	UNRESOLVED
gi|53712734|ref|YP_098726.1|	putative UDP-glucose-6 dehydrogenase	33.8	NO
**gi|53712735|ref|YP_098727.1|**	**glycosyltransferase**	**31.6**	LGT-unresolved
gi|53712736|ref|YP_098728.1|	putative glycosyltransferase	34.7	UNRESOLVED
gi|53712737|ref|YP_098729.1|	aminotransferase	44.5	UNRESOLVED
gi|53712738|ref|YP_098730.1|	putative undecaprenyl-phosphate galactose phosphotransferase	39.1	NO
**Locus 4**			
**gi|53712820|ref|YP_098812.1|**	**putative transcriptional regulator UpxY homolog**	**48.8**	NO
gi|53712821|ref|YP_098813.1|	conserved hypothetical protein UpxZ homolog	47.8	NO
**gi|53712822|ref|YP_098814.1|**	**putative dehydrogenase**	**36.2**	LGT-unresolved
**gi|53712823|ref|YP_098815.1|**	**conserved hypothetical protein**	**40.2**	LGT-unresolved
gi|53712824|ref|YP_098816.1|	putative acyl carrier protein	36.2	UNRESOLVED
gi|53712825|ref|YP_098817.1|	conserved hypothetical protein	36.2	NO
gi|53712826|ref|YP_098818.1|	glucose-1-phosphate cytidylyltransferase	36.8	NO
**gi|53712827|ref|YP_098819.1|**	**hypothetical protein**	**35.8**	LGT-in
**gi|53712828|ref|YP_098820.1|**	**CDP-glucose-4,6-dehydratase**	**40.8**	LGT-unresolved
gi|53712829|ref|YP_098821.1|	putative dTDP-glucose 4,6-dehydratase	32.2	UNRESOLVED
**gi|53712830|ref|YP_098822.1|**	**conserved hypothetical protein**	**33.3**	LGT-in
gi|53712831|ref|YP_098823.1|	2,5-diketo-D-gluconic acid reductase	38.0	UNRESOLVED
gi|53712832|ref|YP_098824.1|	hypothetical protein	34.2	NOVEL
gi|53712833|ref|YP_098825.1|	putative succinyltransferase	31.2	NO
gi|53712834|ref|YP_098826.1|	conserved hypothetical protein	36.2	UNRESOLVED
gi|53712835|ref|YP_098827.1|	glycosyltransferase	34.7	UNRESOLVED
gi|53712836|ref|YP_098828.1|	hypothetical protein	37.3	NO
gi|53712837|ref|YP_098829.1|	probable O-antigen polymerase	26.8	NOVEL
gi|53712838|ref|YP_098830.1|	glycosyltransferase	32.2	UNDECIDED
**gi|53712839|ref|YP_098831.1|**	**putative glycosyltransferase**	**35.0**	LGT-in
**gi|53712840|ref|YP_098832.1|**	**glycosyltransferase**	**31.9**	LGT-unresolved
gi|53712841|ref|YP_098833.1|	conserved hypothetical protein	32.8	NO
**gi|53712842|ref|YP_098834.1|**	**putative UDP-galactose 4-epimerase**	**37.8**	LGT-unresolved
gi|53712843|ref|YP_098835.1|	putative UndPP-QuiNAc-P-transferase	40.8	NO
**Locus 5**			
**gi|53713117|ref|YP_099109.1|**	**putative transcriptional regulatory protein UpxY homolog**	**40.4**	UNRESOLVED
gi|53713118|ref|YP_099110.1|	conserved hypothetical protein UpxZ homolog	40.9	NO

gi|53713119|ref|YP_099111.1|	conserved hypothetical protein	43.0	UNRESOLVED
gi|53713120|ref|YP_099112.1|	putative glucose-1-P-cytidylyltransferase	49.9	NO
gi|53713121|ref|YP_099113.1|	putative phosphoenolpyruvate phosphomutase	50.6	UNRESOLVED
gi|53713122|ref|YP_099114.1|	putative phosphoenolpyruvate decarboxylase	51.4	NO
gi|53713123|ref|YP_099115.1|	putative 2-aminoethylphosphonate pyruvate aminotransferase	47.0	NO
gi|53713124|ref|YP_099116.1|	putative flippase	25.9	NOVEL
gi|53713125|ref|YP_099117.1|	conserved hypothetical protein	30.0	NOVEL
gi|53713126|ref|YP_099118.1|	putative alpha-1,2-fucosyltransferase	32.0	UNRESOLVED
gi|53713127|ref|YP_099119.1|	putative UDP-glucuronic acid epimerase	41.9	NO
gi|53713128|ref|YP_099120.1|	putative UDP-glucose dehydrogenase	42.1	NO
gi|53713129|ref|YP_099121.1|	putative glycosyltransferase	27.7	UNRESOLVED
gi|53713130|ref|YP_099122.1|	putative glycosyltransferase	29.7	UNRESOLVED
gi|53713131|ref|YP_099123.1|	putative polymerase	27.1	NOVEL
gi|53713132|ref|YP_099124.1|	putative glycosyltransferase	27.4	UNRESOLVED
gi|53713133|ref|YP_099125.1|	putative dehydratase	42.1	NO
**gi|53713134|ref|YP_099126.1|**	**putative epimerase**	**40.8**	LGT-in
gi|53713135|ref|YP_099127.1|	putative reductase	41.9	NO
gi|53713136|ref|YP_099128.1|	putative glycosyltransferase	34.5	NO
gi|53713137|ref|YP_099129.1|	putative dehydratase	38.7	UNRESOLVED
gi|53713138|ref|YP_099130.1|	putative UndPP-QuiNAc-P-transferase	41.0	NO
**Locus 6**			
gi|53713857|ref|YP_099849.1|	putative UndPP-QuiNAc-P-transferase	37.9	NO
**gi|53713858|ref|YP_099850.1|**	**putative UDP-galactose 4-epimerase**	**38.2**	LGT-unresolved
**gi|53713859|ref|YP_099851.1|**	**putative glycosyltransferase**	**36.6**	LGT-unresolved
gi|53713860|ref|YP_099852.1|	putative reductase	41.0	NO
**gi|53713861|ref|YP_099853.1|**	**putative epimerase**	**40.8**	LGT-in
gi|53713862|ref|YP_099854.1|	putative dehydratase	41.8	NO
gi|53713863|ref|YP_099855.1|	putative glycosyltransferase	27.2	UNRESOLVED
gi|53713864|ref|YP_099856.1|	glycosyltransferase	28.6	UNRESOLVED
gi|53713865|ref|YP_099857.1|	alpha-1,2-fucosyltransferase	29.0	NO
gi|53713866|ref|YP_099858.1|	hypothetical protein	28.3	NOVEL
gi|53713867|ref|YP_099859.1|	glycosyltransferase	27.9	UNRESOLVED
**gi|53713868|ref|YP_099860.1|**	**putative alcohol dehydrogenase**	**33.8**	LGT-unresolved
gi|53713869|ref|YP_099861.1|	putative phosphoenolpyruvate decarboxylase	36.2	UNRESOLVED
gi|53713870|ref|YP_099862.1|	putative phosphoenolpyruvate phosphomutase	37.1	UNRESOLVED
gi|53713871|ref|YP_099863.1|	putative cholinephosphotransferase	33.3	UNRESOLVED
**gi|53713872|ref|YP_099864.1|**	**putative carbamoylphosphate synthase large subunit short form**	**31.7**	LGT-unresolved
gi|53713873|ref|YP_099865.1|	aminotransferase	40.4	NO
gi|53713874|ref|YP_099866.1|	capsular polysaccharide repeat unit transporter	39.1	UNRESOLVED
gi|53713875|ref|YP_099867.1|	glucose-1-phosphate thymidylyltransferase	43.2	NO
gi|53713876|ref|YP_099868.1|	conserved hypothetical protein UpxZ homolog	38.9	NO
**gi|53713877|ref|YP_099869.1|**	**putative transcriptional regulatory protein UpxY homolog**	**42.2**	UNRESOLVED
**Locus 7**			
**gi|53714067|ref|YP_100059.1|**	**putative transcriptional regulator UpxY homolog**	**59.3**	NO
gi|53714068|ref|YP_100060.1|	putative flippase	38.8	UNRESOLVED
gi|53714069|ref|YP_100061.1|	hypothetical protein	33.2	NOVEL
gi|53714070|ref|YP_100062.1|	conserved hypothetical protein	33.8	UNRESOLVED
gi|53714071|ref|YP_100063.1|	putative glycosyltransferase	33.8	UNRESOLVED

gi|53714072|ref|YP_100064.1|	O-actetyl transferase related protein	33.5	UNRESOLVED
gi|53714073|ref|YP_100065.1|	conserved hypothetical protein	36.2	UNRESOLVED
gi|53714074|ref|YP_100066.1|	hypothetical protein	35.8	NOVEL
gi|53714075|ref|YP_100067.1|	hypothetical protein	32.0	NO
**gi|53714076|ref|YP_100068.1|**	**putative glycosyltransferase**	**32.1**	LGT-unresolved
**gi|53714077|ref|YP_100069.1|**	**serine acetyltransferase**	**34.2**	LGT-unresolved
gi|53714078|ref|YP_100070.1|	putative acetylglucosaminyltransferase	33.0	UNRESOLVED
gi|53714079|ref|YP_100071.1|	putative truncated alpha-1,2-fucosyltransferase	29.7	NOVEL
Inserted by a CTn			
gi|53714213|ref|YP_100205.1|	putative truncated alpha-1,2-fucosyltransferase	32.7	NOVEL
gi|53714214|ref|YP_100206.1|	putative glycosyltransferase	31.0	UNRESOLVED
gi|53714215|ref|YP_100207.1|	putative glycosyltransferase	35.0	UNRESOLVED
gi|53714216|ref|YP_100208.1|	putative glycosyltransferase	35.7	NO
gi|53714217|ref|YP_100209.1|	putative beta-1,4-N-acetyl- mannosaminyltransferase	44.9	NO
gi|53714218|ref|YP_100210.1|	conserved hypothetical protein	43.1	NO
gi|53714219|ref|YP_100211.1|	conserved hypothetical protein	49.9	NO
gi|53714220|ref|YP_100212.1|	conserved hypothetical protein	47.1	NO
gi|53714221|ref|YP_100213.1|	hypothetical protein	45.3	NOVEL
**gi|53714222|ref|YP_100214.1|**	**conserved hypothetical protein**	**39.0**	LGT-in
gi|53714223|ref|YP_100215.1|	conserved hypothetical protein	45.0	NO
gi|53714224|ref|YP_100216.1|	major outer membrane protein OmpA	43.1	UNRESOLVED
gi|53714225|ref|YP_100217.1|	conserved hypothetical protein	45.5	NO
gi|53714226|ref|YP_100218.1|	mannose-1-phosphate guanylyltransferase	43.3	NO
**Locus 8**			
gi|53714929|ref|YP_100921.1|	conserved hypothetical protein	36.3	UNRESOLVED
gi|53714930|ref|YP_100922.1|	conserved hypothetical protein	41.1	NO
gi|53714931|ref|YP_100923.1|	putative capsular polysaccharide biosynthesis protein	45.0	NO
gi|53714932|ref|YP_100924.1|	putative UDP-galactose 4-epimerase	37.1	UNRESOLVED
gi|53714933|ref|YP_100925.1|	putative glycosyltransferase	32.2	UNRESOLVED
gi|53714934|ref|YP_100926.1|	putative glycosyltransferase	31.5	UNRESOLVED
**gi|53714935|ref|YP_100927.1|**	**glycosyltransferase**	**28.4**	LGT-unresolved
gi|53714936|ref|YP_100928.1|	putative O-antigen polymerase	26.9	NOVEL
gi|53714937|ref|YP_100929.1|	putative glycosyltransferase	32.4	UNRESOLVED
**gi|53714938|ref|YP_100930.1|**	**putative O-acetyltransferase**	**32.7**	LGT-in
gi|53714939|ref|YP_100931.1|	putative glycosyltransferase	30.0	UNRESOLVED
gi|53714940|ref|YP_100932.1|	putative glycosyltransferase	34.3	NOVEL
gi|53714941|ref|YP_100933.1|	UDP-GlcNAc 2-epimerase	35.6	NO
**gi|53714942|ref|YP_100934.1|**	**putative glycosyltransferase**	**27.5**	LGT-unresolved
gi|53714943|ref|YP_100935.1|	putative flippase	31.8	UNRESOLVED
gi|53714944|ref|YP_100936.1|	putative aminotransferase	35.2	NO
**gi|53714945|ref|YP_100937.1|**	**conserved hypothetical protein**	**30.9**	LGT-in
gi|53714946|ref|YP_100938.1|	conserved hypothetical protein	35.6	NO
gi|53714947|ref|YP_100939.1|	conserved hypothetical protein	36.1	UNRESOLVED
gi|53714948|ref|YP_100940.1|	conserved hypothetical protein	33.1	NO
gi|53714949|ref|YP_100941.1|	glucose-1-phosphate thymidylyltransferase	39.3	NO
gi|53714950|ref|YP_100942.1|	undecaprenyl-phosphate alpha-N-acetylglucosaminyltransferase	49.7	NO
gi|53714951|ref|YP_100943.1|	conserved hypothetical protein UpxZ homolog	51.4	NO
**gi|53714952|ref|YP_100944.1|**	**putative transcriptional regulator UpxY homolog**	**51.7**	NO
**Locus 9**			

gi|53715195|ref|YP_101187.1|	putative undecaprenyl-phosphate galactose phosphotransferase	39.3	NO
gi|53715196|ref|YP_101188.1|	aminotransferase	44.2	UNRESOLVED
**gi|53715197|ref|YP_101189.1|**	**hypothetical protein**	**32.8**	LGT-in
gi|53715198|ref|YP_101190.1|	putative glycosyltransferase	36.5	UNRESOLVED
gi|53715199|ref|YP_101191.1|	putative glycosyltransferase	32.5	LGT-unresolved
gi|53715200|ref|YP_101192.1|	hypothetical protein	30.2	NOVEL
gi|53715201|ref|YP_101193.1|	putative glycosyltransferase	32.4	NO
gi|53715202|ref|YP_101194.1|	putative polysaccharide polymerase	31.9	NOVEL
gi|53715203|ref|YP_101195.1|	putative glycosyltransferase	33.6	UNRESOLVED
gi|53715204|ref|YP_101196.1|	putative UDP-glucose dehydrogenase	39.1	NO
gi|53715205|ref|YP_101197.1|	putative UDP-glucuronic acid epimerase	39.0	NO
gi|53715206|ref|YP_101198.1|	probable mannosyltransferase	31.0	UNRESOLVED
gi|53715207|ref|YP_101199.1|	putative O-antigen repeat unit transporter	30.7	UNRESOLVED
gi|53715208|ref|YP_101200.1|	conserved hypothetical protein UpxZ homolog	46.3	NO
**gi|53715209|ref|YP_101201.1|**	**putative transcriptional regulator Updx homolog**	**46.5**	NO

Legend: Shown are Gene ID, annotated function, GC content (%) and the predicted evolutionary history of the coding sequence. Code: NOVEL, no homologs found in any other genomes in public databases; NO, not laterally transferred; UNRESOLVED, whether laterally transferred or not is not resolved; LGT-in, laterally transferred into the genome; LGT-out, laterally transferred out of the genome; LGT-unresolved, laterally transferred but direction unknown. See **Materials and Methods** for detailed explanations. Color codes are the same as in **Figure 4B**.
