# Supplementary material for: Evolution of Symbiotic Bacteria in the Distal Human Intestine
Source: PLoS Biol. 2007 Jun 19;5(7):e156. doi: 10.1371/journal.pbio.0050156 (PMC1892571; doi:10.1371/journal.pbio.0050156)
Supplement: Supplementary file 10 [file pbio.0050156.st006.pdf]

**Table S6.** ***CPS*** **loci are among the most polymorphic regions in the two** ***B. fragilis*** **genomes.**

| | ***B. fragilis*** **NCTC 9343** | ***B. fragilis*** **YCH 46** |
|---|---|---|
| **Number of genes in genome** | 4189 | 4578 |
| **Number of genes in** ***CPS*** **loci** | 170 | 204 |
| **Number of gene loss/gain events** | 658 | 1047 |
| **Number of gene loss/gain events in** ***CPS*** **loci** | 77 | 109 |
| **Probability** | 8.8E-20 | 3.3E-21 |

**Legend**: The probability value is based on the tail probability of a binomial distribution. Gene loss/gain events are counted as the difference between the total number of genes in a given strain and the total number of genes shared between the two strains (3,531 in total).
