# Supplementary material for: Evolution of Symbiotic Bacteria in the Distal Human Intestine
Source: PLoS Biol. 2007 Jun 19;5(7):e156. doi: 10.1371/journal.pbio.0050156 (PMC1892571; doi:10.1371/journal.pbio.0050156)
Supplement: Supplementary file 11 [file pbio.0050156.st007.pdf]

Table S7. ECF-σ factor-containing polysaccharide utilization gene clusters in *B. distasonis* ATCC 8503 (A) and *B. vulgatus* ATCC 8482 (B).

(A). ECF-σ factor-containing polysaccharide utilization gene clusters in *B. distasonis* ATCC 8503

**Cluster 1**		
Bd0229	putative RNA polymerase ECF-type sigma factor	UNRESOLVED
Bd0230	putative anti-sigma factor	UNRESOLVED
Bd0231	putative outer membrane protein, probably involved in nutrient binding	NO
Bd0232	putative outer membrane protein probably involved in nutrient binding	UNRESOLVED
Bd0233	conserved hypothetical protein	NO
Bd0234	glycoside hydrolase family 38, distantly related to alpha-mannosidases	NO
Bd0235	putative sodium-dependent transporter	NO
Bd0236	helicase domain protein	LGT-in
Bd0237	conserved hypothetical protein	LGT-in
Bd0238	conserved hypothetical protein	LGT-in
Bd0239	putative exonuclease	NOVEL
Bd0240	conserved hypothetical protein	NO
Bd0241	putative acetyltransferase	NOVEL
**Cluster 2**		
Bd1126c	two-component system sensor histidine kinase	NO
Bd1127c	conserved hypothetical protein	UNRESOLVED
Bd1128c	probable NADH-dependent dehydrogenase	NO
Bd1129c	oxidoreductase, Gfo/Idh/MocA family	NO
Bd1130c	putative arylsulfatase	NO
Bd1131c	putative outer membrane protein, probably involved in nutrient binding	NO
Bd1132c	putative outer membrane protein, probably involved in nutrient binding	NO
Bd1133c	putative anti-sigma factor	NO
Bd1134c	putative RNA polymerase ECF-type sigma factor	NO
**Cluster 3**		
Bd1642c	mucin-desulfating sulfatase	NO
Bd1643c	arylsulfatase A	LGT-unresolved
Bd1644c	putative outer membrane protein, probably involved in nutrient binding	NO
Bd1645c	putative outer membrane protein, probably involved in nutrient binding	NO
Bd1646c	putative anti-sigma factor	NO
Bd1647	conserved hypothetical protein	NO
Bd1648c	RNA polymerase ECF-type sigma factor	NO
**Cluster 4**		
Bd2026	RNA polymerase ECF-type sigma factor	NO
Bd2027	putative anti-sigma factor	NO
Bd2028	putative outer membrane protein probably involved in nutrient binding	NO
Bd2029	putative outer membrane protein probably involved in nutrient binding	NO
Bd2030	putative sulfatase	NO
Bd2031	arylsulfatase A	UNRESOLVED
Bd2032	putative aminotransferase	NO
Bd2033	peptidyl-prolyl cis-trans isomerase SlyD, FKBP-type	NO
Bd2034	chorismate synthase	NO
Bd2035	putative multidrug resistance protein	LGT-in
**Cluster 5**		
Bd2259c	conserved hypothetical protein	NO
Bd2260c	arylsulfatase A	LGT-in
Bd2261c	putative outer membrane protein probably involved in nutrient binding	NO
Bd2262c	putative outer membrane protein probably involved in nutrient binding	NO

Bd2263c	putative anti-sigma factor	NO
Bd2264c	RNA polymerase ECF-type sigma factor	NO
**Cluster 6**		
Bd2265c	putative secreted sulfatase precursor	UNRESOLVED
Bd2266c	arylsulfatase A	UNRESOLVED
Bd2267c	arylsulfatase A	LGT-unresolved
Bd2268c	conserved hypothetical protein, probably involved in nutrient binding	NO
Bd2269c	putative outer membrane protein, probably involved in nutrient binding	NO
Bd2270c	putative anti-sigma factor	NO
Bd2271c	RNA polymerase ECF-type sigma factor	NO
**Cluster 7**		
Bd2277c	putative 1-acyl-sn-glycerol-3-phosphate acyltransferase	NO
Bd2278c	glycoside hydrolase family 92, related to an ill-defined alpha-1,2-mannosidase	UNRESOLVED
Bd2279c	putative outer membrane protein, probably involved in nutrient binding	NO
Bd2280c	putative outer membrane protein, probably involved in nutrient binding	UNRESOLVED
Bd2281c	putative anti-sigma factor	NO
Bd2282	RNA polymerase ECF-type sigma factor	UNRESOLVED
**Cluster 8**		
Bd2405c	hypothetical protein	NOVEL
Bd2406c	conserved hypothetical protein	LGT-unresolved
Bd2407c	conserved hypothetical protein	UNRESOLVED
Bd2408c	glycoside hydrolase family 28, related to polygalacturonases	UNRESOLVED
Bd2409c	putative outer membrane protein, probably involved in nutrient binding	UNRESOLVED
Bd2410c	putative outer membrane protein, probably involved in nutrient binding	UNRESOLVED
Bd2411c	putative anti-sigma factor	UNRESOLVED
Bd2412c	RNA polymerase ECF-type sigma factor	UNRESOLVED
**Cluster 9**		
Bd2413c	putative dehydrogenase	NO
Bd2414c	putative glycosylhydrolase (putative secreted protein)	UNRESOLVED
Bd2415c	putative outer membrane protein, probably involved in nutrient binding	NO
Bd2416c	putative outer membrane protein, probably involved in nutrient binding	NO
Bd2417c	putative anti-sigma factor	NO
Bd2418	RNA polymerase ECF-type sigma factor	NO
**Cluster 10**		
Bd3036	RNA polymerase ECF-type sigma factor	UNRESOLVED
Bd3037	putative anti-sigma factor	UNRESOLVED
Bd3038	putative outer membrane protein probably involved in nutrient binding	NO
Bd3039	putative outer membrane protein, probably involved in nutrient binding	UNRESOLVED
Bd3040	conserved hypothetical protein with endonuclease/exonuclease/phosphatase family domain	UNRESOLVED
Bd3041	conserved hypothetical protein with endonuclease/exonuclease/phosphatase family domain	NO
**Cluster 11**		
Bd3042	RNA polymerase ECF-type sigma factor	UNRESOLVED
Bd3043	putative anti-sigma factor	NO
Bd3044	putative outer membrane protein probably involved in nutrient binding	NO
Bd3045	putative outer membrane protein probably involved in nutrient binding	NO
Bd3046	conserved hypothetical protein	LGT-in
Bd3047	hypothetical protein	NOVEL
Bd3048	glycoside hydrolase family 2, candidate beta-glycosidase	UNRESOLVED
Bd3049c	glycoside hydrolase family 2, candidate beta-glycosidase	NO
Bd3050	conserved hypothetical protein	NO
Bd3051	putative transmembrane protein	UNRESOLVED

**Cluster 12**		
Bd3052	RNA polymerase ECF-type sigma factor	NO
Bd3053	putative anti-sigma factor	NO
Bd3054	putative outer membrane protein, probably involved in nutrient binding	NO
Bd3055	putative outer membrane protein, probably involved in nutrient binding	NO
Bd3056	conserved hypothetical protein	LGT-unresolved
Bd3057	glycoside hydrolase family 97, related to alpha-glucosidases	UNRESOLVED
Bd3058	putative lysophospholipase L1 and related esterase	NO
**Cluster 13**		
Bd3059	RNA polymerase ECF-type sigma factor	NO
Bd3060	putative anti-sigma factor	UNRESOLVED
Bd3061	putative outer membrane protein, probably involved in nutrient binding	UNRESOLVED
Bd3062	putative outer membrane protein, probably involved in nutrient binding	UNRESOLVED
Bd3063	conserved hypothetical protein	UNRESOLVED
Bd3064	putative integrase/transposase	UNRESOLVED
Bd3065	conserved hypothetical protein	UNRESOLVED
Bd3066	hypothetical protein	UNRESOLVED
Bd3067	glycoside hydrolase family 78, related to alpha-L-rhamnosidases	NO
Bd3068	glycoside hydrolase family 92, related to an ill-defined alpha-1,2-mannosidase	NO
**Cluster 14**		
Bd3260c	putative exported protein	NO
Bd3261c	conserved hypothetical protein	UNRESOLVED
Bd3262c	conserved hypothetical protein	NO
Bd3263c	putative outer membrane protein, probably involved in nutrient binding	NO
Bd3264c	putative outer membrane protein, probably involved in nutrient binding	NO
Bd3265c	putative anti-sigma factor	NO
Bd3266c	putative ECF-type RNA polymerase sigma factor	NO
**Cluster 15**		
Bd3386	putative RNA polymerase ECF-type sigma factor	NO
Bd3387	conserved hypothetical protein	NO
Bd3388	hypothetical protein	LGT-unresolved
Bd3389	putative outer membrane protein probably involved in nutrient binding	NO
Bd3390	putative outer membrane protein probably involved in nutrient binding	UNRESOLVED
Bd3391	conserved hypothetical protein	LGT-in
Bd3392	hypothetical protein	NOVEL
Bd3393c	putative permease	NO
**Cluster 16**		
Bd3859	RNA polymerase ECF-type sigma factor	NO
Bd3860	putative anti-sigma factor	NO
Bd3861	putative outer membrane protein, probably involved in nutrient binding	NO
Bd3862	putative outer membrane protein, probably involved in nutrient binding	UNRESOLVED
Bd3863	arylsulfatase precursor	UNRESOLVED
Bd3864	N-acetylgalactosamine 6-sulfatase (GALNS)	UNRESOLVED

(B). ECF-σ factor-containing polysaccharide utilization gene clusters in *B. vulgatus* ATCC 8482

**Cluster 1**		
Bv0103c	hypothetical protein	NOVEL
Bv0104c	conserved hypothetical protein, possible ATP/GTP-binding site	UNRESOLVED
Bv0105c	putative oxidoreductase	NO
Bv0106c	putative outer membrane protein, probably involved in nutrient binding	NO
Bv0107c	putative outer membrane protein, probably involved in nutrient binding	NO
Bv0108c	putative anti-sigma factor	NO
Bv0109c	RNA polymerase ECF-type sigma factor	NO
**Cluster 2**		
Bv0110c	putative thiol:disulfide interchange protein DsbE	NO
Bv0111c	putative thiol:disulfide interchange protein	NO
Bv0112c	conserved hypothetical protein	NO
Bv0113c	conserved hypothetical protein	NO
Bv0114c	putative regulatory protein	NO
Bv0115c	glycoside hydrolase family 88, candidate delta-4,5 unsaturated glucuronyl hydrolase	NO
Bv0116c	glycoside hydrolase family 43, candidate beta-xylosidase/alpha-L-arabinofuranosidase	NO
Bv0117c	glycoside hydrolase family 97, related to alpha-glucosidases	NO
Bv0118c	conserved hypothetical protein	NO
Bv0119c	putative beta-lactamase class C and other penicillin binding proteins	LGT-in
Bv0120c	conserved hypothetical protein	NO
Bv0121c	glycoside hydrolase family 28, distantly related to polygalacturonases	LGT-out
Bv0122c	conserved hypothetical protein	NO
Bv0123c	conserved hypothetical protein	NO
Bv0124c	glycoside hydrolase family 28, distantly related to polygalacturonases	NO
Bv0125c	putative outer membrane protein, probably involved in nutrient binding	NO
Bv0126c	putative outer membrane protein, probably involved in nutrient binding	NO
Bv0127c	RNA polymerase ECF-type sigma factor	NO
**Cluster 3**		
Bv0132c	glycoside hydrolase family 92, related to an ill-defined alpha-1,2-mannosidase	UNRESOLVED
Bv0133c	glycoside hydrolase family 97, related to alpha-glucosidases	NO
Bv0134c	putative outer membrane protein, probably involved in nutrient binding	UNRESOLVED
Bv0135c	putative outer membrane protein, probably involved in nutrient binding	NO
Bv0136c	putative RNA polymerase ECF-type sigma factor	UNRESOLVED
**Cluster 3**		
Bv0293c	glycoside hydrolase family 2, related to beta-galactosidases	UNRESOLVED
Bv0294c	glycoside hydrolase family 63, distantly related to alpha-glycosidases	UNRESOLVED
Bv0295c	putative outer membrane protein, probably involved in nutrient binding	UNRESOLVED
Bv0296c	putative outer membrane protein, probably involved in nutrient binding	NO
Bv0297c	putative anti-sigma factor	NO
Bv0298c	putative RNA polymerase ECF-type sigma factor	NO
**Cluster 4**		
Bv0342c	histidyl-tRNA synthetase	NO
Bv0343c	putative ABC transporter, periplasmic sugar-binding protein	LGT-in
Bv0344c	transposase	UNRESOLVED
Bv0345c	ABC-type sugar transport system, periplasmic component	LGT-in
Bv0346c	putative integral membrane protein	NO
Bv0347c	conserved hypothetical protein	LGT-unresolved
Bv0348c	glycerol kinase 2 (ATP:glycerol 3-phosphotransferase 2)	LGT-unresolved
Bv0349c	transketolase, C-terminal subunit	LGT-unresolved
Bv0350c	transketolase, N-terminal subunit	LGT-unresolved
Bv0351c	transcriptional regulator of sugar metabolism	LGT-in

Bv0352c	3,4-dihydroxy-2-butanone 4-phosphate synthase (cyclohydrolase II)	LGT-unresolved
Bv0353c	conserved hypothetical protein	LGT-unresolved
Bv0354c	two-component system sensor histidine kinase/response regulator, hybrid ('one-component system')	NO
Bv0355c	putative outer membrane protein, probably involved in nutrient binding	UNRESOLVED
Bv0356c	putative outer membrane protein, probably involved in nutrient binding	NO
Bv0357c	conserved hypothetical protein, putative anti-sigma factor	NO
Bv0358c	RNA polymerase ECF-type sigma factor	NO
**Cluster 5**		
Bv0377	putative RNA polymerase ECF-type sigma factor	NO
Bv0378	putative anti-sigma factor	NO
Bv0379	putative outer membrane protein, probably involved in nutrient binding	NO
Bv0380	putative outer membrane protein, probably involved in nutrient binding	NO
Bv0381	glycoside hydrolase family 2, candidate beta-glycosidase	UNRESOLVED
Bv0382	glycoside hydrolase family 20, distantly related to beta-N-acetylhexosaminidases	LGT-unresolved
Bv0383	glycoside hydrolase family 2, candidate beta-glycosidase	NO
Bv0384	arylsulfatase	NO
Bv0385	hypothetical protein	LGT-in
Bv0386	NADH-ubiquinone oxidoreductase subunit	NOVEL
Bv0387	hypothetical protein	NOVEL
**Cluster 6**		
Bv0472c	conserved hypothetical protein	UNRESOLVED
Bv0473c	glycoside hydrolase family 31, candidate alpha-glycosidase; related to beta-	NO
Bv0474c	putative outer membrane protein, probably involved in nutrient binding	NO
Bv0475c	putative outer membrane protein, probably involved in nutrient binding	NO
Bv0476c	two-component system response regulator	NO
Bv0477c	glycoside hydrolase family 35, candidate beta-glycosidase; related to beta-galactosidases	NO
Bv0478c	glycoside hydrolase family 51, related to alpha-L-arabinofuranosidases	NO
Bv0479c	glycoside hydrolase family 43, modular protein with N-terminal domain distantly related to beta-glycosidases and C-terminal related to beta-xylosidases/alpha-L-arabinofuranosidases	UNRESOLVED
Bv0480c	glycoside hydrolase family 30, candidate beta-glycosidase	NO
Bv0481c	putative outer membrane protein, probably involved in nutrient binding	NO
Bv0482c	putative outer membrane protein, probably involved in nutrient binding	NO
Bv0483c	putative anti-sigma factor	NO
Bv0484c	putative RNA polymerase ECF-type sigma factor	NO
**Cluster 7**		
Bv0593c	putative oxidoreductase	NO
Bv0594c	conserved hypothetical protein	NO
Bv0595c	putative signal transducer	NO
Bv0596c	conserved hypothetical protein	NO
Bv0597c	exo-alpha sialidase	NO
Bv0598c	conserved hypothetical protein	NO
Bv0599c	glycoside hydrolase family 18, related to chitinases	NO
Bv0600c	conserved hypothetical protein	NO
Bv0601c	putative outer membrane protein, probably involved in nutrient binding	NO
Bv0602c	glycoside hydrolase family 97, related to alpha-glucosidases	NO
Bv0603c	putative endonuclease/exonuclease/phosphatase family protein	NO
Bv0604c	conserved hypothetical protein	NO
Bv0605c	putative outer membrane protein, probably involved in nutrient binding	NO
Bv0606c	glycoside hydrolase family 92, related to an ill-defined alpha-1,2-mannosidase	NO
Bv0607c	glycoside hydrolase family 92, related to an ill-defined alpha-1,2-mannosidase	NO

Bv0608c	putative anti-sigma factor	NO
Bv0609c	RNA polymerase ECF-type sigma factor	NO
**Cluster 8**		
Bv0709c	hypothetical protein	NOVEL
Bv0710c	conserved hypothetical protein	NO
Bv0711c	conserved hypothetical protein	LGT-in
Bv0712c	putative outer membrane protein, probably involved in nutrient binding	NO
Bv0713c	putative outer membrane protein, probably involved in nutrient binding	NO
Bv0714c	putative anti-sigma factor	NO
Bv0715c	RNA polymerase ECF-type sigma factor	NO
Bv0716c	glycoside hydrolase family 36, candidate alpha-glycosidase; related to alpha-galactosidases	NO
**Cluster 9**		
Bv0915c	RNA polymerase ECF-type sigma factor	NO
Bv0916	putative anti-sigma factor	NO
Bv0917	putative outer membrane protein, probably involved in nutrient binding	NO
Bv0918	putative outer membrane protein, probably involved in nutrient binding	UNRESOLVED
Bv0919	conserved hypothetical protein	NO
Bv0920	conserved hypothetical protein	NO
Bv0921	putative oxidoreductase (putative secreted protein)	LGT-unresolved
**Cluster 10**		
Bv1025	RNA polymerase ECF-type sigma factor	NO
Bv1026	putative anti-sigma factor	NOVEL
Bv1027	putative outer membrane protein, probably involved in nutrient binding	NO
Bv1028	hypothetical protein	NO
Bv1029c	erythronate-4-phosphate dehydrogenase	NO
Bv1030	glycosyltransferase family 9, related to glycosyltransferases	NO
Bv1031	putative acetyltransferase	NO
Bv1032	putative LPS biosynthesis related UDP-galactopyranose mutase	NO
Bv1033	conserved hypothetical protein	NO
Bv1034	conserved hypothetical protein	UNRESOLVED
Bv1035	glycosyltransferase family 2, related to beta-glycosyltransferases	NO
Bv1036	glycosyltransferase family 2, distantly related to beta-glycosyltransferases	UNRESOLVED
Bv1037	glycosyltransferase family 14, related to beta-glycosyltransferases	NO
Bv1038	glycosyltransferase family 4, related to alpha-glycosyltransferases	NO
**Cluster 11**		
Bv1124	putative ECF sigma factor	NO
Bv1125	putative membrane protein	NO
Bv1126	putative outer membrane protein, probably involved in nutrient binding	NO
Bv1127	putative outer membrane protein, probably involved in nutrient binding	NO
Bv1128	sulfatase	LGT-unresolved
Bv1129	arylsulfatase precursor	NO
Bv1130	putative ATP-binding ABC transporter protein	NO
**Cluster 12**		
Bv1663c	RNA polymerase ECF-type sigma factor	NO
Bv1664	putative anti-sigma factor	NO
Bv1665	putative outer membrane protein, probably involved in nutrient binding	NO
Bv1666	putative outer membrane protein, probably involved in nutrient binding	UNRESOLVED
Bv1667	glycerophosphoryl diester phosphodiesterase	NO
**Cluster 13**		
Bv1721c	glycoside hydrolase family 28, related to polygalacturonases	UNRESOLVED
Bv1722c	glycoside hydrolase family 2, candidate beta-glycosidase	NO
Bv1723c	glycoside hydrolase family 28, related to polygalacturonases	UNRESOLVED

Bv1724	hypothetical protein	NOVEL
Bv1725c	iduronate 2-sulfatase precursor	NO
Bv1726c	putative outer membrane protein, probably involved in nutrient binding	NO
Bv1727c	putative outer membrane protein, probably involved in nutrient binding	NO
Bv1728c	putative anti-sigma factor	NO
Bv1729	RNA polymerase ECF-type sigma factor	NO
**Cluster 14**		
Bv1733	putative ECF-type RNA polymerase sigma factor	NO
Bv1734	putative anti-sigma factor	NO
Bv1735	putative outer membrane protein, probably involved in nutrient binding	NO
Bv1736	putative outer membrane protein, probably involved in nutrient binding	NO
Bv1737	conserved hypothetical protein	NO
Bv1738	conserved hypothetical protein	NO
Bv1739	glycoside hydrolase family 2, candidate beta-glycosidase	UNRESOLVED
Bv1740	hypothetical protein	NOVEL
Bv1741	glycoside hydrolase family 78, distantly related to alpha-L-rhamnosidases	UNRESOLVED
Bv1742	two-component system response regulator	NO
Bv1743	aldehyde dehydrogenase A	LGT-unresolved
Bv1744	glycoside hydrolase family 43, related to beta-xylosidases/alpha-L-	NO
Bv1745	conserved hypothetical protein	NO
Bv1746	conserved hypothetical protein	NO
Bv1747	conserved hypothetical protein	NO
**Cluster 15**		
Bv1758	putative RNA polymerase ECF-type sigma factor	NO
Bv1759	putative anti-sigma factor	NO
Bv1760	putative outer membrane protein, probably involved in nutrient binding	NO
Bv1761	putative outer membrane protein, probably involved in nutrient binding	NO
Bv1762	glycoside hydrolase family 2, candidate beta-glycosidase	LGT-out
Bv1763	glycoside hydrolase family 2, candidate beta-glycosidase	NO
Bv1764	glycoside hydrolase family 31, candidate alpha-glycosidase	UNRESOLVED
Bv1765	conserved hypothetical protein	UNRESOLVED
Bv1766c	putative pectin degradation protein	LGT-in
Bv1767	polysaccharide lyase family 10, related to pectate lyases	LGT-in
Bv1768	conserved hypothetical protein	NO
Bv1769	carbohydrate esterase family 8, modular protein with N-terminal domain distantly related to pectin acetylesterases and C-terminal domain related to pectin methylesterases	NO
Bv1770	two-component system sensor histidine kinase/response regulator, hybrid ('one-component system')	NO
Bv1771	hypothetical protein	NO
Bv1772	dipeptidyl peptidase IV	NO
**Cluster 16**		
Bv1927c	putative thiol-disulfide oxidoreductase	UNRESOLVED
Bv1928c	putative disulphide-isomerase	NO
Bv1929c	putative outer membrane protein, probably involved in nutrient binding	UNRESOLVED
Bv1930c	putative outer membrane protein, probably involved in nutrient binding	UNRESOLVED
Bv1931c	putative anti-sigma factor	NO
Bv1932c	RNA polymerase ECF-type sigma factor	UNRESOLVED
Bv1933c	glycoside hydrolase family 43, candidate beta-xylosidase/alpha-L-arabinofuranosidase	NO
Bv1934c	conserved hypothetical protein	NO
Bv1935c	glycoside hydrolase family 28, related to polygalacturonases	NO
**Cluster 17**		
Bv1972	RNA polymerase ECF-type sigma factor	NO

Bv1973	putative anti-sigma factor	NO
Bv1974	putative outer membrane protein, probably involved in nutrient binding	UNRESOLVED
Bv1975	putative outer membrane protein, probably involved in nutrient binding	UNRESOLVED
Bv1976	hypothetical protein	NOVEL
Bv1977	ABC transporter ATP-binding protein	NO
Bv1978	putative endothelin-converting enzyme	NO
Bv1979	conserved hypothetical protein	NO
Bv1980	putative metal resistance related exported protein	NO
Bv1981	AcrB/AcrD/AcrF family cation efflux system protein	NO
Bv1982	conserved hypothetical protein	NO
Bv1983	ThiJ/PfpI family protein	NO
Bv1984	putative nitroreductase	NO
**Cluster 18**		
Bv2160	RNA polymerase ECF-type sigma factor	UNRESOLVED
Bv2161	putative anti-sigma factor	NO
Bv2162	putative outer membrane protein, probably involved in nutrient binding	NO
Bv2163	putative outer membrane protein, probably involved in nutrient binding	UNRESOLVED
**Bv2164**	putative thiol-disulfide oxidoreductase	UNRESOLVED
Bv2165	putative thiol-disulfide oxidoreductase	UNRESOLVED
Bv2166	glycoside hydrolase family 2, candidate beta-glycosidase	NO
Bv2167	aldose 1-epimerase precursor	NO
**Cluster 19**		
Bv2384c	L-serine dehydratase	NO
Bv2385c	conserved hypothetical protein	NO
Bv2386c	putative outer membrane protein, probably involved in nutrient binding	NO
Bv2387c	putative outer membrane protein, probably involved in nutrient binding	NO
Bv2388c	putative anti-sigma factor	NO
Bv2389c	RNA polymerase ECF-type sigma factor	UNRESOLVED
**Cluster 20**		
Bv4006c	conserved hypothetical protein	NO
Bv4007c	putative outer membrane protein, probably involved in nutrient binding	NO
Bv4008c	putative outer membrane protein, probably involved in nutrient binding	NO
Bv4009c	putative anti-sigma factor	NO
Bv4010c	RNA polymerase ECF-type sigma factor	NO

Legend: The three columns represent Gene ID, functional annotation and predicted evolutionary history of the gene (labeled as in **Table S5**).
