# Supplementary material for: Evolution of Symbiotic Bacteria in the Distal Human Intestine
Source: PLoS Biol. 2007 Jun 19;5(7):e156. doi: 10.1371/journal.pbio.0050156 (PMC1892571; doi:10.1371/journal.pbio.0050156)
Supplement: Supplementary file 12 [file pbio.0050156.sd001.doc]

**Supporting Information**

**Overview of strategy used to identify lateral gene transfer**

Many approaches have been used to detect LGT for different applications. For example, compositional methods based on GC content, dinucleotide frequencies, codon usage, and other oligonucleotide frequencies (e.g., [1-5]) are often used to detect transferred genes within a genome. Techniques such as patterns of BLAST hits (e.g., [6-8]) and ratios of sequence divergence between different pairs of genes in different pairs of species (e.g., [9-12]) have also been used in numerous studies. However, these methods for detecting lateral gene transfer are typically seen as surrogates for phylogenetic studies [13], and their sensitivity and specificity have often been criticized. For example, compositional methods are sensitive to equilibration of the gene composition to the genome composition [14-16], and BLAST-based methods are sensitive to loss of paralogs, different rates of evolution, recombination, and many other factors [13,17-21]. Indeed, phylogenetic re-analysis of putative laterally transferred genes that were originally found by reciprocal BLAST hits has indicated that many of these identifications were incorrect (e.g., [22,23]). Similarly, application of parsimony inference to identify gene losses and gains on a species or rRNA tree (e.g., [24-27]) is sensitive to the presence of paralogous sequences and other artifacts [19]. Thus, phylogenetic analysis of individual gene trees, often manually applied, is frequently recommended as the gold standard for lateral gene transfer detection [12,13,17,18,23,28-30].

Phylogenetic analysis itself is subject to many issues, especially because constructing the tree relies on models of sequence evolution that may be simplified or incorrect [31,32], because many protein families lack phylogenetic signal [33], or because of artifacts of tree reconstruction (e.g., [34,35]. There are several formal statistical tests for tree congruence, such as the KH test [36], but these tests require that the two trees to be compared contain the same taxa (i.e. multiple sequences from the same taxon are not allowed). Because lineage-specific duplications in the Bacteroidetes are common, as shown by our analysis (see **Results**) and previous studies [37-39], applying these tests in an automated fashion becomes exceedingly complex. In particular, choices about which duplicate genes to omit affect the values of the test statistics. Additionally, these tests only measure differences in tree topology, and cannot typically distinguish lateral gene transfer from ancient loss of paralogs or other unusual phylogenetic events. Thus, although the SH test has been extremely useful for identifying genes that lead to poor resolution in whole-genome phylogenies [19], it is less suitable for asking which genes in a genome have undergone lateral gene transfer. Similar comments apply to other global tests for changes in tree topology (e.g., [40-42]) because they cannot handle duplications in any lineage, they cannot be reliably applied on a genome-wide scale. For example, Ge and colleagues were able to find only 297 orthologous gene clusters across 40 species that were suitable for application of their method [41].

Because different methods for detecting lateral gene transfer typically have poor agreement about which genes are detected as transferred [9,12,13,30,43], we decided to use phylogenetic analysis and to focus on the types of transfers it detects best: transfers from within one specific lineage to another specific lineage. In particular, because our 16S rRNA trees showed that the gut Bacteroidetes are well-supported as a monophyletic group, we decided to focus on those genes that were transferred from a specific lineage outside this group to individual species within this group. Since we are in the process of gathering more genomic sequences from Bacteroidetes, we left study of lateral gene transfer within the gut Bacteroidetes for future work because we expect substantially better resolution for detecting lateral gene transfer events when better taxonomic sampling within this group is available.

Our goal was to automatically assign taxon labels to the sequences in individual gene trees, such that unknown sequences would acquire labels from their close relatives in a consistent fashion. In order to achieve this outcome, we treated each taxon label as a character state, and inferred the ancestral state at each node using the Fitch parsimony algorithm [44]. For example, taxon labels might correspond to bacterial divisions, such as "Firmicutes", or to other taxonomic groups of interest, such as "gut Bacteroidetes" or "non-gut Bacteroidetes". This procedure, which has often been applied either manually or in an automated fashion to reveal lateral gene transfer among specific lineages (e.g., [45-47]), is based on the idea that a lateral gene transfer event followed by speciation should typically be marked by a monophyletic group of sequences from one lineage that

stems from within a paraphyletic group of sequences from a single other lineage. In other words, the transfer of a gene from lineage X to lineage Y should give a tree in which the sequences from Y are related to a specific group of sequences within lineage X. We would still count this event as a transfer from X to Y even if there are more sequences in Y than remain in X, for example if Y is a very speciose lineage or is a lineage in which paralogy of the relevant genes is rampant. The parsimony approach we used is related to Brooks Parsimony Analysis, a method used for detecting co-speciation between hosts and parasites [48]. The problem of host-parasite co-speciation is mathematically identical to the problem of relating gene trees to species trees, because both cases require the analysis of phylogenies in which duplication, deletion, and switching between hosts (or

genomes) are all possible.

There are two types of events that could conceivably lead to the type of phylogeny in which we are interested (lineage X paraphyletic with respect to lineage Y): lateral transfer from one group to another, and loss of an ancient paralog in all but those two groups. However, because strong selective advantages are required to maintain transferred genes in bacterial populations [49] and because the divergence distances are large (mostly from other bacterial divisions), lateral gene transfer is by far the most likely scenario leading to these trees. We believe that this method is more suitable for detecting a set of high-confidence transfers because global measures of phylogenetic incongruence require rejection over the whole tree, not just for one specific group (potentially leading to high false negative rates), and are influenced by the many factors that can lead to misplacement of taxa not relevant to our analysis (leading to high false positive rates). We confirmed the likely lateral transfer of these genes by testing that the GC content and codon usage of genes chosen by our method differed from those of randomly chosen genes in the genome. Specifically, we compared the GC contents of transferred and non-transferred genes within each genome (excluding unresolved genes) using two-sample *t* tests with Welch’s correction for unequal variances, and compared the codon usage of transferred and non-transferred genes using chi-squared tests. Analysis of the functional categories represented by these genes and presence of groups of genes within apparent genomic islands provided additional supporting evidence of LGT (see **Results**).

**Supplementary References**

1. Karlin S, Mrazek J, Campbell AM (1998) Codon usages in different gene classes of the Escherichia coli genome. Mol Microbiol 29: 1341-1355.

2. Lawrence JG, Ochman H (1998) Molecular archaeology of the Escherichia coli genome. Proc Natl Acad Sci U S A 95: 9413-9417.

3. Wang HC, Badger J, Kearney P, Li M (2001) Analysis of codon usage patterns of bacterial genomes using the self-organizing map. Mol Biol Evol 18: 792-800.

4. Hooper SD, Berg OG (2002) Detection of genes with atypical nucleotide sequence in microbial genomes. J Mol Evol 54: 365-375.

5. Sandberg R, Branden CI, Ernberg I, Coster J (2003) Quantifying the species-specificity in genomic signatures, synonymous codon choice, amino acid usage and G+C content. Gene 311: 35-42.

6. Nelson KE, Clayton RA, Gill SR, Gwinn ML, Dodson RJ, et al. (1999) Evidence for lateral gene transfer between Archaea and bacteria from genome sequence of Thermotoga maritima. Nature 399: 323-329.

7. Lander ES, Linton LM, Birren B, Nusbaum C, Zody MC, et al. (2001) Initial sequencing and analysis of the human genome. Nature 409: 860-921.

8. Pallen MJ, Beatson SA, Bailey CM (2005) Bioinformatics analysis of the locus for enterocyte effacement provides novel insights into type-III secretion. BMC Microbiol 5: 9.

9. Syvanen M (2002) On the occurrence of horizontal gene transfer among an arbitrarily chosen group of 26 genes. J Mol Evol 54: 258-266.

10. Farahi K, Whitman WB, Kraemer ET (2003) RED-T: utilizing the Ratios of Evolutionary Distances for determination of alternative phylogenetic events. Bioinformatics 19: 2152-2154.

11. Novichkov PS, Omelchenko MV, Gelfand MS, Mironov AA, Wolf YI, et al. (2004) Genome-wide molecular clock and horizontal gene transfer in bacterial evolution. J Bacteriol 186: 6575-6585.

12. Kechris KJ, Lin JC, Bickel PJ, Glazer AN (2006) Quantitative exploration of the occurrence of lateral gene transfer by using nitrogen fixation genes as a case study. Proc Natl Acad Sci U S A 103: 9584-9589.

13. Ragan MA (2001) On surrogate methods for detecting lateral gene transfer. FEMS Microbiol Lett 201: 187-191.

14. Lawrence JG, Ochman H (1997) Amelioration of bacterial genomes: rates of change and exchange. J Mol Evol 44: 383-397.

15. Koski LB, Morton RA, Golding GB (2001) Codon bias and base composition are poor indicators of horizontally transferred genes. Mol Biol Evol 18: 404-412.

16. Wang B (2001) Limitations of compositional approach to identifying horizontally transferred genes. J Mol Evol 53: 244-250.

17. Ochman H (2001) Lateral and oblique gene transfer. Curr Opin Genet Dev 11: 616-619.

18. Doolittle WF, Boucher Y, Nesbo CL, Douady CJ, Andersson JO, et al. (2003) How big is the iceberg of which organellar genes in nuclear genomes are but the tip? Philos Trans R Soc Lond B Biol Sci 358: 39-57; discussion 57-38.

19. Lerat E, Daubin V, Moran NA (2003) From gene trees to organismal phylogeny in prokaryotes: the case of the gamma-Proteobacteria. PLoS Biol 1: E19.

20. Philippe H, Douady CJ (2003) Horizontal gene transfer and phylogenetics. Curr Opin Microbiol 6: 498-505.

21. Andersson JO (2005) Lateral gene transfer in eukaryotes. Cell Mol Life Sci 62: 1182-1197.

22. Kinsella RJ, McInerney JO (2003) Eukaryotic genes in Mycobacterium tuberculosis? Possible alternative explanations. Trends Genet 19: 687-689.

23. Genereux DP, Logsdon JM, Jr. (2003) Much ado about bacteria-to-vertebrate lateral gene transfer. Trends Genet 19: 191-195.

24. Jordan IK, Makarova KS, Spouge JL, Wolf YI, Koonin EV (2001) Lineage-specific gene expansions in bacterial and archaeal genomes. Genome Res 11: 555-565.

25. Mirkin BG, Fenner TI, Galperin MY, Koonin EV (2003) Algorithms for computing parsimonious evolutionary scenarios for genome evolution, the last universal common ancestor and dominance of horizontal gene transfer in the evolution of prokaryotes. BMC Evol Biol 3: 2.

26. Hao W, Golding GB (2004) Patterns of bacterial gene movement. Mol Biol Evol 21: 1294-1307.

27. Pal C, Papp B, Lercher MJ (2005) Horizontal gene transfer depends on gene content of the host. Bioinformatics 21 Suppl 2: ii222-ii223.

28. Eisen JA (2000) Horizontal gene transfer among microbial genomes: new insights from complete genome analysis. Curr Opin Genet Dev 10: 606-611.

29. Kurland CG, Canback B, Berg OG (2003) Horizontal gene transfer: a critical view. Proc Natl Acad Sci U S A 100: 9658-9662.

30. Ragan MA, Harlow TJ, Beiko RG (2006) Do different surrogate methods detect lateral genetic transfer events of different relative ages? Trends Microbiol 14: 4-8.

31. Lio P, Goldman N (1998) Models of molecular evolution and phylogeny. Genome Res 8: 1233-1244.

32. Bollback JP (2002) Bayesian model adequacy and choice in phylogenetics. Mol Biol Evol 19: 1171-1180.

33. Teichmann SA, Mitchison G (1999) Is there a phylogenetic signal in prokaryote proteins? J Mol Evol 49: 98-107.

34. Doolittle WF (1999) Phylogenetic classification and the universal tree. Science 284: 2124-2129.

35. Thornton JW, DeSalle R (2000) Gene family evolution and homology: genomics meets phylogenetics. Annu Rev Genomics Hum Genet 1: 41-73.

36. Kishino H, Hasegawa M (1989) Evaluation of the maximum likelihood estimate of the evolutionary tree topologies from DNA sequence data, and the branching order in hominoidea. J Mol Evol 29: 170-179.

37. Coyne MJ, Weinacht KG, Krinos CM, Comstock LE (2003) Mpi recombinase globally modulates the surface architecture of a human commensal bacterium. Proc Natl Acad Sci U S A 100: 10446-10451.

38. Kuwahara T, Yamashita A, Hirakawa H, Nakayama H, Toh H, et al. (2004) Genomic analysis of Bacteroides fragilis reveals extensive DNA inversions regulating cell surface adaptation. Proc Natl Acad Sci U S A 101: 14919-14924.

39. Xu J, Bjursell MK, Himrod J, Deng S, Carmichael LK, et al. (2003) A genomic view of the human-Bacteroides thetaiotaomicron symbiosis. Science 299: 2074-2076.

40. Daubin V, Ochman H (2004) Quartet mapping and the extent of lateral transfer in bacterial genomes. Mol Biol Evol 21: 86-89.

41. Ge F, Wang LS, Kim J (2005) The cobweb of life revealed by genome-scale estimates of horizontal gene transfer. PLoS Biol 3: e316.

42. Planet PJ, Sarkar IN (2005) mILD: a tool for constructing and analyzing matrices of pairwise phylogenetic character incongruence tests. Bioinformatics 21: 4423-4424.

43. Lawrence JG, Ochman H (2002) Reconciling the many faces of lateral gene transfer. Trends Microbiol 10: 1-4.

44. Fitch WM (1970) Distinguishing homologous from analogous proteins. Syst Zool 20: 406-416.

45. Hooper SD, Berg OG (2003) Duplication is more common among laterally transferred genes than among indigenous genes. Genome Biol 4: R48.

46. Kunin V, Ouzounis CA (2003) GeneTRACE-reconstruction of gene content of ancestral species. Bioinformatics 19: 1412-1416.

47. Daubin V, Ochman H (2004) Bacterial genomes as new gene homes: the genealogy of ORFans in E. coli. Genome Res 14: 1036-1042.

48. Brooks DR (1981) Hennig's parasitological method: a proposed solution. Syst Zool 30: 229-249.

49. Berg OG, Kurland CG (2002) Evolution of microbial genomes: sequence acquisition and loss. Mol Biol Evol 19: 2265-2276.

50. Sonnenburg ED, Sonnenburg JL, Manchester JK, Hansen EE, Chiang HC, et al. (2006) A hybrid two-component system protein of a prominent human gut symbiont couples glycan sensing in vivo to carbohydrate metabolism. Proc Natl Acad Sci U S A 103: 8834-8839.

**Supplemental Figure Legends**

**Figure S1. *B. distasonis* ATCC 8503 (A) and *B. vulgatus* ATCC 8482 (B) chromosomes.** The coding potential of the leading and lagging strands is relatively unbiased. Circles shown in the figure represent, from inside out, GC skew, GC content variation, rRNA operons, tRNA genes, conjugative transposons (CTns), *CPS* loci, extra-cytoplasmic function (ECF) σ factors, SusC paralogs, and all predicted genes with assigned functions on reverse and forward strands, respectively. Color codes for genes are based on their COG functional classification.

**Figure S2. COG-based characterization of all proteins with annotated functions in the proteomes of sequenced Bacteroidetes.** The term, ‘Bacteroides orthologs’ refers to the 1,416 orthologs shared by the sequenced gut Bacteroidetes (*B. vulgatus, B. distasonis, B. thetaiotaomicron,* plus the two *B. fragilis* strains). Color codes are the same as **Figure S1**.

**Figure S3. Pair-wise alignments of the human gut Bacteroidetes genomes reveal rapid deterioration of global synteny with increasing phylogenetic distance**. Each data point on the Dotplot represents one pair of mutual best hits (BLASTP) between the two genomes, plotted by pair-wise genome location. Diagonal lines indicate synteny.

**Figure S4. *CPS* loci are the most polymorphic regions in the gut Bacteroidetes genomes.** High-resolution synteny map of *CPS* loci and flanking regions in the two sequenced *B. fragilis* strains**.** There are 9 *CPS* loci in each genome. Each data point represents a pair of orthologs (mutual best hits; e-value cutoff: 10^-6^). Brackets define the coordinates for component genes within a given locus (some pairs are missing due to gene loss or gain): X-axis, coordinate of the middle point of the gene on the NCTC 9343 chromosome; Y-Axis, coordinate of the middle point of the gene on YCH 46 chromosome. With the exception of *CPS* locus 5, which is strictly conserved, the 9 *CPS* loci are affected by non-homologous gene replacement and rearrangement.

**Supplemental Tables**

**Table S1. Comparison of genome parameters for *B. distasonis* ATCC 8503, *B. vulgatus* ATCC 8482, *B. thetaiotaomicron* ATCC 29148, *B. fragilis* NCTC 9343 and *B. fragilis* YCH 46**. ‘*’, the numbers of SusC/SusD homologs provided are based on BLASTP e-value ≤10^-20^; the numbers shown in parentheses are based on criteria described in *SusC/SusD alignments* in **Materials and Methods**. See <http://gordonlab.wustl.edu/BvBd.html> for complete lists of SusC/SusD homologs. A hybrid two-component system protein contains all of the domains present in classical two-component systems but in one polypeptide [50].

**Table S2. Shared orthologs in *B. distasonis* ATCC 8503, *B. vulgatus* ATCC 8482, *B. thetaiotaomicron* ATCC 29148, and *B. fragilis* strains NCTC 9343 and YCH 46**. For an explanation of COG-based functional codes, see **Figure S1**.

## Table S3. Glycoside hydrolases found in *B. distasonis* ATCC 8503, *B. vulgatus* ATCC 8482, *B. thetaiotaomicron* ATCC 29148 and *B. fragilis* strains NCTC 9343 and YCH 46*.* The classification scheme used is described in the Carbohydrate-Active enZYme (CAZy) database.

**Table S4. List of putative xenologs in *B. distasonis* ATCC 8503 (A), *B. vulgatus* ATCC 8482 (B), *B. thetaiotaomicron* ATCC 29148 (C), *B. fragilis* NCTC 9343 (D), and *B. fragilis* YCH 46 (E)**. For an explanation of COG-based functional codes, see **Figure S1**. The lateral gene transfer (LGT) column defines the predicted evolutionary history of the coding sequence: LGT-in, laterally transferred into the genome; LGT-out, laterally transferred out of the genome; LGT-unresolved, laterally transferred but direction unknown. See **Materials and Methods** for detailed explanations.

**Table S5. *CPS* loci of *B. distasonis* ATCC 8503 (A), *B. vulgatus* ATCC 8482 (B), *B. thetaiotaomicron* ATCC 29148 (C), *B. fragilis* NCTC 9343 (D) and *B. fragilis* YCH 46 (E)**. Shown are Gene ID, annotated function, GC content (%) and the predicted evolutionary history of the coding sequence. Code: NOVEL, no homologs found in any other genomes in public databases; NO, not laterally transferred; UNRESOLVED, whether laterally transferred or not is not resolved; LGT-in, laterally transferred into the genome; LGT-out, laterally transferred out of the genome; LGT-unresolved, laterally transferred but direction unknown. See **Materials and Methods** for detailed explanations. Color codes are the same as in **Figure 4B**.

**Table S6. *CPS* loci are among the most polymorphic regions in the two *B. fragilis* genomes*.*** The P value is based on the tail probability of a binomial distribution. Gene loss/gain events (3,531 in total) are counted as the difference between the total number of genes and the total number of genes shared between the two genomes.

**Table S7. ECF-σ factor-containing polysaccharide utilization gene clusters in *B. distasonis* ATCC 8503 (A) and *B. vulgatus* ATCC 8482 (B).** The three columns represent Gene ID, functional annotation and predicted evolutionary history of the gene (labeled as in **Table S5**).
